# Discovery and characterization of a nickel enzyme family that catalyses intermolecular hydride shuttling

**DOI:** 10.1038/s41557-026-02184-9

**Published:** 2026-06-17

**Authors:** Zoë L. Semersky, Conor G. Raymond, John M. Rossi, Heike Hofstetter, Pranav Rao, Monica E. Neugebauer

**Affiliations:** 1 Department of Biochemistry, University of Wisconsin-Madison, Madison, WI USA; 2 Department of Chemistry, University of Wisconsin-Madison, Madison, WI USA

**Keywords:** Metalloproteins, Biosynthesis, Biochemistry

## Abstract

Nickel-dependent enzymes catalyse incredible transformations. However, few have been discovered so far, limiting our understanding of the role of nickel in nature. Here we develop a bioinformatic pipeline to discover a family of nickel pincer mononucleotide (NPMN)-dependent enzymes with structures that are completely distinct from previously reported NPMN-dependent enzymes. We characterize one family member, NPMN-dependent hydride transferase (NphT), and find that it catalyses intermolecular hydride transfer, which we elucidate through its promiscuous disproportionation of sugars. We solve a 1.3-Å resolution crystal structure of NphT bound to NPMN and interrogate its mechanism through mutagenesis. We discover that NphT is one of many unexplored nickel enzymes within the large aldo-keto reductase superfamily, which catalyses reactions on a wide range of molecules, including secondary metabolites, sugars and drugs. This work reveals a unique enzymatic scaffold that can harness nickel, expands the known NPMN-catalysed transformations to intermolecular hydride transfer and establishes a pipeline for the discovery of distinct families of nickel-dependent enzymes.

**Figure:**
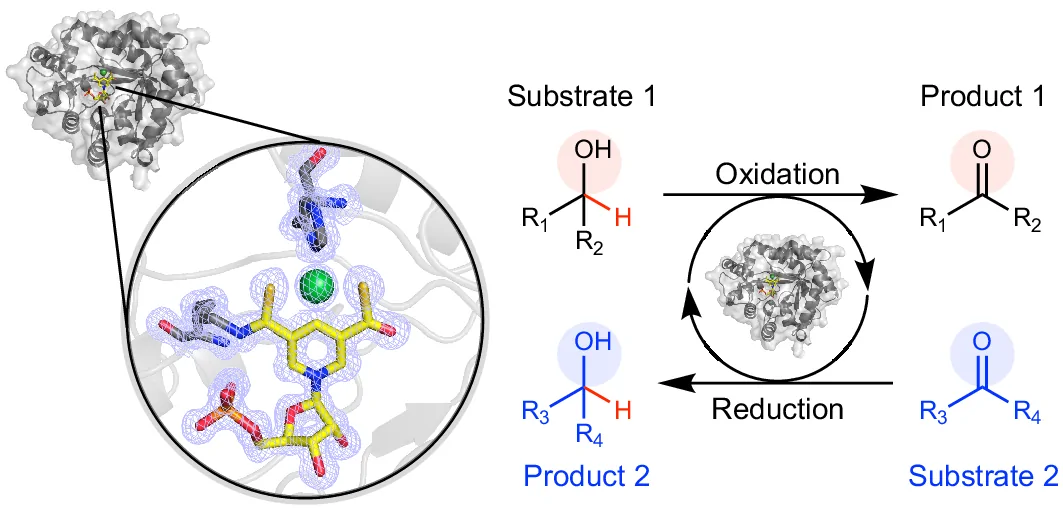

## Abstract (web summary)

There is limited understanding of nickel-catalysed chemistry in nature. Now a bioinformatic pipeline reveals a structurally distinct family of nickel pincer nucleotide-dependent enzymes. Nickel pincer hydride transferase (NphT) was found to catalyse intermolecular hydride transfer by characterizing its crystal structure, conducting structure-guided mutagenesis and measuring its capacity to disproportionate sugars.

## Main

**Fig. 1:**
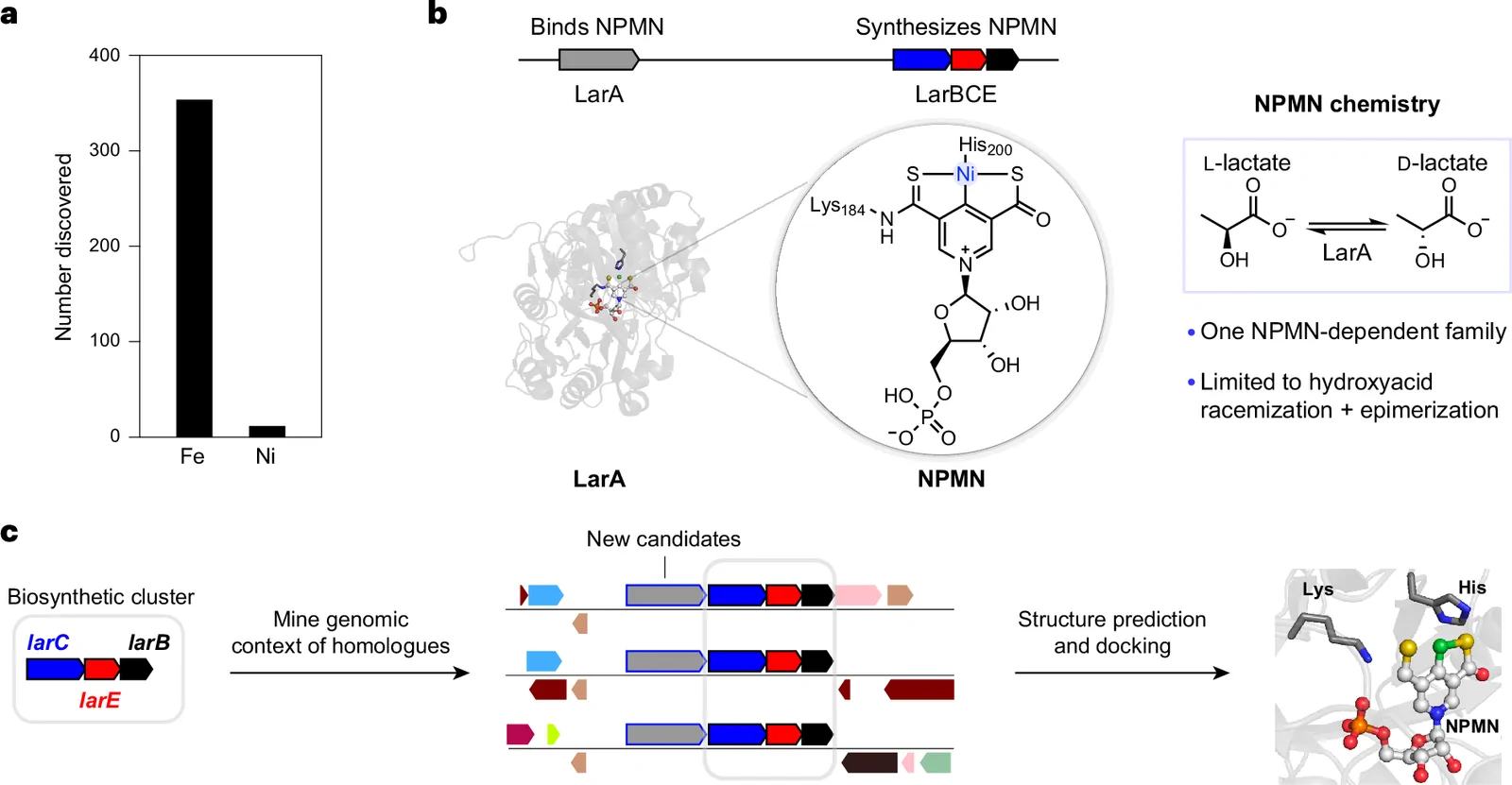
Bioinformatic pipeline for the discovery of nickel pincer-dependent enzymes. **a**, Scarcity of reported nickel-dependent enzymes compared with iron-dependent enzymes. **b**, The lactate racemase, LarA, catalyses lactate racemization using the NPMN cofactor. NPMN is synthesized by LarB, LarC and LarE and then covalently tethered to LarA via a lysine side chain. **c**, Bioinformatic search for pincer-utilizing enzymes using LarB, LarC and LarE as a search query. Genes that co-occur with nickel pincer biosynthesis genes are nominated as candidates that may bind NPMN. Structural prediction and cofactor docking are used to support the nomination of candidates for further experimental investigation. Source data

Metalloenzymes are of significant interest in biology, where they catalyse key reactions that sustain life^1–7^, and in chemistry, where they provide a blueprint for harnessing earth-abundant metals for important chemical transformations under mild conditions^8,9^. The study of nickel-dependent metalloenzymes is particularly valuable: while nickel has emerged as a versatile catalyst for synthetic transformations^10–14^, achieving stereo- and regioselective control with purely synthetic nickel catalysts remains challenging^15^. In nature, nickel-dependent metalloenzymes catalyse incredibly demanding reactions such as methane formation^16^, carbon fixation^17^ and hydrogen evolution^18^, which are central to biogeochemical cycles and bioenergy conversion. However, only 12 nickel enzymes have been discovered so far^19–22^, in contrast to the hundreds of known iron enzymes^23–26^, highlighting a major gap in our understanding of biological nickel chemistry (Fig. 1a). The discovery and characterization of new nickel enzymes would therefore enhance our fundamental understanding of the role of nickel in biology and accelerate the development of nickel catalysts as sustainable alternatives to precious metals such as palladium.

The structural complexity of select nickel cofactors provides a powerful handle for enzyme discovery. Unlike enzymes that simply coordinate nickel, those requiring biosynthetically assembled nickel cofactors could be identified through characteristic biosynthetic gene clusters. One such cofactor is the nickel pincer mononucleotide (NPMN), a unique organometallic nickel cofactor found in the active site of the lactate racemase, LarA^27,28^. NPMN is biosynthesized in three steps by the enzymes LarB, LarC and LarE^29^ and then covalently tethered to a lysine residue in LarA via a thioamide bond^27^ (Fig. 1b). LarA uses NPMN to catalyse proton-coupled hydride transfer between lactate and the resulting pyruvate intermediate to achieve racemization^30^. Since its discovery in 2015, homologues of LarA have been found to use NPMN either covalently or non-covalently but the scope of their known reactions is limited to racemization and epimerization of alpha-hydroxyacids^31–35^. Excitingly, hundreds of prokaryotes contain genes for biosynthesizing NPMN but lack LarA homologues, suggesting that entirely new classes of NPMN-dependent enzymes await discovery^28^. Indeed, an ornithine cyclodeaminase was recently discovered to use a modified nickel pincer adenine dinucleotide (NPAD) cofactor in a non-covalent manner^22^, further highlighting the promise of a bioinformatics-guided approach for discovering unique nickel enzymes. Thus, the identification and characterization of additional nickel enzymes would greatly expand the scope of known enzymes that can harness nickel for catalysis.

Here, we report the bioinformatically guided discovery of a family of NPMN-dependent enzymes with structures and substrates completely distinct from LarA and the ornithine cyclodeaminase. We characterize one family member, NPMN-dependent hydride transferase (NphT), and find that it catalyses intermolecular hydride transfer, which we characterized through its promiscuous activity on five-carbon and six-carbon sugars. NphT is thermostable and catalyses over 1,600 turnovers, highlighting its catalytic potential. To elucidate the structural basis for this reactivity, we solved a 1.3-Å resolution crystal structure of NphT bound to NPMN, revealing that NphT is related to the broad superfamily of aldo-keto reductases (AKRs), which perform NAD(P)(H)-dependent redox reactions on diverse substrates, including steroids, carbohydrates and secondary metabolites^36^. We perform structure-guided mutagenesis of key active site residues and deuterium labelling experiments to probe the mechanism of intermolecular hydride shuttling. Finally, we perform bioinformatic analysis of the AKR superfamily, revealing that unexplored nickel-containing enzymes are broadly distributed throughout this protein fold.

This work uncovers an additional enzyme scaffold that covalently tethers NPMN. Together with the recently discovered ornithine cyclodeaminase^22^, these findings demonstrate that structurally diverse enzymes beyond LarA can harness nickel pincer cofactors for catalysis. Furthermore, the AKR enzyme superfamily exhibits cofactor plasticity: while many AKRs use NAD(P)(H), others have instead evolved to use NPMN. Given the prevalence of NAD(P)(H)-dependent enzymes in nature, this paradigm suggests that many additional nickel pincer-dependent enzymes with diverse catalytic functions await discovery.

## Results

### Discovery of distinct NPMN-dependent enzymes

**Extended Data Fig. 1:**
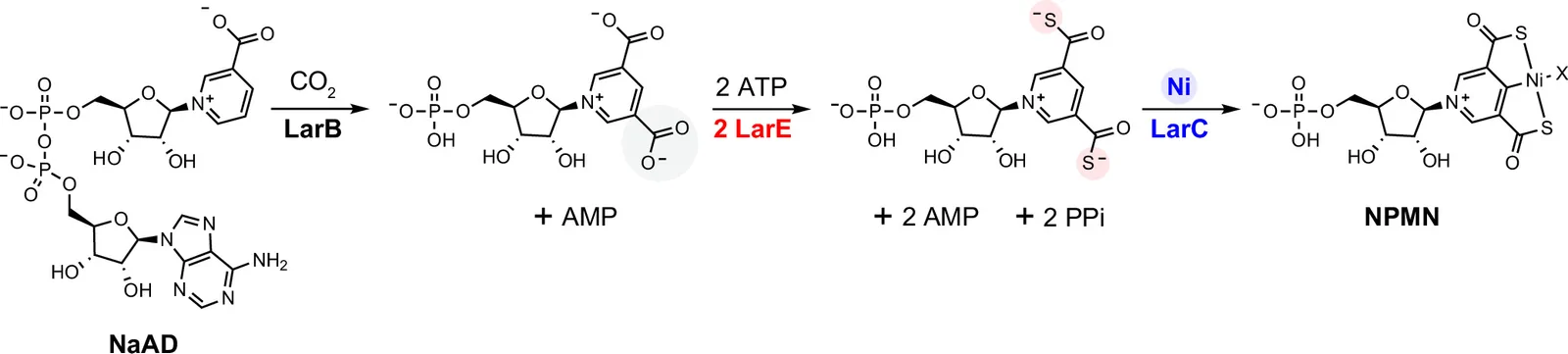
Biosynthetic pathway for NPMN. NPMN is synthesized in three enzymatic steps from nicotinic acid adenine dinucleotide (NaAD)^29^ to yield NPMN, which is then covalently tethered to LarA.

We developed a bioinformatic pipeline to discover NPMN-utilizing candidate enzymes with sequences and predicted structures that are completely different from those of LarA. To achieve this, we mined bacterial genomes for candidates that lie adjacent to genes that make the NPMN cofactor (Fig. 1c), taking advantage of the organization of bacterial genes into ‘clusters’ of enzymes that function together in a biochemical pathway. The nickel pincer mononucleotide cofactor is synthesized in three steps from nicotinic acid adenine dinucleotide (NaAD) by the enzymes LarB, LarE and LarC^29^ (Extended Data Fig. 1). LarB catalyses carboxylation of NaAD and hydrolysis of the phosphoanhydride bond, releasing adenosine monophosphate (AMP)^37^. Next, LarE catalyses sacrificial sulfur insertion, transferring sulfur from a cysteine side chain to yield a thiocarboxylate^38^. Finally, LarC generates a nickel–carbon bond, yielding NPMN^39,40^, which is then covalently tethered to LarA via a lysine residue. Of the biosynthetic enzymes, we hypothesized that LarC, which forms the unique carbon–nickel bond in NPMN^40^, would be the strongest predictor of NPMN synthesis by a host organism. We therefore used the Enzyme Function Initiative–Enzyme Similarity Tool (EFI–EST)^41^ to identify LarC homologues (InterPro family IPR002822) with BLAST (*E*-value of 10^−5^) and filtered the resulting list for clusters that contain LarB and LarE but lack LarA within 20 open reading frames of LarC^41^ (Supplementary Fig. 1). The recently reported NPAD-dependent ornithine cyclodeaminase was discovered using a similar bioinformatic approach but using LarE as a search anchor rather than LarC^22^.

**Fig. 2:**
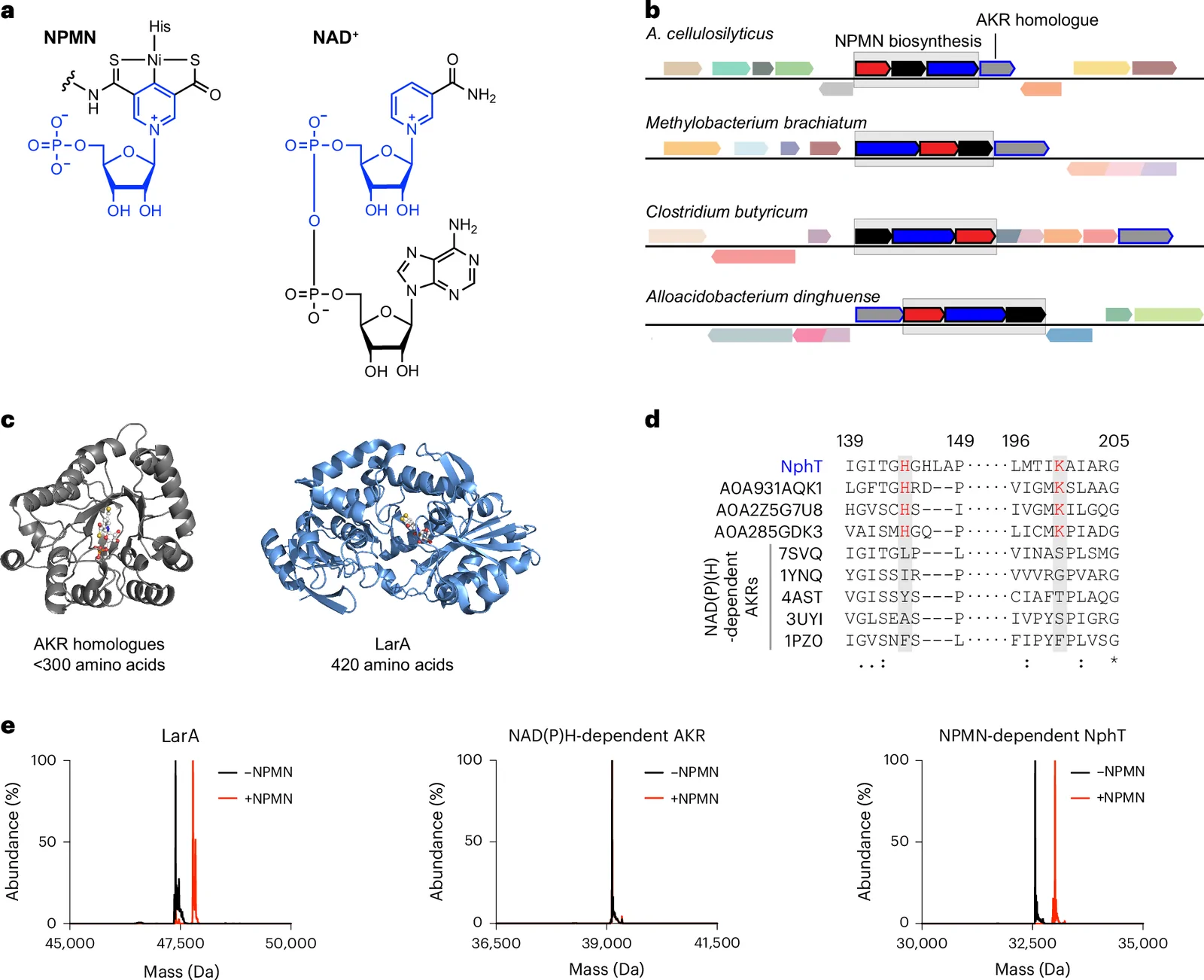
Nickel pincer-dependent hydride transferase (NphT) is an NPMN-utilizing enzyme. **a**, The NPMN cofactor is structurally related to NAD^+^. **b**, Genes encoding for homologues of AKRs co-occur with the biosynthesis genes. This AKR enzyme fold was not previously known to use NPMN. **c**, AKRs (grey) and LarA (blue) are not structurally related. AKRs have a TIM barrel fold, in contrast to LarA, which has a unique α/β fold. **d**, The AKR homologues adjacent to the NPMN biosynthetic cluster (NphT, blue; A0A931AQK1, 29.1% sequence identity to NphT; A0A2Z5G7U8, 23.3% ID; A0A285GDK3, 34.9% ID) possess conserved histidine and lysine residues that are absent in characterized AKRs that do not bind NPMN. PDB accession numbers are provided for the characterized AKRs. **e**, Intact protein MS analysis of LarA, AKR14A1 (a canonical NAD(P)H-dependent AKR) or our candidate (NphT) incubated with NPMN. Covalent binding of NPMN increases the protein mass by 450 Da. **LarA** − **NPMN** expected: 47,392.4 Da, observed: 47,392.3 Da. **LarA** + **NPMN** expected: 47,842.3 Da, observed: 47,842.5 Da. **Canonical AKR** − **NPMN** expected: 39,123.1 Da, observed: 39,124.0 Da. **Canonical AKR** + **NPMN** expected: 39,573.0 Da, observed: 39,124.0 Da. **NphT** − **NPMN** expected: 32,559.7 Da, observed: 32,560.7 Da. **NphT** + **NPMN** expected: 33,009.6 Da, observed: 33,010.4 Da. The results are representative of *n* = 3 experimental replicates. Source data

We next analysed the genomic context to identify protein families that co-occur with NPMN, focusing on proteins that lack homology to LarA. Interestingly, many enzymes that co-occur with LarB, LarC and LarE are annotated as NAD(P)(H)-dependent oxidoreductases. Given the structural similarity between NAD^+^ and NPMN (Fig. 2a), we hypothesized that these enzymes may be misannotated and may bind NPMN instead. We performed structural prediction of the enzymes with Chai-1^42^ followed by in silico NPMN and NAD(P)^+^ cofactor docking with DiffDock^43^ to identify enzymes that are predicted to bind NAD(P)^+^ poorly but bind NPMN well. As LarA contains a lysine that covalently tethers NPMN via a thioamide, as well as a histidine residue that coordinates nickel, we searched the active sites for appropriately positioned histidine and lysine residues to further support the nomination of these candidates as NPMN binders.

### NPMN-binding homologues of AKRs

Through this approach, we discovered a family of AKRs (PFAM00248) that co-occur with NPMN biosynthetic genes in 427 instances (Fig. 2b and Supplementary Fig. 2). AKRs are a widespread superfamily of NAD(P)(H)-dependent oxidoreductases that are found across all domains of life^36^. They catalyse the reduction of carbonyls to alcohols or the oxidation of alcohols to carbonyls (Supplementary Fig. 3). AKRs have an (α/β)_8_ triose-phosphate isomerase (TIM) barrel fold that is completely distinct from LarA, with three loops that define substrate specificity^44^. Furthermore, AKRs are over 100 amino acids smaller than LarA (~290 versus ~420 amino acids; Fig. 2c).

Canonical AKRs contain a tunnel that accommodates NAD(P)(H)^36^. By contrast, many of the AKRs that co-occur with the NPMN pathway lack space for the AMP moiety. Consistent with this observation, two characterized AKRs that are known to bind NAD(P)^+^ (UniProt Q46851 and Q9KE47) were predicted by DiffDock to do so (Supplementary Fig. 4). By contrast, docking of NAD(P)^+^ into LarA or our putative NPMN binder resulted in low DiffDock confidence scores with the AMP moiety of NAD(P)^+^ extending outside of the binding pocket. Encouragingly, these enzymes were predicted by DiffDock to accommodate NPMN (Supplementary Fig. 5).

We next wondered if the AKRs that co-occur with LarC contain the key histidine and lysine residues that are known to bind NPMN in LarA. Sequence alignments revealed a histidine/lysine motif among candidate AKRs associated with the Lar pathway that is absent in canonical NAD(P)(H)-dependent AKRs (Fig. 2d and Supplementary Fig. 6). Among these candidates, 34% contained both the histidine and lysine (Supplementary Fig. 7). Lysine was never observed without histidine, while 40% of sequences contained a histidine without lysine. These observations suggest that AKR homologues may bind NPMN covalently or non-covalently. It is also possible that AKR homologues with extended cofactor binding cavities might bind the recently discovered NPAD^22^. Thus, while canonical AKRs bind NAD(P)(H), we hypothesized that some homologues have evolved to bind NPMN instead and prioritized those with the histidine/lysine motif for further characterization.

### NphT binds the NPMN cofactor

To establish these AKR homologues as NPMN-dependent enzymes, we investigated NphT, a member of this enzyme family from *Alicyclobacillus cellulosilyticus*^45^, which grows at an optimal temperature of 55 °C and may contain thermostable nickel enzymes to facilitate characterization efforts. We first optimized conditions for the in vitro biosynthesis of NPMN using purified LarB, LarC and LarE. We incubated the pathway substrate, NaAD, with LarB S127A, a variant that was reported to favour the desired dicarboxylated product over unproductive hydrolysis products^37,46^. Next, we added LarE, which installs the sulfur atoms on the pincer ligand, and LarC, which inserts nickel (Supplementary Fig. 8). Following NPMN synthesis, we boiled and centrifuged the reactions to remove the biosynthetic enzymes, keeping the supernatant for subsequent loading of candidate enzymes with the cofactor.

We then tested covalent binding of NPMN to NphT by whole-protein mass spectrometry (MS). As controls, we also tested LarA, which binds NPMN, and L-glyceraldehyde 3-phosphate reductase (AKR14A1, UniProt Q46851)^47,48^, an AKR that binds NAD(P)(H) (Fig. 2e). Excitingly, when LarA or NphT were incubated with cofactor, we observed a mass increase of 450 Da, consistent with covalent cofactor binding. By contrast, incubation of L-glyceraldehyde 3-phosphate reductase with NPMN did not lead to a mass increase, consistent with its known dependence on NAD(P)(H). These results demonstrate that NphT binds NPMN.

### NphT catalyses intermolecular hydride transfer

**Fig. 3:**
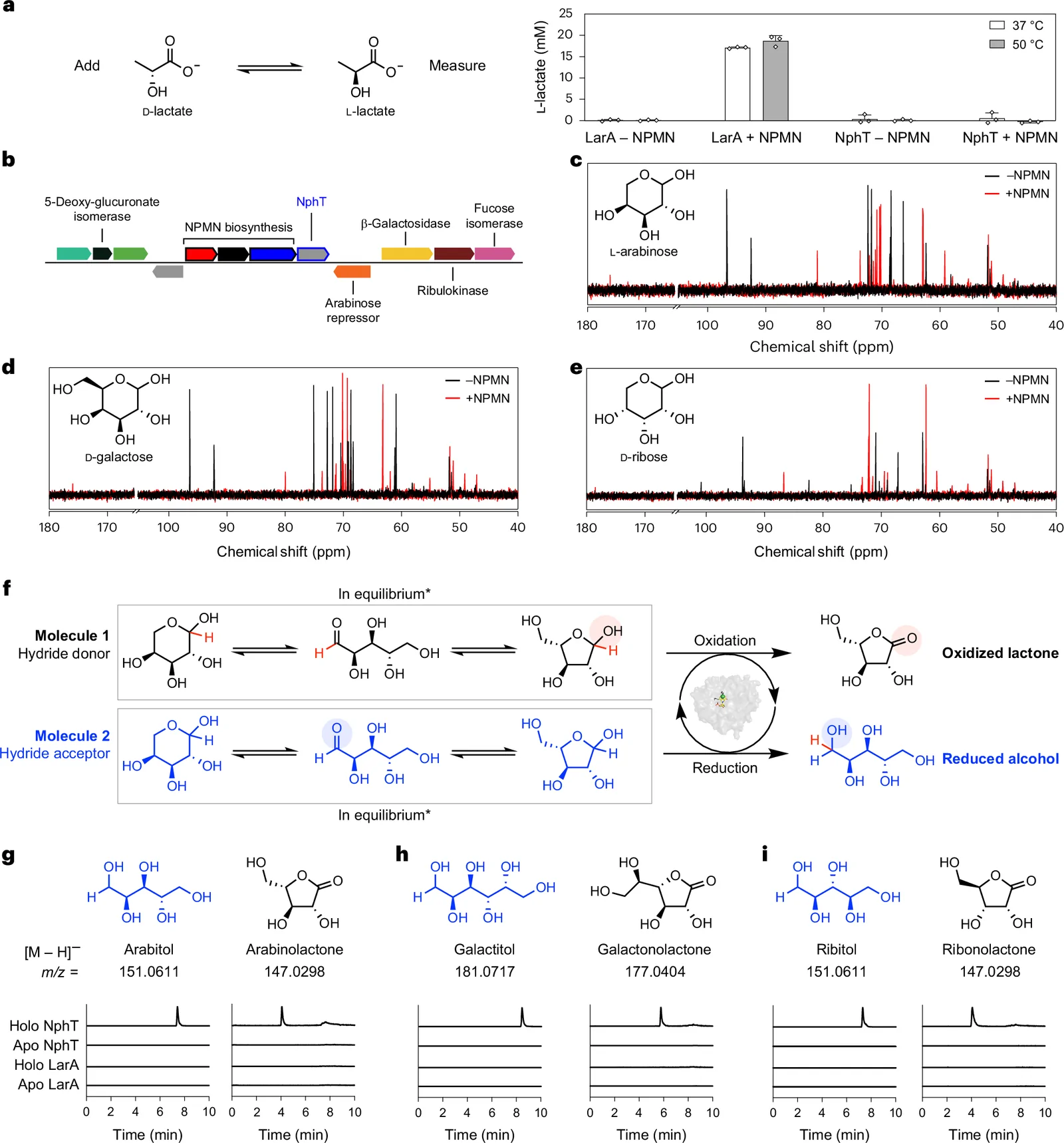
NphT catalyses intermolecular hydride transfer on carbohydrate substrates. **a**, NphT and LarA are assayed for lactate racemization activity at 50 °C and 37 °C for 2 h. Reactions (50 µl) contained 42.3 mM d-lactate and 0.4 µM enzyme in 100 mM MES buffer (pH 6). l-lactate resulting from racemization was quantified through enzymatic coupling to NADH production by l-lactate dehydrogenase^33^. Data are technical replicates (*n* = 3) with mean ± s.d. **b**, Genomic context suggests that NphT may act on sugar substrates. **c**–**e**, Incubation of NphT with l-arabinose (**c**), d-galactose (**d**) or d-ribose (**e**) leads to products that can be detected by ^13^C NMR. Multidimensional NMR analysis and product assignments are provided in Supplementary Information. **f**, Schematic of the observed intermolecular hydride transfer. *Although it is not known if the enzyme directly oxidizes the pyranose, furanose or linear aldose forms of the sugars (Supplementary Note 1), we observe the oxidized five-membered lactone product by NMR. **g**–**i**, LC–MS analysis of NphT and LarA activity on l-arabinose, d-galactose and d-ribose. Extracted ion chromatograms [M − H]^−^ of the sugar alcohol and lactone products generated by incubating NphT with arabinose (**g**, arabitol (calculated *m*/*z* 151.0611) and arabinolactone (calculated *m*/*z* 147.0298)), galactose (**h**, galactitol (calculated *m*/*z* 181.0717) and galactonolactone (calculated *m*/*z* 177.0404)) and ribose (**i**, ribitol (calculated *m*/*z* 151.0611) and ribonolactone (calculated *m*/*z* 147.0298)). Reactions (60 µl) contained 10 mM l-arabinose or d-galactose and 20 µM enzyme in 10 mM HEPES pH 7.5. Reactions proceeded for 20 h at 50 °C before quenching in 3 volumes of acetonitrile to precipitate protein before LC–MS analysis. For the ribose reactions, conditions were optimized to increase the yield of the lactone product before hydrolysis. Reactions (40 µl) contained 10 mM ribose and 5 µM enzyme in 25 mM HEPES pH 7.5 and proceeded for 2 h at 50 °C. Extracted ion chromatograms are representative of *n* = 3 experimental replicates. Source data

We next sought to determine the reaction catalysed by NphT. We first assayed NphT and LarA for lactate racemization activity at both 50 °C and 37 °C to account for the growth temperatures of their respective host organisms (Fig. 3a). While LarA catalysed racemization of d-lactate, NphT did not. Thus, we hypothesized that NphT catalyses a reaction distinct from lactate racemization.

To identify potential substrates for NphT, we examined its genomic context and found neighbouring genes associated with sugar metabolism, such as 5-deoxy-glucuronate isomerase (A0A917K6S5), β-galactosidase (A0A917K627), arabinose repressor (A0A917K5S6) and fucose isomerase (A0A917NHY4) (Fig. 3b). We therefore hypothesized that NphT might perform catalysis on a sugar substrate. To test this, we curated a panel of 28 mono- and disaccharide substrates including d-galactose, l-arabinose, d-ribose, d-glucose, l-rhamnose and α-lactose (Supplementary Fig. 9). NphT was loaded with NPMN, desalted to remove small molecules, incubated with each substrate and screened by NMR (Supplementary Fig. 10). While most of the tested sugars yielded no products, the incubation of d-galactose, l-arabinose and d-ribose with holo NphT resulted in the formation of new peaks and depletion of substrate peaks (Fig. 3c–e). By contrast, apo-enzyme negative controls lacking the cofactor did not yield products. Incubation of NphT with the substrates and various combinations of NAD^+^, NADP^+^, NADH and NADPH also yielded no products, suggesting that NphT modifies these substrates in an NPMN-dependent manner (Supplementary Fig. 11).

From our substrate panel (Supplementary Fig. 9), it was not clear which structural features lead to processing by NphT. NphT exhibits very low levels of activity on d-glucose and l-rhamnose compared with its relatively higher activity on d-galactose, l-arabinose and d-ribose (Supplementary Fig. 12). To test whether glucose dismutation is thermodynamically unfavourable, we incubated gluconolactone and sorbitol with NphT to probe the reverse reaction (Supplementary Fig. 13). Glucose formation was not detected under these conditions, indicating that the lack of glucose dismutation is due to poor substrate processing rather than an unfavourable reaction equilibrium.

We next assigned the structures of the products by NMR. The signals for the anomeric protons of arabinose, galactose and ribose were depleted after enzyme incubation, consistent with modification at C1. In comparison with synthetic standards, we determined that the products of incubation with l-arabinose are arabinolactone and arabitol (Supplementary Figs. 14–17). Similarly, we identified the products of incubation with d-galactose as galactonolactone and galactitol (Supplementary Figs. 18–21) and the products of d-ribose as ribonolactone and ribitol (Supplementary Figs. 22–25). NphT catalyses over 1,600 turnovers, with 15 μM enzyme fully consuming 50 mM ribose substrate (Supplementary Fig. 26).

**Extended Data Fig. 2:**
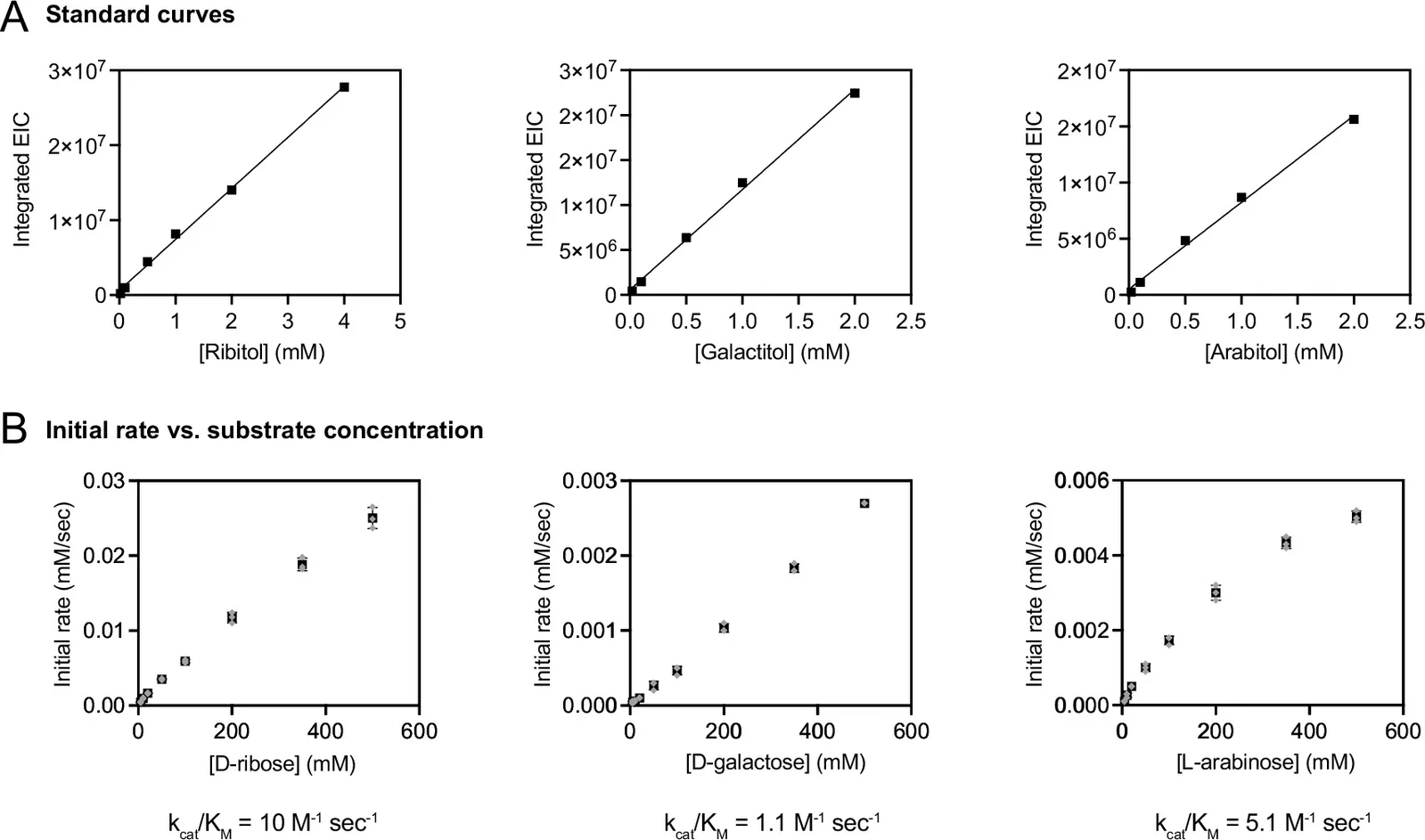
Kinetic analysis of NphT. (**a**) Standard curve for quantification of reduced alcohol products by LCMS. Data points are average of n = 2 sample injections. Line represents linear fit of data. (**b**) Initial rate vs. substrate concentration. Black squares are the average of n = 3 experimental replicates, error bars represent ±s.d., and grey diamonds are individual data points. In a Coy anaerobic chamber, NphT was loaded with NPMN cofactor as described in the Methods. Following loading, NphT was purified anaerobically on a size exclusion column. Substrate stocks of varying concentration were pre-heated to 50 °C for 15 min in PCR strips. Separately, enzyme and HEPES pH 7.5 were mixed and heated. At t = 0, substrate was added to the diluted enzyme using a multichannel pipette. Final reactions contained 5 μM enzyme, 40 mM HEPES pH 7.5, and substrate concentrations ranging from 5 mM to 500 mM. At t = 15, 60, 120, and 240 s, 10 μL samples of the reaction were quenched in 70 μL of 6:1 acetonitrile:water with 1% formic acid. Samples were freeze-thawed at -80 °C before adding 10 μL methanol and centrifuging at 20,000 *x g* for 7 min. Supernatant was transferred to LCMS vials for analysis on a HILIC column (Waters). The plotted initial rate is the product formed per second by 5 μM NphT. Note that the arabinose substrate co-elutes with the arabitol product, which may lead to ion suppression at the highest arabinose concentrations tested. In the absence of saturation, the specificity constant (k_cat_/K_M_) was calculated from the slope of the initial velocity plots, which provides a measure of catalytic efficiency. For ribose, k_cat_/K_M_ was determined to be 10 M^−1^s^−1^. For galactose, k_cat_/K_M_ was determined to be 1.1 M^−1^s^−1^. For arabinose, k_cat_/K_M_ was determined from the linear region (0-20 mM substrate) to be 5.1 M^−1^s^−1^. Source data

We also performed kinetic analysis of NphT on l-arabinose, d-galactose and d-ribose (Extended Data Fig. 2). As we did not observe saturation at 500 mM substrate concentrations, we calculated the specificity constants (*k*_cat_/*K*_M_) from the slope of the initial velocity plots. NphT was most active on d-ribose (*k*_cat_/*K*_M_ = 10 M^−1^ s^−1^), less active on l-arabinose (*k*_cat_/*K*_M_ = 5.1 M^−1^ s^−1^) and the least active on d-galactose (*k*_cat_/*K*_M_ = 1.1 M^−1^ s^−1^). These results suggest that these sugars are probably promiscuous substrates rather than primary biological substrates. While we cannot conclude the physiological substrate, our results demonstrate that NphT catalyses disproportionation (dismutation) of sugars into lactones and alcohols (Fig. 3f).

We then investigated the stoichiometry of the products to determine whether the oxidized and reduced products were formed in a 1:1 ratio, as expected for a redox-neutral disproportionation in which a hydride is transferred from one substrate to another. Although it is not known if NphT directly oxidizes the pyranose, furanose or linear aldose forms of the sugars (Supplementary Note 1), we observe oxidized five-membered lactones by NMR, which could arise from direct oxidation of the furanose. The lactone can then be hydrolysed to yield a ring-opened hydroxyacid, which we also observe^49,50^ (Supplementary Fig. 27). Quantitative ^13^C NMR shows a 1:1 ratio of oxidized (lactone plus hydroxyacid) and reduced (alcohol) products, supporting intermolecular hydride transfer (Supplementary Fig. 28). We confirmed the identity of these products by liquid chromatography (LC)–MS comparison to synthetic standards (Supplementary Fig. 29) and demonstrated that LarA does not catalyse these reactions (Fig. 3g–i and Supplementary Fig. 30). To our knowledge, this chemistry has not previously been observed in a nickel enzyme.

### Crystal structure of NPMN-bound NphT

**Fig. 4:**
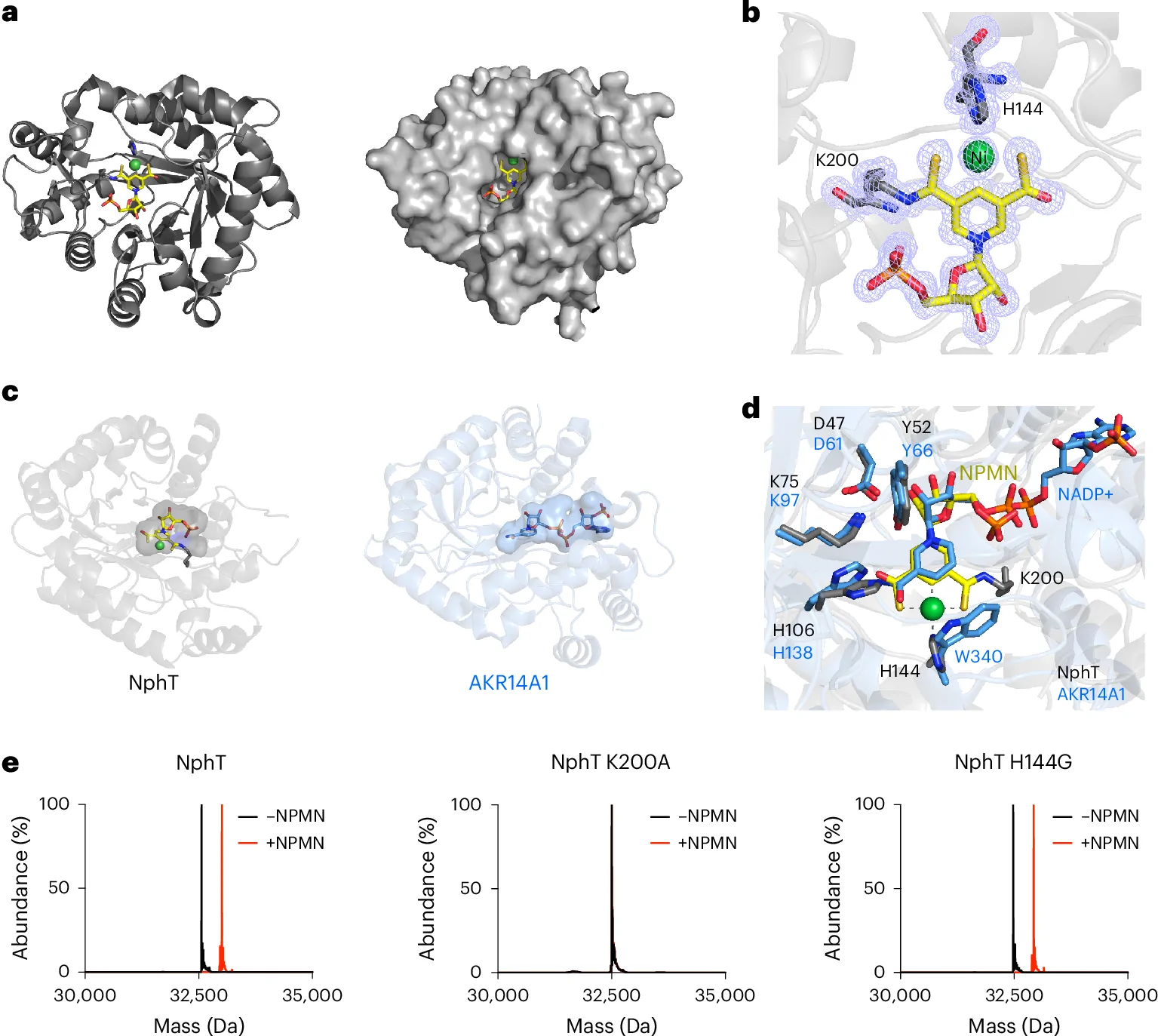
Crystal structure of NphT bound to NPMN identifies cofactor-binding residues. **a**, Overall fold of NphT structure solved to 1.3 Å resolution (PDB accession 9YEB). **b**, Composite omit electron density map (2*F*_o_ − *F*_c_, contoured at 1 σ) around NPMN (yellow), His144 and K200. **c**, Cofactor cavity in NphT accommodates NPMN. Larger cofactor cavity in AKR14A1 (PDB 4AUB) accommodates NADP^+^. **d**, Overlay of the active sites of NphT (grey) and AKR14A1 (blue). The NPMN cofactor in NphT is shown in yellow. **e**, Protein MS to analyse the mass of NphT mutants following incubation with NPMN (red) or with a control lacking full cofactor synthesis (black). **NphT** − **NPMN** expected: 32,559.7 Da, observed: 32,560.7 Da; **NphT** + **NPMN** expected: 33,009.6 Da, observed: 33,010.6 Da; **NphT K200A** − **NPMN** expected: 32,502.6 Da, observed: 32,503.8 Da. **NphT K200A** + **NPMN** expected: 32,952.5 Da, observed: 32,503.8 Da; **NphT H144G** − **NPMN** expected: 32,479.6 Da, observed: 32,480.7 Da; **NphT H144G** + **NPMN** expected: 32,929.5 Da, observed: 32,930.6 Da. The results are representative of *n* = 3 experimental replicates. Source data

To further investigate NphT and its unprecedented activity, we solved a crystal structure with NPMN bound in the active site to 1.3 Å resolution (Fig. 4a and Supplementary Table 2). In an anaerobic chamber, we synthesized NPMN in vitro and then loaded, purified and crystallized NphT. The resulting crystals were pale orange and diffracted to 1.3 Å resolution. We solved the crystal structure of NphT and found that it displays the TIM barrel fold that is characteristic of the AKR superfamily, with eight parallel strands in a barrel arrangement and helices on the outside^51^. NphT also contains three loops near the active site entrance, which may contribute to substrate selectivity as in canonical AKR enzymes^36^. Except for the key NPMN-binding lysine and histidine residues, the structure of NphT is completely different from that of LarA, which contains a unique α/β fold that is not a TIM barrel, demonstrating that distinct protein folds have evolved to accommodate NPMN.

Our data elucidate the structural basis for how NphT binds NPMN instead of the NAD(P)(H) cofactor used by its AKR relatives. The maps contained clear electron density for NPMN, which we modelled with an occupancy of one on the basis of 100% loading by LC–MS (Fig. 4b and Supplementary Fig. 31). Data collected at the nickel edge (1.48 Å) confirm the location of nickel (Supplementary Fig. 32). The polar functional groups of NPMN are coordinated by Y219, W218, D47, H106, T142 and the backbone amides of G248, A20 and A21, while I199 stabilizes the pyridine ring through hydrophobic interactions (Supplementary Fig. 33). These residues are highly conserved among NphT homologues (Supplementary Fig. 34). Although LarA and its homologues^35^ differ in fold from NphT, they also use polar residues to stabilize NPMN and provide a hydrophobic pocket for the pyridine ring.

NphT has a smaller cofactor cavity than the NAD(P)H-dependent homologue AKR14A1, which has an extended cavity to accommodate the AMP moiety of the larger cofactor (Fig. 4c). An overlay of NphT with AKR14A1 reveals residues in NphT that cap the NPMN-binding cavity and would clash with the larger NADP^+^ cofactor found in AKR14A1 (Fig. 4d and Supplementary Fig. 35). Our findings show that AKR enzyme family members can evolve to bind either NAD(P)(H) or NPMN, demonstrating evolutionary plasticity of cofactor binding. These results suggest that more nickel enzymes await discovery from diverse superfamilies of enzymes thought to rely solely on NAD(P)(H).

We next investigated the role of the histidine and lysine residues in cofactor binding. Consistent with the structure, mutation of K200 to alanine abolishes cofactor binding (Fig. 4e). Mutation of the nickel-coordinating H144 to glycine does not prevent NPMN loading but diminishes activity (see below). Beyond the differences in overall fold between NphT and LarA, these two enzymes differ in their placement of NPMN. In NphT, nickel is near the surface of the protein and solvent exposed, rather than buried as in LarA^34^. Size exclusion chromatography suggests that NphT is a monomer, so NPMN is not occluded by oligomerization of NphT (Supplementary Fig. 36). Taken together, our data establish NphT as a member of a unique structural family of nickel enzymes.

We next performed molecular docking to probe substrate selectivity. We docked linear and cyclic forms of d-galactose, l-arabinose, d-ribose, d-glucose and l-rhamnose into the NphT structure (Supplementary Fig. 37). Because the direct substrate (linear or cyclic) is unclear (Supplementary Note 1), and because all sugars were accommodated within the large, solvent-exposed active site, the docking studies were inconclusive in explaining the observed differences in substrate processing. One possibility is that these sugars differ in the equilibrium distribution of linear aldose, α/β-pyranose and α/β-furanose species, which could impact reaction rates depending on which is used by the enzyme^49^. Future structural studies of NphT bound to substrates may explain these differences.

### Mutagenesis of active site residues to probe mechanism

**Fig. 5:**
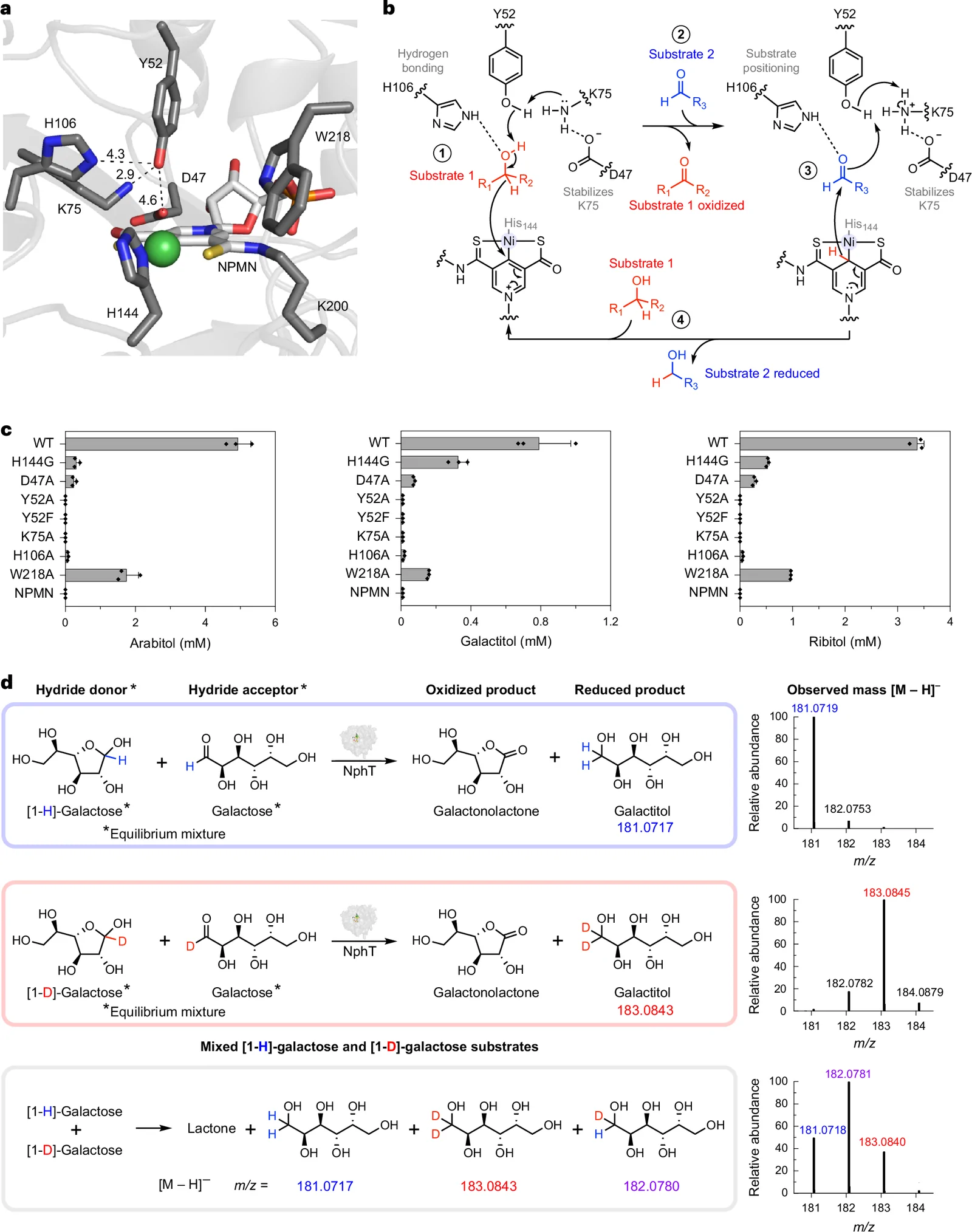
Mechanistic investigation of NphT. **a**, The active site of NphT contains residues that correspond to the proposed catalytic tetrad of canonical NAD(P)(H)-dependent AKRs. **b**, Proposed role of catalytic tetrad by analogy to AKRs with the proton relay network depicted. This schematic depicts hydride transfer to C4 of NPMN by analogy to the proposed mechanism of racemization by LarA^30^. **c**, Mutagenesis of key active site residues impacts catalytic activity on arabinose, galactose and ribose. Reactions (40 µl) contained 10 mM substrate and 5 µM enzyme in 25 mM HEPES (pH 7.5) at 50 °C. The resulting sugar alcohol product was quantified by LC–MS. NPMN without enzyme was included as a negative control. Data are technical replicates (*n* = 3) with mean ± s.d. **d**, Deuterated galactose substrate to identify the hydride source. Reactions (40 µl) contained 8 µM enzyme, 20 mM HEPES (pH 7.5), and 10 mM d-[1-^**1**^**H**]-galactose, 10 mM d-[1-^**2**^**H**]-galactose or both. Following incubation at 50 °C for 5 h, products were analysed by LC–MS in negative ionization mode [M − H]^−^. *Although it is not known whether NphT acts directly on the pyranose, furanose or linear aldose (Supplementary Note 1), galactose is depicted as a furanose here, as we observe the five-membered lactone product by NMR. This depiction facilitates identification of the chemical transformation. The results are representative of *n* = 3 experimental replicates. Source data

In addition to the histidine and lysine residues that bind NPMN, the active site contains the conserved catalytic tetrad (Y52, H106, K75 and D47) found in canonical AKRs (Fig. 5a). In canonical AKRs, histidine, lysine and aspartate tune the pKa of tyrosine, which acts as a catalytic base for alcohol deprotonation^52^, promoting hydride transfer to NAD(P)^+^ and formation of the oxidized product. The reverse reaction occurs via hydride transfer from NAD(P)H to a carbonyl, yielding the alcohol. Histidine is proposed to contribute to substrate binding^52^ and proton transfer^53^, while aspartate forms a salt bridge that positions lysine to tune the pKa of tyrosine, the catalytic base^52^.

**Extended Data Fig. 3:**
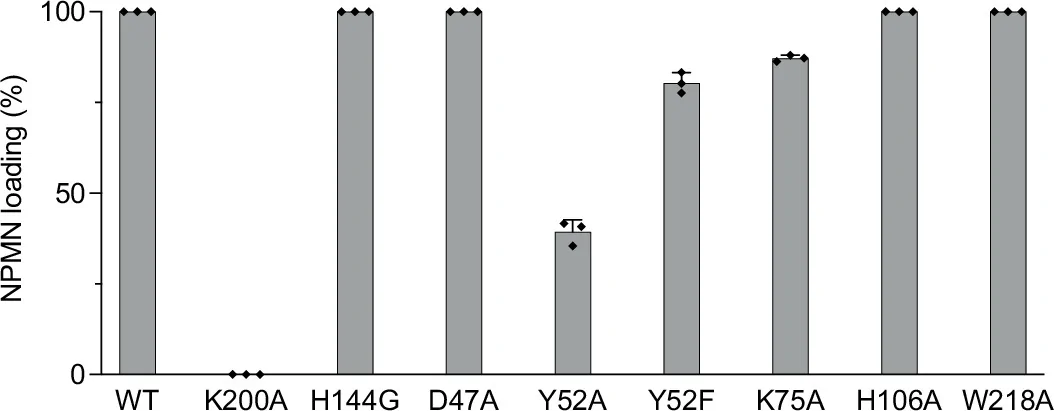
Summary of cofactor loading of NphT mutants. *In vitro* cofactor synthesis using LarB, LarE, and LarC was performed as described in the Methods. The NPMN cofactor was incubated with 40 µM enzyme for 20 h at room temperature. 20 µl of each of the loaded enzyme samples were desalted using a ZebaSpin desalting column (7 kDa MWCO). 20 µl of the desalted protein was mixed with 20 µl of water and 20 µl of acetonitrile (mass spectrometry grade) and analyzed by LCMS as described in the Methods. The percentage of loaded protein was calculated from the deconvoluted spectra (Agilent BioConfirm). The data are the average of n = 3 experimental replicates ± s.d. Source data

In NphT, Y52 is positioned to serve as a general acid/base, while H106, D47 and K75 may serve mechanistic roles by analogy to canonical AKRs (Fig. 5b). First, we investigated how mutagenesis of these residues impacts NPMN binding. We generated D47A, Y52F, Y52A, K75A and H106A point mutants of NphT. We also tested W218A, located on the substrate binding loop. All the mutants retained cofactor binding (Extended Data Fig. 3 and Supplementary Fig. 38), although loading was diminished in K75A (87.2 ± 0.9%), Y52A (39.3 ± 3.4%) and Y52F (80.4 ± 2.9%).

**Extended Data Fig. 4:**
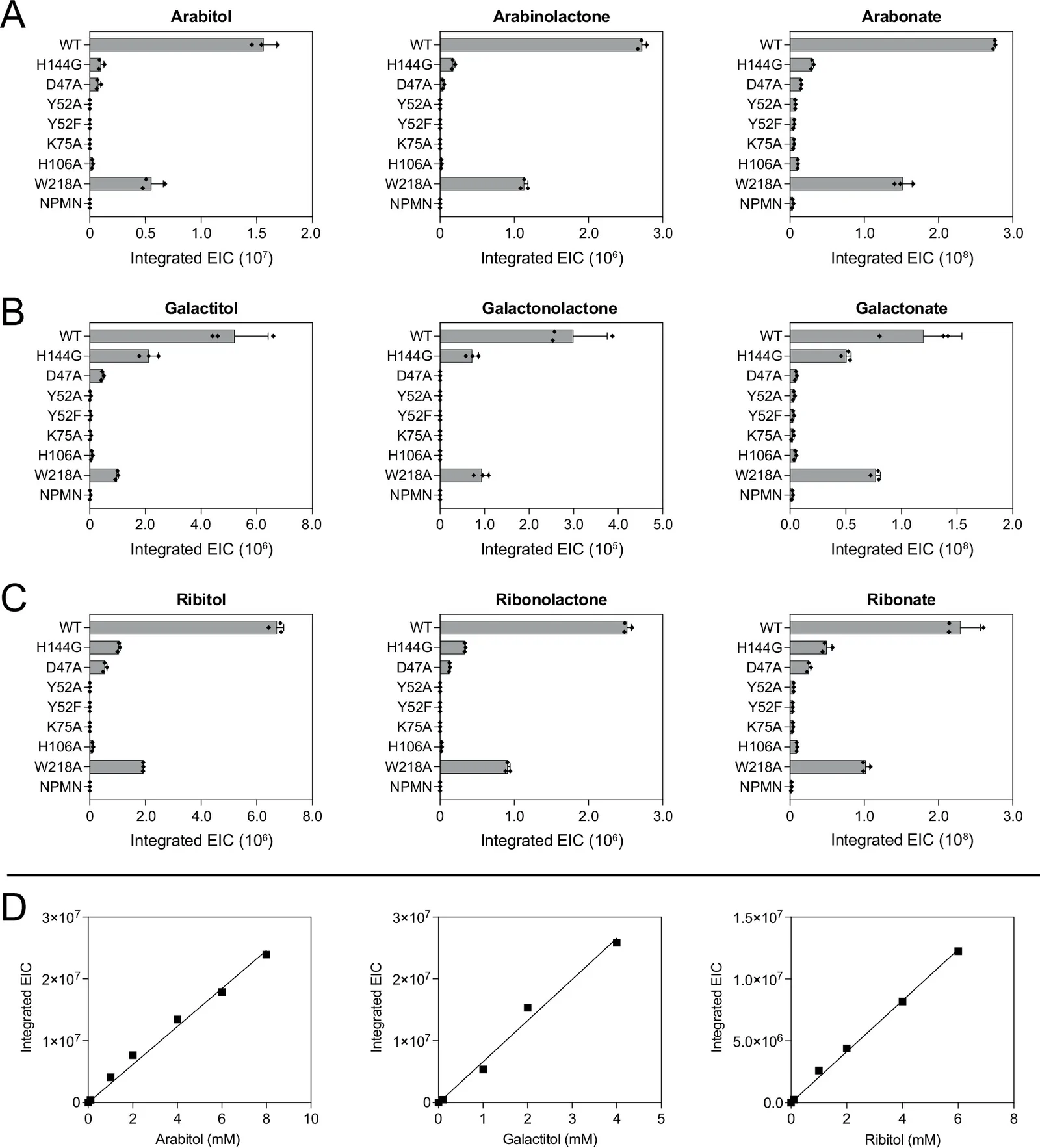
Activity of NphT variants. Mutagenesis of key active site residues impacts catalytic activity on (**a**) l-arabinose, (**b**) d-galactose, and (**c**) d-ribose. Reactions (40 µl) contained 10 mM substrate and 5 µM enzyme in 25 mM HEPES pH 7.5 at 50 °C. NPMN without enzyme was included as a negative control. Reactions were allowed to proceed for 5 h (l-arabinose), 4 h (d-galactose), or 2 h (d-ribose) at 50 °C. These times were selected to optimize the detection of the lactone species before hydrolysis to the acid. 10 µL of the reaction was removed from the chamber and quenched in 70 µL of 6:1 acetonitrile:water and freeze-thawed on dry ice for a total of 3 times. After the final thaw, 10 µl of methanol was added and the sample was briefly vortexed and then centrifuged for 10 min at 13000 *x g* before transfer of the supernatant into an HPLC vial. The data in (**a**) through (**c**) are technical replicates (n = 3) with mean ±s.d. (**d**) Standard curve of arabitol, galactitol, and ribitol used to quantify the sugar alcohol products, which are presented in Fig. 5c. The standard curve was made once (n = 1). Source data

Next, we tested the activity of the NphT variants (Fig. 5c and Extended Data Fig. 4). Mutagenesis of the proposed proton relay network impaired activity: Y52A, Y52F and K75A variants were inactive, indicating that these residues are essential for catalysis. Relative to wild-type NphT, H106A and D47A yielded <3% and <10% of the sugar alcohol products, respectively, while W218A yielded 19–35% of the products depending on the sugar substrate. The H144G variant still yielded products but at diminished levels. Together, these results suggest that NphT uses the nickel pincer cofactor to catalyse a redox-neutral hydride transfer that is mechanistically related to the chemistry performed by AKRs.

NphT’s intermolecular hydride transfer activity is distinct from the chemistry reported in LarA, which has two catalytic histidine residues that serve as general acids and bases to facilitate lactate racemization. Notably, some LarA homologues lack one of these histidine residues and therefore may perform chemistry other than racemization^35^. Although these LarA homologues adopt a different fold from NphT, they have a tyrosine residue positioned similarly to Y52 (Supplementary Fig. 39). Several histidines lie within ≤3.7 Å of the tyrosine, including one that is stabilized by an aspartate, analogous to the D47-K75 interaction in NphT. Although their functions remain unknown, it is possible that these LarA homologues could also catalyse hydride transfer.

### Disproportionation of isotopically labelled substrates

Intermolecular hydride transfer has not been reported in the LarA family of NPMN-dependent enzymes and therefore expands the chemistry of NPMN. To further investigate this activity in NphT, we performed deuterium tracing. We incubated NphT with either protiated or deuterated (d-[1-^2^H])-galactose and analysed the resulting products by LC–MS (Fig. 5d). While d-[1-^**1**^**H**]-galactose yields a protiated galactitol product with a mass of 181.0719, d-[1-^**2**^**H**]-galactose yields a product with a mass of 183.0845 that contains two deuterium atoms. Thus, the source of the hydride or deuteride is the C1 position of galactose and the destination is the galactose aldehyde substrate. Finally, mixing d-[1-^**1**^**H**]-galactose and d-[1-^**2**^**H**]-galactose substrates yields a product distribution centred around 182.0781, which contains one deuterium in the alcohol product. These results support an intermolecular hydride transfer and demonstrate that the NPMN cofactor can catalyse disproportionation of sugar substrates, revealing distinct chemistry for nickel enzymes.

### Transhydrogenation activity

We next tested whether NphT could catalyse transhydrogenation by shuttling hydride between different substrates. Incubation of NphT with a mixture of protiated arabinose and deuterated (d-[1-^2^H])-galactose yields galactitol with a mass of 182.0780, consistent with hydride transfer from arabinose to galactose (Supplementary Fig. 40). We also performed a time-course experiment in which NphT was incubated with equal concentrations of d-galactose, l-arabinose and d-ribose (Supplementary Fig. 41). In contrast to the experiment in which sugars were tested individually, the ratio of oxidized and reduced products of a particular sugar is not 1:1 throughout the experiment, suggesting that one sugar can serve as the hydride donor for another.

We next took advantage of the transhydrogenation activity to probe whether NphT might act on linear or cyclic substrates, which we could not conclude through docking (Supplementary Fig. 37). Incubation of butanal, an aldehyde substrate that cannot cyclize, with ribose and NphT yielded butanol, consistent with hydride transfer from ribose to butanal (Supplementary Fig. 42). We also detected low levels of butanal disproportionation when NphT was incubated with butanal alone. By contrast, the cyclic substrate inositol was not modified by NphT. However, this may reflect poor substrate compatibility rather than an inability to process cyclic substrates (Supplementary Fig. 43). While future efforts will be required to identify which sugar tautomers NphT acts on, our results demonstrate that NphT can reduce linear aldehydes and exhibits promiscuous transhydrogenase activity.

### NPMN-dependent AKRs are widespread in nature

**Fig. 6:**
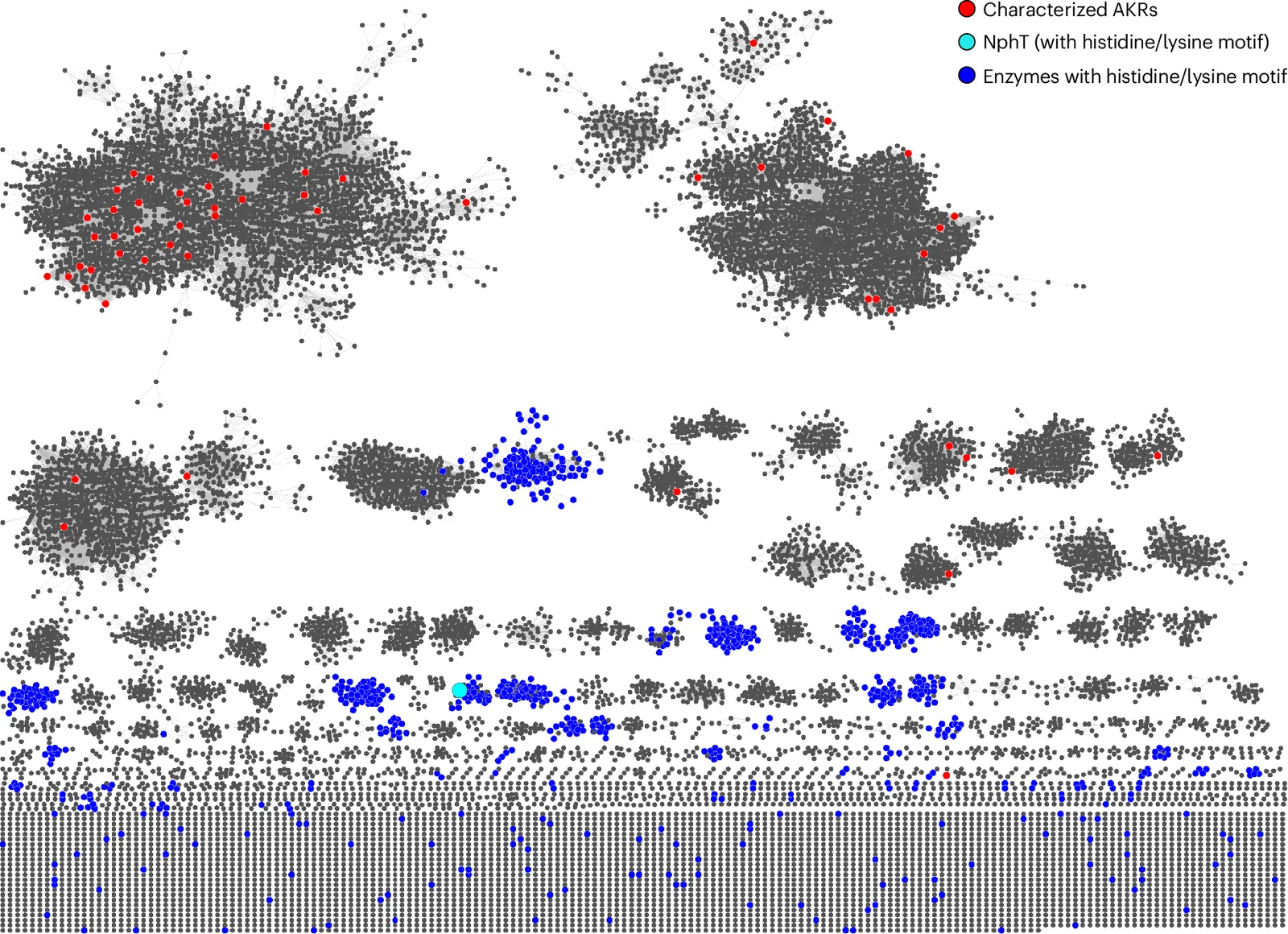
Unexplored putative nickel pincer enzymes within the AKR superfamily. Each dot represents a distinct enzyme, and the lines connecting them indicate sequence similarity above a given threshold. This SSN was generated using EFI–EST option D, containing UniProt accession numbers of NphT and characterized AKRs from the literature. The query was modified to exclude fragments and include Protein Family Addition Options for AKR superfamily (PF00248) as a query for BLAST (*E*-value of 10^−5^). UniRef50 was used to condense from 369,773 to 30,309 sequences. After initial processing, sequences below 150 amino acids in length were excluded from the SSN, resulting in 19,356 sequences. Using Cytoscape v3.10.1, the network was processed by removing edges on the basis of an alignment score value up to 67 and visualized using the yFiles Organic Layout. A multiple sequence alignment was generated using the MAFFT DPPartTree model in FASTA format, then parsed by an R script to extract sequences containing the histidine/lysine motif characteristic of NphT (Supplementary Code 3). Canonical AKRs characterized in literature are shown in red, our discovered NphT is shown in cyan and unexplored, putative nickel pincer-dependent NphT homologues containing the histidine/lysine motif are shown in blue. Source data

AKRs are a broad class of enzymes with diverse substrates found across all domains of life^36^. If additional AKRs use NPMN instead of NAD(P)(H), this would greatly increase the number of known nickel enzymes that serve as starting points for discovering uncharted reactions that use NPMN. To probe this possibility, we aligned sequences of NphT homologues and found that 798 of them contain the histidine/lysine motif indicative of nickel-pincer binding (Source Data Fig. 6 and Supplementary Fig. 34). These homologues may bind the cofactor covalently, such as LarA and NphT, or non-covalently, such as some LarA homologues^28^ and the NPAD-dependent ornithine cyclodeaminase^22^. It is also possible that some AKR homologues could bind NPAD rather than NPMN. Notably, these putative nickel pincer-dependent AKR homologues do not cluster with any previously characterized AKRs and are broadly distributed across the sequence similarity network (SSN), suggesting that they may act on diverse substrates (Fig. 6). Our bioinformatic results set the stage for the discovery of nickel-dependent members of the AKR superfamily.

## Discussion

Since the discovery of LarA in 2015, no other protein fold has been shown to covalently tether NPMN for catalysis. Although LarA homologues have been identified, reactions catalysed by the LarA family are limited to racemization and epimerization of hydroxyacids. In this work, we demonstrate that members of the diverse, NAD(P)(H)-dependent AKR enzyme superfamily, which has a completely different fold than LarA, have evolved to use the nickel pincer mononucleotide instead. We solved a 1.3-Å resolution crystal structure, providing insights into the mechanism of intermolecular hydride shuttling on organic substrates—chemistry that is unprecedented for nickel enzymes. Interestingly, Raney nickel catalyses sugar disproportionation, highlighting a convergence between biological and synthetic catalysis^54^.

NphT demonstrates that the AKR enzyme superfamily exhibits cofactor plasticity: while all previously characterized AKR homologues are NAD(P)(H) dependent, NphT exhibits covalent utilization of NPMN. By contrast, no members of the LarA family have been shown to use NAD(P)(H) for catalysis. In light of this discovery, it is exciting to consider whether other superfamilies of NAD(P)(H)-dependent enzymes, which catalyse over 2,000 types of redox reactions^55^, may harbour unexplored NPMN-dependent enzymes. The recent discovery of a NPAD-dependent ornithine cyclodeaminase demonstrates diversity in cofactor structure^22^, further suggesting that even more exciting discoveries lie ahead.

Our results have broad implications for the discovery and engineering of nickel-dependent enzymes. First, we demonstrate a pipeline to discover NPMN-dependent enzymes in a manner that relies on adjacency to LarB, LarC and LarE homologues, thereby escaping the narrow sequence space of LarA to enable the identification of NPMN-dependent enzymes with diverse folds and functions. Our bioinformatic pipeline identified several putative NPMN-dependent enzymes spread throughout the AKR superfamily, which has a broad substrate scope and suggests that nickel-dependent enzymes could act on diverse substrates. Second, we developed an experimental pipeline to achieve cofactor synthesis and the stoichiometric in vitro loading of an NPMN-dependent enzyme, which enabled our high-resolution crystal structure and will facilitate future characterization of distinct NPMN enzymes. Third, NphT has properties that make it a promising starting point for protein engineering: NphT is thermostable to 55 °C, catalyses over 1,600 turnovers and has a solvent-accessible active site. While the physiological substrate of NphT remains to be determined, NphT acts on sugars, expanding the substrate scope of nickel biocatalysis beyond the small, gaseous substrates of many nickel enzymes towards synthetically useful building blocks.

Our work expands the limited landscape of nickel biochemistry by identifying a unique family of nickel enzymes, demonstrates that NAD(P)(H)-dependent enzyme families can evolve to house the nickel pincer mononucleotide cofactor and sets the stage for furthering our understanding of the role of nickel in nature and developing biocatalysts that harness nickel for different chemistries.

## Methods

### General

Please see Supplementary Methods for commercial sources of materials and protein purification methods, as well as more detailed experimental methods.

### Bioinformatic identification of nickel pincer enzymes

To identify potential NPMN-utilizing candidate enzymes, the InterPro family of LarC homologues (IPR002822) was used as a query in the EFI–EST web tool^41^ using option B for BLAST (*E*-value of 10^−5^), yielding 11,067 hits. The EFI-GNT web tool^41^ was then used to identify genes adjacent to LarC. An R script was used to parse the list to include genomes containing LarB and LarE while removing genomes containing LarA within 20 open reading frames of LarC (the maximum allowable value in the EFI-GNT web tool), resulting in 2,108 sequences (Supplementary Code 1). The remaining LarC sequences were input into the EFI–EST web tool using option D to generate a sequence similarity network (SSN) from UniProt accession IDs. An alignment score threshold of 10^−18^ was used after initial processing. Using Cytoscape v3.10.1, the network was processed to estimate isofunctional clusters by removing edges on the basis of an alignment score value up to 131. The EFI-GNT web tool was used to identify adjacent genes as potential NPMN-utilizing candidates. The output.sqlite file generated from the EFI-GNT web tool above was read into an R script to profile PFAM co-occurrences with the NPMN biosynthesis genes (Supplementary Code 2).

### In silico docking of ligands with LarA and AKR homologues

The Chai-1-predicted structure of NphT was used as an input for Foldseek^56^ to search structures from the Protein Data Bank (PDB) that were most similar. DiffDock was used to dock the ideal SDF files of cofactors from the PDB into AKR homologues.

### Sequence alignment of AKR homologues

The five closest characterized structural homologues to NphT in the PDB were extracted from Foldseek^56^. These homologues, and NPMN biosynthesis-adjacent AKR homologues with lower sequence identify to NphT, were aligned using Clustal Omega.

### Generation of PF00248 SSN

To identify the distribution of putative nickel pincer-dependent AKRs containing the histidine/lysine motif shown in NphT, an SSN was generated using EFI–EST^41^. Option D was used to process UniProt accession numbers of characterized AKRs^36^ and NphT (Source Data Fig. 6). Cytoscape v3.10.1 was used to visualize and process the network by removing edges on the basis of an alignment score value up to 67. An alignment of the sequences was generated using the MAFFT DPPartTree and parsed by an R script to extract the 798 sequences containing the histidine/lysine motif characteristic of NphT (Source Data Fig. 6).

### Bacterial strains

Bacterial strains, plasmids and oligonucleotide sequences are presented in Supplementary Table 1. *Escherichia coli* Turbo cells (New England Biolabs) were used for plasmid construction. BL21(DE3) was used for heterologous protein production.

### Construction of plasmids

Gibson assembly^57^ was used to carry out plasmid construction using *E. coli* Turbo cells (New England Biolabs) as the cloning host. PCR amplifications were carried out with Phusion polymerase (New England Biolabs) using the oligonucleotides presented in Supplementary Table 1. Following plasmid construction, all cloned inserts were sequenced at Plasmidsaurus (South San Francisco, CA).

### Expression of His_10_-tagged and Strep-tagged proteins

*E. coli* BL21(DE3) was transformed with the appropriate protein expression plasmid. In the morning, colonies were picked into a 2-ml starter culture of terrific broth (TB), grown to mid-log, diluted to 6 ml and grown to mid-log again. This culture was used to inoculate TB (0.5 l) containing the appropriate antibiotics (100 μg ml^−1^ carbenicillin) in a 2.8-l baffled shake flask. The cultures were grown at 37 °C with shaking at 200 rpm to an optical density at 600 nm (OD_600_) of 0.6–0.8 at which point cultures were cooled on ice for 20 min, followed by induction of protein expression with isopropyl β-D-1-thiogalactopyranoside (IPTG) (0.25 mM) and overnight growth at 16 °C. In the case of LarC, nickel chloride (1 mM final concentration) was added to the media immediately before induction with IPTG. Cell pellets were harvested by centrifugation at 8,000*g* for 8 min at 4 °C and stored at −80 °C until purification (described in Supplementary Methods).

### In vitro cofactor synthesis for crystallography

All steps were performed in anaerobic chamber (Coy). Reactions (600 μl) contained NaAD (0.4 mM), sodium bicarbonate (50 mM) and magnesium chloride (0.4 mM) in 100 mM Tris buffer (pH 7.5). Reactions were initiated by the addition of purified LarB S127A (12 µM final concentration) and allowed to proceed for 20 h at room temperature to yield pyridinium-3,5-biscarboxylic acid mononucleotide (P2CMN). The samples were then centrifuged at 15,000*g* for 5 min to remove any precipitated LarB. To generate NPMN, reactions (725 μl) contained 196.5 μl of the LarB reaction from step 1, magnesium chloride (2.5 mM), ATP (2.5 mM), βME (5 mM), CTP (0.25 mM), manganese chloride (0.4 mM), nickel chloride (75 μM), LarC (80 μM) and LarE (130 μM). The reactions proceeded for 3.5 h at room temperature before boiling at 90 °C for 10 min to remove LarB, LarC and LarE. The sample was vortexed for 2 s, and the precipitated LarB, LarC and LarE were removed by centrifugation at 13,000*g* for 10 min. The supernatant (650 μl) was transferred to a fresh tube and used as the NPMN source for loading NphT for crystallography as described below.

### Cofactor loading for crystallography

In an anaerobic chamber (Coy), the NPMN cofactor was incubated with 40 μM NphT in a final volume of 685 μl for 18 h. The following day, the sample was centrifuged at 13,000*g* for 5 min to remove precipitate. The remaining soluble holo protein was concentrated with a Pall Nanosep 10k centrifugal filter to a volume of 400 μl and loaded onto a Superdex 75 Increase 10/300 GL column (Cytiva) equilibrated with Buffer C (20 mM HEPES, 100 mM NaCl) and eluted at a flow rate of 0.4 ml min^−1^. Fractions containing NphT were pale orange. NphT samples were combined and concentrated to 10 mg ml^−1^ using a 0.5-ml Amicon Ultra Centrifugal Filter, 10 kDa MWCO (Millipore). Glycerol was added to 3% (v/v), and the protein was immediately crystallized as described below.

### Crystallization and structure determination

Apo crystals of NphT were first obtained by the hanging drop vapour diffusion method by combining equal volumes of apo protein solution (4 mg ml^−1^ NphT) and reservoir solution (sodium acetate pH 4.6 (100 mM), ammonium chloride (200 mM), 22% (w/v) PEG 3350). Thin plates and rectangular prisms grew within 5 days. Crystals were transferred to an Eppendorf tube containing 250 μl reservoir solution and vortexed for 30 s with 10 × 1 mM diameter zirconia/silica beads to produce a microseed solution. The seed solution was divided into 50-μl aliquots and stored at −80 °C for future use.

The microseeds described above were used to seed the growth of NPMN-loaded NphT crystals by microseeding equal volumes of protein solution (4 mg ml^−1^ NphT in Buffer C) and reservoir solution (sodium acetate pH 4.5 (100 mM), ammonium chloride (200 mM), 25.5% (w/v) PEG 3350). Needles, rectangular rods and plates grew within 2 days and were looped and flash-frozen in liquid nitrogen on day 8. Data were collected at Beamline 8.3.1 at the Advanced Light Source (Lawrence Berkeley National Laboratory) at wavelengths of 1.11 Å and 1.48 Å.

Data were processed with XDS^58^ and scaled and merged with Aimless within the CCP4 suite^59^. The data were phased with molecular replacement in the Phaser module of Phenix^60^ using the AlphaFold3^61^-predicted structure of NphT as a search model. The structure was refined to 1.3 Å resolution iteratively using Coot^62^ for model building, followed by Phenix for refinement. Waters were added manually using Coot after the protein backbone and sidechains were built. Restraints for the NPMN ligand (PDB 4EY) were generated using the Elbow programme within Phenix. NPMN was added to the model using Coot, and the structure was further refined using Phenix. The occupancy of NPMN was set to 1 on the basis of LC–MS analysis of the holoenzyme preparation, and individual B-factors were refined (Supplementary Fig. 31).

Omit maps for NPMN were generated using the Phenix Composite Omit Map function. Phenix was then used to generate a map around NPMN, His144 and Lys200. Anomalous maps were generated using the dataset collected at 1.48 Å. Overall structural alignments were performed using the Chimera software (UCSF v1.7.1)^63^. The structures were analysed in Pymol^64^.

### Preparing samples for polar metabolite analysis

Reactions were initiated by the addition of purified enzyme and allowed to proceed at the indicated times and temperatures. Samples were then quenched in 3 volumes of acetonitrile, vortexed and treated with three freeze–thaw cycles (dry ice, then room temperature) to precipitate protein. Next, 0.5 volumes of methanol were added, and the samples were vortexed and centrifuged at 15,000*g* for 15 min to remove precipitated protein. Samples were then analysed by LC–MS on an Agilent 1290 ultra performance LC (UPLC)–6546 quadrupole time-of-flight (QTOF) using the protocol for polar metabolite analysis below.

### General procedure for high-resolution HPLC–MS analysis of polar metabolites with HILIC

Samples containing polar compounds were analysed using an Agilent 1290 UPLC–6546 QTOF in negative ionization mode. Chromatography was performed using an Atlantis Premier BEH Z-HILIC column or an Atlantis Premier BEH HILIC Amide column (1.7 μm, 2.1 × 150 mm; Waters) using buffer A (100% water, 15 mM ammonium bicarbonate, pH 9) and buffer B (90% acetonitrile, 10% water, 15 mM ammonium bicarbonate, pH 9) with a 25-min method, followed by a 5-min postrun equilibration (0.2 ml min^−1^). The gradient profile can be found in Supplementary Methods.

### Preparing samples for protein MS

Protein samples were desalted using Zeba Spin Desalting Columns. In brief, the 0.05% sodium azide storage solution was removed by centrifugation at 1,500*g* for 1 min, followed by equilibration with 3 × 300 µl of milliQ water. The column was then placed in a clean collection tube, and 20–30 µl of the protein sample (20–30 µM) was applied to the resin bed followed by 15 µl water. The sample was centrifuged at 1,500*g* for 2 min. The flowthrough was applied to the bed of a second equilibrated column and stacked with 15 µl water. The sample was again eluted by centrifugation at 1,500*g* for 2 min. The sample was combined 1:1 with LC–MS-grade 100% acetonitrile, centrifuged at 10,000*g* for 10 min to remove precipitates and transferred into a high-performance liquid chromatography (HPLC) vial. Samples were then analysed by LC–MS on an Agilent 1290 UPLC–6546 QTOF using the protocol for protein MS analysis below.

### General procedure for high-resolution HPLC–MS analysis of protein samples

Samples containing protein were analysed using an Agilent 1290 UPLC equipped with a PLRP-S column (1,000 Å, 5 μm, 2.1 × 50 mm; Agilent) using buffer A (100% water containing 0.1% formic acid) and buffer B (100% acetonitrile). The method was 9.5 min long, followed by a 2-min postrun equilibration (0.350 ml min^−1^). The gradient profile can be found in Supplementary Methods. Mass spectra were acquired in positive ionization mode using a 6546 QTOF (Agilent) and deconvoluted using Bioconfirm (v12.1, Agilent) via the Maximum Entropy algorithm.

### NMR data collection

The initial sugar screening reaction spectra (^1^H and ^13^C) were recorded on a Bruker Avance III HD 500 MHz spectrometer using a DCH LHe cryoprobe. Final experiment and standard spectra (^1^H, ^13^C, q^13^C, HH-COSY, HC-HSQC, HC-HMBC) were acquired on a Bruker Avance III HD 600 MHz spectrometer equipped with a TCI-F cryoprobe. Both instruments were controlled using Topspin (version 3.5.7) software. The gluconolactone and sorbitol kinetics reaction spectra (^1^H) were recorded on a Bruker Avance NEO 500 MHz spectrometer with a Prodigy BBO cryoprobe controlled using Topspin (version 4.3.0) software. Data were processed in Topspin. Spectra were analysed using Mnova software (version 16.0, Mestrelab Research). Additional NMR experiments are described in Supplementary Methods.

### Analysis of enzyme activity on sugar substrates by NMR

The NPMN cofactor was synthesized in vitro as described above and then incubated with 40 μM NphT for 20 h. A total of 20 µl of the loaded protein was set aside for protein MS to verify loading. NphT was exchanged into water using a PD-10 column, and fractions were combined and concentrated to 6.39 mg ml^−1^ using a 500-µl Modified PES VWR Centrifugal Filter, 10 kDa MWCO (VWR). Reactions (100 µl) contained 45 mM substrate in 20 mM HEPES (pH 7.5). Reactions to screen for activity on the substrates shown in Supplementary Fig. 9 were initiated by the addition of enzyme (20 µM) and incubated for 20 h at 50 °C. On removal from the anerobic chamber, reactions were dried using a Thermo Scientific Savant SC250EXP SpeedVac Concentrator before resuspending in 600 µl D_2_O and transferring to a tube for NMR analysis. Promising substrates (d-galactose, l-arabinose and d-ribose) were then assayed with 95 µM NphT to increase conversion and facilitate product characterization. For assays with butanal and inositol, NPMN cofactor was synthesized in vitro as described in Methods and incubated with 40 μM NphT for 20 h. NphT was purified using size exclusion chromatography, and fractions were combined and concentrated to 100 μM using a 500-µl Modified PES VWR Centrifugal Filter, 10 kDa MWCO (VWR). Reactions (100 µl) contained varying combinations of holo NphT (8 or 10 µM), d-ribose (50 mM), inositol (50 mM) and butanal (30 mM) in 20 mM HEPES pH 7.5 in D_2_O. Reactions proceeded at 50 °C for 18 h before removal from the anaerobic chamber. In total, 500 µl D_2_O was added to each sample before analysis by NMR.

### Lactate racemization assay

Anaerobic in vitro cofactor synthesis was performed as described above. The resulting NPMN cofactor or control with LarC and LarE omitted from the reaction was incubated with 20 μM LarA or NphT for 20 h. Reactions (50 µl) containing 42.3 mM d-lactate in 100 mM MES buffer (pH 6.0) and 0.36 µM enzyme were incubated at 37 °C or 50 °C for 2 h in an anaerobic chamber. The reaction was then removed from the chamber and stopped by incubating at 90 °C for 10 min. Conversion of d-lactate to l-lactate was measured via an l-lactate assay kit (Neogen). The protocol was adapted to 100 µl reaction volumes in Corning 96-well plates. NADH absorbance at 340 nm was read with a Tecan Infinite M Plex plate reader.

### LC–MS analysis of LarA and NphT activity on sugar-derived substrates

Anaerobic in vitro cofactor synthesis was performed as described above. The NPMN cofactor or control with LarC and LarE omitted from the reaction was incubated with 20 μM NphT or LarA for 20 h. Reactions (30 µl) contained 10 mM sugar (l-arabinose or d-galactose) in 3.33 mM HEPES (pH 7.5). Reactions were initiated by the addition of enzyme (1.66 µM final concentration) and allowed to proceed for 20 h at either 50 °C or 37 °C. For the d-ribose reactions, the NPMN cofactor reaction was incubated with 40 μM NphT or LarA for 20 h, and reactions (40 µl) contained 10 mM d-ribose and 5 µM enzyme in 25 mM HEPES pH 7.5. Reactions proceeded for 2 h at 50 °C or 37 °C before quenching and analysis by LC–MS.

### Activity of NphT variants

Anaerobic in vitro cofactor synthesis was performed as described above. The resulting NPMN cofactor was incubated with 40 μM enzyme for 20 h in a volume of 120 µl. Reactions (40 µl) contained 10 mM sugar (d-galactose, l-arabinose or d-ribose) in 25 mM HEPES (pH 7.5). Reactions were initiated by the addition of enzyme (5 µM final concentration) and allowed to proceed for 5 h (arabinose), 4 h (galactose) or 2 h (ribose) at 50 °C. These times were selected to optimize the detection of the lactone species before hydrolysis to the hydroxyacid. In total, 10 µl of the reaction was removed from the chamber and quenched in 70 µl 6:1 acetonitrile:water and prepared for polar metabolite LC–MS analysis as described above.

### LC–MS analysis of reactions with deuterated galactose substrates

Following anaerobic cofactor loading, NphT was purified anaerobically by size exclusion chromatography. Fractions containing NphT were pooled, concentrated to 40 μM using a Pall Nanosep Spin Concentrator (10 kDa MWCO) and used for the activity assay as follows: reactions (35 µl) contained 8 µM enzyme and 10 mM substrate in 20 mM HEPES (pH 7.5). Reactions proceeded for 5 h at 50 °C. A total of 10 µl of the reaction was removed from the chamber, quenched in 70 µl 6:1 acetonitrile:water and prepared for polar metabolite LC–MS analysis as described above. For transhydrogenation assays, reactions (35 µl) proceeded at 50 °C for 5 h with varied substrates as described in the figure legends.

### Estimating number of known iron and nickel enzymes

To estimate the number of characterized iron- and nickel-utilizing enzymes in Fig. 1a, PDBeChem was used to compile PDB ligand entries that contained iron or nickel by inputting Fe or Ni into the formula range search query. Each ligand entry page was scraped for bound protein structures and compiled into a master list of PDB entries containing iron or nickel (Source Data Fig. 1). An R script was used to select entries with a corresponding UniProt entry containing the keyword ‘nickel’ or ‘iron’ in the ligand section (Source Data Fig. 1) and to minimize redundancy (Source Data Fig. 1). Manual inspection was used to remove false positives or duplicates, such as nickel-binding proteins that do not utilize nickel for catalysis. The resulting nickel enzyme list was refined to 12 Enzyme Commission (EC) numbers, corresponding to distinct reactions performed by the ten nickel enzymes known at the time of the analysis (Source Data Fig. 1).

## Online content

Any methods, additional references, Nature Portfolio reporting summaries, source data, extended data, supplementary information, acknowledgements, peer review information; details of author contributions and competing interests; and statements of data and code availability are available at https://doi.org/10.1038/s41557-026-02184-9.

## Supplementary information


Supplementary InformationSupplementary Methods, Figs. 1–45, Tables 1 and 2, Note 1, and Codes 1–4.
Supplementary Data 1All source data for the Supplementary Information in one file.
Supplementary Data 2Raw NMR data for Supplementary Fig. 12.
Supplementary Data 3Raw NMR data for Supplementary Fig. 13.
Supplementary Data 4Raw NMR data for Supplementary Fig. 14.
Supplementary Data 5Raw NMR data for Supplementary Fig. 18.
Supplementary Data 6Raw NMR data for Supplementary Fig. 22.
Supplementary Data 7Raw NMR data for Supplementary Fig. 28.
Supplementary Data 8Raw NMR data for Supplementary Fig. 41.
Supplementary Data 9Raw NMR data for Supplementary Fig. 42.
Supplementary Data 10Raw NMR data for Supplementary Fig. 43.


## Source data


Source Data Fig. 1Table of sourced enzymes from Fig. 1a.
Source Data Fig. 2Normalized and raw deconvoluted protein LC–MS data.
Source Data Fig. 3LC–MS EIC data.
Source Data Fig. 4Normalized and raw deconvoluted protein LC–MS data.
Source Data Fig. 5LC–MS-integrated EICs and *m*/*z* data.
Source Data Fig. 6Sequence data from the SSN.
Source Data Extended Data Fig. 2LC–MS-integrated EICs and kinetics rate values.
Source Data Extended Data Fig. 3Cofactor loading percentage values from LC–MS.
Source Data Extended Data Fig. 4LC–MS integrated EICs and standard curves.


## Data Availability

The data supporting the findings of this study are available within the article and its Supplementary Information. Accession codes for proteins in this study are presented in Supplementary Table 1. The Protein Data Bank (PDB) accession code for the structure of NphT is 9YEB. Should any raw data files be needed in another format, they are available from the corresponding author upon reasonable request. Source data are provided with this paper.

## Code availability

The code used to perform bioinformatics is included in Supplementary Codes 1–4, which can be found in Supplementary Information and via GitHub at https://github.com/zsemersky/Semerskyetal_2026 (ref. ^65^).
