# Supplementary material for: Discovery and characterization of a nickel enzyme family that catalyses intermolecular hydride shuttling
Source: Nat Chem. 2026 Jun 17;18(9):1582–92. doi: 10.1038/s41557-026-02184-9 (PMC13524815; doi:10.1038/s41557-026-02184-9)
Supplement: Supplementary file 1 [file 41557_2026_2184_MOESM1_ESM.pdf]

nature chemistry

Supplementary information https://doi.org/10.1038/s41557-026-02184-9

Discovery and characterization of a nickel enzyme family that catalyses intermolecular hydride shuttling

In the format provided by the authors and unedited

Table of contents

Supplementary Methods

Commercial materials 3
Bioinformatic identification of nickel pincer enzymes 3
In silico docking of ligands with LarA and AKR homologs 4
Sequence alignment of AKR homologs 4
Generation of PF00248 sequence similarity network 4
Bacterial strains 4
Construction of plasmids 4
Expression of His_{10}-tagged and Strep-tagged proteins 5
Purification of His_{10}-tagged proteins 5
Purification of Strep-tagged proteins 6
Preparation of NphT for crystallography 6
In vitro cofactor synthesis for crystallography 6
Cofactor loading for crystallography 7
Crystallization and structure determination 7
Preparing samples for polar metabolite analysis 8
General procedure for high-resolution HPLC/MS analysis of polar metabolites with HILIC 8
Preparing samples for protein mass spectrometry 8
General procedure for high-resolution HPLC/MS analysis of protein samples 9
NMR data collection 9
Analysis of enzyme activity on sugar substrates by NMR 10
Lactate racemization assay 10
LC-MS analysis of LarA and NphT activity on sugar-derived substrates 10
Activity of NphT variants 11
Kinetic analysis of NphT 11
LC-MS analysis of reactions with deuterated galactose substrate 11
Estimating number of known iron and nickel enzymes. 11
Docking studies 12

Supplementary Figures and Tables

Supplementary Table 1. Strains, plasmids, oligonucleotides, and synthetic gene sequences 13
Supplementary Table 2. Crystallography table for NphT 15
Supplementary Figure 1. Bioinformatic workflow for identifying candidates 16
Supplementary Figure 2. Genes that co-occur with the NPMN biosynthetic pathway 17
Supplementary Figure 3. Redox reactions catalyzed by AKRs 18
Supplementary Figure 4. Docking NPMN and NAD(P) into AKR homologs and LarA 19
Supplementary Figure 5. Docking NPMN and NAD(P) into AKR homologs 20
Supplementary Figure 6. Full alignment of AKRs with lysine and histidine highlighted 21
Supplementary Figure 7. Sequence alignment of AKRs adjacent to the NPMN biosynthetic cluster 23
Supplementary Figure 8. *In vitro* synthesis of NPMN for generating holoenzymes 27
Supplementary Figure 9. Panel of sugars used to test the activity of NphT 28

Supplementary Figure 10. NMR reactions to test NphT activity 29
Supplementary Figure 11. LC-MS data for assessing NphT activity with NAD(P)(H) 34
Supplementary Figure 12. NMR reactions to test NphT activity on weak substrates 35
Supplementary Figure 13. ^{1}H NMR kinetics to monitor NphT activity on gluconolactone and sorbitol 36
Supplementary Figure 14. NMR analysis of products from incubating NphT with L-arabinose 37
Supplementary Figure 15. NMR analysis of arabitol standard for comparison to NphT products 39
Supplementary Figure 16. NMR analysis of arabinolactone standard for comparison to NphT products 40
Supplementary Figure 17. NMR analysis of arabonate standard for comparison to NphT products 41
Supplementary Figure 18. NMR analysis of products from incubating NphT with D-galactose 42
Supplementary Figure 19. NMR analysis of galactitol standard for comparison to NphT products 44
Supplementary Figure 20. NMR analysis of galactonolactone standard for comparison to NphT products 45
Supplementary Figure 21. NMR analysis of galactonate standard for comparison to NphT products 46
Supplementary Figure 22. NMR analysis of products from incubating NphT with D-ribose 48
Supplementary Figure 23. NMR analysis of ribitol standard for comparison to NphT products 49
Supplementary Figure 24. NMR analysis of ribonolactone standard for comparison to NphT products 50
Supplementary Figure 25. NMR analysis of ribonate standard for comparison to NphT products 51
Supplementary Figure 26. Assessment of NphT total turnover number 52
Supplementary Figure 27. Product distribution over time 53
Supplementary Figure 28. Quantitative ^{13}C NMR analysis of NphT products 54
Supplementary Figure 29. LC-MS comparison of products to synthetic standards 55
Supplementary Figure 30. LC-MS data for LarA and NphT at 37 °C and 50 °C 56
Supplementary Figure 31. LC-MS analysis of holo NphT prior to crystallography 57
Supplementary Figure 32. Nickel anomalous difference map 58
Supplementary Figure 33. Residues contacting the NPMN cofactor 59
Supplementary Figure 34. Alignment of AKRs in the same SSN cluster as NphT 60
Supplementary Figure 35. Structural comparison of NphT and a canonical AKR 64
Supplementary Figure 36. Size exclusion chromatography to determine oligomeric state of NphT 65
Supplementary Figure 37. Docking of sugar tautomers with NphT 66
Supplementary Figure 38. Cofactor loading 78
Supplementary Figure 39. Structural comparison of NphT and Sp LarA 70
Supplementary Figure 40. Transhydrogenation with L-arabinose and D-[2H]-galactose substrates 71
Supplementary Figure 41. NMR analysis of competition between L-arabinose, D-galactose, and D-ribose 72
Supplementary Figure 42. NMR analysis of transhydrogenation between ribose and butanal 73
Supplementary Figure 43. NMR analysis of inositol incubation with NphT 75
Supplementary Figure 44. SDS-PAGE analysis of purified proteins used in this study 76
Supplementary Figure 45. Raw gel image. 77

Supplementary Note 1 78

References 81

Supplementary Code 1–4 83

Supplementary Methods

Commercial materials. Luria-Bertani (LB) Broth Miller, LB Agar Miller, and Terrific Broth (TB) were purchased from Dot Scientific (Burton, Michigan). Glycerol was purchased from Fisher Scientific (Waltham, MA). DNA fragments were purchased from Twist Biosciences (South San Francisco, CA). Oligonucleotides were purchased from Life Technologies (Carlsbad, CA) or Integrated DNA Technologies (Coralville, IA), resuspended at a stock concentration of 100 µM in elution buffer (Qiagen) and stored at room temperature. Carbenicillin (Cb), kanamycin (Km), chloramphenicol (Cm), and isopropyl-β-D-thiogalactopyranoside (IPTG) were purchased from GoldBio (St. Louis, MO). Sodium chloride, dithiothreitol (DTT), 4-(2-hydroxyethyl)-1-piperazineethanesulfonic acid (HEPES), magnesium chloride hexahydrate, acetonitrile, hydrochloric acid, 3kDa MWCO dialysis tubing, ammonium bicarbonate, sodium acetate, and sodium hydroxide were purchased from Fisher Scientific (Pittsburgh, PA). Adenosine triphosphate sodium salt (ATP), nicotinamide adenine dinucleotide reduced form dipotassium salt (NADH), lysozyme, β-mercaptoethanol (βME), betaine, dimethylsulfoxide (DMSO), sodium L-ascorbate, acetonitrile (LC/MS-grade), ammonium formate (LC/MS-grade), deuterium oxide (D_2O), 4-morpholineethanesulfonic acid (MES), Pall Nanosep centrifugal Omega membrane (10 kDa MWCO), nickel chloride ($NiCl_2$), and 2-(4-Hydroxyphenylazo)benzoic acid (HABA) were purchased from Sigma-Aldrich (St. Louis, MO). Lactate detection kit was purchased from Neogen (Lansing, Michigan). Formic acid was purchased from Acros Organics (Morris Plains, NJ). Restriction enzymes, T4 DNA ligase, Deoxynucleotides (dNTPs), Phusion DNA polymerase, T5 exonuclease, Taq DNA ligase, and Color Prestained Protein Ladder were purchased from New England Biolabs (Ipswich, MA). Ni-NTA agarose was purchased from Qiagen (Valencia, CA). DNA purification kits were purchased from Omega Biotech. Complete EDTA-free protease inhibitor was purchased from Roche Applied Science (Penzberg, Germany). PD-10 desalting columns, Superdex 75 16/60 pg, and Superdex 75 Increase 10/300 GL columns were purchased from Cytiva (Uppsala, Sweden). Amicon Ultra 10,000 MWCO centrifugal concentrators were purchased from Millipore (Billerica, MA). Ultrapure lab water was obtained from an Elga water purification system (Woodridge, IL). 500 µL Modified PES VWR Centrifugal Filters, 10 kDa MWCO were purchased from VWR (Radnor, PA). 12% polyacrylamide gels were purchased from MP Biosciences (Solon, OH). Ultrayield baffled flasks were purchased from Thompson Instrument Company (Oceanside, CA). Streptactin superflow resin, desthiobiotin, and avidin were purchased from IBA (Göttingen, Germany). PLRPS column (1000 Å, 5 µm, 2.1 x 50 mm) was purchased from Agilent. Atlantis Premier BEH Z-HILIC and BEH HILIC Amide were purchased from Waters (Milford, MA). For crystallography, sodium acetate and ammonium chloride were purchased from Fisher Scientific (Pittsburgh, PA), and PEG 3350 was purchased from Sigma-Aldrich (St. Louis, MO). Siliconized glass slides and 24-well dishes for hanging-drop crystallization were purchased from Hampton Research. Zirconia/silica beads for generating microseeds were purchased from BioSpec Products (Bartlesville, OK).

Bioinformatic identification of nickel pincer enzymes. To identify potential NPMN-utilizing candidate enzymes, the InterPro family of LarC homologs (IPR002822) was used as a query in the EFI-EST web tool[1] using option B for BLAST (E-value of 10^{-5}), yielding 11067 hits. The EFI-GNT web tool[1] was then employed to identify genes adjacent to LarC. An R script was used to parse the list to include genomes containing LarB and LarE while removing genomes containing LarA within 20 open reading frames of LarC (the maximum allowable value in the EFI-GNT web tool), resulting in 2108 sequences (**Supplementary Code 1)**. The remaining LarC

sequences were input into the EFI-EST web tool using option D to generate an SSN from UniProt accession IDs. An alignment score threshold of 10^{-18} was used after initial processing. Using Cytoscape v3.10.1, the network was procesed to estimate isofunctional clusters by removing edges based on alignment score value up to 131. The EFI-GNT web tool was used to identify adjacent genes as potential NPMN-utilizing candidates. The output .sqlite file generated from the EFI-GNT web tool above was read into an R script to profile PFAM co-occurrences with the NPMN biosynthesis genes (**Supplementary Code 2**).

***In silico* docking of ligands with LarA and AKR homologs.** The Chai-1-predicted structure of NphT was used as an input for FoldSeek[2] to search structures from the PDB that were most similar. AKR11C1 (PDB: 1YNQ) from *Bacillus halodurans*, was one such close match, with 22.8% sequence identity and an E-Value of 3.37e-17. DiffDock was used to dock the ideal SDF files of $NADP^+$ (NAP), NAD^+ (NAD), and NPMN (represented by PDB ID 4EY, which contains the NPMN ligands) from the PDB into a known AKR from *E. coli* (AKR14A1, Chai-1 prediction), AKR11C1 (PDB: 1YNQ), LarA (PDB: 5HUQ), and NphT (Chai-1 prediction).

Sequence alignment of AKR homologs. The five closest characterized structural homologs to NphT in the PDB were extracted from FoldSeek[2]. NPMN-adjacent AKR homologs with lower sequence identify to NphT were also examined. An MSA of the sequences was generated using Clustal Omega in Clustal format.

Generation of PF00248 Sequence similarity network. To identify the distribution of putative nickel pincer-dependent AKRs containing the histidine/lysine motif shown in NphT, an SSN was generated using EFI-EST[1]. Option D was used to process UniProt accession numbers of characterized AKRs[3] and NphT (**Figure 6 Source Data**). The query was modified to exclude fragments and include Protein Family Addition Options for PF00248, the AKR superfamily for BLAST (E-value of 10^{-5}). UniRef50 was used to condense from 369773 to 30309 sequences. After initial processing, sequences below 150 amino acids in length were excluded from the SSN, resulting in 19356 sequences. Using Cytoscape v3.10.1, the network was processed by removing edges based on alignment score value up to 67 and visualized using the yFiles Organic Layout. An alignment of the sequences was generated using the MAFFT DPPartTree model in FASTA format, then parsed by an R script to extract those containing the histidine/lysine motif characteristic of NphT, of which there were 798. (**Figure 6 Source Data**).

Bacterial strains. Bacterial strains, plasmids, and oligonucleotide sequences are listed in **Supplementary Table 1**. *E. coli* Turbo cells (New England Biolabs) were used for plasmid construction. BL21(DE3) was used for heterologous protein production.

Construction of plasmids. Gibson assembly[4] was used to carry out plasmid construction using *E. coli* Turbo cells (New England Biolabs) as the cloning host. PCR amplifications were carried out with Phusion polymerase (New England Biolabs) using the oligonucleotides listed in **Supplementary Table 1**. Following plasmid construction, all cloned inserts were sequenced at Plasmidsaurus (South San Francisco, CA).

Plasmids for the expression NphT variants. The gene encoding NphT was obtained as a GeneBlock (**Supplementary Table 1**, Twist Biosciences, San Francisco, CA) with the appropriate overhangs for Gibson insertion into NdeI/BamHI-digested His-PrescissionCutSite-IMPDH to generate pNphT. To clone D47A, H144G, K75A, H106A, Y52F, Y52A, K200A, and W218A point mutants, NphT was amplified in two pieces from pNphT. The first piece was amplified with NphT-F and the reverse primer indicated in **Supplementary Table 1**. The second piece was

amplified with the forward primer indicated in **Supplementary Table 1** and NphT-R. Then both pieces were inserted into NdeI/BamHI-digested His-PrescissionCutSite-IMPDH using Gibson cloning to yield the final constructs.

Plasmid for the expression of AKR14A1. L-glyceraldehyde 3-phosphate reductase (AKR14A1, UniProt Q46851) was amplified from genomic DNA of *E. coli* BL21 Star (DE3) using AKR14A1-F and AKR14A1-R. The purified PCR product was inserted into NdeI/BamHI-digested His-PrescissionCutSite-IMPDH using Gibson cloning.

Plasmids for the expression of LarA. LarA was amplified from *L. plantarum* genomic DNA using LarA-F and LarA-Ra. The resulting PCR product was amplified using LarA-F and LarA-Rb to construct the Strep tag. The purified product was inserted in NcoI/BamHI-digested His-PrescissionCutSite-IMPDH using Gibson cloning to yield the final construct.

Plasmids for the expression of LarB, LarC, and LarE. LarC was amplified in two pieces from *L. plantarum* genomic DNA to remove the encoded ribosomal frameshift. The first piece was amplified using LarC1-F and LarC1-R. The second piece was amplified using LarC2-F and LarC2-Ra. The pieces were annealed with overlap extension PCR and amplified using LarC1-F and LarC2-Rb to construct the Strep tag. LarE was amplified from *L. plantarum* genomic DNA using LarE-F and LarE-Ra. The resulting PCR product was amplified using LarE-F and LarE-Rb to construct the Strep tag. LarB was amplified from *L. plantarum* genomic DNA using LarB-F and LarB-Ra. The resulting PCR product was amplified using LarB-F and LarB-Rb to construct the Strep tag. To generate the S127A mutant used in this study, LarB was amplified in two pieces. The first piece was amplified with LarB-F and LarB-S127A-R. The second piece was amplified with LarB-S127A-F and LarB-Rb. The amplified fragments described above were inserted in NcoI/BamHI-digested His-PrescissionCutSite-IMPDH using Gibson cloning to yield the final constructs.

Expression of His_{10}-tagged and Strep-tagged proteins. *E. coli* BL21(DE3) was transformed with the appropriate protein expression plasmid. In the morning, colonies were picked into a 2 mL starter culture of Terrific Broth (TB), grown to mid-log, diluted to 6 mL, and grown to mid-log again. This culture was used to inoculate TB (0.5 L) containing the appropriate antibiotics (100 µg/mL carbenicillin) in a 2.8 L baffled shake flask. The cultures were grown at 37°C at with shaking at 200 rpm to optical density 600 nm (OD_{600}) = 0.6 to 0.8 at which point cultures were cooled on ice for 20 min, followed by induction of protein expression with IPTG (0.25 mM) and overnight growth at 16°C. In the case of LarC, nickel chloride (1 mM final concentration) was added to the media immediately before induction with IPTG. Cell pellets were harvested by centrifugation at 8,000 × *g* for 8 min at 4ºC and stored at -80ºC.

Purification of His_{10}-tagged proteins. Frozen cell pellets were thawed and resuspended at 5 mL/g of cell paste in lysis buffer (50 mM HEPES, 300 mM NaCl, 10 mM imidazole, 20 mM βME, 0.1 mg/mL lysozyme, 20% (*v/v*) glycerol, pH 7.5) supplemented with EDTA-free Protease Inhibitor Cocktail (Roche). The cell paste was homogenized and then lysed by sonication with a Qsonica Q700 sonicator (Amplitude = 45, 5 s on, 25 s off, 2.5 min total process time, ½" tip). The lysate was then centrifuged at 13,500 × *g* for 20 min at 4°C to separate the soluble and insoluble fractions. The soluble lysate was incubated with Ni-NTA (0.5 mL resin/g of cell paste) for 45 min at 4°C, then resuspended and loaded onto a column by gravity flow. The column was washed with wash buffer (50 mM HEPES, 300 mM NaCl, 20 mM imidazole, 20 mM βME, 20% (*v/v*) glycerol, pH 7.5) for 15-20 column vol. The column was then eluted with elution buffer (50 mM HEPES,

300 mM NaCl, 300 mM imidazole, 20 mM βME, 20% (*v/v*) glycerol, pH 7.5). The eluted protein was diluted 2-fold in MilliQ water and incubated with Prescission protease (1 mg protease/50 mg protein) overnight at 4°C to remove the His Tag. The following morning, cleaved protein was diluted to a final imidazole concentration of 20 mM and final NaCl concentration of 20 mM using Buffer A (25 mM HEPES, 2 mM βME, pH 7.5) and loaded onto a 5 mL HiTrap-Q column for ion exchange with the AKTA Pure FPLC system (Cytiva). The protein was eluted using a gradient from 0-100% Buffer A to Buffer B (25 mM HEPES, 1M NaCl, 2 mM βME, pH 7.5) over 40 min. Fractions containing the target protein were pooled by $A_{280\ nm}$ and concentrated using an Amicon Ultra spin concentrator (10 kDa MWCO, Millipore). The NphT variants eluted in 110 mM NaCl and were frozen without buffer exchange. Other proteins were exchanged into storage buffer (50 mM HEPES, 100 mM sodium chloride, 20% (*v/v*) glycerol, 1 mM DTT, pH 7.5) using PD-10 desalting columns. Final protein concentrations before storage were estimated using the $\varepsilon_{280\ nm}$ calculated by ExPASy ProtParam[5] and measured by nanodrop. They are as follows: NphT: 15.11 mg/mL, ($\varepsilon_{280\ nm}$ = 31400 $M^{-1}cm^{-1}$), NphT K200A: 11.90 mg/mL ($\varepsilon_{280\ nm}$ = 31400 $M^{-1}cm^{-1}$), NphT H144G: 15.06 mg/mL ($\varepsilon_{280\ nm}$ = 31400 $M^{-1}cm^{-1}$), NphT D47A: 13.82 mg/mL ($\varepsilon_{280\ nm}$ = 31400 $M^{-1}cm^{-1}$), NphT H106A: 13.99 mg/mL ($\varepsilon_{280\ nm}$ = 31400 $M^{-1}cm^{-1}$), NphT K75A: 12.77 mg/mL ($\varepsilon_{280\ nm}$ = 31400 $M^{-1}cm^{-1}$), NphT W218A: 16.43 mg/mL ($\varepsilon_{280\ nm}$ = 25900 $M^{-1}cm^{-1}$), NphT Y52A: 14.59 mg/mL ($\varepsilon_{280\ nm}$ = 29910 $M^{-1}cm^{-1}$), NphT Y52F: 15.69 mg/mL ($\varepsilon_{280\ nm}$ = 29910 $M^{-1}cm^{-1}$), AKR14A1: 7.21 mg/mL ($\varepsilon_{280\ nm}$ = 59360 $M^{-1}cm^{-1}$). All proteins were aliquoted, flash-frozen in liquid nitrogen, and stored at -80°C.

Purification of Strep-tagged proteins. Frozen cell pellets were thawed, lysed, and clarified as above, except Strep lysis buffer was used instead (100 mM Tris, 150 mM NaCl, 20 mM βME, 20% (*v/v*) glycerol, pH 7.5). The column was washed with wash buffer (100 mM Tris, 150 mM NaCl, 20 mM βME, 10% (*v/v*) glycerol, pH 7.5) for 5 column vol. The column was then eluted with elution buffer (100 mM Tris, 150 mM NaCl, 20 mM βME, 10% (*v/v*) glycerol, 2.5 mM desthiobiotin, pH 7.5). When purifying LarE, the concentration of NaCl was increased to 300 mM in all purification and storage buffers. Final protein concentrations before storage were estimated using the $\varepsilon_{280\ nm}$ calculated by ExPASy ProtParam[5] and measured by nanodrop. They are as follows: *L. plantarum* LarB S127A: 2.86 mg/mL ($\varepsilon_{280\ nm}$ = 14440 $M^{-1}cm^{-1}$), *L. plantarum* LarE: 15.42 mg/mL ($\varepsilon_{280\ nm}$ = 28420 $M^{-1}cm^{-1}$), *L. plantarum* LarC: 17.83 mg/mL ($\varepsilon_{280\ nm}$ = 27515 $M^{-1}cm^{-1}$), *L. plantarum* LarA: 13.18 mg/mL ($\varepsilon_{280\ nm}$ = 31400 $M^{-1}cm^{-1}$). All proteins were aliquoted, flash-frozen in liquid nitrogen, and stored at -80°C.

Preparation of NphT for crystallography. Following elution from a Ni-NTA column, NphT was diluted 2-fold in MilliQ water and incubated with Prescission protease (1 mg protease/50 mg protein) overnight at 4°C to remove the His tag. The following morning, cleaved protein was diluted to a final imidazole concentration of 20 mM and final NaCl concentration of 20 mM using Buffer A (25 mM HEPES, 2 mM βME, pH 7.5) and loaded onto a 5 mL HiTrap-Q column for ion exchange with the AKTA Pure FPLC system (Cytiva). The protein was eluted using a gradient from 0-100% Buffer A to Buffer B (25 mM HEPES, 1M NaCl, 2 mM βME, pH 7.5) over 40 min. The NphT eluted in an NaCl concentration of 90-100 mM. The protein sample was concentrated to 21.17 mg/mL (656 μM) and frozen at -80°C for storage before cofactor loading.

***In vitro* cofactor synthesis for crystallography.** All steps were performed in anaerobic chamber (Coy). Reactions (600 μL) contained NaAD (0.4 mM), sodium bicarbonate (50 mM), and magnesium chloride (0.4 mM) in 100 mM Tris Buffer (pH 7.5). Reactions were initiated by the addition of purified LarB S127A (12 μM final concentration) and allowed to proceed for 20 hours

at room temperature to yield P2CMN. LarB S127A is a variant with higher yield of the target P2CMN product over an unproductive hydrolysis product, but it is slower than wild-type LarB[6]. We therefore perform this step separately to allow P2CMN to accumulate before adding the single-turnover enzyme LarC and LarE. The samples were then centrifuged at 15,000 × *g* for 5 minutes to remove any precipitated LarB. To generate NPMN, reactions (725 µL) contained 196.5 µL of the LarB reaction from step 1, magnesium chloride (2.5 mM), ATP (2.5 mM), βME (5 mM), CTP (0.25 mM), manganese chloride (0.4 mM), nickel chloride (75 µM), LarC (80 µM), and LarE (130 µM). The reactions proceeded for 3.5 hours at room temperature before boiling at 90°C for 10 minutes to remove LarB, LarC, and LarE. The sample was vortexed for 2 seconds, and the precipitated LarB, LarC, and LarE were removed by centrifugation at 13,000 × *g* for 10 minutes. The presence of βME helped to retain NPMN in the soluble fraction after boiling, consistent with literature results[7]. The supernatant (650 µL) was transferred to a fresh tube and used as the NPMN source for loading NphT for crystallography as described below.

Cofactor loading for crystallography. In an anaerobic chamber (Coy), the NPMN cofactor was incubated with 40 µM NphT in a final volume of 685 µL for 18 hours. The following day, the sample was centrifuged at 13,000 × *g* for 5 min to remove precipitate. The remaining soluble holo protein was concentrated with a Pall Nanosep 10k centrifugal filter to a volume of 400 µL and loaded onto a Superdex™ 75 Increase 10/300 GL column (Cytiva) equilibrated with Buffer C (20 mM HEPES, 100 mM NaCl) and eluted at a flow rate of 0.4 mL/min. Fractions containing NphT were pale orange. NphT samples were combined and concentrated to 10 mg/mL using a 0.5 mL Amicon Ultra Centrifugal Filter, 10 kDa MWCO (Millipore). Glycerol was added to 3% (*v/v*), and the protein was immediately crystallized as described below.

Crystallization and structure determination. Apo crystals of NphT were first obtained by the hanging drop vapor diffusion method by combining equal volumes of apo protein solution (4 mg/mL NphT) and reservoir solution (sodium acetate pH 4.6 (100 mM), ammonium chloride (200 mM), 22% (*w/v*) PEG 3350). Thin plates and rectangular prisms grew within 5 days. Crystals were transferred to an Eppendorf tube containing 250 µL of reservoir solution and vortexed for 30 seconds with 10 x 1 mM diameter zirconia/silica beads to produce a micro-seed solution. The seed solution was divided into 50 µL aliquots and stored at -80°C for future use.

The microseeds described above were then used to seed the growth of NPMN-loaded NphT crystals. NPMN-bound NphT crystals were prepared in a Coy Anaerobic chamber by micro-seeding equal volumes of protein solution (4 mg/mL NphT in Buffer C) and reservoir solution (sodium acetate pH 4.5 (100 mM), ammonium chloride (200 mM), 25.5% (*w/v*) PEG 3350). Needles, rectangular rods, and plates grew within 2 days. Crystals continued to grow until day 8 before looping and flash-freezing in liquid nitrogen. Crystals were not cryoprotected, but the protein sample contained 3% glycerol from the storage buffer. Data were collected at Beamline 8.3.1 at the Advanced Light Source (Lawrence Berkeley National Laboratory) at wavelengths of 1.11 Å and 1.48 Å.

Data were processed with XDS[8] and scaled and merged with Aimless within the CCP4 suite[9]. The data were phased with molecular replacement in the Phaser module of Phenix[10] using the AlphaFold3[11]-predicted structure of NphT (Uniprot accession A0A917NHY8) as a search model. There are two molecules of NphT per asymmetric unit. The structure was refined to 1.3 Å resolution iteratively using COOT[12] for model building, followed by Phenix for refinement. All residues of the coding sequence of NphT are modeled. Our construct has a 3-amino acid scar (GPH) on the N-terminus remaining from Prescission protease cleavage of the His Tag. These

three residues are visible in Chain A, but only the His is visible from the scar in Chain B. Waters were added manually using Coot during the later rounds of refinement, after the protein backbone and sidechains were built. Waters were placed where there was clear density in the F_o-F_c map (>3 σ), and water placement was inspected after refinement. Restraints for the NPMN ligand (PDB 4EY) were generated using the Elbow program within Phenix. NPMN (nickel and the 4EY ligand) was added to the model using Coot, and the structure was further refined using Phenix. During refinement, the occupancy of NPMN was set to 1, based on LC-MS analysis of the holoenzyme preparation, and individual B-factors were refined (**Supplementary Fig. 31**).

Omit maps for NPMN were generated using the Phenix Composite Omit Map function within Phenix. Parameters were as follows: Omit map method: Simple; Fraction of atoms to omit: 0.05; Exclude bulk solvent from omit regions = on. Phenix was then used to generate a map around NPMN, His144, and Lys200. Anomalous maps were generated using the dataset collected at 1.48 Å. Overall structural alignments were performed using the Chimera software (UCSF v1.7.1)[13]. Alignments of NphT (PDB 9YEB) and AKR14A1 (PDB 4AUB) were performed using the Match Function in Chimera. Parameters: Chain pairing: bb; Alignment algorithm: Needleman-Wunsch; Similarity matrix: BLOSUM-62. RMSD between 125 pruned atom pairs is 1.043 Å. RMSD cross all 262 atom pairs is 5.597 Å. The structures were analyzed in PyMol[14], which was also used to create figures. In the structural analyses, residue numbering is based on the NphT sequence.

Preparing samples for polar metabolite analysis. Reactions were initiated by addition of purified enzyme and allowed to proceed at the indicated times and temperatures. Samples were then quenched in 3 volumes of acetonitrile, vortexed and treated with three freeze-thaw cycles (dry ice, then room temp) to precipitate protein. Next, 0.5 volumes of methanol were added, and the samples were vortexed and centrifuged at 15,000 × *g* for 15 min to remove precipitated protein. Samples were then analyzed by LC/MS on an Agilent 1290 UPLC-6546 QTOF using the protocol for polar metabolite analysis below.

General procedure for high-resolution HPLC/MS analysis of polar metabolites with HILIC. Samples containing polar compounds were analyzed using an Agilent 1290 UPLC/6546 QTOF. Chromatography was performed using an Atlantis Premier BEH Z-HILIC column or an Atlantis Premier BEH HILIC Amide column (1.7 µm, 2.1 × 150 mm; Waters) using Buffer A (100% water, 15 mM ammonium bicarbonate, pH 9) and Buffer B (90% acetonitrile, 10% water, 15 mM ammonium bicarbonate, pH 9) with a 25 minute method, followed by a 5-min post-run equilibration. The gradient profile was as follows: 10% Buffer A held for 2 min, followed by linear increases to 15% A at 3 min, 20% A at 5 min, 25% A at 6 min, followed by an isocratic hold until 7 min. The gradient was linearly increased to 30% buffer A at 8 min and held until 9 min, followed by an increase to 50% A by 10 minutes and another isocratic hold until 12 min. Buffer A was further increased to 70% by 13 min and held until 16 min, then increased again to 98% by 18 min and held until 23 min. The column was re-equilibrated to 10% Buffer A between 23 and 25 min, followed by a 5 min hold at the same buffer concentration. The method was applied at a flow rate of 0.2 mL/min. Mass spectra were acquired in negative ionization mode using the 6546 QTOF.

Preparing samples for protein mass spectrometry. Protein samples were desalted using Zeba™ Spin Desalting Columns. Briefly, the 0.05% sodium azide storage solution was removed by centrifugation at 1500 × *g* for 1 minute, followed by equilibration with 3 x 300 µL of milliQ water. The column was then placed in a clean collection tube, and 20-30 µL of the protein sample (20-30 µM) was applied to the resin bed followed by 15 µL of water. The sample was centrifuged at 1500 × *g* for 2 minutes. The flowthrough was applied to the bed of a second equilibrated column

and stacked with 15 µL of water. The sample was again eluted by centrifugation at 1500 × *g* for 2 minutes. The sample was combined 1:1 with LCMS grade 100% acetonitrile, centrifuged at 10,000 × *g* for 10 min to remove precipitates, and transferred into an HPLC vial. Samples were then analyzed by LC/MS on an Agilent 1290 UPLC-6546 QTOF using the protocol for protein mass spectrometry analysis below.

General procedure for high resolution HPLC/MS analysis of protein samples. Samples containing protein were analyzed using an Agilent 1290 UPLC equipped with a PLRPS column (1000 Å, 5 µm, 2.1 x 50 mm; Agilent) using Buffer A (100% water containing 0.1% formic acid) and Buffer B (100% acetonitrile). The method was 9.5 min long, followed by a 2 min post-run equilibration. The gradient profile was as follows: a linear gradient from 20% to 60% Buffer B from 0 to 4 min followed by a linear increase of B to 90% at 5 min. The gradient was linearly increased to 95% B from 5 to 6.5 min with an isocratic hold until 7.8 min. The column was re-equilibrated to 20% Buffer B from 7.8 min to 9.5 min, followed by a 2 min post-run hold at the same concentration. The method was applied at a flow rate of 0.350 mL/min. Mass spectra were acquired in positive ionization mode using a 6546 QTOF (Agilent) and deconvoluted using Bioconfirm (v12.1, Agilent) via the Maximum Entropy algorithm with a mass range of 20000.00-160000.00 Daltons and mass step of 1.0000 Daltons. Additional deconvolution settings included a limited *m/z* range of 600.0000-5000.0000 *m/z*, baseline subtraction factor of 7.00, an automatic isotope width of 20.0000 Daltons, and a default peak filter of >= 30 signal-to-noise.

NMR data collection. The initial sugar screening reaction spectra (^{1}H and ^{13}C) were recorded on a Bruker Avance III HD 500 MHz spectrometer using a DCH LHe cryoprobe. Final experiment and standard spectra (^{1}H, ^{13}C, q^{13}C, HH-COSY, HC-HSQC, HC-HMBC) were acquired on a Bruker Avance III HD 600 MHz spectrometer equipped with a TCI-F cryoprobe. Both instruments were controlled using Topspin (version 3.5.7) software. The gluconolactone and sorbitol kinetics reaction spectra (^{1}H) were recorded on a Bruker Avance NEO 500 MHz spectrometer with a Prodigy BBO cryoprobe controlled using Topspin (version 4.3.0) software. Data were processed in Topspin by zero-filling once in each dimension, followed by apodization, Fourier transformation, and phasing. Spectra were analyzed using Mnova software (version 16.0, Mestrelab Research, Santiago de Compostela, Spain).

1D ^{1}H spectra at 298 K were recorded using the Bruker pulse program zg30 with a spectral width of 20 ppm, centered at 6.175 ppm, an acquisition time of 3.3 s (500MHz) or 2.7 s (600MHz), relaxation delay of 1s, and 8 scans. Kinetic 1D ^{1}H spectra at 323 K were recorded using the Bruker pulse program kin1d with a spectral width of 20 ppm, centered at 6.175 ppm, and acquisition time of 3.3 s, relaxation delay of 1 s, and 8 scans. 1D ^{13}C spectra at 258 K were recorded using the pulse program zgpg30 with a spectral width of 285 ppm, centered at 110 ppm, an acquisition time of 1s, relaxation delay of 2s and between 32 to 600 scans. Gradient HH-COSY experiments using Bruker's cosygp45ppqf pulse program were run with time dimensions of 2048 x 256, a relaxation delay of 1.5 s, and 2 scans. Sweep width was optimized with the automation program au_gelcosy. HC-HSQC data was acquired with the pulse program hsqcedetgpsisp2p3 with time dimensions of 2048 x 256, a relaxation delay d1 of 2 s, and 4 scans. Sweep width was optimized with the automation program au_gelinv. HMBC spectra were taken with dimensions of 4096 x 256, a d1 of 2s, and 4 scans using the pulse program hmbcetgpl3nd. No automatic sweep width optimization was performed. Quantitative ^{13}C measurements were carried out after determination of the T1 relaxation delay using the pulse program zgig, a d1 of 14 s, aq of 1 s and 4096 scans. The T1

relaxation of ^{13}C was determined to be 2.35 seconds using a pseudo 3D setup of HSQC experiments (with the Bruker pulse program hsqct1etgpsi3d.2) as described in the literature[15,16].

Analysis of enzyme activity on sugar substrates by NMR. The NPMN cofactor was synthesized *in vitro* as described above and then incubated with 40 μM NphT for 20 hours. 20 μL of the loaded protein was set aside for protein mass spectrometry to verify loading. NphT was exchanged into water using a PD-10 column, and fractions were combined and concentrated to 6.39 mg/mL using a 500 μL Modified PES VWR Centrifugal Filter, 10 kDa MWCO (VWR). Reactions (100 μL) contained 45 mM substrate in 20 mM HEPES (pH 7.5). Reactions to screen for activity on the sugar-derived substrates listed in **Supplementary Fig. 9** were initiated by the addition of enzyme (20 μM) and incubated for 20 hours at 50°C. Upon removal from the anaerobic chamber, reactions were dried using a Thermo Scientific Savant SC250EXP SpeedVac Concentrator before resuspending in 600 μL of D_2O and transferring to a tube for NMR analysis. Control reactions where LarE and LarC were omitted from NPMN synthesis were processed concurrently. Promising substrates (D-galactose, L-arabinose, D-ribose) were then assayed with 95 μM NphT to increase conversion and facilitate product characterization. For assays with butanal and inositol, NPMN cofactor was synthesized *in vitro* as described in the methods and incubated with 40 μM NphT for 20 hours. NphT was purified using size exclusion chromatography, and fractions were combined and concentrated to 100 μM using a 500 μL Modified PES VWR Centrifugal Filter, 10 kDa MWCO (VWR). Reactions (100 μL) contained varying combinations of holo NphT (8 or 10 μM), D-ribose (50 mM), inositol (50 mM) and butanal (30 mM) in 20 mM HEPES pH 7.5 in D_2O. Reactions proceeded at 50°C for 18 hours before removal from the anaerobic chamber. 500 μL D_2O was added to each sample prior to analysis by NMR.

Lactate racemization assay. LarA and NphT were assayed for their ability to convert D-lactate to L-lactate. In a Coy anaerobic chamber, *in vitro* cofactor synthesis was performed as described above. The resulting NPMN cofactor or control with LarC and LarE omitted from the reaction ("-NPMN") was incubated with 20 μM LarA or NphT for 20 hours. A portion of the loaded protein was set aside for protein mass spectrometry to verify loading. Reactions (50 μL) containing 42.3 mM D-lactate in 100 mM MES buffer (pH 6.0) and 0.36 μM enzyme were incubated at 37°C or 50°C for 2 hours. The samples were then removed from the Coy chamber and the reaction was stopped by incubating at 90°C for 10 min. Conversion of D-lactate to L-lactate was measured via a L-lactate assay kit (Neogen) that oxidizes L-lactate to pyruvate, converting NAD^+ to NADH in the process. The protocol was adapted to 100 μL reaction volumes in Corning 96-well plates, where 4.46 μL of reaction sample was added per well. A standard curve of L-lactate at final concentrations of 0, 10, 100, 200, and 300 μM was prepared, and NADH absorbance at 340 nm was read with a Tecan Infinite M Plex plate reader.

LC-MS analysis of LarA and NphT activity on sugar-derived substrates. In a Coy anaerobic chamber, *in vitro* cofactor synthesis was performed as described above. The NPMN cofactor or control with LarC and LarE omitted from the reaction was incubated with 20 μM NphT or LarA for 20 hours. A portion of the loaded protein was set aside for protein mass spectrometry to verify loading. Reactions (30 μL) contained 10 mM sugar (L-arabinose or D-galactose) in 3.33 mM HEPES (pH 7.5). Reactions were initiated by the addition of enzyme (1.66 μM final concentration) and allowed to proceed for 20 hours at either 50°C or 37°C. For the D-ribose reactions, conditions were optimized to increase the yield of the lactone product before hydrolysis. The NPMN cofactor reaction was incubated with 40 μM NphT or LarA for 20 hours. Reactions (40 μL) contained 10 mM D-ribose and 5 μM enzyme in 25 mM HEPES pH 7.5 and proceeded for

2 hours at 50°C or 37°C. Once removed from the anaerobic chamber, 15 µL of the reaction was quenched with 45 µL of 100% LC-MS-grade acetonitrile and freeze-thawed on dry ice for a total of 3 times. After the final thaw, 10 µL of methanol was added and the sample was briefly vortexed, followed by centrifugation for 10 minutes at 13000 × *g* before transfer of the supernatant into an HPLC vial.

Activity of NphT variants. In a Coy anaerobic chamber, *in vitro* cofactor synthesis was performed as described above. The resulting NPMN cofactor was incubated with 40 µM enzyme for 20 hours in a volume of 120 µL. 30 µL of the loaded protein was used to verify loading by LC/MS as described above. Reactions (40 µL) contained 10 mM sugar (D-galactose, L-arabinose, or D-ribose) in 25 mM HEPES (pH 7.5). Reactions were initiated by the addition of enzyme (5 µM final concentration) and allowed to proceed for 5 hours (L-arabinose), 4 hours (D-galactose), or 2 hours (D-ribose) at 50°C. These times were selected to optimize the detection of the lactone species before hydrolysis to the hydroxyacid. 10 µL of the reaction was removed from the chamber and quenched in 70 µL of 6:1 acetonitrile:water and freeze-thawed on dry ice three times. After the final thaw, 10 µL of methanol was added and the sample was briefly vortexed and then centrifuged for 10 minutes at 13000 × *g* before transfer of the supernatant into an HPLC vial for LC-MS analysis.

Kinetic analysis of NphT. In a Coy anaerobic chamber, NphT was loaded with NPMN as described in the methods. Following loading, NphT was purified anaerobically on a size exclusion column to remove unbound small molecules. The protein was concentrated to 100 µM stock concentration. Reactions were set up as follows: Substrate stocks of varying concentration were pre-heated to 50°C for 15 min in PCR strips. Separately, enzyme and HEPES pH 7.5 were mixed and heated. At t=0, 37.33 µL substrate was added to 18.67 µL enzyme in a final volume of 56 µM using a multichannel pipet. Final reactions contained 5 µM enzyme, 40 mM HEPES pH 7.5, and substrate concentrations ranging from 5 mM to 500 mM. At t = 15, 60, 120, and 240 seconds, 10 µL samples of the reaction were quenched in 70 µL of 6:1 acetonitrile:water with 1% formic acid. Samples were freeze-thawed at -80°C before adding 10 µL methanol and centrifuging at 20,000 *x g* for 7 min. Supernatant was transferred to LCMS vials for analysis on a HILIC column (Waters).

LC-MS analysis of reactions with deuterated galactose substrate. In a Coy anaerobic chamber, *in vitro* cofactor synthesis was performed as described above. The resulting NPMN cofactor was incubated with 40 µM enzyme for 20 hours in a total volume of 335 µL. The loaded protein was purified on a Superdex™ 75 Increase 10/300 GL column (Cytiva) equilibrated with Buffer C (20 mM HEPES, 100 mM NaCl) and eluted at a flow rate of 0.4 mL/min. Fractions containing NphT were pooled, concentrated to 40 µM using a Pall Nanosep Spin Concentrator (10 kDa MWCO), and used for the activity assay as follows: Reactions (35 µL) contained 8 µM enzyme and 10 mM substrate (D-[1-^{1}H]-galactose, D-[1-^{2}H]-galactose, or 5 mM each) in 20 mM HEPES (pH 7.5). Reactions proceeded for 5 hours at 50°C. 10 µL of the reaction was removed from the chamber, quenched in 70 µL of 6:1 acetonitrile:water, and freeze-thawed on dry ice three times. 10 µL of methanol was added, the sample was briefly vortexed, and then the sample was centrifuged for 10 minutes at 13000 × *g* before transfer of the supernatant into an HPLC vial. For transhydrogenation assays, reactions (35 µL) contained NphT (5 µM), 20 mM HEPES pH 7.5, and 10 mM of the following substrates: A) L-[1-H]arabinose, B) D-[1-H]galactose, C) D-[1-D]-galactose, D) L-[1-H]arabinose and D-[1-H]galactose, E) L-[1-H]arabinose and D-[1-D]galactose. Following incubation at 50°C for 5 h, 10 µL of the reaction were quenched and analyzed as described for the deuterated galactose samples.

Estimating number of known iron and nickel enzymes. To estimate the number of characterized iron- and nickel-utilizing enzymes in Fig. 1a, PDBeChem was used to compile PDB ligand entries that contained iron or nickel, respectively, by inputting Fe or Ni into the formula range search query. Each ligand entry page was systematically scraped for bound protein structures, and these were compiled into a master list of PDB entries containing iron or nickel (**Source Data Figure 1**). An R script was used to parse this list, retaining only entries with a corresponding UniProt entry containing the keyword “nickel” or “iron” in the ligand section (**Supplementary Code 4**). The script also removed duplicate EC numbers to ensure functionally distinct enzymes were counted only once, yielding 354 distinct EC numbers for iron enzymes and 25 distinct EC numbers for nickel enzymes (**Source Data Figure 1**). Manual inspection of the nickel enzyme list revealed that some entries were false positives or duplicates, such as nickel-binding proteins that do not utilize nickel for catalysis. The resulting nickel enzyme list was refined to 12 EC numbers, corresponding to distinct reactions performed by the 10 characterized nickel enzymes known at the time of analysis (Aug. 2025, **Source Data Figure 1**).

Docking studies. Molecular docking was performed using AutoDock Vina 1.2.7[17,18]. The receptor was obtained as the chain B of the crystal structure of NphT (PDB accession: 9YEB). The water molecules were removed before preparing the receptor using the AutoDock Tools from the AutoDock Suite[19]. The ligands were input as SMILES strings into the Scrubber script (https://github.com/forlilab/molscrub) to generate the 3D conformers of the ligand, which are then parameterized using the Meeko package[20]. The active site of NphT was contained in a cubic grid box of dimensions 15 Å x 15 Å x 15 Å with 0.375 Å spacing, centered around the position -43.928 x 3.180 x 37.358, which corresponds to the Ni center of NPMN. The exhaustiveness parameter was set to 32 and the rest of the docking parameters were set to default. The results were analyzed using PyMol. The top nine docking poses were evaluated for providing a reasonable estimate of the distance for hydride transfer.

Supplementary Results

Supplementary Table 1. Strains, plasmids, oligonucleotides, and synthetic gene sequences. (A) Strains and plasmids used in this study. (B) Oligonucleotides used for plasmid construction and sequencing. (C) Sequences of synthetic genes. (D) Uniprot accession numbers.

A. Strains and plasmids

Strain	Description	Source
BL21 Star (DE3)	*F-ompT hsdSB (rB-, mB-) gal dcm rne131* (DE3)	Thermo-Fisher
Turbo	F' *proA$^+$B$^+$ lacI^q ΔlacZM15 / fhuA2 Δ(lac-proAB) glnV galK16 galE15 R(zgb-210::Tn10)*Tet^S *endA1 thi-1 Δ(hsdS-mcrB)5*	New England Biolabs
Alicyclobacillus cellulosilyticus JCM 18487	*Alicyclobacillus cellulosilyticus* Kusube *et al.* 2014	JCM
L. plantarum (genomic DNA)	Genomic DNA from *Lactiplantibacillus plantarum* strain NCIMN 8826, catalog no. BAA-793D-5	ATCC

Plasmid	Description	Source
pLarB S127A	LarB S127A -Strep from *L. plantarum* (T7), *lacI*, Cb^R, ColE1	This study
pLarC	Strep-LarC from *L. plantarum* (T7), *lacI*, Cb^R, ColE1	This study
pLarE	LarE-Strep from *L. plantarum* (T7), *lacI*, Cb^R, ColE1	This study
pLarA	LarA-Strep from *L. plantarum* (T7), *lacI*, Cb^R, ColE1	This study
pNphT	His_{10}-NphT from *A. cellulosilyticus*, Cb^R	This study
pNphT H144G	His_{10}-NphT H144G (T7), *lacI*, Cb^R, ColE1	This study
pNphT D47A	His_{10}-NphT D47A (T7), *lacI*, Cb^R, ColE1	This study
pNphT H106A	His_{10}-NphT H106A (T7), *lacI*, Cb^R, ColE1	This study
pNphT K75A	His_{10}-NphT K75A (T7), *lacI*, Cb^R, ColE1	This study
pNphT K200A	His_{10}-NphT K200A (T7), *lacI*, Cb^R, ColE1	This study
pNphT W218A	His_{10}-NphT W218A (T7), *lacI*, Cb^R, ColE1	This study
pNphT Y52A	His_{10}-NphT Y52A (T7), *lacI*, Cb^R, ColE1	This study
pNphT Y52F	His_{10}-NphT Y52F (T7), *lacI*, Cb^R, ColE1	This study
pAKR14A1	His_{10}-L-glyceraldehyde 3-phosphate reductase (T7), *lacI*, Cb^R, ColE1	This study
pET16b-His-Prescission-IMPDH	His_{10}-PrescissionCutSite-IMPDH (T7), *lacI*, Cb^R, ColE1	This study

B. Oligonucleotide sequences

Name	Sequence
LarB-F	aactttaagaaggagatataccatggcaaccacagcagaaatattacaac
LarB-Ra	gggtggctccagcttgccatacctgcacccatttgattgaccatactagctgagtaggc
LarB-Rb	ttcgggctttgttagcagccggatccttacttttcgaactgcgggtggctccagcttgcc
LarB-S127A-F	ggggaccgccgatcaaccggttgctg
LarB-S127A-R	ggttgatcggcggtccccgccgtcac
LarE-F	aactttaagaaggagatataccatggcaacattagcaacaaaaaaagcaac
LarE-Ra	ggctccagcttgccatacctgcaccggcgaaagtggccaattgtgc
LarE-Rb	ttcgggctttgttagcagccggatccttacttttcgaactgcgggtggctccagcttgcc
LarC1-F	gtttaactttaagaaggagatataccatggcaagctggagccacccgcagttcga

LarC1-R	gatttgctgacttagtttttttttttcgaataatacggctcgcagt
LarC2-F	cgtattattcgaaaaaaaaaaactaagtcagcaaatcgttaaccgg
LarC2-Ra	gctttgttagcagccggatcctcattacgcctcctcatctaattgatctaccg
LarC2-Rb	agccacccgcagttcgaaaagggtgcaggtatgcaaacactttatttagacgctttttcg
LarA-F	gtttaactttaagaaggagatataccatgtccgttgcaattgatttacc
LarA-Ra	ttctcaaattgtggatgactccagctagccttcacaataacgcctaaaccatcagg
LarA-Rb	cgggctttgttagcagccggatccttacttctcaaattgtggatgactccag
AKR14A1-F	gctttttcagggcccgcatatggtctggttagcgaatcccg
AKR14A1-R	gctttgttagcagccggatcctcatttatcggaagacgcctgc
NphT-F	gctttttcagggcccgcat
NphT-R	gctttgttagcagccggatcc
pNphT K200A-F	gatggtcataaggccgacatcct
pNphT K200A-R	gatgtcggccttatgaccatcgcagccatcgcgcgcg
pNphT H144G-F	ggtcatttagcccctaaagtgc
pNphT H144G-R	actttaggggctaaatgacctccacccgtaataccgatgtgtttc
pNphT D47A-F	actgctgcaagttatggtgagtc
pNphT D47A-R	caccataacttgcagcagtagcaaaatgattaatccctgagtcaagtgc
pNphT H106A-F	gctgtaacgtcatttgaggagttag
pNphT H106A-R	cctcaaatgacgttacagcagcaagttgcatcaaatcgacactatctac
pNphT K75A-F	actggtgagcgtacgcg
pNphT K75A-R	tgatcatcgaacggttcataagcagtagcatactttttgcgtcctc
pNphT W218A-F	tatgaaccgttcgatgatcaagaac
pNphT W218A-R	tgatcatcgaacggttcataagcagtagcatactttttgcgtcctc
pNphT Y52A-F	ggtgagtccgagttacgtttg
pNphT Y52A-R	aaacgtaactcggactcaccagcacttgcagcagtatcaaaatgattaatc
pNphT Y52F-F	ggtgagtccgagttacgtttg
pNphT Y52F-R	aaacgtaactcggactcaccaaaacttgcagcagtatcaaaatgattaatc

---

C. Synthetic gene sequences

Gene block for A0A917NHY8 (pNphT, *E. coli* codon optimized). Homology for Gibson.

```
GAAGTGCTTTTTCAGGGCCCGCATATGGAGACACGTCGTTTGGGCCGTATCGGACATATGAGTTCGGTGATCATTTT
TGGTGCTGCCTCTTTGTCGGACGTGAGCCAGGAAGAGGCGGATGCATCCATCCAGTTCGCACTTGACTCAGGGATTA
ATCATTTTGATACTGCTGCAAGTTATGGTGAGTCCGAGTTACGTTTGGGACCTTGGATGCCGAAAATCCGCAATAAG
ATCTTCTTGGCGACGAAAACTGGTGAGCGTACGCGTGACGAGGCCATGCGCTCGATCGAGCGTTCTCTGAAGCGCTT
ACAGGTAGATAGTGTCGATTTGATGCAACTTCACGCTGTAACGTCATTTGAGGAGTTAGACCAATGCACTCGTAAAG
GAGGTGCTTTGGAAGCTGCTATCGCTGCGAAGGAACAGGGTATGGTGAAACACATCGGTATTACGGGTCATGGTCAT
TTAGCCCCTAAAGTGCATTTAGAAGCGCTTCGTCGCTACCCCTTTGAAACAGTTTTGACCCCATACAACTTCATCTT
GTATAATAACCCGGAATATCGCCAGGCATTTGACGCCCTTGTTGAGGAGGTGAAACGCCAGGATGTCGGCCTTATGA
CCATCAAAGCCATCGCGCGCGGGCCTTGGCCATCCGAGGACGCAAAAAAGTATGCTACTTGGTATGAACCGTTCGAT
GATCAAGAACACATTGATCGCTGTGTATCATTTGTCCTTTCACGCCCTGAAATTACGGGGCTGGCAAGTGCGGGAGA
TGTTCGCCTTTTGCCAAAGATCGTTGAGGCATACAAGCGTTATCGTAAACTTAGCGGGGAGGAACAGGAAGCGCTTA
TGGCTACAGCGGGAAGCTATGCTTCTCCGTTCGGGCCGTTCTAAGGATCCGGCTGCTAACAAAG
```

D. Uniprot accession numbers for proteins in this study

NphT: A0A917NHY8
AKR14A1: Q46851
LarA: F9USS9
LarB: F9UST0
LarC: F9UST1
LarE: F9UST4

Supplementary Table 2. Crystallography table for NphT. Data collection and refinement parameters for NphT bound to NPMN (PDB ID 9YEB). All residues of the coding sequence of NphT are modeled. Our construct has a 3-amino acid scar (GPH) on the N terminus remaining from Prescission protease cleavage of the His Tag. These three residues are visible in Chain A, but only His is visible from the scar in Chain B. The nickel peak was collected at 1.483540 Å and used to generate the anomalous difference map found in Supplementary Figure 32. Both data sets were collected using the same single crystal.

	NphT•NPMN (9YEB)	Ni peak[†]
**Data collection**		
Space group	C 1 2 1	C 1 2 1
Cell dimensions		
a, *b*, *c* (Å)	147.60, 49.15, 79.22	147.58, 49.07, 79.10
α, β, γ (°)	90.00, 117.34, 90.00	90.00, 117.37, 90.00
Resolution (Å)	70.37–1.30 (1.32–1.30)*	70.25–1.70 (1.73–1.70)*
R_{sym} or R_{merge}	0.067 (0.803)	0.055 (0.257)
$I / \sigma I$	13.7 (2.1)	17.3 (5.5)
Completeness (%)	99.3 (97.6)	99.1 (95.8)
Redundancy	6.6 (6.6)	6.6 (5.7)
$CC_{1/2}$	0.999 (0.813)	0.999 (95.8)
**Refinement**		
Resolution (Å)	70.37–1.30	
No. reflections	123212	
R_{work} / R_{free}	0.145/0.167	
No. atoms	5896	
Protein	4948	
Ligand/ion	52	
Ni	2	
4EY(pincer ligand)	48	
Na	2	
Water	896	
B-factors (Å^2)	12.93	
Protein	10.86	
Ligand/ion	7.9	
Ni	8	
4EY (pincer ligand)	7.25	
Na	16	
Water	24.61	
R.m.s. deviations		
Bond lengths (Å)	0.006	
Bond angles (°)	0.990	
Ramachadran plot		
Favored (%)	98.44	
Allowed (%)	1.56	
Outliers (%)	0	
Rotamer outliers	0.58	

*Values are reported for the highest-resolution shell.
[†]Bijvoet pairs were not merged during data processing.

Supplementary Figure 1. Bioinformatic workflow for identifying candidates. To identify potential NPMN-utilizing candidate enzymes, the InterPro family of LarC homologs (IPR002822) was used as an input in the EFI-EST[1] web tool using option B for BLAST (E-value of 10^{-5}), yielding 11067 hits. The EFI-GNT[1] web tool was then employed to identify genes adjacent to LarC. An R script was used to parse the list to include genomes containing LarB and LarE but remove genomes containing LarA within 20 open reading frames of LarC, resulting in 2108 sequences (**Supplementary Code 1**). The remaining LarC sequences were input into the EFI-EST web tool using option D to generate an SSN from UniProt accession IDs. An alignment score threshold of 10^{-18} was used after initial processing. Using Cytoscape v3.10.1, the network was processed to estimate isofunctional clusters by removing edges based on alignment score value up to 131. The EFI-GNT web tool was used to identify adjacent genes as potential NPMN-utilizing candidates.

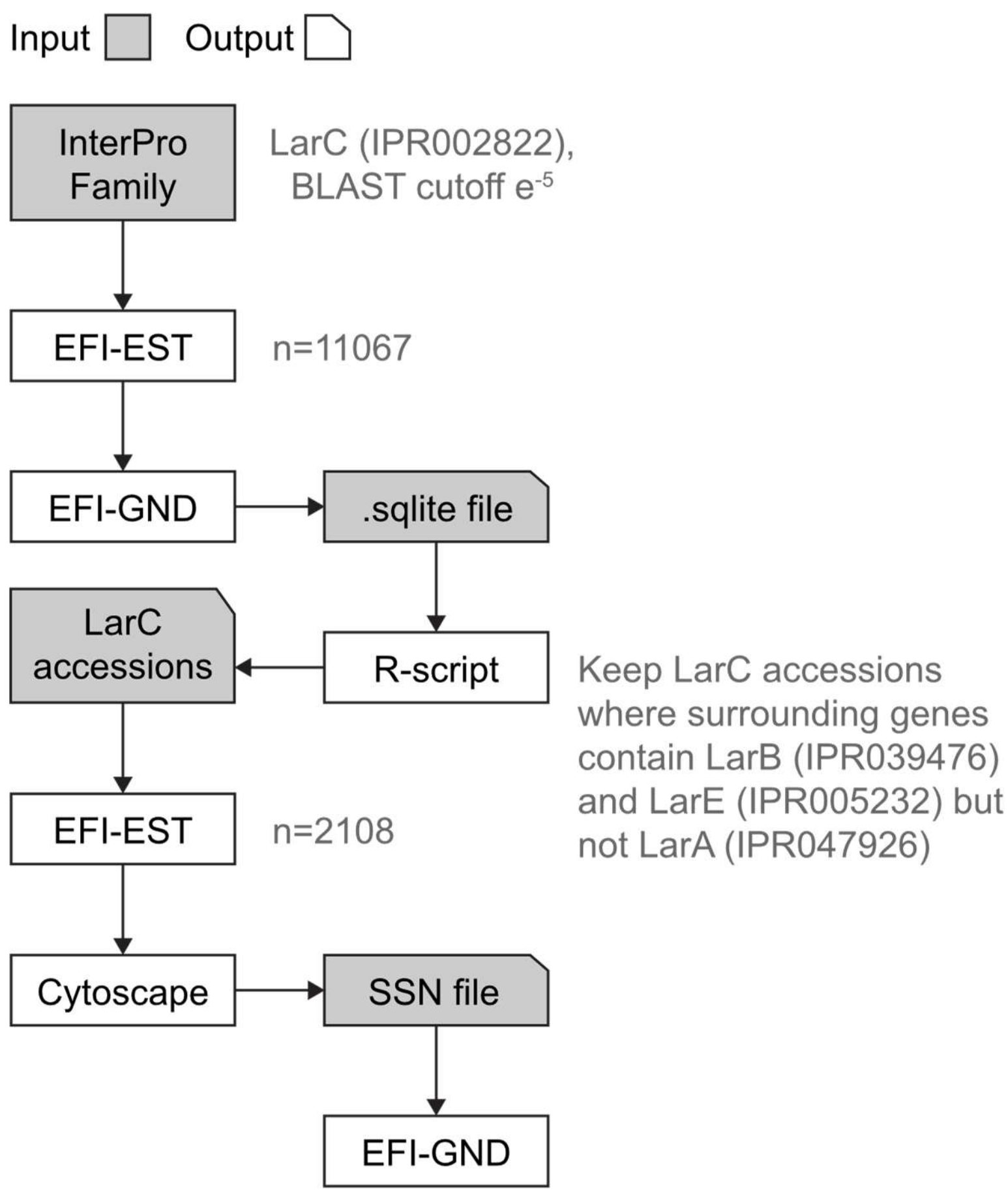

Supplementary Figure 2. Genes that co-occur with the NPMN biosynthesis pathway. The bioinformatic workflow described above was used to generate a LarC sequence similarity network with genomes containing LarB and LarE but lacking LarA within 20 open reading frames (**Supplementary Figure 1**). The output file from the EFI-GND web tool was read into an R script to profile PFAM co-occurrences with the NPMN biosynthesis genes (**Supplementary Code 2**). PF00248 (aldo-keto reductase superfamily) is highlighted in red.

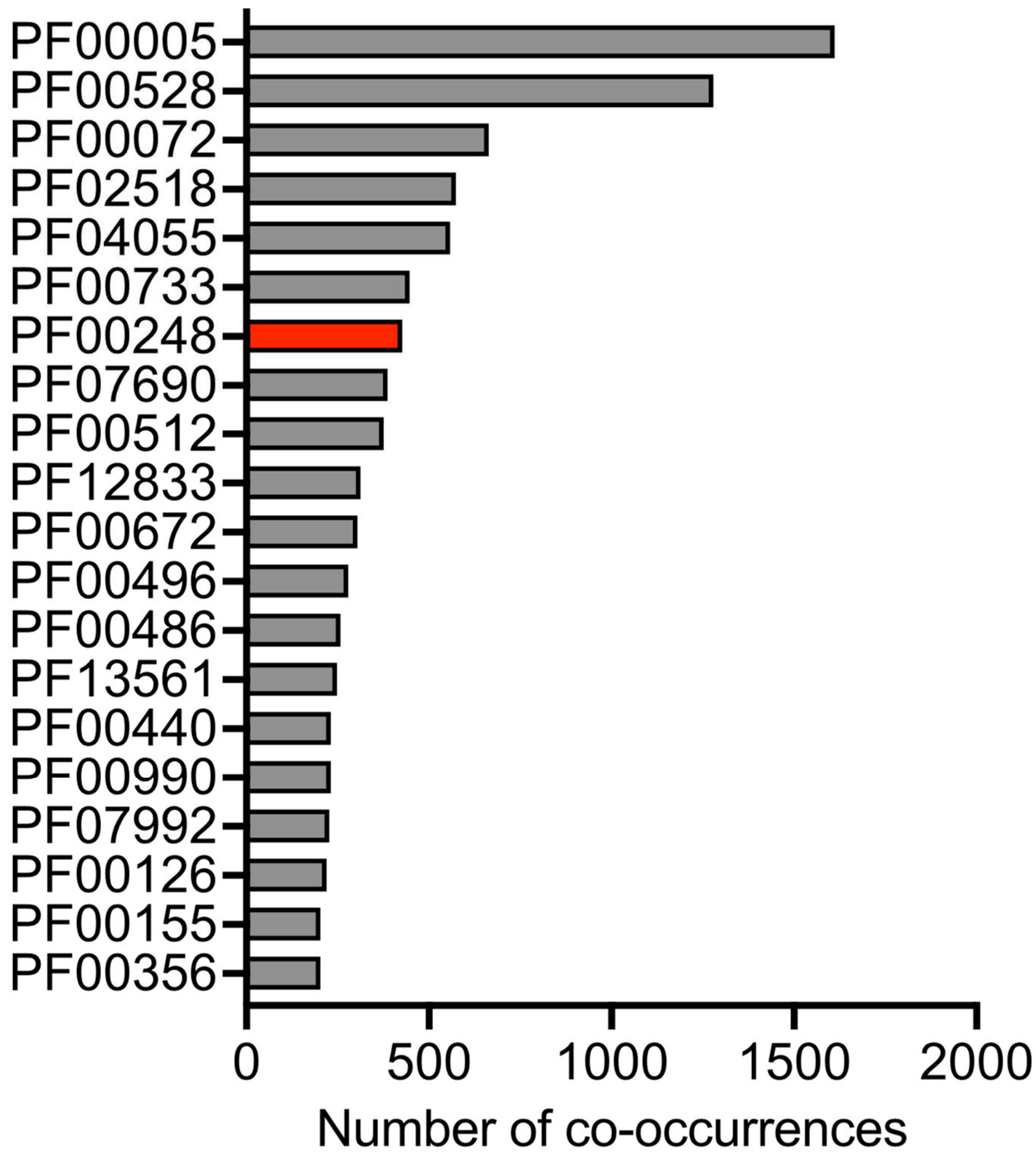

Supplementary Figure 3. Redox reactions catalyzed by AKRs. (A) Canonical AKRs catalyze NAD(P)(H)-dependent redox reactions. (B) The nickel pincer nucleotide (NPMN) cofactor is structurally related to NAD^+. Both cofactors are synthesized from a common precursor (NaAD, **Extended Data Figure 1**). NPMN lacks the adenosine monophosphate (red) moiety found in NAD^+. The nickel in NPMN is coordinated by a histidine residue, and the entire cofactor is covalently tethered to the enzyme active site via a thioamide bond to a lysine residue.

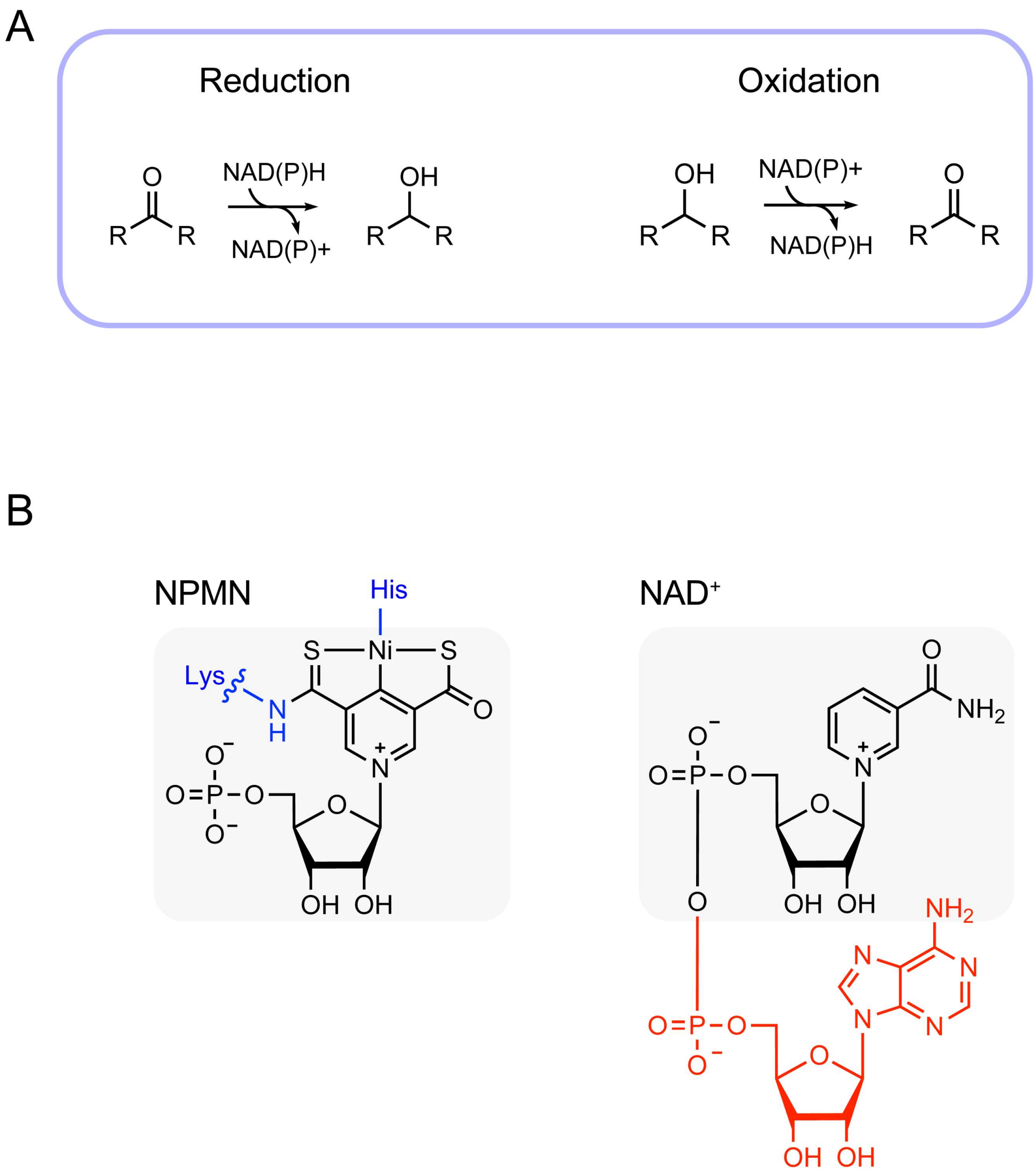

Supplementary Figure 4. Docking NAD(P)$^+$ and NPMN cofactors into AKR homologs and LarA. DiffDock was used to dock the ideal SDF files of NADP$^+$ (PDB NAP), NAD$^+$ (PDB NAD), and NPMN (represented by PDB ID 4EY, which contains the NPMN ligands) from the PDB into a known AKR from *E. coli* (AKR14A1, Chai-1 prediction), AKR11C1 (PDB 1YNQ), LarA (PDB 5HUQ), and NphT (Chai-1 prediction). **(A)** Structure of cofactors used in docking. **(B)** Docking scores.

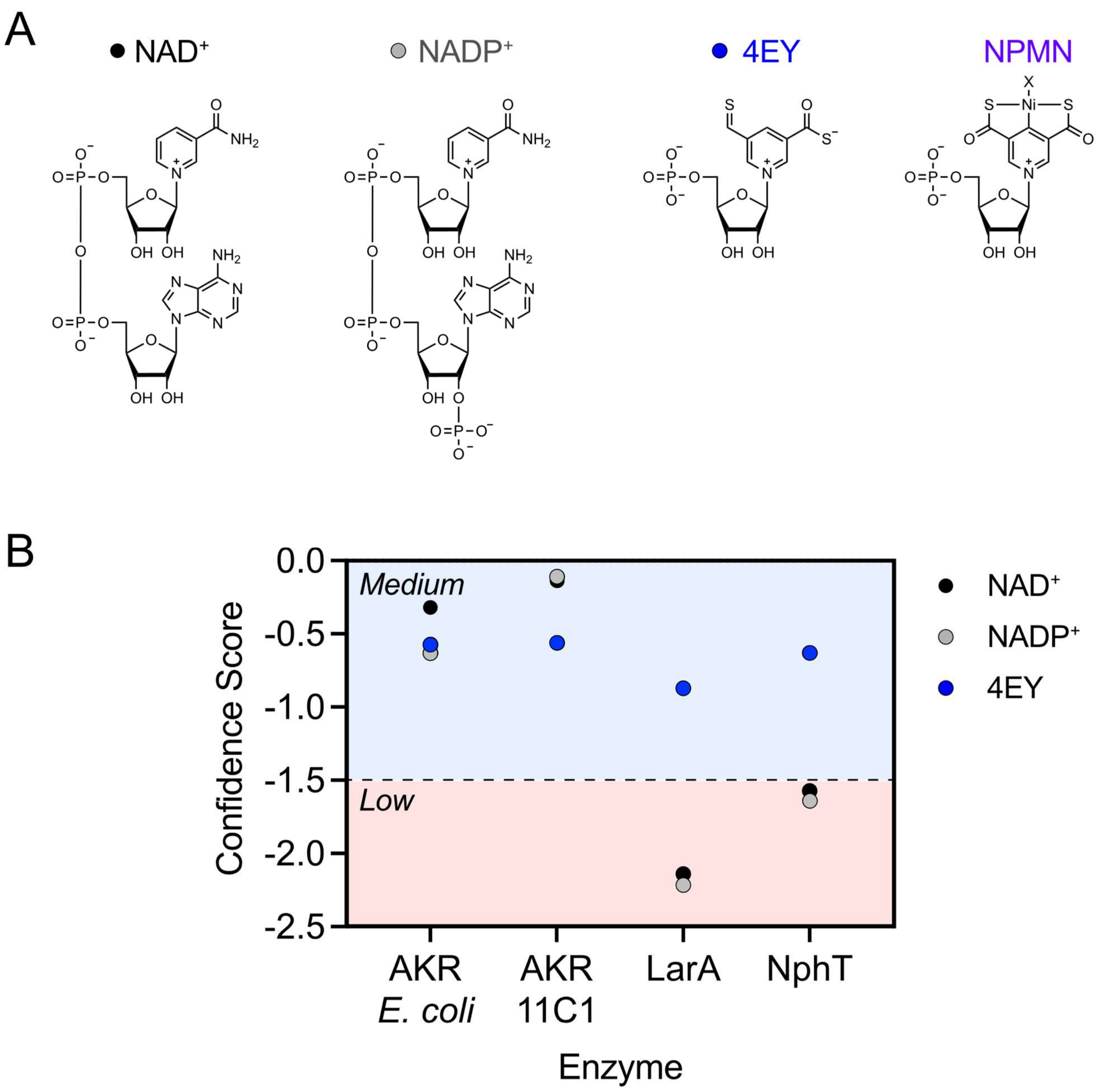

Supplementary Figure 5. Docking NAD(P)$^+$ and NPMN cofactors into AKR homologs and LarA. DiffDock was used to dock the ideal SDF files of NADP$^+$ (NAP), NAD$^+$ (NAD), and NPMN (represented by PDB ID 4EY, which contains the NPMN ligands) from the PDB into a known AKR from *E. coli* (AKR14A1, Chai-1 prediction), AKR11C1 (PDB: 1YNQ), LarA (PDB: 5HUQ), and NphT (Chai-1 prediction). (A) NPMN fits into NphT. (B) NAD$^+$ does not fit into NphT. (C) NPMN in *E. coli* AKR. (D) NAD$^+$ in *E. coli* AKR.

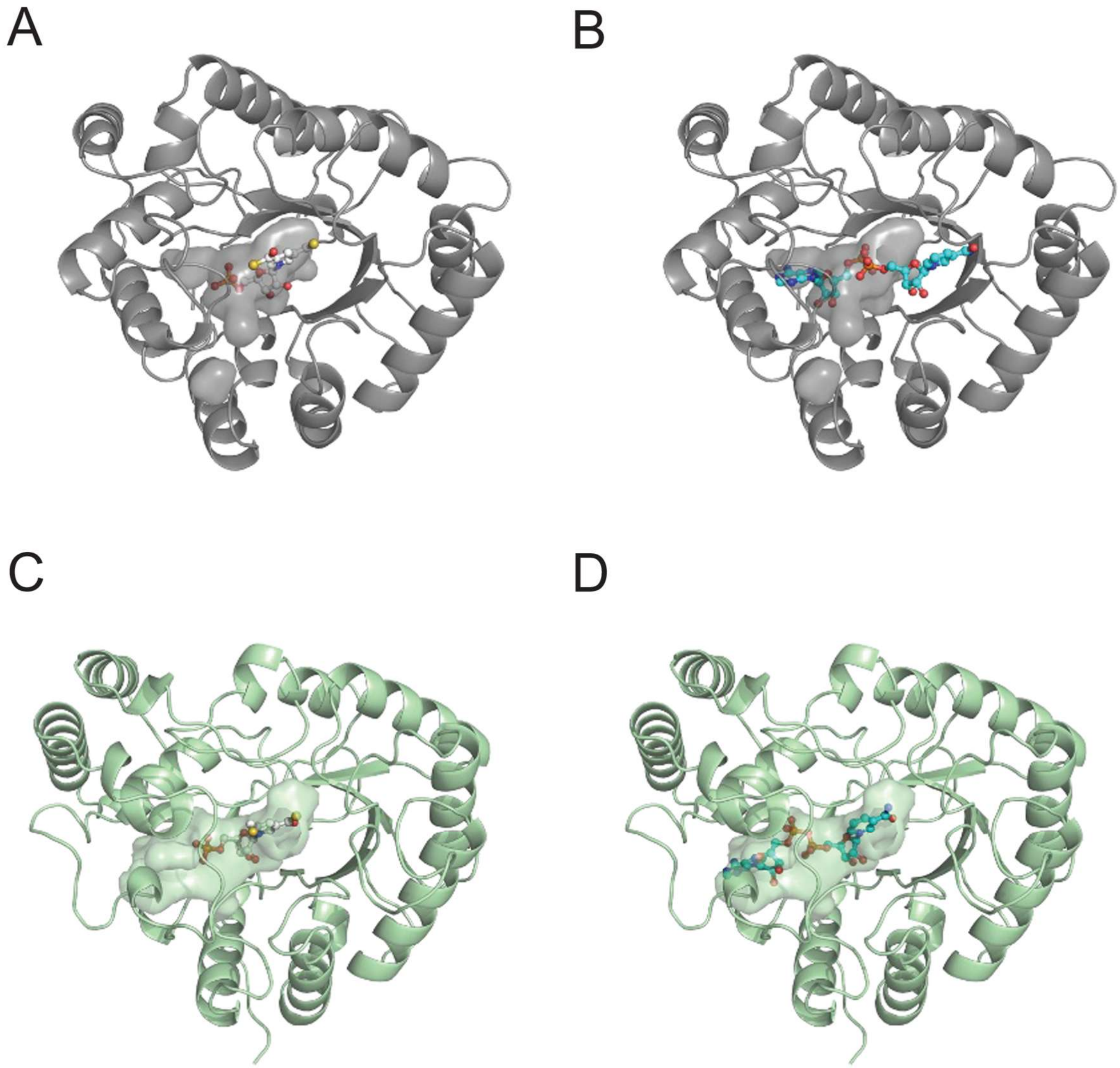

Supplementary Figure 6. Full alignment of AKRs with lysine and histidine highlighted. Expanded version of **Fig. 2d**. The five closest structural homologs of NphT in the PDB were extracted from FoldSeek[2]. AKR homologs located adjacent to the NPMN biosynthetic cluster, but with lower sequence identity to NphT were also examined. An MSA of the sequences was generated using the Clustal Omega in Clustal format. Residues matching H144 and K200 of NphT are highlighted in red, and the aligned position is boxed in grey. Sequence numbering is based on NphT. PDB accession numbers are provided for NAD(P)(H)-dependent structural homologs of NphT.

NAD(P)(H)-dependent AKRs: 7SVQ, 1YNQ, 4AST, 3UYI, 1PZ0

```
                                                                1       10
NphT         -----------------------------------------METRRLGRIGHMSSVIIFG
A0A931AQK1   -------------------------------------MASRVPTRVLGSTGEEVSILGLG
A0A2Z5G7U8   MQRREFLRRAATGIGSAWLAQRVAPAAASFEQTTLPAKFKAHDDVVLGKTGIRTSRLAMG
A0A285GDK3   -----------------------------------------MEKRVLGRTGLEVSLLGFG
7SVQ         -----------------------------------MNTHQKLERRELGNTGLNLSCVGFG
1YNQ         -----------------------------------------MKKRQLGTSDLHVSELGFG
4AST         ---------------MVWLA-------------NPERYGQMQYRYCGKSGLRLPALSLG
3UYI         -----------------------------------------MPRVKLGTQGLEVSKLGFG
1PZ0         -----------------------------------------MKKAKLGKSDLQVFPIGLG
                                                         *  .     : :*

             20                  30        40        50            60
NphT         AASLSDV---------SQEEADASIQFALDSGINHFDTAASYGE----SELRLGPWMP-K
A0A931AQK1   GYHIGLL---------EENDSIGLIREAIDQGITFMDNAWCYHNG--LSEKLMGKALQDG
A0A2Z5G7U8   TGTIGYAGASNQTRLGTDRISRMLIDGYNENGLRFYDTADSYGSHP-----YIAAALKHV
A0A285GDK3   GFHLLEI---------PTEKARSLLNYYLDQGGNYIETAADYGDG--ESERKIGGVIA-Q
7SVQ         ASPLGNV--F---GDVSEEQSIATVIEAFNQGINFFDTSPYYGAT--LSEKVLGKCLKAL
1YNQ         CMSLGTD----------ETKARRIMDEVLELGINYLDTADLYNQG--LNEQFVGKALKGR
4AST         LWHNFGH-------VNALESQRAILRKAFDLGITHFDLANNYGPPPGSAEENFGRLLRED
3UYI         CMGLSGD-YN---DALPEEQGIAVIKEAFNCGITFFDTSDIYGEN-GSNEELLGKALKQL
1PZ0         TNAVGGHNLY---PNLNEETGKELVREAIRNGVTMLDTAYIYGIG--RSEELIGEVLREF
                                       :      *    : :   *            ..  :

                    70        80                    90        100      110
NphT         ---IRNKIFLATKTGERTRDE------------AMRSIERSLKRLQVDSVDLMQLHAVTS
A0A931AQK1   ---YREKAFLMTKNHGRTVES------------FNKQLEDSLKRLQTDYIDLLQFHEIIN
A0A2Z5G7U8   ---PRDKVTVLTKTDTRDAAG------------VRDDLDRFRRELGTDYIDVVLIHCVT-
A0A285GDK3   ---RRDELVLATKTSSRDKAG------------AKADIERSMKNLQTDYLDLIFMHAVGT
7SVQ         -GASRDEYIVATKCG-RYAEG-----FDFSAERVTKSIDESLERLQLEYVDILQCHDIEF
1YNQ         ---RQ-DIILATKVGNRFEQGKEGWWWDPSKAYIKEAVKDSLRRLQTDYIDLYQLHGGT-
4AST         FAAYRDELIISTKAGYDMWPG--PYGSGGSRKYLLASLDQSLKRMGLEYVDIFYSHRVD-
3UYI         ---PREKIQVGTKFG-IHEIGFSGVKAKGTPDYVRSCCEASLKRLDVDYIDLFYIHRID-
1PZ0         ---NREDVVIATKAA-HRKQGN-DFVFDNSPDFLKKSVDESLKRLNTDYIDLFYIHFPD-
                 : .  : **                      .    ..:  : :*:   *

                 120       130       140      150            160
NphT         FEELDQCTRKGGALEAAIAAKEQGMVKHIGITGHGHLAPKVHLEALRR-----YPFETVL
A0A931AQK1   EGEPAR-IEKEKVLEAALKAKEAGKIRYLGFTGHRD--PHYHQQMLELD----IDWDTFQ
A0A2Z5G7U8   --EDDWTTRYRGAMDVLSEAKQKGVIRAHGVSCHS---IGALRAAAA--SPWVELDLVRL
A0A285GDK3   KEDLDKILSSGGAMEAVRELQEAGKIGYVAISMHGQ--PDTLIEAIRT-----DEFDVVM
7SVQ         GSLD---QIVNETIPALQKIKESGKTRFIGITGLP---LEVYTYVLDRVPPGTIDVVLSY
1YNQ         --ID---DPIDETIEAFEELKQEGVIRYYGISSIR---PNVIKEYLK-----RSNIVSIM
4AST         --EN---TPMEETASALAHAVQSGKALYVGISSYS---PERTQKMVELLREWKIPLLIHQ
3UYI         --TT---VPIEITMGELKKLVEEGKIKYVGLSEAS---PDTIRRAHA-----VHPVTALQ
1PZ0         --EH---TPKDEAVNALNEMKKAGKIRSIGVSNFS---LEQLKEANK-----DGLVDVLQ
                       .        : *       ..:
```

```
                170             180             190            200             210             220
NphT        TPY-NFIL-YNNPEYRQAFDA-LVEEVKRQDVGLMTIKAIARGP-WPSEDAKKYATWYEP
A0A931AQK1  IPV-NILD-Y---HYRSFQNQ-LIPQAEERGIGVIGMKSLAAGRIFNTDLS---------
A0A2Z5G7U8  NPVQARMDAE--PD----TVVSVIKQMRASGKAIVGMKILGQGAMRSRPSEAL-------
A0A285GDK3  ATL-NYFDRFNFPKL---EGE-LIPLAQEKNVGLICMKPIADGFLWRSAKQAFRYSFSLP
7SVQ        CHY-CINDST-------L--EDMLPYFKSKGVGVINASPLSMGLHTENGPP----EW---
1YNQ        MQY-SILDRR-------PE-E-WFPLIQEHGVSVVVRGPVARGLLSRRPL---------P
4AST        PSY-NLLNRW-------VDKSGLLDTLQNNGVGCIAFTPLAQGLLTGKYLN----GI--P
3UYI        IEY-SLWTRD-------IEDE-IVPLCRQLGIGIVPYSPIGRGLFAGKAIK----E-SLP
1PZ0        GEY-NLLNRE-------AEKT-FFPYTKEHNISFIPYFPLVSGLLAGKYTE----DTTFP
                                     .     .    .  .  :      :    *

                                                                 230
NphT        FD--------------------------------------------DQEHIDRCVSFVLSR
A0A931AQK1  ----------------------------------------------VEDAITYCLSLPIST
A0A2Z5G7U8  ---------------------------------------------------HYALGTGVLD
A0A285GDK3  V-----------------------------------------------------SM----
7SVQ        --HPA----------SP--------EIKAACKAAADYCKKNGKNISKLALQYSLSNKDIS
1YNQ        EGEG--------------YLNYRYDELKL-----LRESLPTDRPLHELALQYCLAHDVVA
4AST        QDSRMHREGNKVRGLTPKMLTE---ANLNSLRLLNEMAQQRGQSMAQMALSWLLKDDRVT
3UYI        ENSVL--------TSHPRFVGENLEKNKQIYYRIEALSQKHGCTPVQLALAWVLHQGEDV
1PZ0        EGDLR--------NEQEHFKGERFKENIRKVNKLAPIAEKHNVDIPHIVLAWYLARPEID

               240        250         260          270         280
NphT        PEITGLASAGDVRLLPKIVEAYKRYRKLSGEEQEALMATA--GSYASPFGPF--------
A0A931AQK1  -LVSGIDSFQVLEQNLKIVENFK---PATEEELYSLRTRIEPEAYKGQH-----------
A0A2Z5G7U8  AFTIGAESRAEQDDLVRRIAAA--------------------------------------
A0A285GDK3  -VVTGMNSEEMVKLDIDFA---NNFVAMTKDEKEKLFKDA------SELGDYICRQCGSC
7SVQ        TTLVGMNSVKQVEENVGAALEL----ETAGKDEKTFAEI--------EN-----------
1YNQ        TVAAGASSIDQVKANVQAVEAT----PLTAEERQHIQKLAKAAVYEQ-------------
4AST        SVLIGASRAEQLEENVQALNNL----TFSTKELAQIDQHIADGELN--------------
3UYI        VPIPGTTKIKNLHNNVGALKVK-----LTKEDLKEISDAVPLDEVAGES-----------
1PZ0        ILIPGAKRADQLIDNIKTADVT-----LSQEDISFIDKLFA-------------------
                *

NphT        ------------------------------------------------------------
A0A931AQK1  -----------------EYYKH--------------------------------------
A0A2Z5G7U8  ------------------------------------------------------------
A0A285GDK3  DDVCPEGINIREIFKLEGYYDRQMRDGKPRNPADFALRDRLRFWF-GNQEMARDLYKKLE
7SVQ        ---------ILKPIKNQ-------------------------SWPSGIQQT---------
1YNQ        ----------HRE-----------------------------------------------
4AST        ------------------------------------------LWQASSDK----------
3UYI        ---------IHEVIAVT-------------------------NWKFANTPPLK-------
1PZ0        ------------------------------------------------------------

NphT        ----------------------------------------
A0A931AQK1  ----------------------------------------
A0A2Z5G7U8  ----------------------------------------
A0A285GDK3  VKANHCTECEKCLDACPYDLPMIDKLKMCHYKLGDDNSLF
7SVQ        ----------------------------------------
1YNQ        ----------------------------------------
4AST        ----------------------------------------
3UYI        ----------------------------------------
1PZ0        ----------------------------------------
```

NAD(P)(H) -dependent AKRs: 7SVQ, 1YNQ, 4AST, 3UYI, 1PZ0

Supplementary Figure 7. Sequence alignment of AKRs adjacent to the NPMN biosynthetic cluster. The bioinformatic workflow described in **Supplementary Figure 1** was used to generate a LarC sequence similarity network with genomes containing LarB and LarE but lacking LarA within 20 open frames. The output file from the EFI-GND web tool was read into an R script to profile PFAM co-occurrences with the NPMN biosynthesis genes (**Supplementary Code 2**), where PF00248 returned 427 AKR entries. UniProt ID mapping was used to generate a FASTA file of these entries by UniProt accession number, resulting in 395 unique accessions. An MSA was generated using Clustal Omega in FASTA format, and the character positions for NphT H144 and K200 were used to identify sequences containing the histidine/lysine motif (**Supplementary Code 3**) (A) Table of the distribution of AKRs with the histidine/lysine motif. Global accession counts (427) differ from unique accession counts (395) because some genomes contained multiple *larC* genes, resulting in duplicate neighbors. (B) Sequence alignment highlighting the histidine/lysine motif. Residues matching H144 and K200 of NphT are highlighted in red, and the aligned position is boxed in grey. Sequence numbering is based on NphT.

A

Motif	Accession Counts (Unique)	Percentage (Unique)	Accession Counts (Global)	Percentage (Global)
Histidine/Lysine	135	34.20%	145	34.00%
Histidine only	159	40.30%	173	40.50%
Lysine only	0	0.00%	0	0.00%
Other residues	101	25.50%	109	25.50%

B

```
             139       149    195       204                 139       149    195       204
NphT        IGITGHGHLAP·····GLMTIKAIA--R      A0A520PSN7  IGVTGHSWSVA·····AVQTIKSIA--R
A0A355Q2M5  -----------·····------------      A0A968ECY3  IGVTGHGTRVA·····AVQTIKSVA--R
A0A1H8CFR3  -----------·····------------      A0A2E2ULT1  IGVTGHGLTVA·····AIQTIKTLL--R
A0A161WFJ8  -----------·····------------      A0A3C1TW85  IGVTGHGTYAA·····GLQTIKSIA--H
A0A1H8CF24  IGITGHGHIAP·····GLMVIKAVA--K      A0A2E7XF39  IGVTGHGTRAA·····AMQTIKSIA--R
R5QQG8      FEISFHDNADV·····PVIVMEPVK--G      A0A845D2M8  IGVTGHGLKIA·····AVQVIKSLL--R
A0A957T4T2  IGVTGHDVAVA·····------------      A0A2E5M5S5  IGXTGHGTQVA·····AVQTIKSVA--R
A0A2S3QH11  IGITGHGHQAP·····GFCTIKAVA--K      A0A6N8WAX9  IGVTGHGLKIA·····SVQVIKSLL--R
A0A3S6Z483  IGITGHGHQAP·····GFCTIKAVA--K      A0A2E8J7G1  IGVTGHGTRIA·····AMQTIKSIV--R
A0A7T5ESJ1  IGITGHGHQAP·····ALRVIKAVA--K      A0A6L7MSK1  IGVTGHGTYIA·····AIQTIKGIA--H
A0A523G1R0  IGFTGHTDFKA·····------------      M1MPZ2      IGITNHRLAVA·····GYICMKALA--G
A0A916ZEU8  IGITGHGHQAP·····AVRVIKAVA--K      A0A2E8KBE9  IGVTGHGTRIA·····AMQTIKSIV--R
A0A5E4P1W0  IGFTGHKDPRI·····SSCRI---S--S      A0A173V5M0  IGVTAHKIGIA·····GFIAMKGLA--G
A0A3D0KQ50  FGFSYHAGPEL·····SITVMEPVK--G      A0A533TG00  VGVSIYNPLEL·····EIHTRSAFL--Q
A0A355Q274  IGVTGHSWSVA·····AVQTIKSIA--R      A0A285GDK3  VAISMHGQPDT·····GLICMKPIA--D
A0A933PEP3  IGITGHGDQAA·····GLMTIKTVS--R      A0A971GXP4  LGFSAHSDEAA·····GRLALKALA--K
A0A2N3PWL1  -----------·····------------      A0A9D1WUQ1  IGVTAHKLGVA·····GFIAMKGLA--G
A0A2E0NDB3  AGFTGHTDPKV·····AVLGMKGFG--G      A0A916Q7I5  IGVTAHKLNVA·····GFIAMKGLA--G
A0A540VJS1  IGVTGHGLHAP·····AVQTIKSIT--R      A0A6L7YHP2  IGISSHSLEVL·····GVIVMRPLG--G
A0A917S355  IGITGHGTTVA·····AFQTIKAIT--R      A0A971GXT8  IGVTGHDWSVL·····GVTLMSVAR--S
A0A1H2A8E5  IGITGHGVTVA·····AVQTIKAIT--K      A0A7W7ZGN3  IGFTGHKDPQI·····GVLAMKTFG--D
A0A1C5LIU7  FEISFHDNADV·····PVIVMEPVK--G      A0A4Q7Z1P2  IGFTGHKDPAV·····GVLAMKTFG--D
A0A516Q0H1  IGITGHGVTVA·····AVQTIKAIT--R      A0A9D2PH95  IGVTAHKLNVA·····GFIAMKGLA--G
```

	139 149		195 204
A0A928K2I9	IGITNHRLAVA	·····	GFIAMKALS--G
A0A2Z5G8J1	IGFTGHKDPLV	·····	GVLAMKTFG--D
A0A497DTQ7	LGFSAHSVEAA	·····	GILALKAMA--W
A0A5R9JCU6	IGFTGHKSPKI	·····	GIIGMKAFG--D
A0A3C0U084	IGITAHKIGVA	·····	GFIAMKGLA--G
A0A2U8VR51	IGFTGHKDPRI	·····	APLAMKTFG--D
A0A0L8V375	IGFTGHQNPFA	·····	GVLAMKTLA--D
A0A0Q5CY14	IGFTGHKDPRI	·····	AALGMKTFG--D
A0A0Q4ZV57	IGFTGHKDPRI	·····	AALGMKTFG--D
A0A7L5A6M2	IGFTGHKDPAV	·····	GVLAMKTFG--D
A0A0S9RTS1	IGFTGHKDPRI	·····	AALGMKTFG--D
A0A2S4LDQ3	IGFTGHKDPRI	·····	AALGMKTFG--D
A0A6I7NMU2	IGFSAHTVEAA	·····	GILALKSMV--R
A0A931AQK1	LGFTGHRDPHY	·····	GVIGMKSLA--A
A0A9E3G229	IGFTGHKDPAV	·····	GVLGMKSMG--D
A0A509EFY8	IGFTGHKDPRI	·····	APLAMKTFG--D
A0A6B9YPB2	IGFTGHKDPAV	·····	GVLAMKTFG--G
A0A7G8BR18	IGFTGHKSPEI	·····	GILGMKPMG--D
A0A7Y8SPT1	TGMSGHGGNLI	·····	GVIAMKTLR--G
A0A926E8X6	IGITNHRLHVA	·····	GFIAMKALS--G
A0A0S9R3D8	IGFTGHKDPRI	·····	AALGMKTFG--D
A0A971GV72	FGVSANDWDPF	·····	GIIVRVPFE--E
A0A2Z5G7E1	VGFTGHKNPSI	·····	AVFSMKSMS--G
A0A968BLS1	TGISGHGGKLN	·····	GVIAMKTLV--G
A0A4Z0NR40	IGFTGHKDPRI	·····	AALGMKTFG--D
A0A4R1LFU6	IGFTGHKSPEI	·····	GILGMKPMG--D
A0A6M1LW93	IGFTGHKDPRI	·····	AALAMKTFG--D
E8LIQ5	IGITSFSNERL	·····	AHEAWAPFA--E
A0A516Q5U2	FGVSNHTPGQI	·····	TLQAWSPFQ--K
A0A3D3XUG1	VGMSGHAGYLV	·····	GTVVMKTLQ--G
A0A075KDF9	IGVSNFYPDRL	·····	QIQAWAPFA--E
A0A951NMP7	IGFTGHKSPEI	·····	GILGMKPMG--D
A0A2U8WW46	IGFTGHKDPRI	·····	AALAMKTFG--D
A0A1B2I986	LGLSNFNPHHI	·····	TAECWSPLR--Q
A0A6S6M741	IGISNFHPDRV	·····	QIESWGPFA--E
A0A0M9UDR8	VGASNYPAWRL	·····	GVIPYSPLA--A
A0A0J6SQ61	IGFTGHKDPRI	·····	AALAMKTFG--D
A0A3N6MVT3	LGFAAHYQPDP	·····	GIIGMKIAA--R
A0A6I7NXZ7	IGISSHS-TEV	·····	GIIAMKLTG--R
A0A959JY82	LGVSTHNDPAG	·····	GVIGMKVAM--A
A0A5R9J563	IGFTGHKDPHI	·····	AVITMKPFGGSN
A0A2Z5G7C9	VGFTGHKDPDV	·····	AALGMKPIG--G
A0A6B0YHG7	AGMSGHGGYLI	·····	GVTVMKTQH--G
A0A9D5UME0	LAVSSHGPNNL	·····	GVIAMKTLA--G
A0A1I4P3C8	IGFTGHKDPRI	·····	APLAMKTFG--D
A0A2E7LJ50	LGVSSHENAQN	·····	ALVGMKAGRLLA
A0A9E3QMW2	IGFTGHKSPEI	·····	GILAMKPMG--D
A0A1I3TMY5	IGFTGHKDPRI	·····	APLAMKTFG--D
A0A7V7U153	IGFTGHKDPRI	·····	AVLGMKTFG--D
A0A2E6LNA6	TGLSGHAGHLI	·····	GVVTMKTLM--G
A0A7Y8MA76	WGVSTHENAEK	·····	GLIGMKAARHLA
A0A1M7PFH9	LGASSMWAWQF	·····	GVIPWSPLA--R
A0A2Z5G7U8	HGVSCHSIGAL	·····	AIVGMKILG--Q
A0A0L6J200	IGFTGHKDPRI	·····	APFAMKTFG--D
A0A2N2NRK9	YGVSVEKVEEA	·····	GVIVRVPLA--S
A0A2E7LHQ8	VGMSGHAGYLV	·····	GTVVMKTLH--G
A0A2S3QH05	YGVSVETVDEG	·····	GILARVPLA--S
A0A2E0ND90	IGTSNMTLPKL	·····	QPIGYCPLG--S
A0A3S6Z2N7	YGVSVETVDEG	·····	GILARVPLA--S
U5W2M8	LGVSMVTSEQL	·····	AFVPFFPLG--G
A0A8T6I303	SGMSGHAGHLI	·····	GVTVMKTQH--G
A0A356K8D8	VGMSGHAGYLV	·····	GTVVMKTLQ--G
A0A7Y1VXM3	IGFTGHSDFHA	·····	GILAMKSLS--N
A0A8T6EF98	SGMSGHAGRLN	·····	GTITMKTLS--G

	139 149		195 204
A0A2A5VXB9	IGTSNMTLPKL	·····	QPIGYCPLG--S
A0A7T0D0U5	FGVSNHNAMQI	·····	TLQAWSPLA--G
A0A2S7F5X5	IGVDNFKQDRL	·····	QMEAWSPFA--A
A0A7C6FHV1	LGISNFHKKHI	·····	ILEAYSPFG--T
A0A1C4T8W9	LGVSNVGIEQV	·····	AYVPFYPLG--G
A0A7K2HHX9	LGVSNVGIEQV	·····	AYVPFYPLG--G
A0A3D8PZW3	IGVSNFHPDRL	·····	QIESWGPFA--E
A0A937FI62	FGVSNHNPMQI	·····	TIQAWSPFQ--Y
A0A6L7MUX6	AGMSGHAGYLL	·····	GVIAMKTLM--G
A0A6L7MSJ1	FGVSTHENADA	·····	GLVGMKAGRLLA
A0A6L7VPU6	SGVSGHAGYLD	·····	GVTVMKTLR--G
A0A9E4BDS4	LGLSTHENAQQ	·····	GLVGMKVGRLLA
A0A6N9B318	AGMSGHAGRLA	·····	GTVVMKTLM--G
A0A6L7VVT6	SGMSGHAGRLN	·····	GTIAMKTLS--G
A0A9E4E8Q0	SGVSGHAGYLD	·····	GVTVMKTLR--G
A0A6L8A945	SGMSGHAGRLK	·····	GTIAMKTLS--G
A0A162U891	WGVSNFDTEDM	·····	PLMAYCPIA--K
J3EXT1	IGASSMYAWQF	·····	GVIPWSPLA--R
A0A350AXG2	TGFTGHTDPQI	·····	AILGMKGFG--G
A0A959JWS5	IGFTGHANFRA	·····	GILAMKTLS--N
A0A6I6JIY0	WGLSEPGVATV	·····	GLVPFSPLG--K
A0A940TTW1	IGCSNYPAWRL	·····	GVIPYSPLA--A
A0A7Y1W060	LGLSTHNDPAG	·····	GIIAMKVAM--A
A0A1H8CDW0	VGVSNYNVPLL	·····	GVVAYSPLT--S
A0A496AD42	FGFSTHDRAEV	·····	GLIGMKAARILA
A0A917K5T5	YGVSVETVAEG	·····	GILVRLPLA--S
A0A962KUM2	TGMSGHAGRLL	·····	GVVAMKTLM--G
A0A6L7TGF4	TGMSGHAGRLN	·····	GTIAMKTLS--G
A0A927BT76	IGYSGDSTDAL	·····	GIIVKRPVA--N
R5TXN8/	YGVSNFDPWKF	·····	HPMIWSPLA--G
A0A413X049	YGVSNFDPWKF	·····	HPMIWSPLA--G
A0A174PM28	YGVSNFDPWKF	·····	HPMIWSPLA--G
R5KRL7	IGVCNFYPDRL	·····	LPEAWGPFA--E
A0A3S5JVE0	TGFTGHTDAKV	·····	AVLGMKGFG--G
A0A540VJY7	IGTSTFAAWQV	·····	GIIPWSPLA--G
A0A2A5W4Z2	TGFTGHTDAKV	·····	AVLGMKGFG--G
A0A7X3FKD3	WGVSNFDAADM	·····	PVLAYSPLA--Q
A0A6S6M6B0	FGLSEAGVETI	·····	GFVPFSPLG--K
A0A2E8J8A2	TGMSGHAGRLA	·····	GIVVMKVLR--G
A0A0M8YJ73	IGAGMNQTAML	·····	GVIVGGVFN--S
A0A9E4KMW5	LGLSTHENAQE	·····	GLVAMKTGRLLA
A0A0K0GE03	IGTSNLTPAQL	·····	AFIPFFPLA--A
A0A6N8UN18	FGLSTHDNAQN	·····	ALVGMKVARHLA
A0A845D2Q8	LGSSTYAAWQF	·····	GLIPWSPLA--G
A0A5R9JCS8	TGVSNWAAWQI	·····	GLMVWSPLA--G
A0A387H8I9	LGLSNSDEDTI	·····	GLVAYSPLA--R
A0A352BA23	WGFSFHDDHEF	·····	SIVVMEPVK--G
A0A496ABH7	SGMSGHAGNLT	·····	GVTVMKTLR--G
A0A1H8CF38	PAVSNWAAWQI	·····	GIISYSPLG--G
A0A5M4AK57	IGASNLPPERL	·····	GVITYFSLA--K
A0A2A5VWJ7	AGFTGHTDPKV	·····	AVLGMKGFG--G
A0A7W3SV73	LGITSHYVSV-	·····	GTVPMKIYG--A
A0A2N3PWJ9	FGLSEAGAQTI	·····	GFVPFSPLG--K
A0A512D9H9	AGISNADVAQI	·····	AFLPWSPLG--G
A0A6V8SGB3	FGVSNQNPMQI	·····	TIQAWSPYQ--Y
A0A6L7VQ73	LGFSSHENASA	·····	GLVAMKAGRMLA
A0A1B3N2A1	IGASSMYAWQF	·····	GVLPWSPLA--R
A0A2E8K998	TGMSGHAGRLT	·····	GIVAMKVLR--G
A0A6N8WBT8	LGSSTFAAWQV	·····	GLIPWSPLG--G
A0A919IWG3	VGSSNFAGWHL	·····	GVLPYSPLH--F
A0A0Q5CN73	LGATVWRDSQL	·····	AVMVNVPFG--R
A0A3L7WRN4	IGSSTFAAWQV	·····	ATIPWSPLA--G
A0A6L7TJX6	FGLSTHNRAAE	·····	ALVGMKVARHLA
A0A9D2L6L7	IGISNCFAWQL	·····	AMTPYSSLA--G

	139 149		195 204
A0A368BI36	GGFTGHTEPKI	·····	AVLGMKGFG--G
A0A7T5ESB9	AGVSEWSAAQI	·····	GQIVFSPLA--Q
G2FNF5/	IGVTQVDADTL	·····	GVVAFGAAA--H
A0A972YH26	NGFTGHTTPQV	·····	AVLGMKGFG--G
A0A285GDM4	LVFSTHANGDE	·····	GVVTMNPLG--G
A0A523MA74	VGMSGHAGRLI	·····	GVIAMKTLM--G
A0A0S9R338	LGATVWRDSQL	·····	AVMVNVPFG--R
A0A5C1MJJ5	FGLSEAGVQTI	·····	GFVPFSPLG--K
A0A2S4LDR3	LGATVWRDSQL	·····	AVMVNVPFG--R
A0A1H2A7F5	FGVSNHTPGQI	·····	TLQAWSPFQ--K
A0A117JSF6	IGASSMWTWQF	·····	GVLPWSPLA--R
A0A3A0B0K2	LGTSTFAAWQL	·····	GIIPWSPLA--G
A0A917NI10	VGASNFAGWHL	·····	GVIPWSPLA--G
A0A101AUP9	IGASNLMAWQL	·····	GILPWSPLA--R
A0A7T5ESD1	IGASNFRAWQL	·····	GVIPWSPLR--G
A0A363TC27	IGVSNFSVEQL	·····	GVVVYSPMQ--K
A0A7J4ZMA4	FGLSEAGAQTI	·····	GFVPFSPLG--K
A0A6N8WAY7	LGSSTFAAWQV	·····	GLIPWSPLA--G
A0A075K835	WGLSEAGVETI	·····	GFVPFSPLG--K
A0A1F3EJX5	IGFSTHAPLNV	·····	GVFIISPND--K
A0A1S9C8Y2	IGMSNMTIAKL	·····	LPIAFCPIG--S
A0A420XK54	FGVSINDYQPE	·····	GVIVRVALD--E
A0A3E2WZF1	AGISNYDRKHT	·····	GIICFSPLA--Q
A0A6L8A9B6	FGLSTHNRAAE	·····	ALVGMKVARHLA
A0A845D4C8	LGTSTFAAWQV	·····	GLIPWSPLA--G
A0A6L7VVL5	FGLSTHNRAAE	·····	ALVGMKVARHLA
A0A174HZK1	AGISNYDREHT	·····	GIICFSPLA--Q
R5KAJ0	WGQSQSTEEQI	·····	GFVPFSPLA--S
A0A7S9ND69	ITFSSHLQNKE	·····	GTIAMNPLA--T
A0A971GVZ4	FGASNWTAARM	·····	PVAAYSSQA--A
A0A7Y2II69	WGTSEWTADEI	·····	GLTTWSPLA--G
C0C006	LGFSAHGRYDT	·····	PIWVMEPLR--G
A0A5A9XBB6	FGLSEAGAQTI	·····	GFVPFSPLG--K
A0A7V7TXV0	FGLSEAGVGTI	·····	GLVPFSPLG--R
F0YY19	AGISNYDKEHT	·····	GIICFSPLA--Q
A0A9D2PVC4	IGISNCFAWQL	·····	AMTPYSSLA--S
A0A6I8LJN0	AGLSDVPAWYA	·····	GVTAWSPLA--G
A0A427T5B4	PGLSDVPSWYA	·····	GITAWSPLA--G
A0A1H5RI31	AGLSDVPAWYA	·····	GVTAWSPLA--G
A0A6S6M1A0	FGLSEAGADTI	·····	GFVPWGPLG--T
A0A7Y2DPP5	WGTSEWSADEI	·····	GTTTWSPLA--G
A0A7C6RXM9	FGLSEAGVPSI	·····	GFVPFSPLG--K
A0A3B0CEN3	LGFTGHQNPKT	·····	GVIAMKTMA--G
A0A512D9N5	FGVSNHTPAQI	·····	TLQAWSPFQ--A
A0A0P6XDV4	FGLSEASAQTI	·····	GFVPFSPLG--R
A0A7X9T3B6	IGFSYHDDAEL	·····	PVVVMEPVK--G
A0A1H2MGS3	IGVGANDTAAI	·····	ALVAGGVYA--S
A0A2N5RDZ6	FGLCEASAETI	·····	GFVAYSPLG--K
A0A1S8L134	FGVSNMNTEQI	·····	QIQAYLSLE--N
A0A374AUK4	IGLSSHNPEAA	·····	GITVMKAFG--G
A0A6N3FWI2	IGAANVDINQI	·····	TIQAYSPLE--M
A0A373N9R2	IGAANVDINQI	·····	TIQAYSPLE--M
A0A373NI80	IGAANVDINQI	·····	TIQAYSPLE--M
A0A564TX92	IGAANVDINQI	·····	TIQAYSPLE--M
A0A2G6K8P8	WGTSEWSADEI	·····	GNTIWSPLA--S
B0MEF6	IGLSSHNPEAA	·····	GITVMKAFG--G
D4LZ06	IGAANVDINQI	·····	TIQAYSPLE--M
A0A6B2M386	CGFSCHDKHLI	·····	GLVAMKEMR--P
A0A6N2UTL7	IGMSSHNPIAA	·····	GITVMKAFG--G
A0A3N0I324	VGISFHDKATV	·····	SLLVMEPVK--G
A0A1M6NZS2	LGFSIHDDIEL	·····	PIMIMEPLK--G
A0A316MN31	IGMSSHNPIAA	·····	GITVMKAFG--G
A0A3A9AY39	LGFSAHGDYDV	·····	PVWVMEPVR--G
D5EKK1	VGFSTHGACQA	·····	GVFIISPTD--K
A0A317FWY4	WGFSFHDTPEF	·····	SIVVMEPVK--G
A0A5C1MF38	VGISFHDTPEL	·····	PIIVMEPCK--G
A0A7U9D4V9	IGFSFHDGPEL	·····	PVIVMEPVK--G
A0A7J4ZMT6	VGISFHDTPEL	·····	PIIVMEPCK--G
A0A5A9XFD4	VGISFHDTPEL	·····	PIIVMEPCK--G
A0A2S7F5C0	IGFSFHDGPEL	·····	PVIVMEPVK--G
A0A7I8DY60	FEISFHDNADV	·····	SVIVMEPVK--G
A0A512TT24	IGFSFHDGPEL	·····	PVIVMEPVK--G
C4IBB0	IGFSFHDGPEL	·····	PVIVMEPVK--G
A0A7W0FFJ9	LGFSFHDKLDT	·····	AVVIMEPLR--G
A0A2N2N606	LGFSFHDKLDT	·····	AVVIMEPLR--G
A0A0K9NCD5	LGFSAHGRYDT	·····	PIWVMEPLR--G
A0A6N2VMV1	IGLSSHNPEAA	·····	GITVMKAFG--G
A0A1S8TMF4	MGFSFHDNANL	·····	QVVVMEPIK--G
W1U622	IGFSFNDGPEL	·····	PVIVMEPVK--G
G7M1Y3	MGFSFHDNANL	·····	QVVVMEPIK--G
A0A1H9W6R5	WGFSFHDTPEF	·····	SIVVMEPVK--G
A0A1H1VRF7	IGVSNVDLDQL	·····	AFVPFFPLG--S
A0A7Y3WU55	IGFSMHDSADH	·····	PVIVMEAVK--G
A0A4R4FGT0	LGFSAHGRYDT	·····	PIWVMEPLR--G
A0A424M2Z5	IGAGVNHTGMI	·····	SVVIGAPFA--S
A0A437UTH2	LGFSSHAAPAN	·····	PIIVMEPVR--G
A0A2D7FGF6	IGAGVNHTGMI	·····	SVVIGAPFA--S
A0A7G5P2E7	IGFSFHDGPEL	·····	PVIVMEPVK--G
A0A1F1KZ38	IGFSFHDGPEL	·····	PVIVMEPVK--G
A0A0F4W2M0	IGFSFHDGPEL	·····	PVIVMEPVK--G
A0A0F4W1I8	IGFSFHDGPEL	·····	PVIVMEPVK--G
A0A7Y3NAC5	MGFSFHDKADL	·····	PMIAMEPVK--G
A0A1Y4B7N9	IGFSFHSTPEE	·····	PVIIMEPVK--G
A0A2G3DUN9	WGFSFHSTPER	·····	SIVVMEPIK--G
A0A6H2D030	IGFSFHGANED	·····	AVIIMEPLR--G
A0A1H9FHB0	WGFSFHDTPEF	·····	SIVVMEPVK--G
A0A1Y4V8T9	IGFSFHSTPQE	·····	PVIIMEPVK--G
R6PDL6	IGFSYHDNAEL	·····	PIVVMEPIK--G
S7TRU6	AGFSFHGLLPD	·····	GVVIMEPLR--G
A0A259UG94	VGMSFHDTPEL	·····	PIFVMEPCK--G
A0A075K844	AGFSFHEQGGL	·····	GVAIMEPLR--G
A0A161WU95	LGISTHNTDIA	·····	ALTVMKGYA--A
A0A544QY00	YGVSIEKVEEG	·····	GIICRVPLA--S
A0A1C6CYC4	LGFSFHDRPEL	·····	PVIVMEPVR--G
R5KS22	LGFSFHGDPAM	·····	PVIVMEPLR--G
A0A1S8NCT8	AGFSFHDELDV	·····	GVVIMEPLR--G
C6BXJ7	AGFSFHGVPED	·····	AVMIMEPLR--G
A0A1I6IMY7	WGFSFHSTPER	·····	SIVVMEPIK--G
A0A285GD92	AGFSYHGVAED	·····	AVIVMEPLR--G
A0A3A9F469	IGISNMTIPKL	·····	QPVGFMPLG--S
A0A3D4N7L7	LGFSFHDKLET	·····	AVVIMEPLR--G
A0A1Y4F0G8	LGVSSHNPEAA	·····	GITVMKAFG--G
A0A1G5F652	WGFSFHSTPER	·····	SIVVMEPIK--G
A0A2N2MZ81	LGFSFHDKLET	·····	AVVIMEPLR--G
A0A6M0LKW2	WGFSFHSTPER	·····	SIVVMEPIK--G
K6GVM8	AGFSFHGEGKD	·····	GVVIMEPLR--G
A0A2G3E8D0	WGFSFHSTPER	·····	SIVVMEPIK--G
A0A937K5F9	AGFSFHDKLEV	·····	GVAIMEPLR--G
A0A943JLF5	IGFSFHSTPEE	·····	PVIIMEPVK--G
A0A380S2Z1	FGFSYHGTPGL	·····	PIIVMEPCK--G
A0A1S8TGY8	AGFSFHDKLEI	·····	GVVIMEPLR--G
R7F6J3	IGFSFHSTPEE	·····	PVIIMEPVK--G
A0A944A4G6	IGFSYHDNAEL	·····	PVVVMEPIK--G
A0A3N9PC11	PGFSFHDELPV	·····	GIAVMEPLR--G
A0A4P8I8Y8	IGLSSHNPEAA	·····	GITVMKAFG--G
E1K2K6	AGFSFHGWLPD	·····	GVIIMEPLR--G
A0A8J6MCP6	LGFSFHDRPEL	·····	PVVVMEPVR--G
A0A316B985	FGFSYHGTPGL	·····	PIIVMEPCK--G

	139 149 195 204
A0A6H2CZ31	AGFSFHGVTED · · · · · AVMVMEPLR--R
A0A368ZIL9	IGFSGHNNPEA · · · · · GVIGMKVFG--A
A0A267MHM9	AGFSFHDDIDT · · · · · GVAIMEPLR--G
C4XPG7	AGFSFHGEGKD · · · · · GVIIMEPLR--G
A0A2N2IAE3	AGFSFHGLPED · · · · · GVIIMEPLR--G
A0A1M6FBD9	WGFSFHDTPEF · · · · · SIVVMEPVK--G
A0A844K2U5	LGFSFHDRPEL · · · · · PVVVMEPVR--G
A0A9D2E1H6	LGFSFHGDPQM · · · · · PVIVMEPLR--G
A0A1V4ICK5	AGFSFHDKLDV · · · · · GVTIMEPIR--G
A0A1T4K882	YGFSFHDQPEV · · · · · PVVVMEPVK--G
A6NR74	LGFSFHDRPEL · · · · · PVIVMEPVR--G
A0A1E9J882	VGFSFHDTADL · · · · · PVIVMEPVK--G
A0A0P6WXX9	LGFSFHDNLRT · · · · · GVVIMEPLL--G
K6U7S0	AGFSFHDKLEV · · · · · GVVIMEPLR--G
C6JM23	VGFSFHDTADL · · · · · PVIVMEPVK--G
A0A3S4XS87	VGFSFHDTADL · · · · · PVIVMEPVK--G
A0A161WU84	AGFSFHDDLDV · · · · · GVVIMEPLR--G
A0A414TT92	VGFSFHDTADL · · · · · PVIVMEPVK--G
A0A921SSW0	LGFSFHDRPEL · · · · · PVIVMEPVR--G
A0A1S8SJX5	AGFSFHDKLEV · · · · · GVVIMEPLR--G
A0A3R5V9T6	AGFSFHDKLEV · · · · · GITIMEPIR--G
G7M1Y4	AGFSFHDKLDV · · · · · GVVIMEPLR--G
A0A1I0PK17	IGFSYHDSADL · · · · · PVIVMEPVK--G
A0A1S8TMJ2	AGFSFHDKLDV · · · · · GVVIMEPLR--G
A0A6P1ZM48	AGFSFHGLAED · · · · · GVIIMEPLR--G
A0A964RS23	AGFSFHDKLEV · · · · · GVVIMEPLR--G
A0A9D9DLS2	IGFSFHGDPDM · · · · · PIIVMEPLR--G
L1QA15	IGFSYHDSAEL · · · · · PVIVMEPIK--G
A0A6M1PEW5	PGFSFHDELPV · · · · · GIAVMEPLR--G
A0A1G8UXW8	FGFSYHGSPEL · · · · · PIIVMEPVR--G
A6LQW5	AGFSFHDKLEV · · · · · GVVIMEPLR--G
A0A6V8SH00	AGFSFHDKLEV · · · · · GVTIMEPIR--G
A0A7Y9A7F0	AGFSFHDKLEV · · · · · GVVIMEPLR--G
A0A5P2EPJ8	AGFSFHDKLEV · · · · · GVVIMEPLR--G
A0A1S9N691	AGFSFHDKLEV · · · · · GVVIMEPLR--G
A0A0B5Q506	AGFSFHDKLEV · · · · · GVVIMEPLR--G
A0A0K2MJC9	AGFSFHDKLEV · · · · · GVVIMEPLR--G
A0A1S8RBF2	AGFSFHDKLEV · · · · · GVVIMEPLR--G
G7QBP2	AGFSFHGWLPD · · · · · GVVVMEPLR--G
A0A1W7LP96	AGFSFHDKLEV · · · · · GVVIMEPLR--G
A0A1S8PYH1	AGFSFHDKLEV · · · · · GVVIMEPLR--G
A0A089HRS1	PGFSFHDELPV · · · · · GIAVMEPLR--G
A0A4P6HKP2	AGFSFHGEGKD · · · · · GVVIMEPLR--G
A0A0F7F9W0	PGFSFHDELPV · · · · · GIAVMEPLR--G
I2Q6T3	AGFSFHGWLPD · · · · · GVIVMEPLR--G
A0A6V8LXJ9	AGFSYHGLPED · · · · · GVVVMEPLR--G
A0A1B9BIU5	AGFSFHDKLEV · · · · · GVVIMEPLR--G
A0A1S8L0Z1	IGFSYHDNSEL · · · · · PVIVMEPIK--G
A0A0B1TTC9	AGFSFHGLAEE · · · · · GVIIMEPLR--G
A0A075K816	IGFSYHDNADL · · · · · PVIIMEPVK--G
M1WND8	AGFSFHGILPD · · · · · GIIVMEPLR--G
A0A6I6JRP1	PGFSFHGLYED · · · · · GVVVMEPLR--G
A0A6I6JGK5	PGFSFHGLAED · · · · · GVVVMEPLR--G
A0A7Y3SYF3	LAVSSHSPKAC · · · · · GITVMKPYG--G
A0A939BRS6	IGFSFHDDYDL · · · · · PVVVMEPLR--G
A0A1Q8QQ36	AGFSFHGLAED · · · · · GVIVMEPLR--G
A0A1I6Y8M9	WGFSFHDTPEL · · · · · PIVVMEPVK--G
A0A2P1S6C5	VGFSFHDTANL · · · · · PVIVMEPVK--G
A0A2T0BEV1	AGFSFHDKLEV · · · · · GVVIMEPLR--G
A0A516GW31	IGFSGHKNPEA · · · · · GVIGMKVFG--A
R4K5I7	AGFSFHDKLDL · · · · · GITIMEPLR--G
H1PT42	VGFSFHDTANL · · · · · PVIVMEPVK--G
A0A9D1Z3G8	FGFSFHGSYED · · · · · PLIIMEPVK--G
A0A928KLS9	YGFSFHAGPQL · · · · · SVVVMEPVK--G

	139 149 195 204
A0A1C5LIA8	IGFSYHDGPEL · · · · · PVIVMEPVK--G
A0A1Q9JGX8	FGLSLHETPEF · · · · · PIVVMEPCK--G
A0A6L5Y7A4	FGLSLHETPEF · · · · · PIVVMEPCK--G
I0X701	IGISFHDDAAF · · · · · PVIIMEPVK--G
A0A841KY02	VGFSYHDNADL · · · · · PVIVMEPVK--G
A0A3A9AVV7	IGISSHNPVVA · · · · · AIDVMKVFG--G
A0A7G8WDM4	IGFSGHNNPEA · · · · · GVIGMKVFG--A
A0A9E2NU07	LGFSTHNPTIA · · · · · GISVMKPFA--G
A0A7I8DY66	IGFSYHDGPEL · · · · · PVIVMEPVK--G
R5QA53	IGFSYHDGPEL · · · · · PVIVMEPVK--G
A0A1M5YPE5	AGFSFHDKLEV · · · · · GVAIMEPLR--G
A0A7D7A1A4	AGFSFHDKLEV · · · · · GVAIMEPLR--G
A0A1C5QM06	IGFSYHDGPEL · · · · · PVIVMEPVK--G
A0A1C5LLY9	IGFSYHDGPEL · · · · · PVIVMEPVK--G
A0A4P8IE47	IGFSFHDTAEV · · · · · PVIVMEPVK--G
A0A7U9T4N4	VGLSSHNPVIA · · · · · AIDVMKVFG--G
A0A974BIM8	IGFSFHGAQKE · · · · · PVIIMEPLL--G
A0A1M6FB53	IGFSGHKNPEA · · · · · GVIGMKVFG--A
U2N1E7	AGFSFHDKLEV · · · · · GVAIMEPLR--G
A0A9D9MDC3	WGFSFHADPAL · · · · · SIVVMEPVK--G
E8LIR5	LGFSTHTPVIA · · · · · AISAMKPFA--G
R5QKH9	IGFSFHDTPEV · · · · · PVMVMEPLR--G
A0A1B2I931	LGFCSHTLSIC · · · · · GIHVMKVFA--G
A0A0J1IYA7	YGFSFHDTADC · · · · · PVIIMEPVK--G
A0A9D2AIJ3	FGVSLHDSPEF · · · · · PVVIMEGLK--G
A0A3C0U093	WGFSFHDDHEF · · · · · SIVVMEPVK--G
A0A4U8QBB4	YGFSFHDTADC · · · · · PVIIMEPVK--G
A0A1Y4WB59	IGLSSHTPELI · · · · · GISVMKPYS--A
A0A350KNS3	IGFSFHDKADL · · · · · PVVVMEPVK--G

Supplementary Figure 8. *In vitro* synthesis of NPMN for generating holoenzyme. All steps were performed in anaerobic chamber (Coy). **(A)** Workflow for *in vitro* synthesis. See **Extended Data Figure 1** for biosynthetic pathway. Reactions (600 µL) contained NaAD (0.4 mM), sodium bicarbonate (50 mM), and magnesium chloride (0.4 mM) in 100 mM Tris Buffer (pH 7.5). Reactions were initiated by the addition of purified LarB S127A (12 µM final concentration) and allowed to proceed for 20 hours at room temperature to yield P2CMN, and any LarB that precipitated was removed by centrifugation at 15,000 x *g* for 5 minutes. To generate NPMN, reactions (725 µL) contained 196.5 µL of the LarB reaction from step 1, magnesium chloride (2.5 mM), ATP (2.5 mM), βME (5 mM), CTP (0.25 mM), manganese chloride (0.4 mM), nickel chloride (75 µM), LarC (80 µM), and LarE (130 µM). LarC and LarE were omitted as a negative control. **(B)** Reactions containing all components turn pale orange (left), while the negative control does not (right). Reactions proceeded for 3.5 hours at room temperature before boiling at 90°C for 10 minutes to remove LarB, LarC, and LarE. The sample was vortexed for 2 seconds, and the precipitated LarB and LarE were removed by centrifugation at 13,000 x *g* for 10 minutes. The supernatant (650 µL) was transferred to a fresh tube and used as the NPMN source for loading candidate enzymes. This experiment was performed n=4 times at this scale, with similar results.

A

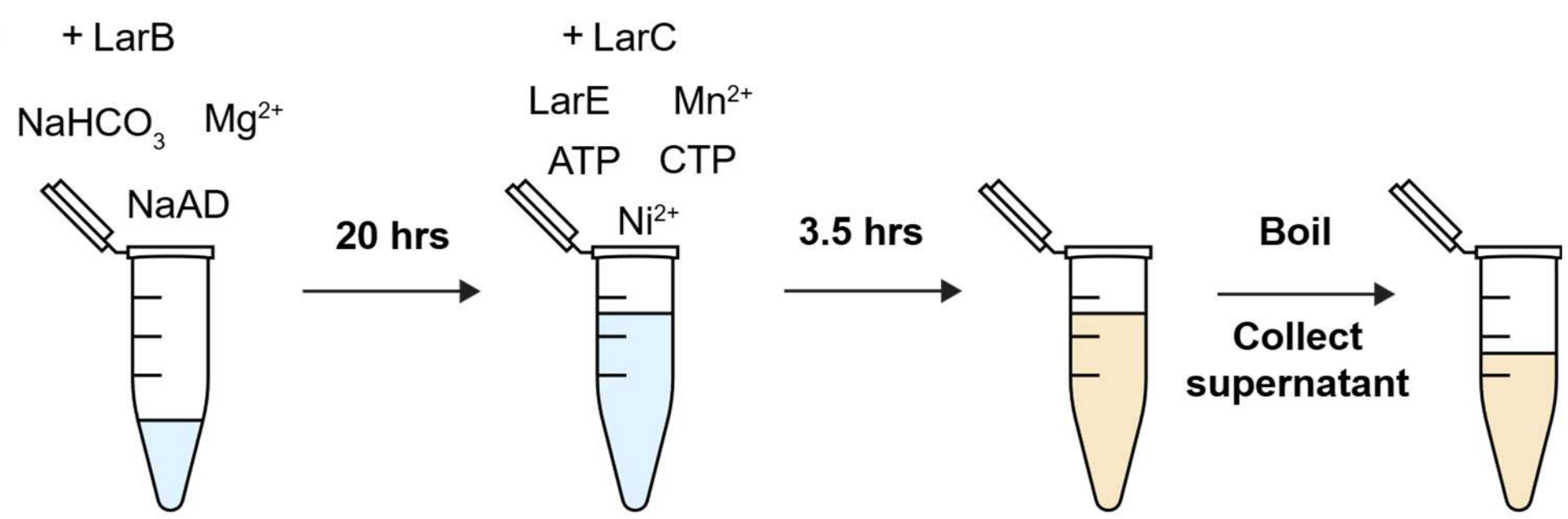

B

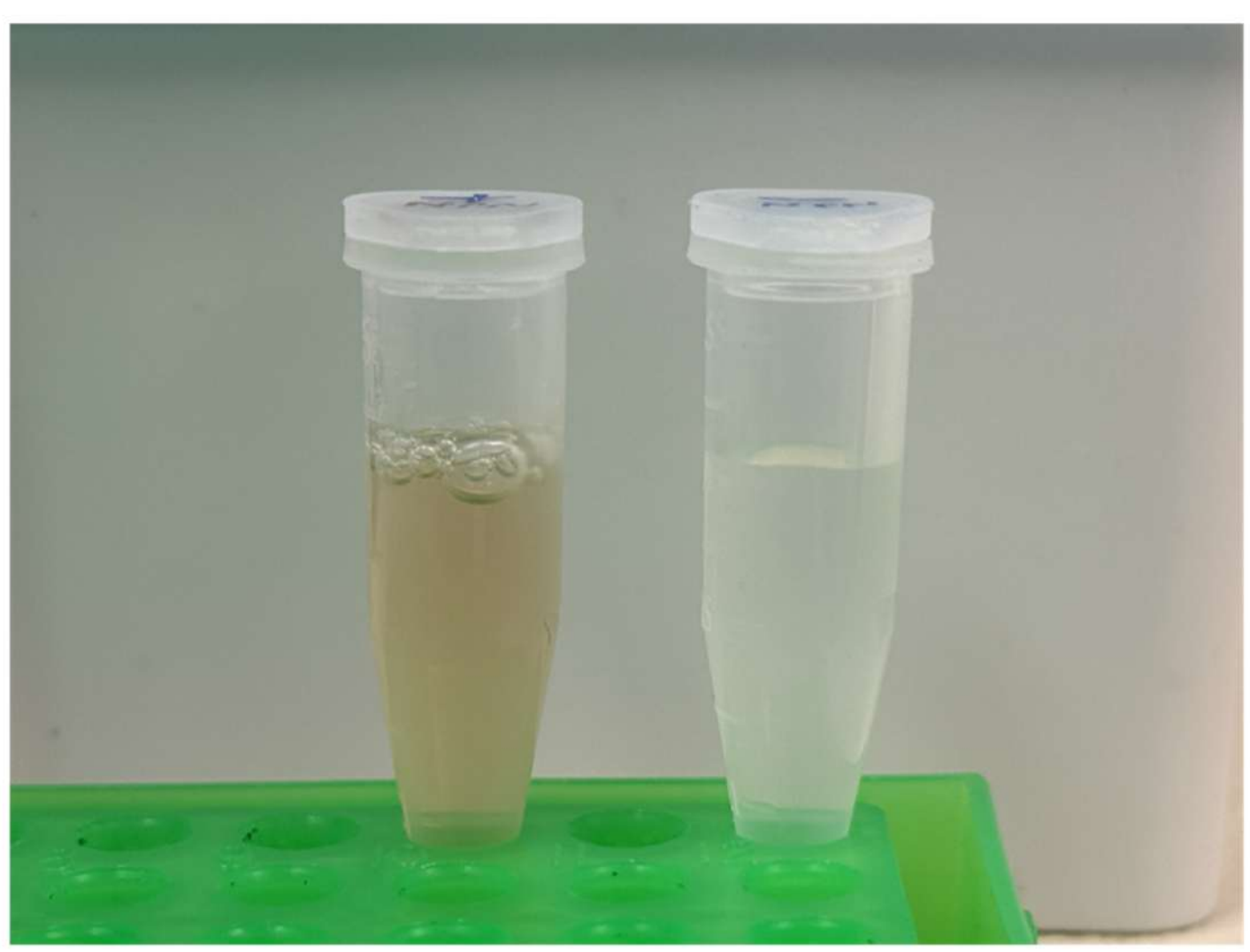

Supplementary Figure 9. Panel of sugars and sugar derivatives used to test the activity of NphT. The following substrates were used to assay for NphT activity by NMR. Substrates were curated based on the genomic context of NphT, commercial availability, common roles in metabolism, and position-dependent substituent effects.

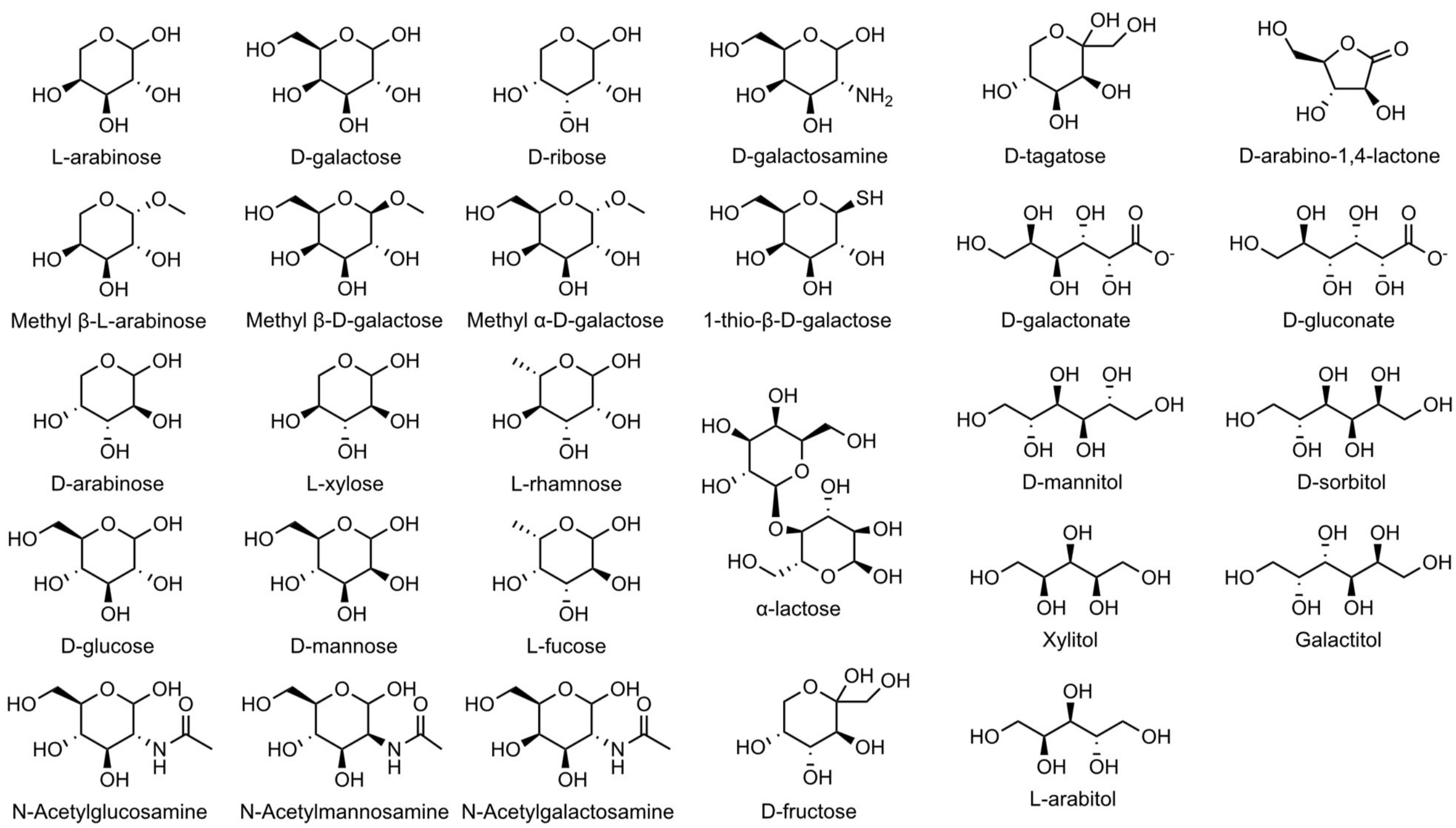

Supplementary Figure 10. NMR reactions to test NphT activity. In a Coy anaerobic chamber, the NPMN cofactor was synthesized *in vitro* as described in the methods and incubated with 40 µM NphT for 20 hours. A portion of the loaded protein was set aside for protein mass spectrometry to verify loading. NphT was exchanged into water using a PD-10 column, and fractions were combined and concentrated to 6.39 mg/mL using a 500 µL Modified PES VWR Centrifugal Filter, 10 kDa MWCO (VWR). Reactions (100 µL) containing holo (+NPMN) or apo (-NPMN) NphT (20 µM) were incubated with a given substrate (45 mM), in 20 mM HEPES pH 7.5 for 20 hours at 50°C. After drying down in a speed vacuum concentrator, the samples were resuspended in a final volume of 600 µL in D_2O before being transferred to a tube for NMR analysis. ^1H and ^{13}C spectra were obtained for each sample. Apo (-NPMN, black) and Holo (+NPMN, red) spectra are overlaid below. Residual water appears at ~4.7 ppm. This experiment was performed once.

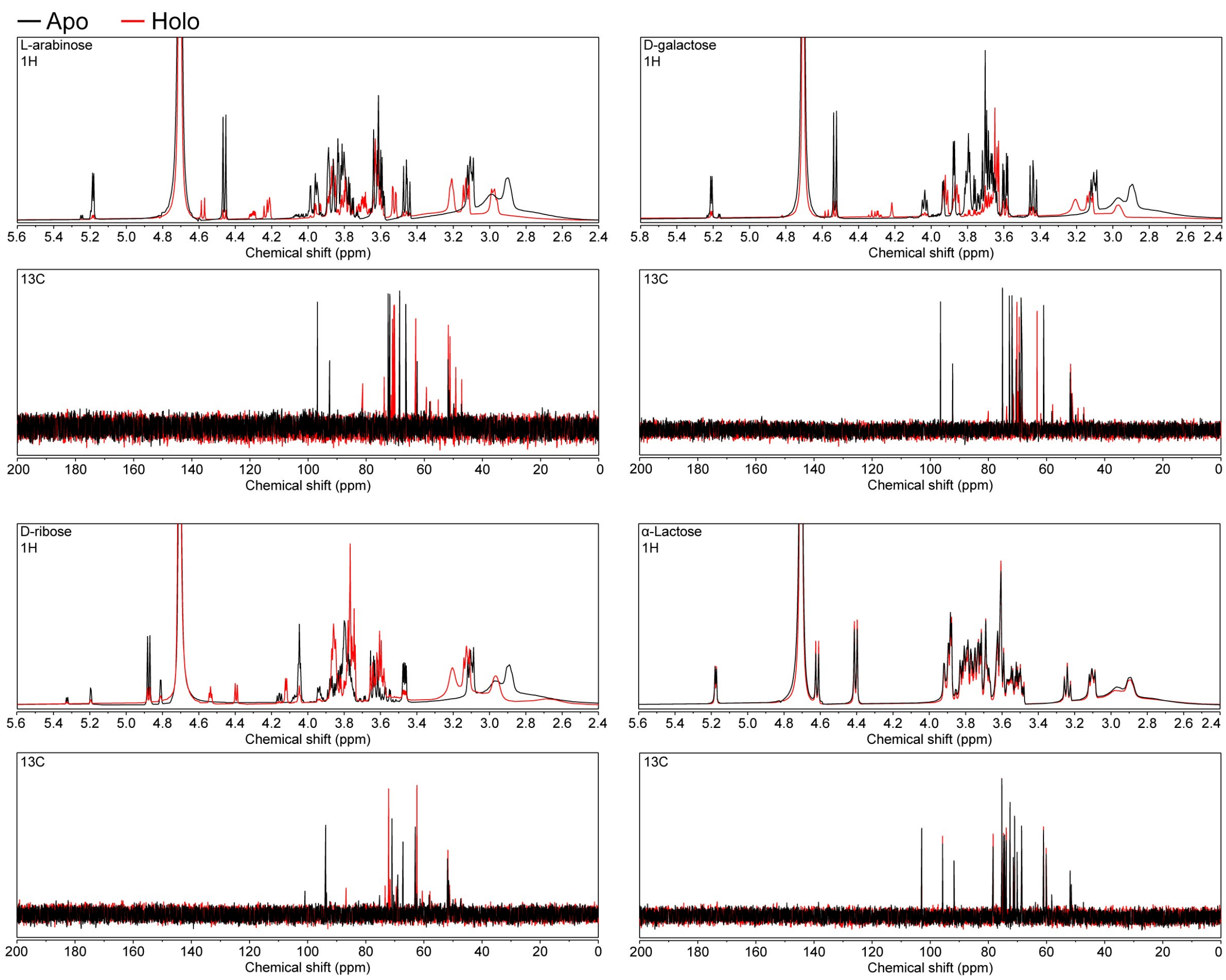

Supplementary Figure 10. NMR reactions to test NphT activity (continued).

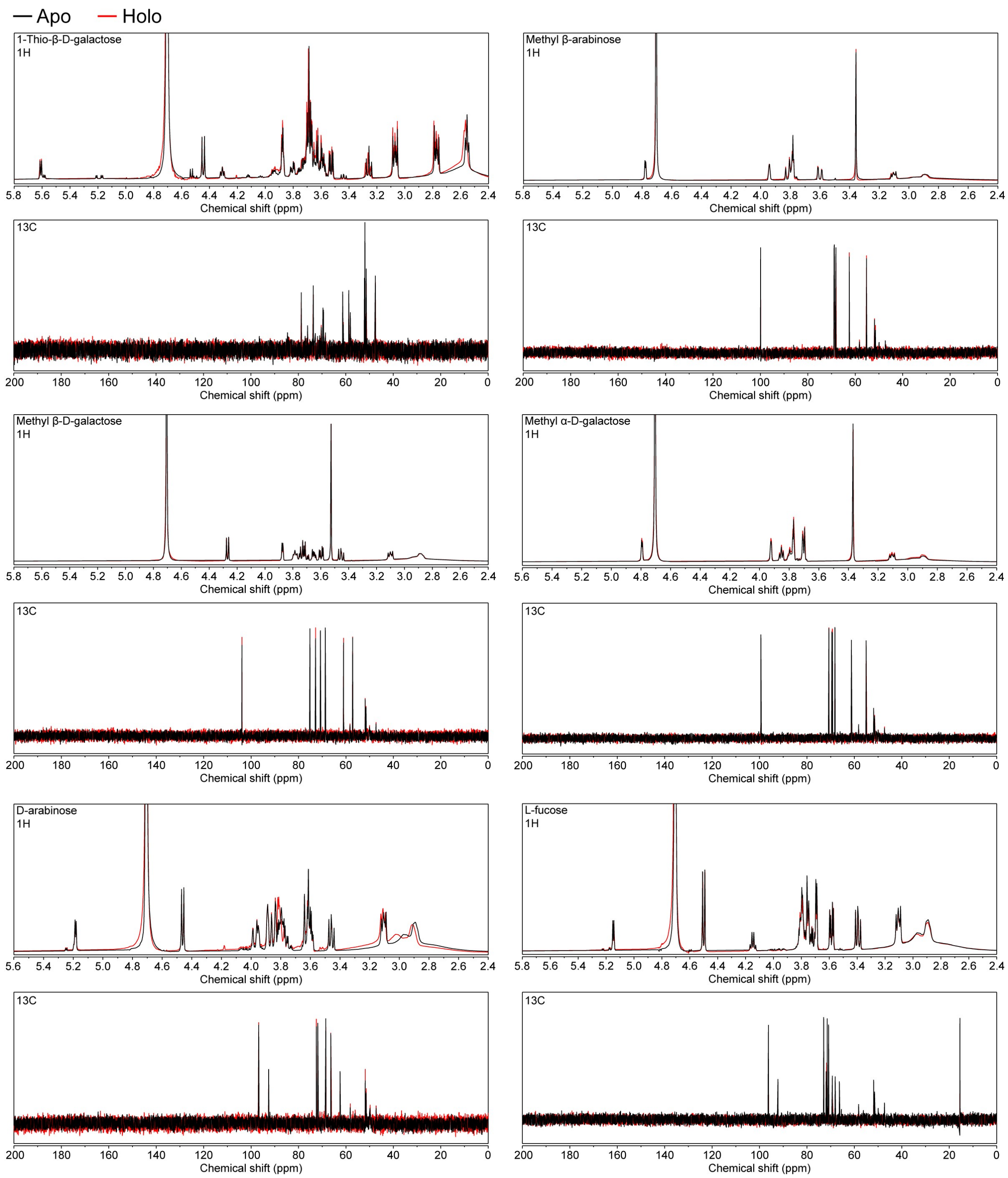

Supplementary Figure 10. NMR reactions to test NphT activity (continued).

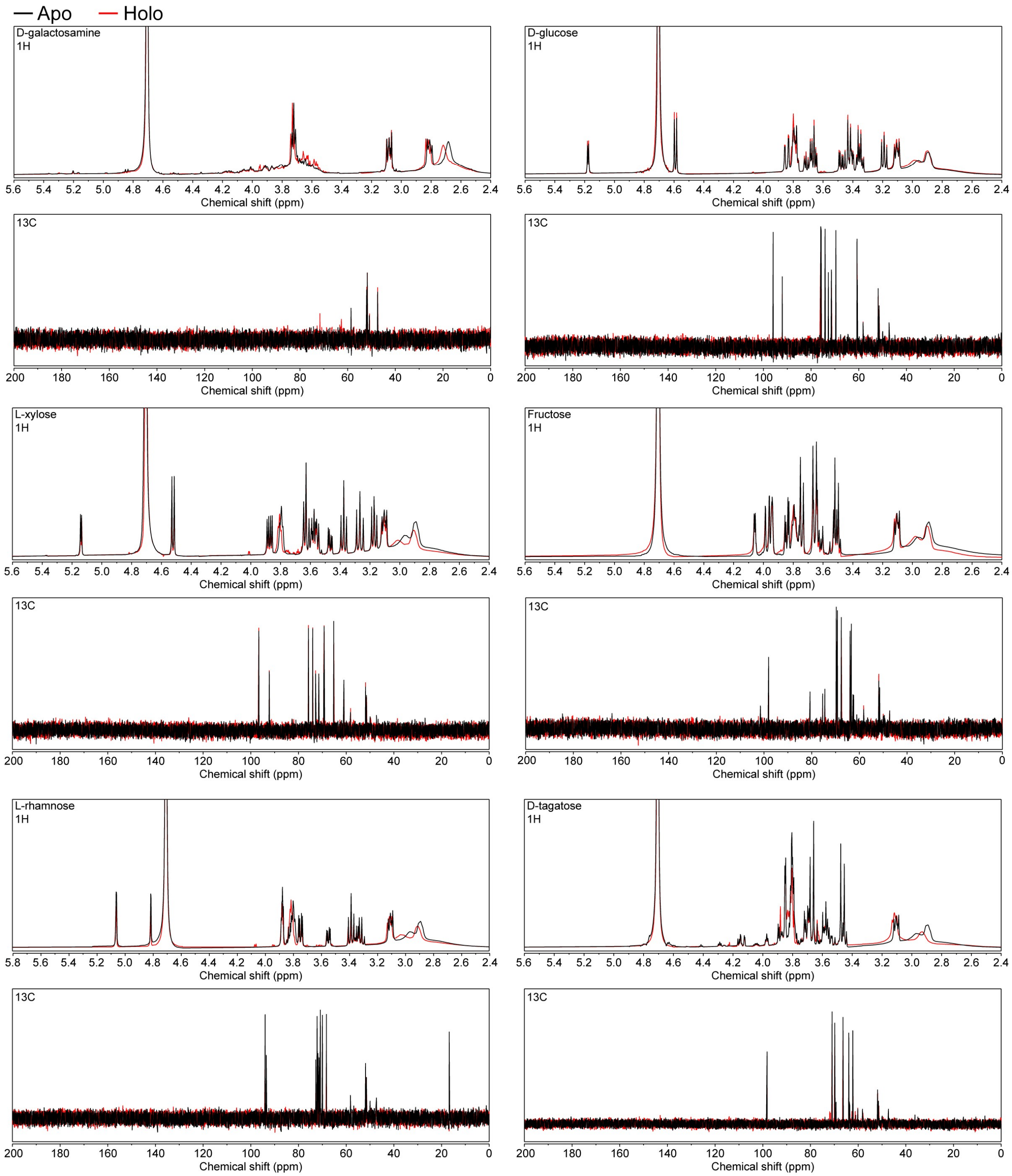

Supplementary Figure 10. NMR reactions to test NphT activity (continued).

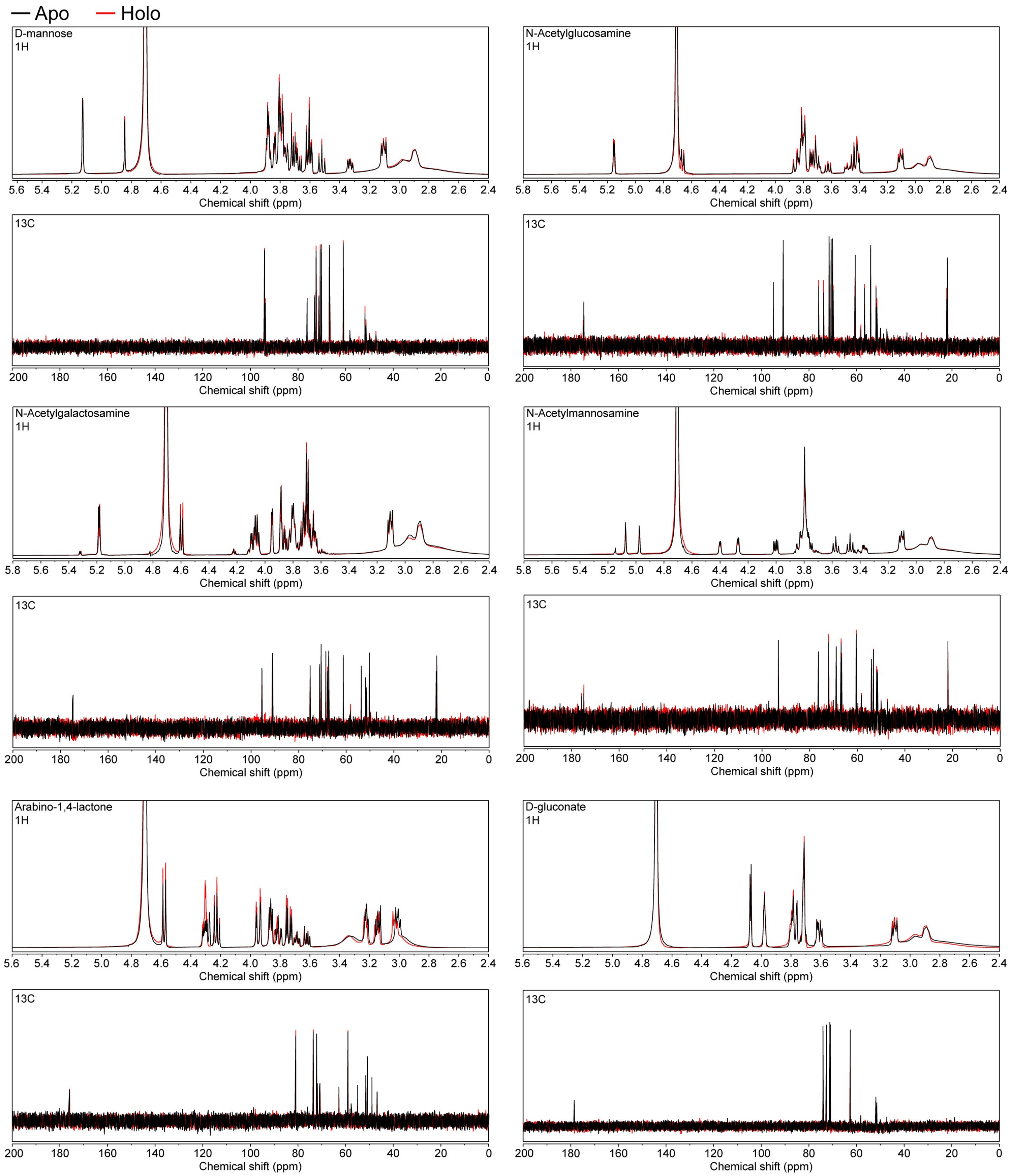

Supplementary Figure 10. NMR reactions to test NphT activity (continued).

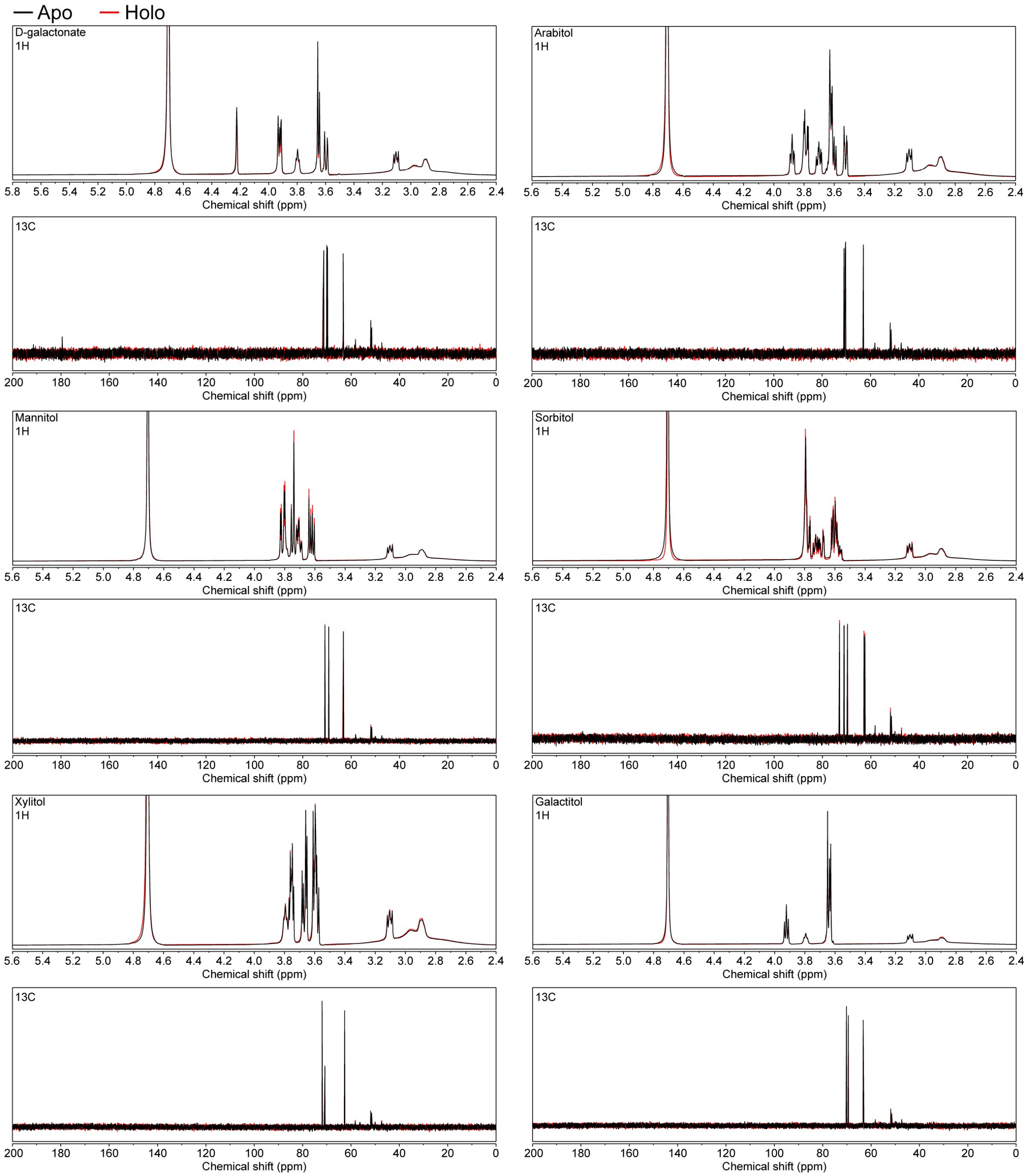

Supplementary Figure 11. LC-MS data for assessing NphT activity with NAD(P)(H). Reactions (30 µL) containing apo NphT (5 µM), sugar substrate (10 mM), and NAD(P)(H) cofactor (10 mM) or $NAD(P)^+$ /NAD(P)(H) cofactor pair (5 mM each) in 25 mM HEPES pH 7.5 were incubated at 50°C. Holo NphT and water were used for positive and negative controls, respectively. After 5 hours, 10 µL of each reaction was removed from the chamber and quenched in 70 µL of 6:1 acetonitrile:water and freeze-thawed on dry ice for a total of 3 times. After the final thaw, 10 µL of methanol was added and the sample was briefly vortexed before centrifugation for 10 minutes at 13000 x *g* and transfer of the supernatant into an HPLC vial for LC-MS analysis. Data are representative of n=2 experimental replicates.

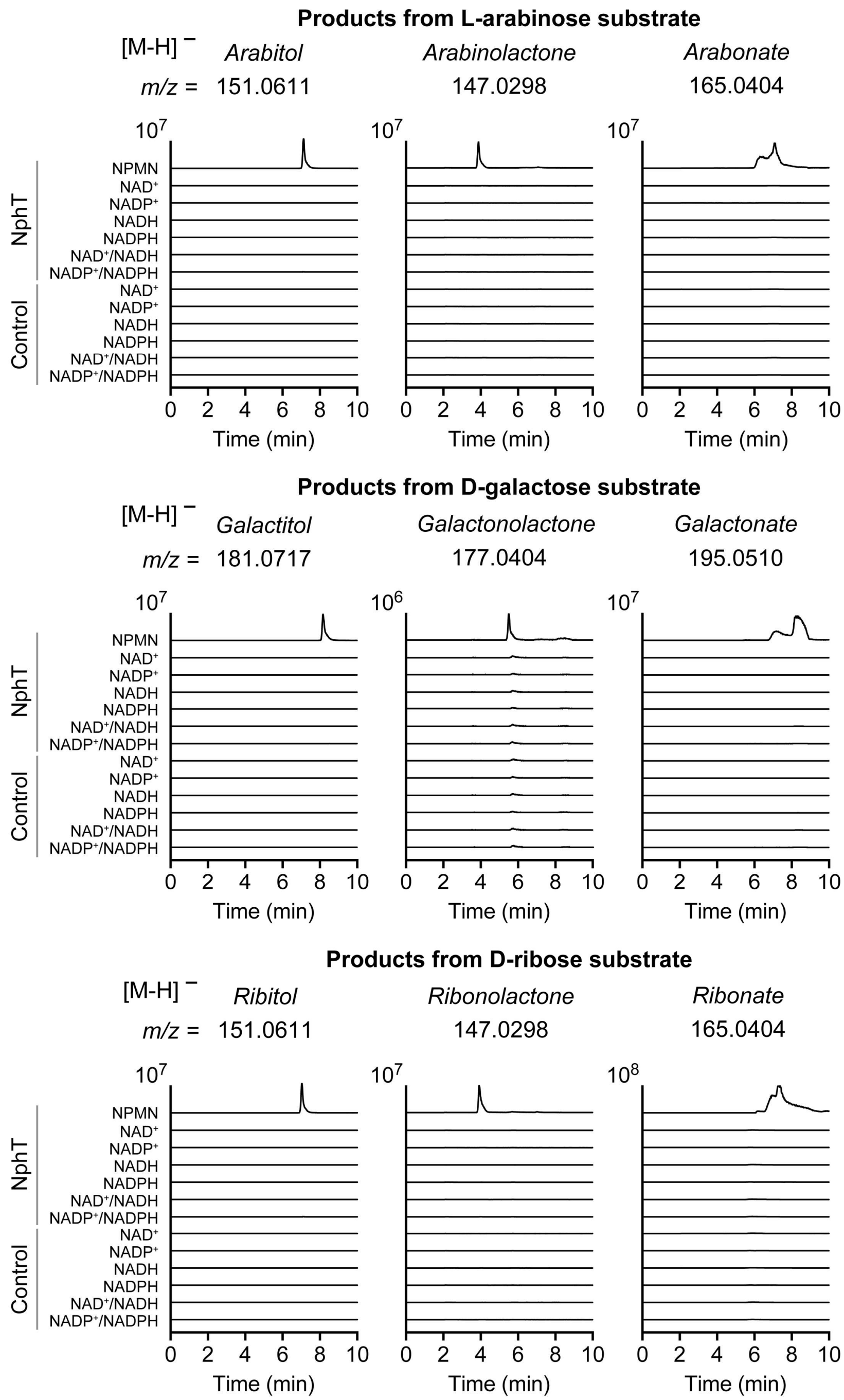

Supplementary Figure 12. NMR reactions to test NphT activity on weak substrates. In a Coy anaerobic chamber, the NPMN cofactor was synthesized *in vitro* as described in the methods and incubated with 40 µM NphT for 20 hours. A portion of the loaded protein was set aside for protein mass spectrometry to verify loading. NphT was exchanged into water using a PD-10 column, and fractions were combined and concentrated to 6.39 mg/mL using a 500 µL Modified PES VWR Centrifugal Filter, 10 kDa MWCO (VWR). Reactions (100 µL) containing holo (+NPMN) or apo (-NPMN) NphT (20 µM) were incubated with a given substrate (45 mM), in 20 mM HEPES pH 7.5 for 20 hours at 50°C. After drying down in a speed vacuum concentrator, the samples were resuspended in a final volume of 600 µL in D_2O before being transferred to a tube for NMR analysis. ^1H and ^{13}C spectra were obtained for each sample. Apo (-NPMN, black) and Holo (+NPMN, red) spectra are overlaid below. Residual water appears at ~4.7 ppm. This experiment was performed once.

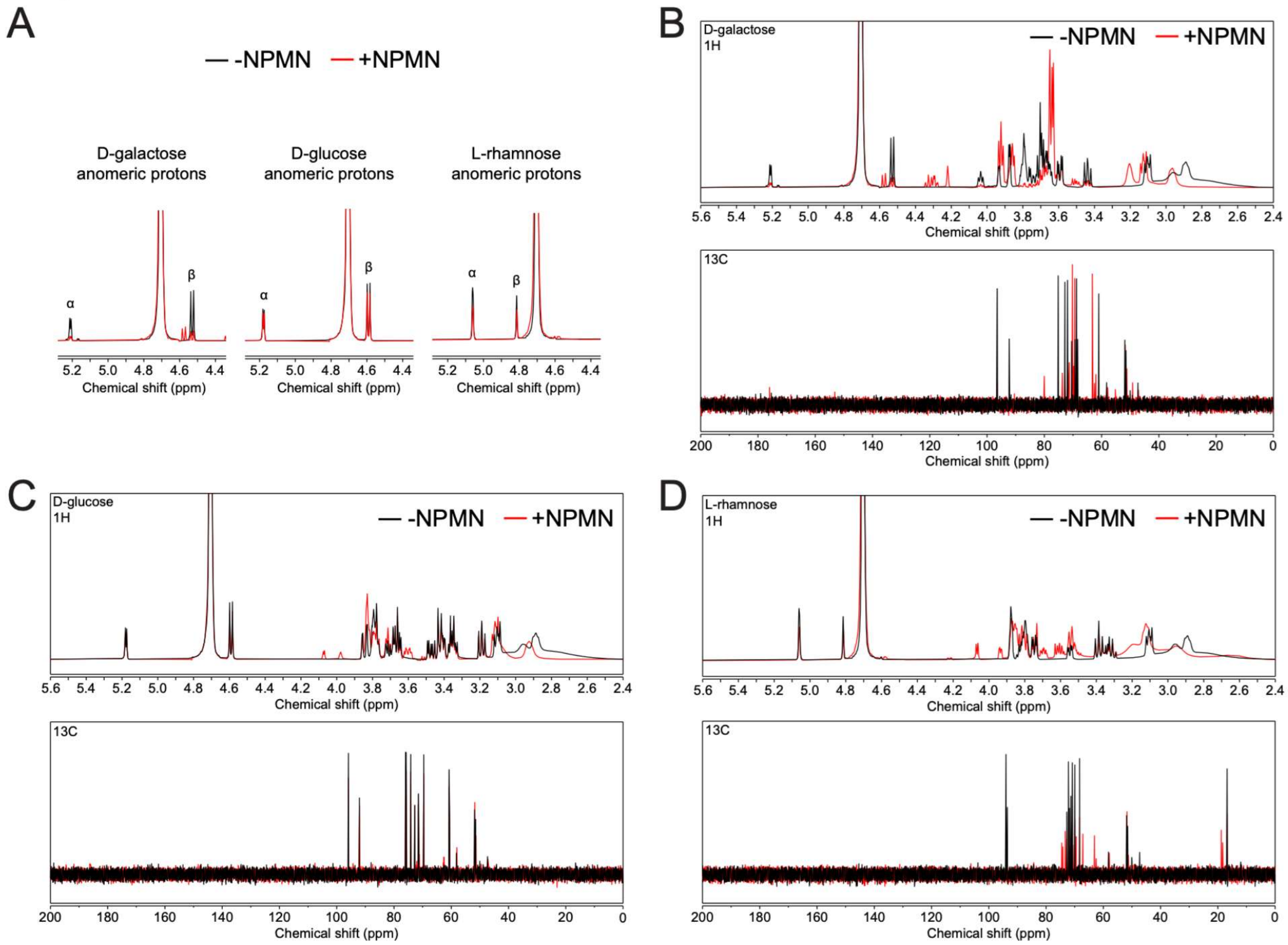

Supplementary Figure 13. ¹H NMR kinetics to monitor NphT activity on gluconolactone and sorbitol to check for reversibility. In a Coy anaerobic chamber, the NPMN cofactor was synthesized *in vitro* and incubated with 40 µM NphT for 20 hours as described in the methods. A portion of the loaded protein was set aside for protein mass spectrometry to verify loading. NphT was exchanged into D_2O using a PD-10 column, and fractions were combined and concentrated to 7.68 mg/mL using a 500 µL Modified PES VWR Centrifugal Filter, 10 kDa MWCO (VWR). Reactions (100 µL) containing holo (+NPMN) or apo (-NPMN) NphT (40 µM) were prepared with glucono-1,4-lactone and sorbitol (25 mM each) in 20 mM HEPES pH 7.5 in an anaerobic NMR tube, then topped with 500 µL of D_2O. Samples were removed from the glove box, and then ^{1}H NMR kinetics spectra were collected every 10 minutes over 5 hours while incubating at 50°C. +NPMN spectra are shown at one-hour intervals for clarity. This experiment was performed once.

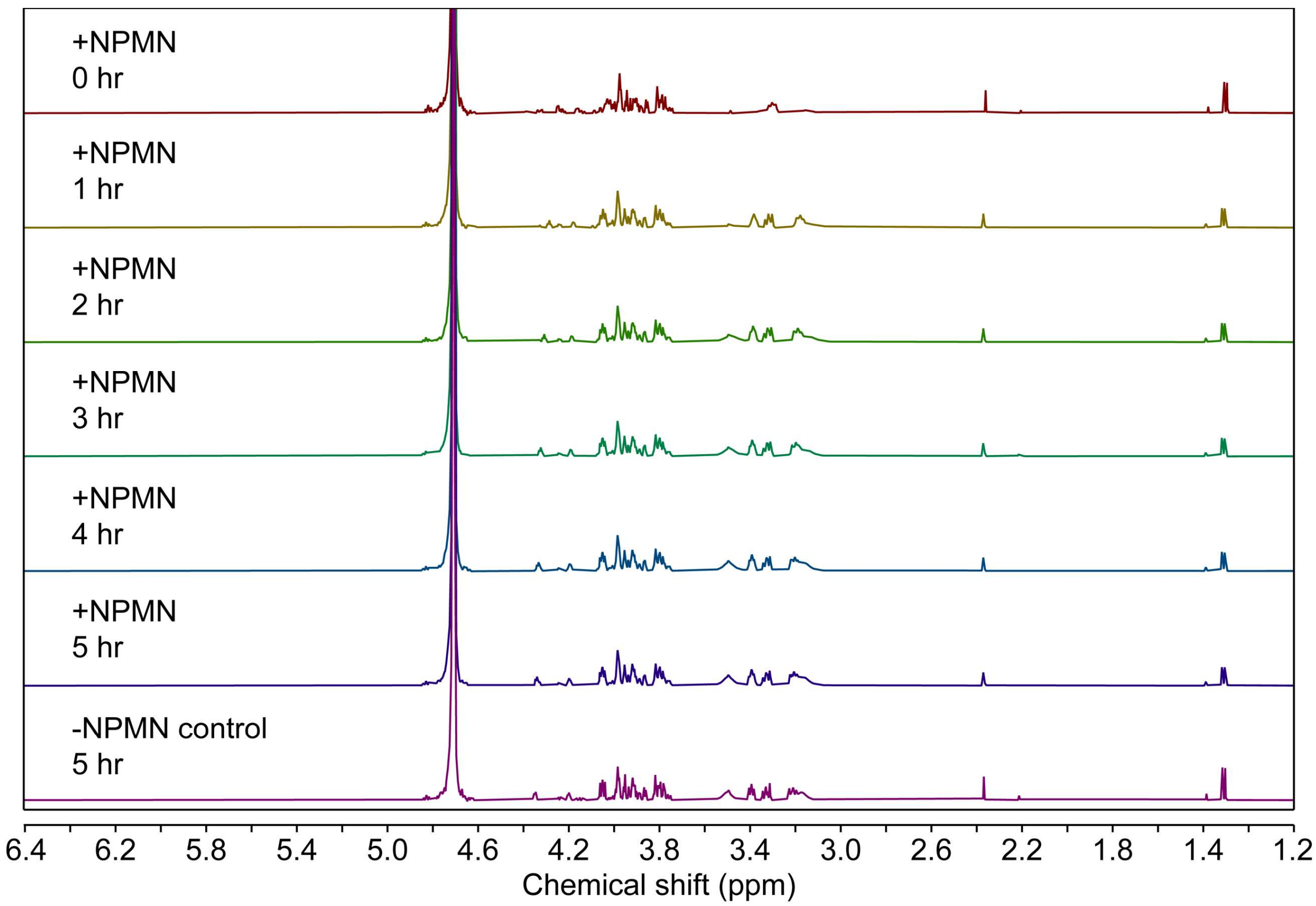

Supplementary Figure 14. NMR analysis of products from incubating NphT with L-arabinose. In a Coy anaerobic chamber, the NPMN cofactor was synthesized as described in the methods and incubated with 40 µM NphT for 20 hours. A portion of the loaded protein was set aside for protein mass spectrometry to verify loading. NphT was exchanged into water using a PD-10 column, and fractions were combined and concentrated to 6.39 mg/mL using a 500 µL Modified PES VWR Centrifugal Filter, 10 kDa MWCO (VWR). Reactions (100 µL) containing holo (+NPMN) or apo (-NPMN) NphT (95 µM) were incubated with L-arabinose (45 mM) in 20 mM HEPES pH 7.5 for 20 hours at 50°C. After drying down in a speed vacuum concentrator, the samples were resuspended in a final volume of 600 µL in D_2O before being transferred to a tube for NMR analysis. (A) 1-D overlaid ^{1}H NMR spectra. (B) COSY experiment. (C) HSQC experiment. (D) HMBC experiment. (E) Chemical shifts determined from the +NPMN 2D-NMR data. Spectral assignments for products are provided in list format in the NMR analysis of the standard supplemental figures below. Apo-enzyme spectra are shown in black and holo-enzyme spectra are shown in red. Residual water appears at ~4.7 ppm. Data are representative of n=3 experimental replicates.

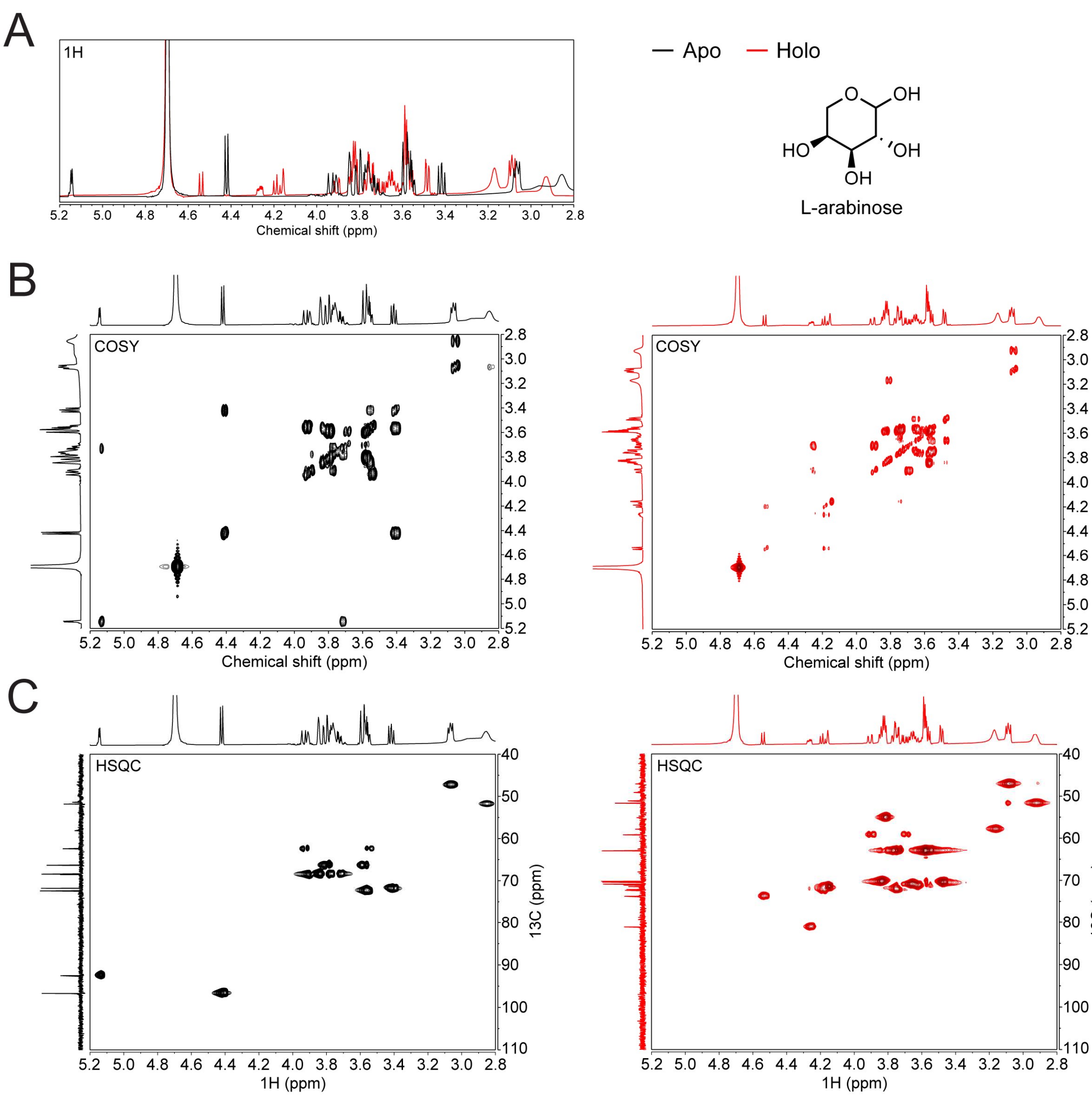

D

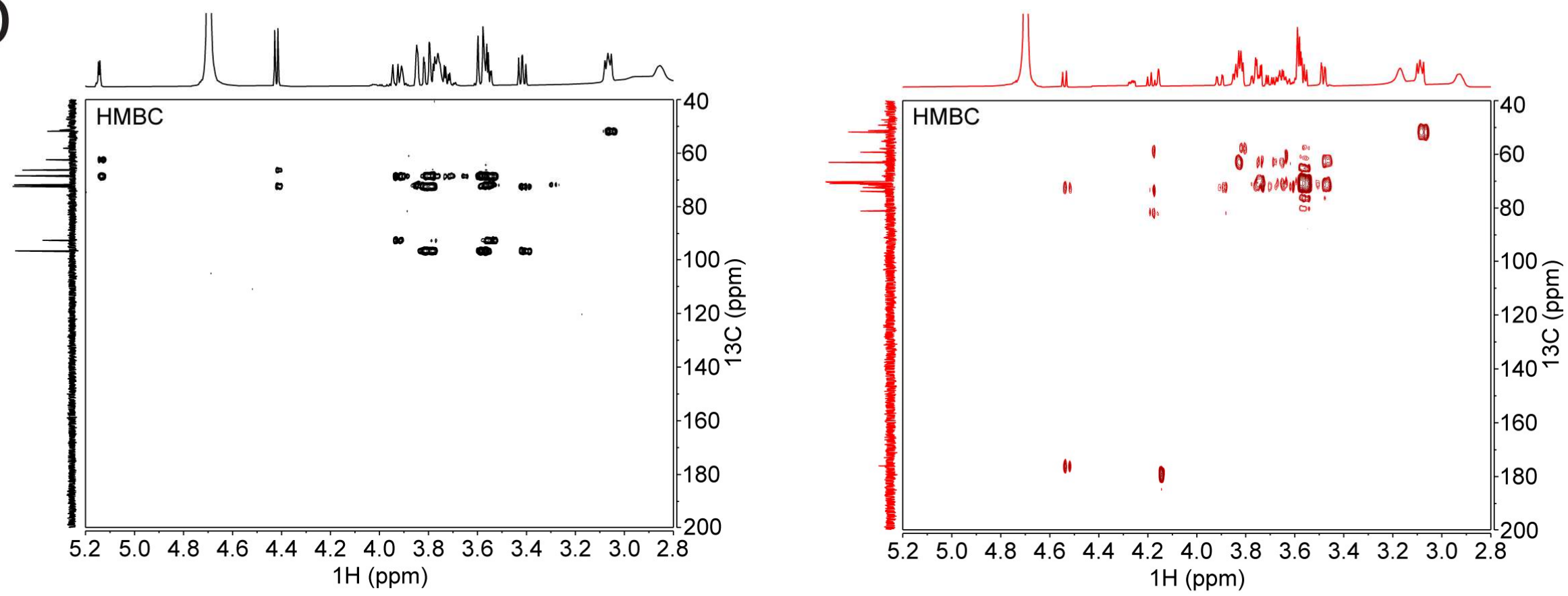

E

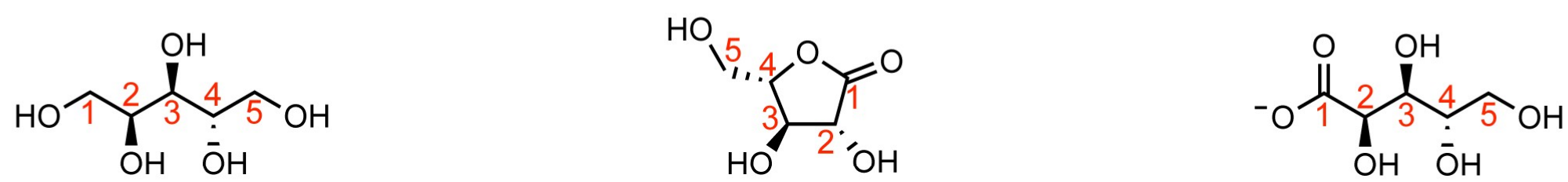

Arabitol

Arabino-1,4-lactone

Arabonate

	1H (ppm)	13C (ppm)
1	3.75, dd	62.90
2	3.84, ddd	72.22
3	3.48, dd	70.42
4	3.66, ddd	70.88
5	3.58, m	62.99

	1H (ppm)	13C (ppm)
1	-	176.12
2	4.54, d	73.76
3	4.19, t	72.37
4	4.27, ddd	81.13
5	3.91, dd	59.19
	3.70, dd	

	1H (ppm)	13C (ppm)
1	-	179.11
2	4.15, d	71.46
3	3.76, m	72.14
4	3.64, ddd	71.13
5	3.76, m	63.06
	3.58, dd	

Supplementary Figure 15. NMR analysis of L-arabitol standard for comparison to NphT products. 5 mM arabitol was prepared in 600 µL of D_2O before transferring to a tube for NMR analysis. (A) Structure and table of NMR assignments. ^{1}H NMR (600 MHz, D_2O) δ 3.84 (ddd, J = 7.4, 5.5, 2.1 Hz, 1H), 3.75 (dd, J = 11.8, 3.0 Hz, 1H), 3.66 (ddd, J = 8.8, 6.4, 2.9 Hz, 1H), 3.58 (m, 3H), 3.48 (dd, J = 8.4, 2.1 Hz, 1H). ^{13}C NMR (151 MHz, D_2O) δ 70.88, 70.42, 70.22, 62.99, 62.90. (B) ^{1}H and ^{13}C spectra. (C) COSY experiment. (D) HSQC experiment. (E) HMBC experiment. Residual water appears at ~4.7 ppm. These data were collected once.

A

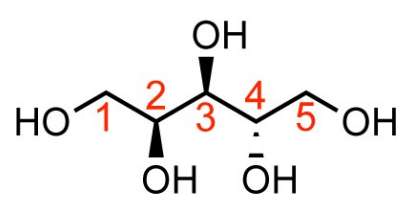

Arabitol

	1H (ppm)	13C (ppm)
1	3.75, dd	62.90
2	3.84, ddd	72.22
3	3.48, dd	70.42
4	3.66, ddd	70.88
5	3.58, m	62.99

B

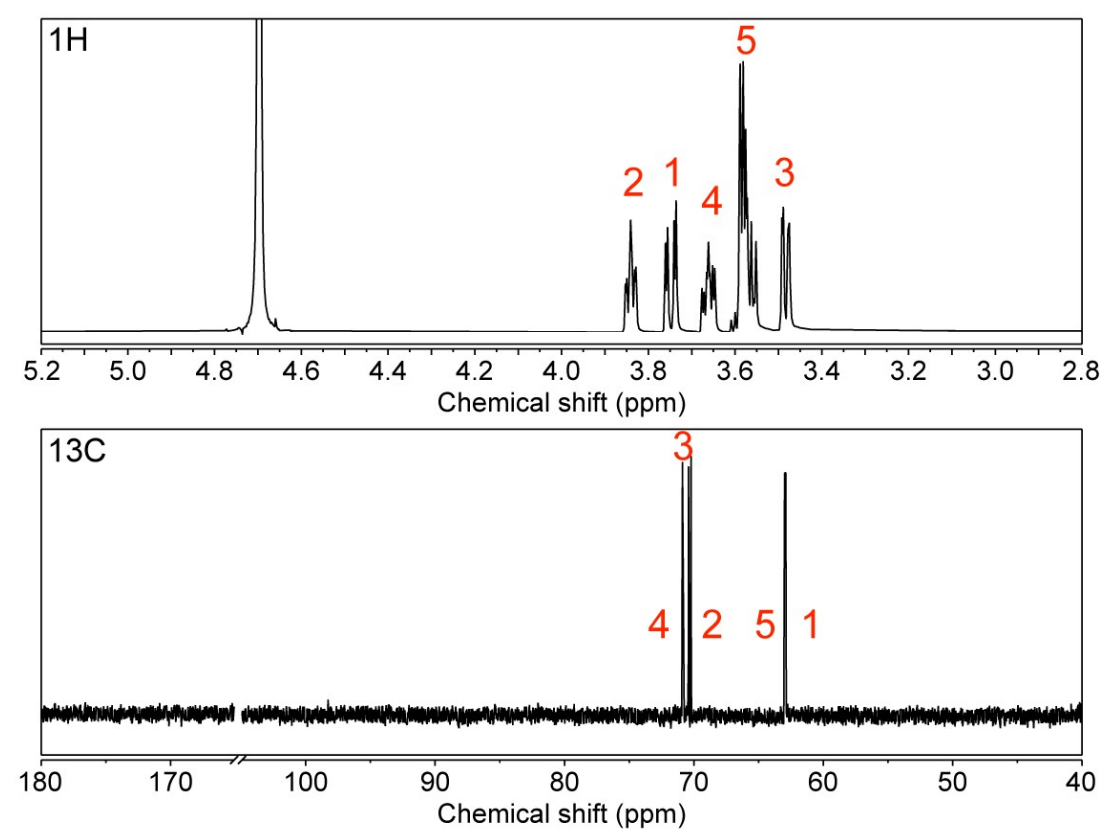

C

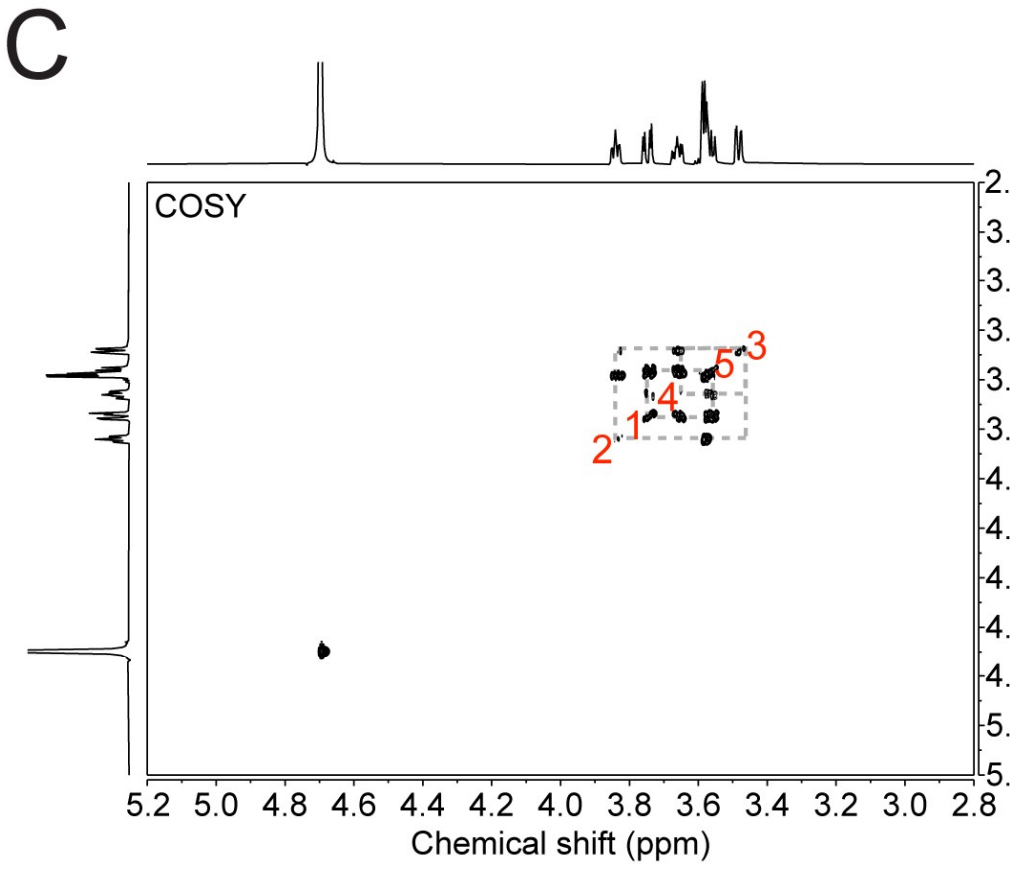

D

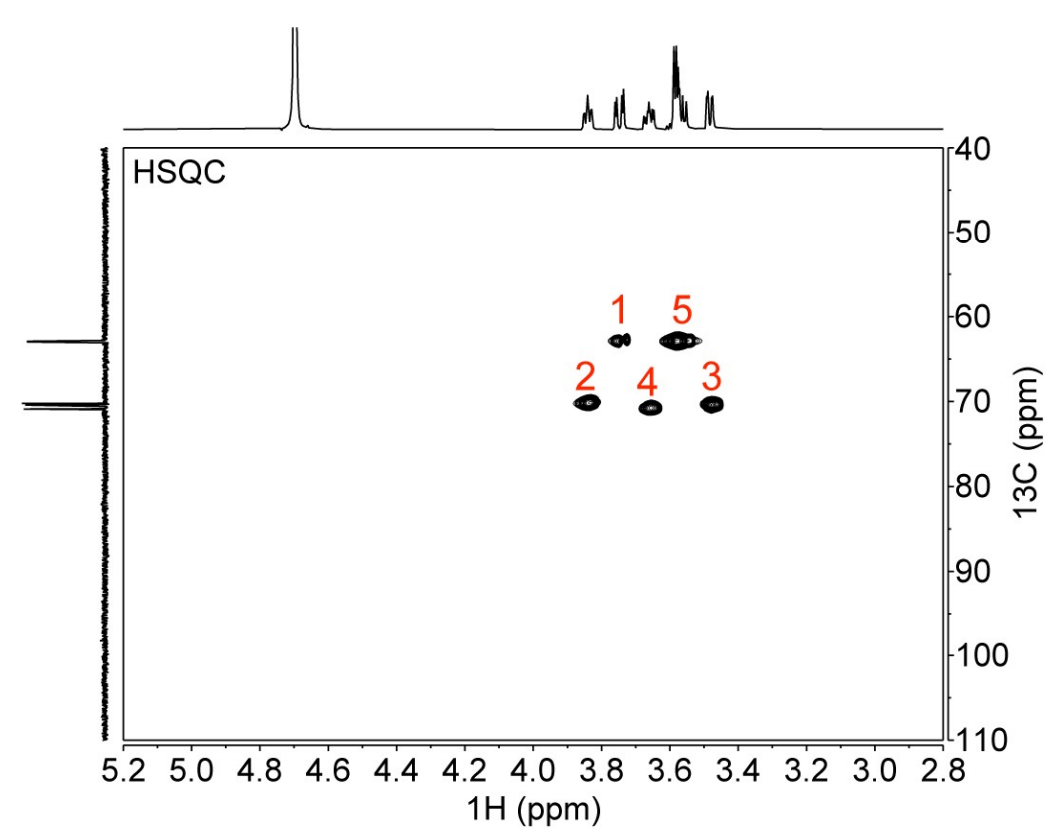

E

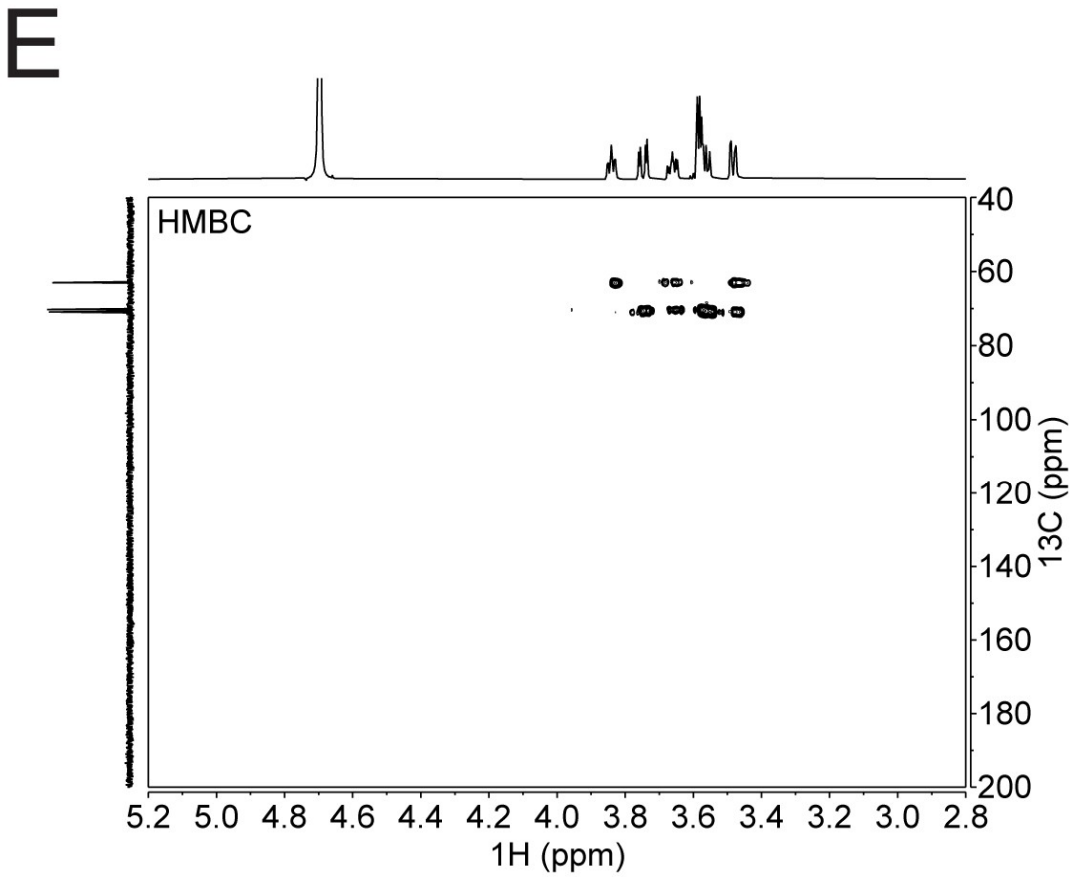

Supplementary Figure 16. NMR analysis of D-arabinolactone standard for comparison to NphT products. 5 mM D-arabino-1,4-lactone was prepared in 600 µL of D_2O before transferring to a tube for NMR analysis. (A) Structure and table of NMR assignments. ^{1}H NMR (600 MHz, D_2O) δ 4.54 (d, J = 9.2 Hz, 1H), 4.27 (ddd, J = 8.5, 4.7, 2.2 Hz, 1H), 4.19 (t, J = 8.9 Hz, 1H), 3.91 (dd, J = 13.3, 2.3 Hz, 1H), 3.70 (dd, J = 13.3, 4.8 Hz, 1H). ^{13}C NMR (151 MHz, D_2O) δ 176.12, 81.13, 73.76, 72.37, 59.19. (B) ^{1}H and ^{13}C spectra. (C) COSY experiment. (D) HSQC experiment. (E) HMBC experiment. Residual water appears at ~4.7 ppm. These data were collected once. Note that L-arabino-1,4-lactone was not commercially available.

A

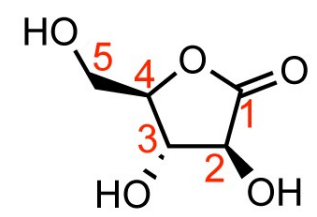

D-Arabino-1,4-lactone

	1H (ppm)	13C (ppm)
1	-	176.12
2	4.54, d	73.76
3	4.19, t	72.37
4	4.27, ddd	81.13
5	3.91, dd	59.19
	3.70, dd	

B

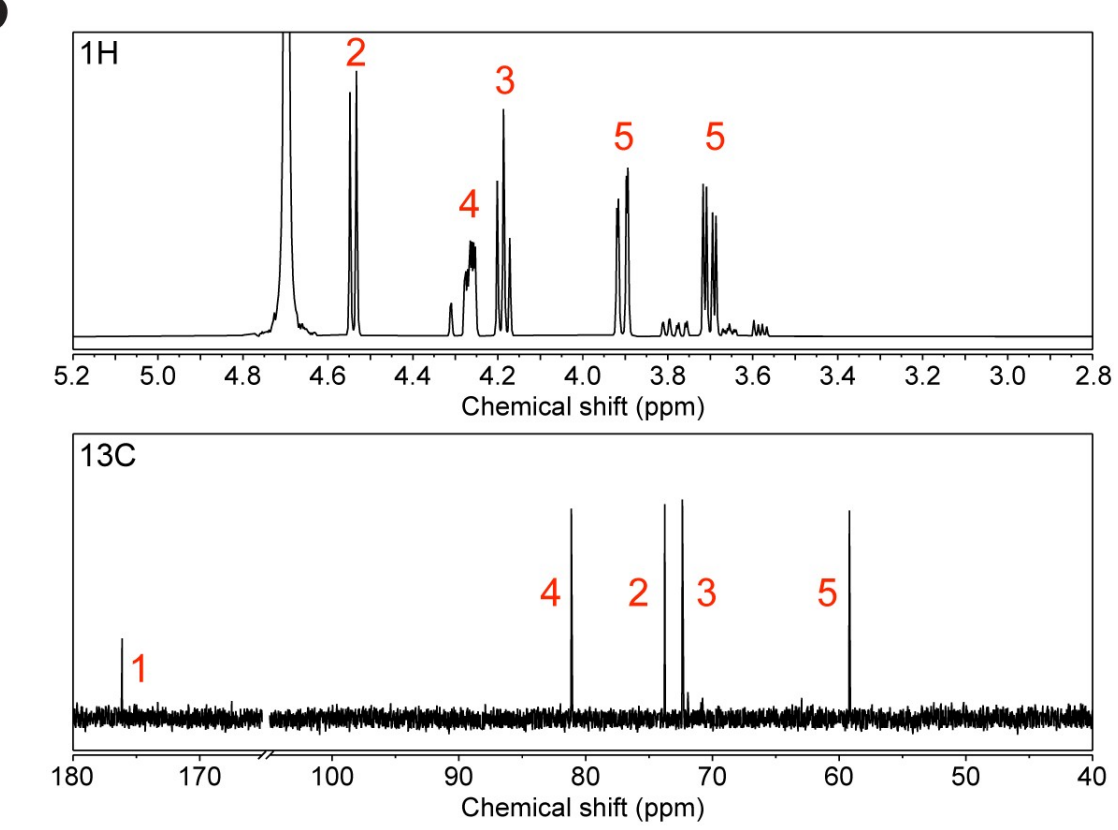

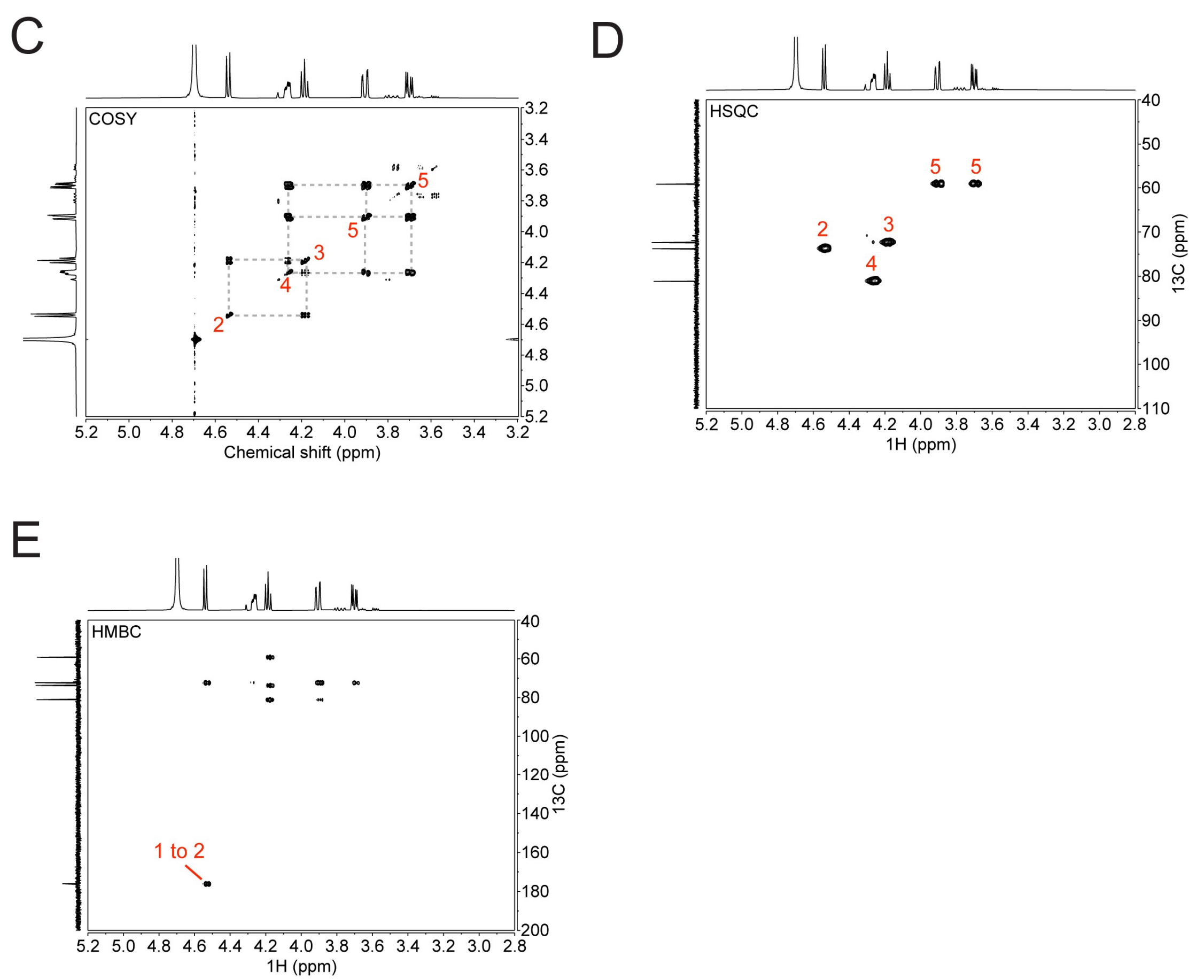

Supplementary Figure 17. NMR analysis of D-arabonate standard for comparison to NphT products. To make 5 mM D-arabonate, a 100 µL stock of 30 mM D-arabino-1,4-lactone containing 30 mM NaOH prepared from a 3 M stock in D_2O was incubated at 100°C for 1 hour. Upon cooling, the sample was brought to a final volume of 600 µL in D_2O and transferred to a tube for NMR analysis. Full conversion of arabino-1,4-lactone to arabonate did not occur, resulting in a mixture. In this figure, only the assignments for the arabonate signals are provided. (A) Structure and table of NMR assignments. ^{1}H NMR (600 MHz, D_2O) δ 4.15 (d, J = 1.7 Hz, 1H), 3.76 (m, 2H), 3.64 (ddd, J = 8.9, 6.4, 2.6 Hz, 1H), 3.58 (dd, J = 11.8, 6.4 Hz, 1H). ^{13}C NMR (151 MHz, D_2O) δ 179.11, 72.14, 71.46, 71.13, 63.06. (B) ^{1}H and ^{13}C spectra. (C) COSY experiment. (D) HSQC experiment. (E) HMBC experiment. Residual water appears at ~4.7 ppm. These data were collected once. Note that L-arabino-1,4-lactone was not commercially available.

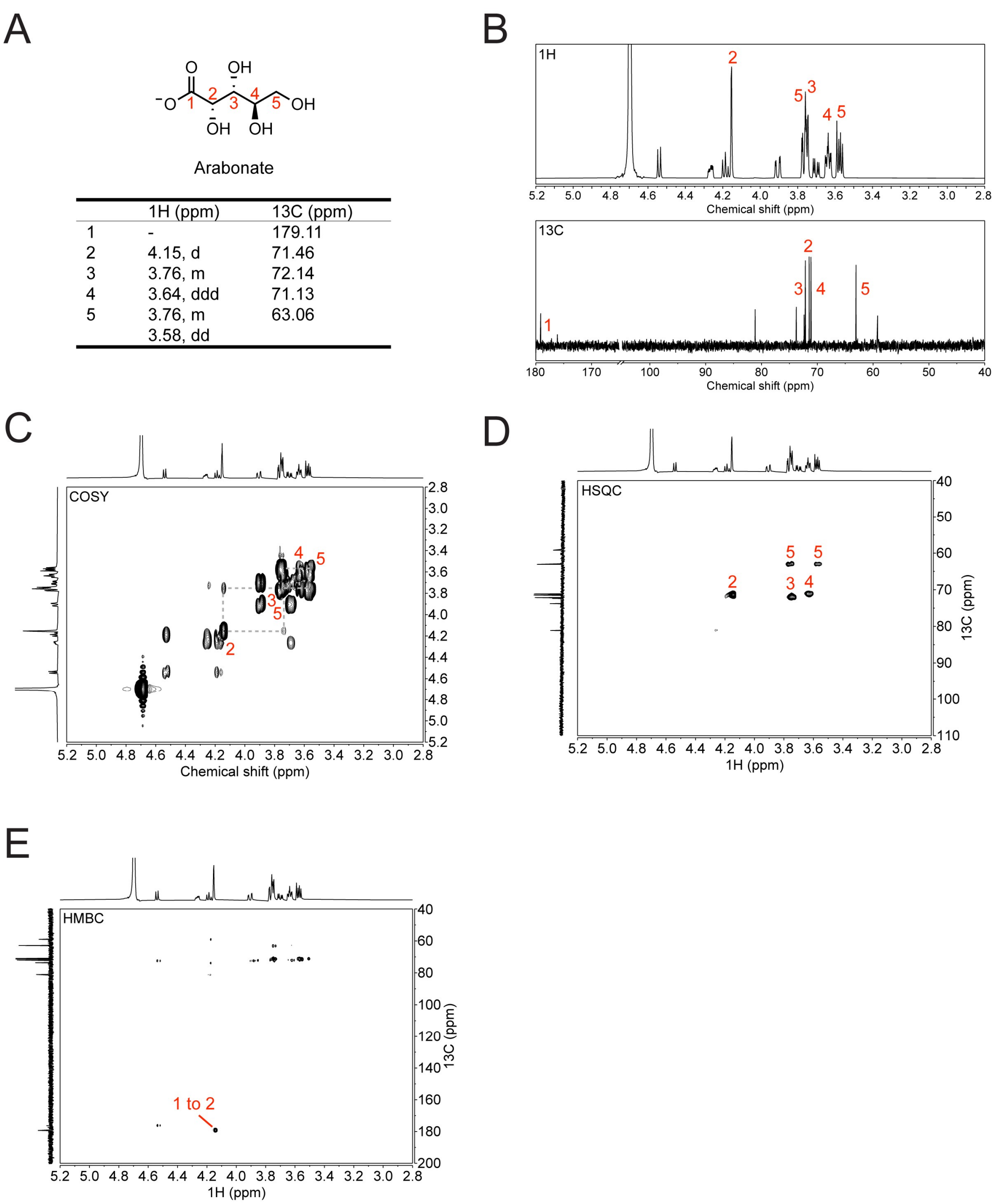

	1H (ppm)	13C (ppm)
1	-	179.11
2	4.15, d	71.46
3	3.76, m	72.14
4	3.64, ddd	71.13
5	3.76, m<br>3.58, dd	63.06

Supplementary Figure 18. NMR analysis of products from incubating NphT with D-galactose. In a Coy anaerobic chamber, the NPMN cofactor was synthesized *in vitro* as described in the methods and incubated with 40 µM NphT for 20 hours. A portion of the loaded protein was set aside for protein mass spectrometry to verify loading. NphT was exchanged into water using a PD-10 column, and fractions were combined and concentrated to 6.39 mg/mL using a 500 µL Modified PES VWR Centrifugal Filter, 10 kDa MWCO (VWR). Reactions (100 µL) containing holo (+NPMN) or apo (-NPMN) NphT (95 µM) were incubated with D-galactose (45 mM) in 20 mM HEPES pH 7.5 for 20 hours at 50°C. After drying down in a speed vacuum concentrator, the samples were resuspended in a final volume of 600 µL in D_2O before being transferred to a tube for NMR analysis. (A) 1-D overlaid ^{1}H NMR spectra. (B) COSY experiment. (C) HSQC experiment. (D) HMBC experiment. (E) Chemical shifts determined from the +NPMN 2D-NMR data. Spectral assignments for products are provided in list format in the NMR analysis of the standard supplemental figures below. -NPMN spectra are shown in black, and +NPMN spectra are shown in red. Residual water appears at ~4.7 ppm. Data are representative of n=3 experimental replicates.

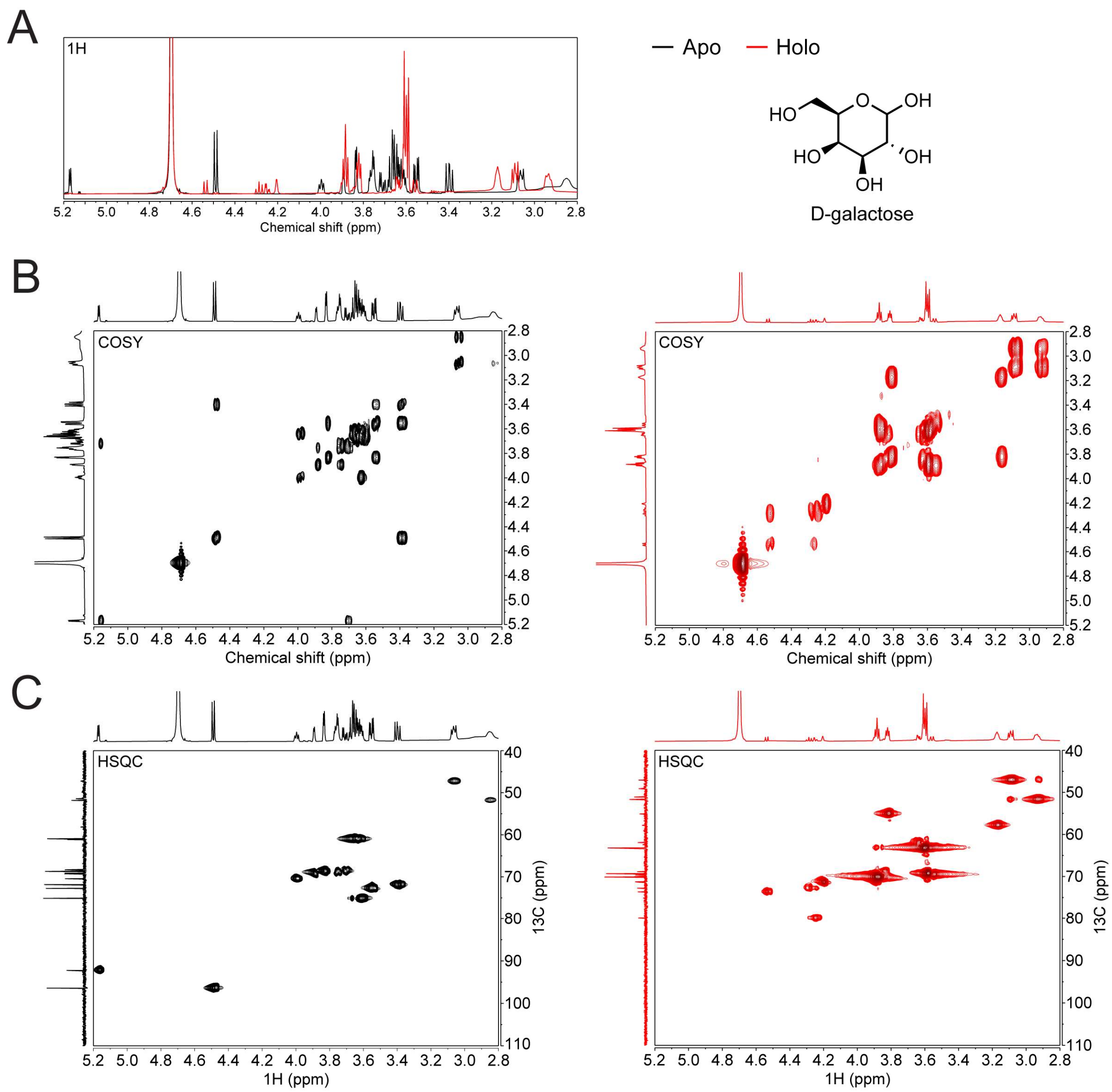

D

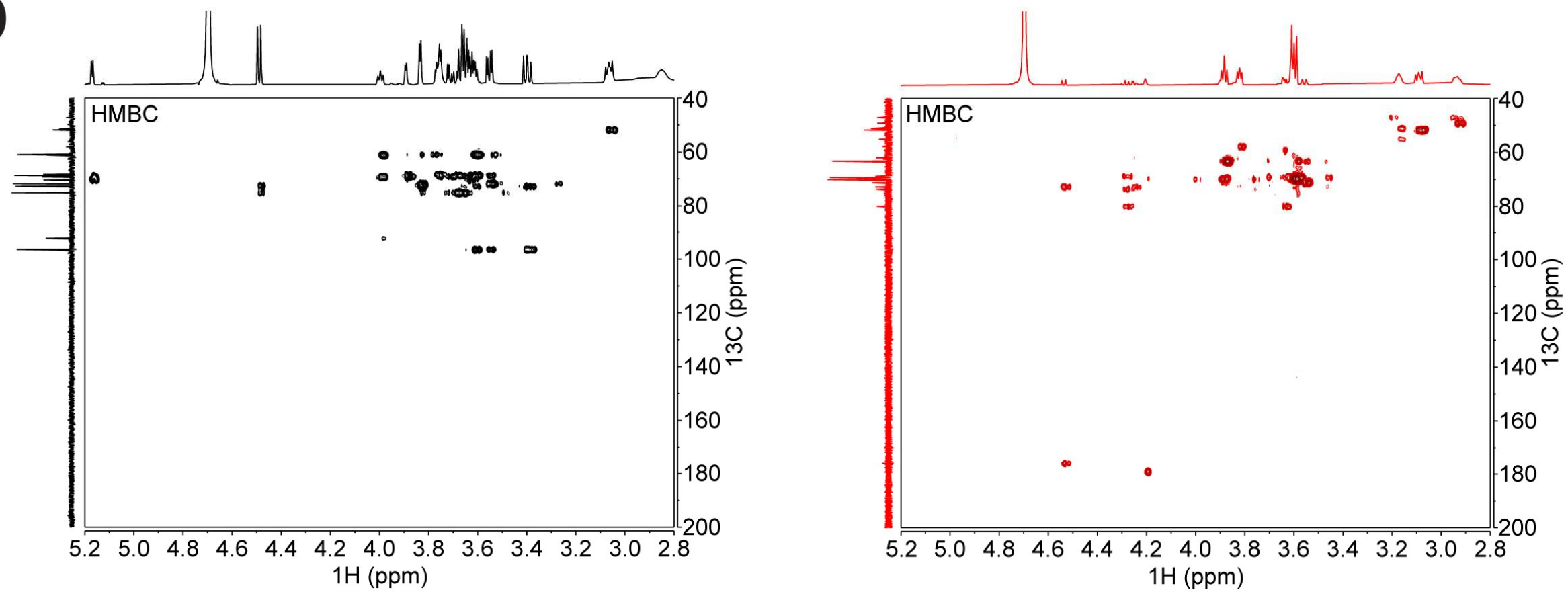

E

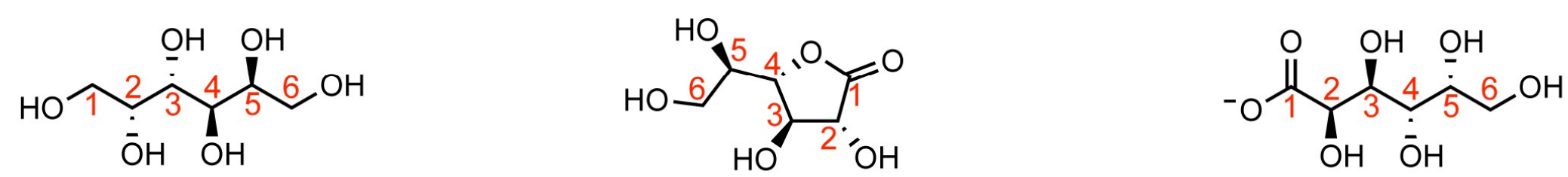

Galactitol

Galactono-1,4-lactone

Galactonate

Galactitol

	1H (ppm)	13C (ppm)
1	3.60, m	63.21
2	3.88, t	70.13
3	3.60, m	69.32
4	3.60, m	69.32
5	3.88, t	70.13
6	3.60, m	63.21

Galactono-1,4-lactone

	1H (ppm)	13C (ppm)
1	-	175.94
2	4.54, d	73.66
3	4.29, t	72.78
4	4.25, dd	79.97
5	3.84, ddd	68.86
6	3.64, dd	61.94

Galactonate

	1H (ppm)	13C (ppm)
1	-	179.49
2	4.19, d	71.58
3	3.88, m	71.27
4	3.56, dd	69.70
5	3.88, m	70.03
6	3.61, m	63.26

Supplementary Figure 19. NMR analysis of galactitol standard for comparison to NphT products. 5 mM galactitol was prepared in 600 µL of D_2O before transferring to a tube for NMR analysis. (A) Structure and table of NMR assignments. ^{1}H NMR (600 MHz, D_2O) δ 3.88 (t, J = 6.4 Hz, 2H), 3.63 – 3.56 (m, 6H). ^{13}C NMR (151 MHz, D_2O) δ 70.13, 69.32, 63.21. (B) ^{1}H and ^{13}C spectra. (C) COSY experiment. (D) HSQC experiment. (E) HMBC experiment. Residual water appears at ~4.7 ppm. These data were collected once.

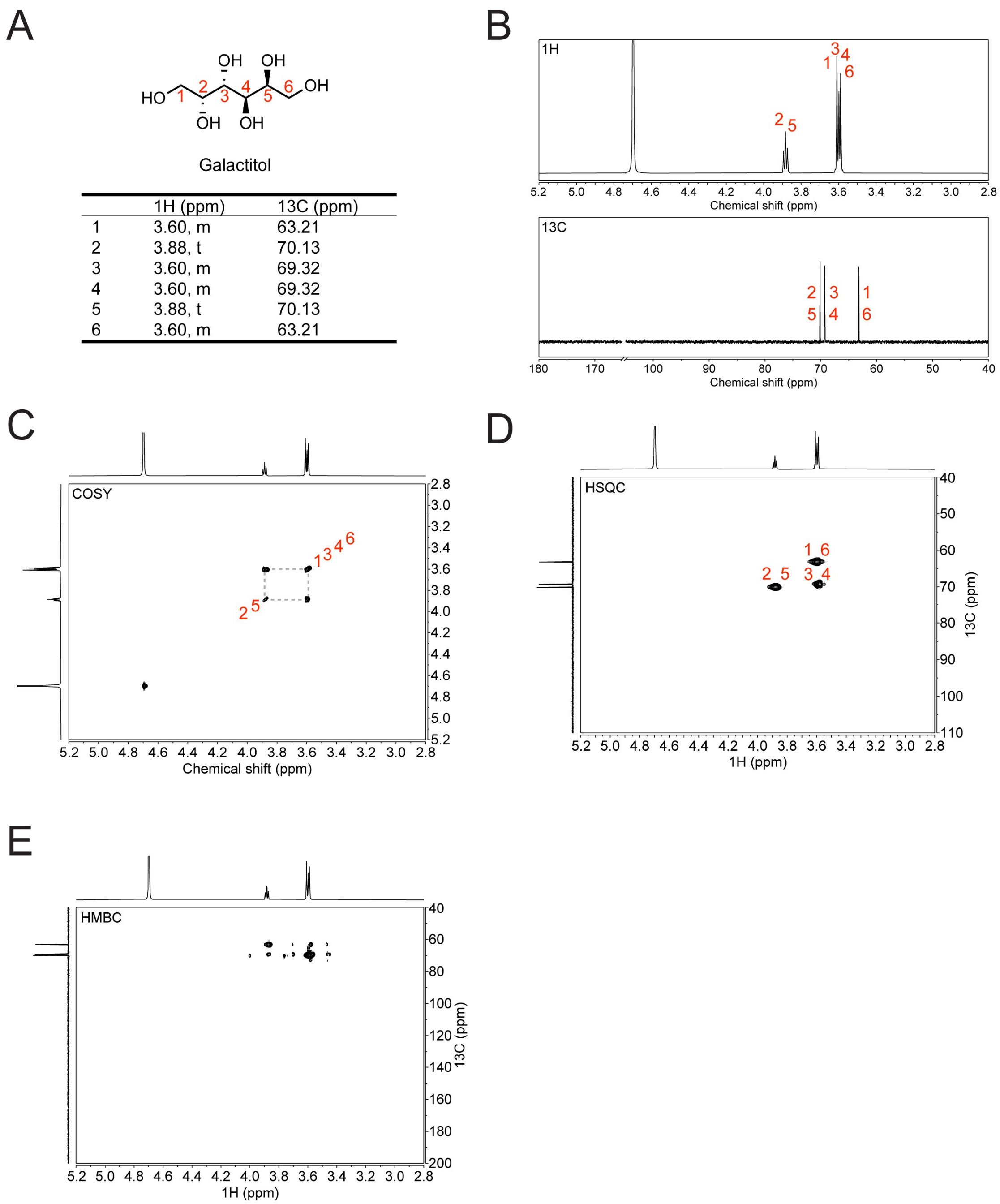

	1H (ppm)	13C (ppm)
1	3.60, m	63.21
2	3.88, t	70.13
3	3.60, m	69.32
4	3.60, m	69.32
5	3.88, t	70.13
6	3.60, m	63.21

Supplementary Figure 20. NMR analysis of galactono-1,4-lactone standard for comparison to NphT products. To make 5 mM D-galactono-1,4-lactone, a 300 µL stock of 10 mM commercial D-galactonate containing 30 mM HCl was incubated at 100°C for 1 hour. Upon cooling, the sample was brought to a final volume of 600 µL in D_2O and transferred to a tube for NMR analysis. Full conversion of galactonate to galactono-1,4-lactone did not occur, resulting in a mixture. In this figure, only the assignments for the galactono-1,4-lactone signals are provided. (A) Structure and table of NMR assignments. ^{1}H NMR (600 MHz, D_2O) δ 4.54 (d, J = 8.8 Hz, 1H), 4.29 (t, J = 8.7 Hz, 1H), 4.25 (dd, J = 8.5, 2.8 Hz, 1H), 3.84 (ddd, J = 7.0, 5.5, 2.8 Hz, 1H), 3.64 (dd, J = 6.3, 3.4 Hz, 2H). ^{13}C NMR (151 MHz, D_2O) δ 175.94, 79.97, 73.66, 72.78, 68.86, 61.94. (B) ^{1}H and ^{13}C spectra. (C) COSY experiment. (D) HSQC experiment. (E) HMBC experiment. Residual water appears at ~4.7 ppm. These data were collected once.

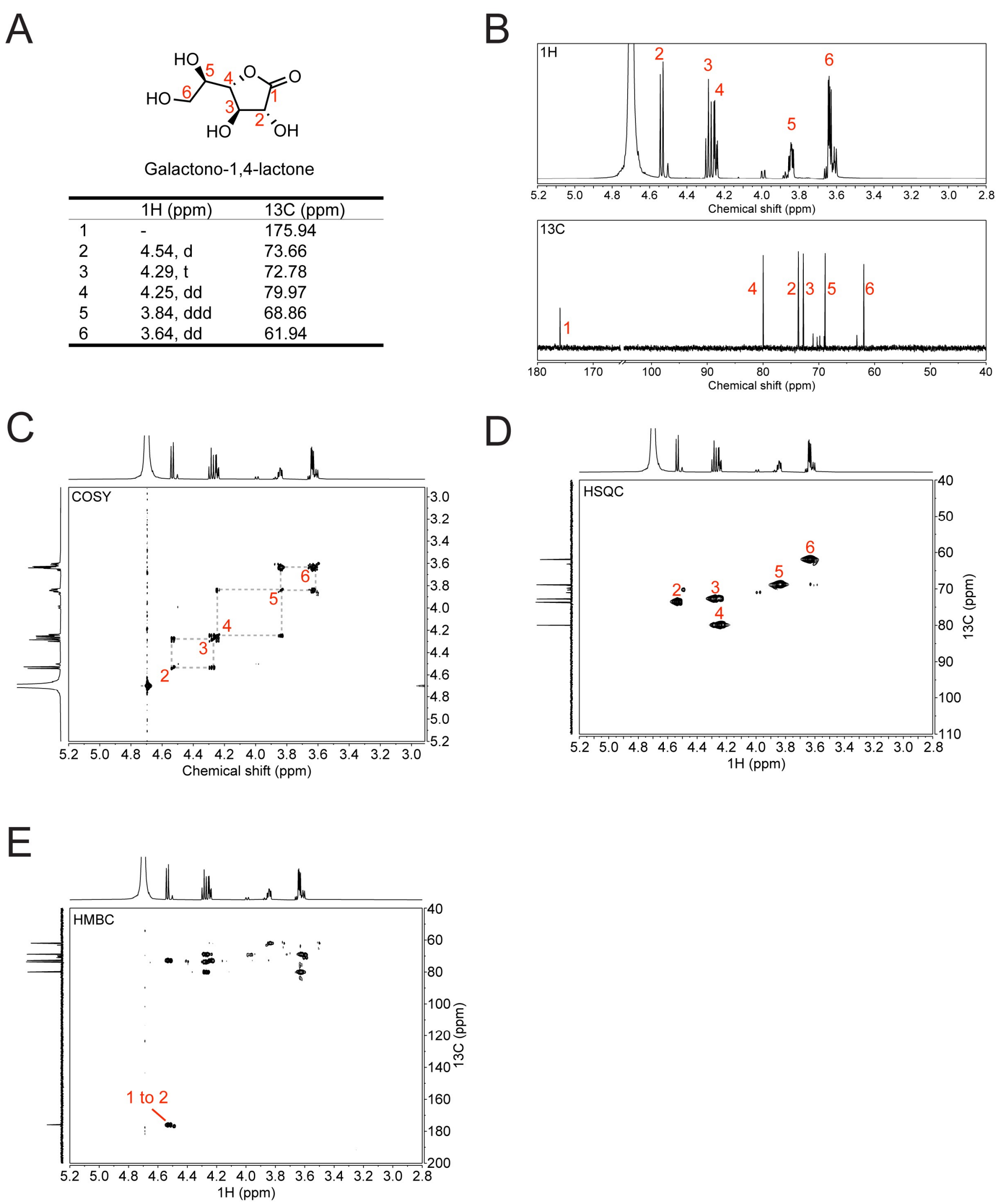

	1H (ppm)	13C (ppm)
1	-	175.94
2	4.54, d	73.66
3	4.29, t	72.78
4	4.25, dd	79.97
5	3.84, ddd	68.86
6	3.64, dd	61.94

Supplementary Figure 21. NMR analysis of galactonate standard for comparison to NphT products. 5 mM D-galactonate was prepared in 600 µL of D_2O before transferring to a tube for NMR analysis (A) Structure and table of NMR assignments. ^{1}H NMR (600 MHz, D_2O) δ 4.19 (d, J = 1.4 Hz, 1H), 3.88 (m, 2H), 3.61 (m, 2H), 3.56 (dd, J = 9.6, 1.6 Hz, 1H). ^{13}C NMR (151 MHz, D_2O) δ 179.49, 71.58, 71.27, 70.03, 69.70, 63.26. (B) ^{1}H and ^{13}C spectra. (C) COSY experiment. (D) HSQC experiment. (E) HMBC experiment. Residual water appears at ~4.7 ppm. These data were collected once.

A

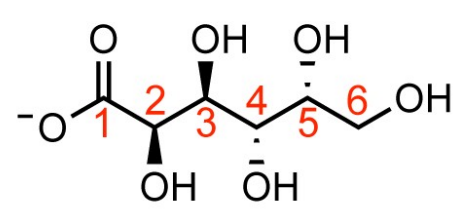

Galactonate

	1H (ppm)	13C (ppm)
1	-	179.49
2	4.19, d	71.58
3	3.88, m	71.27
4	3.56, dd	69.70
5	3.88, m	70.03
6	3.61, m	63.26

B

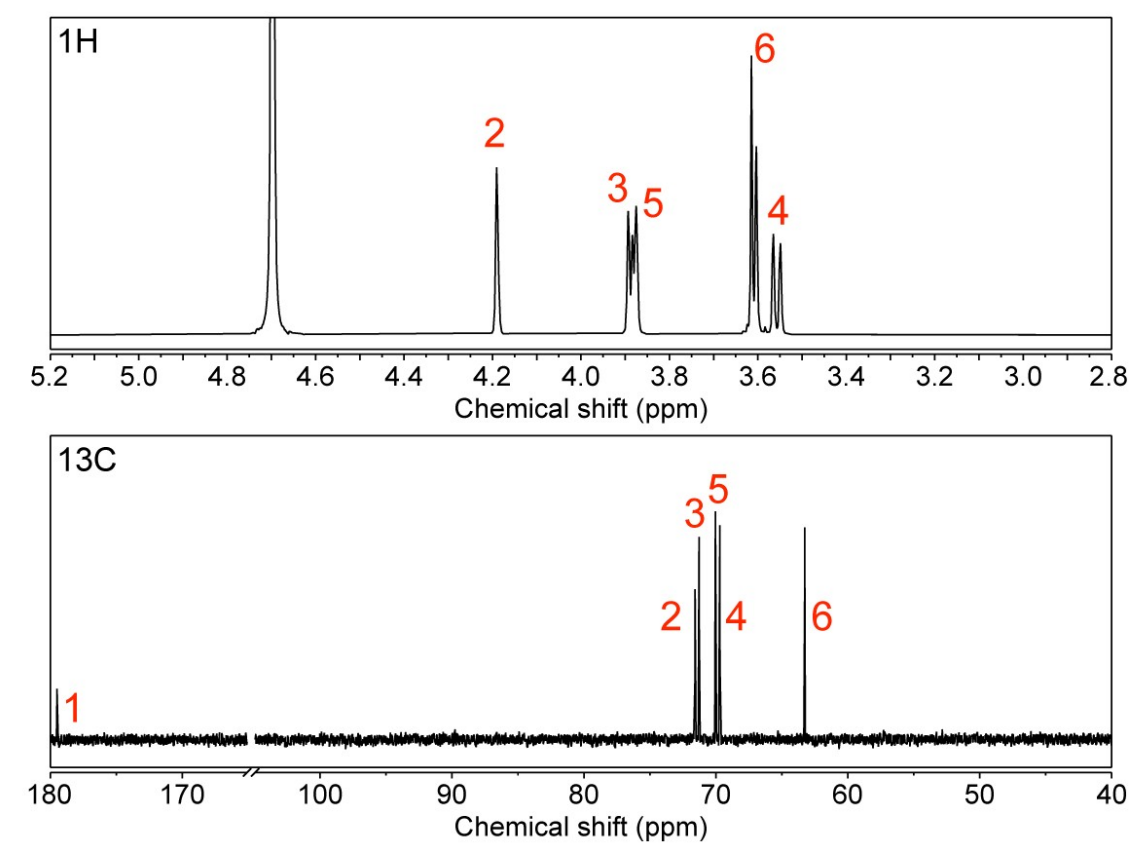

C

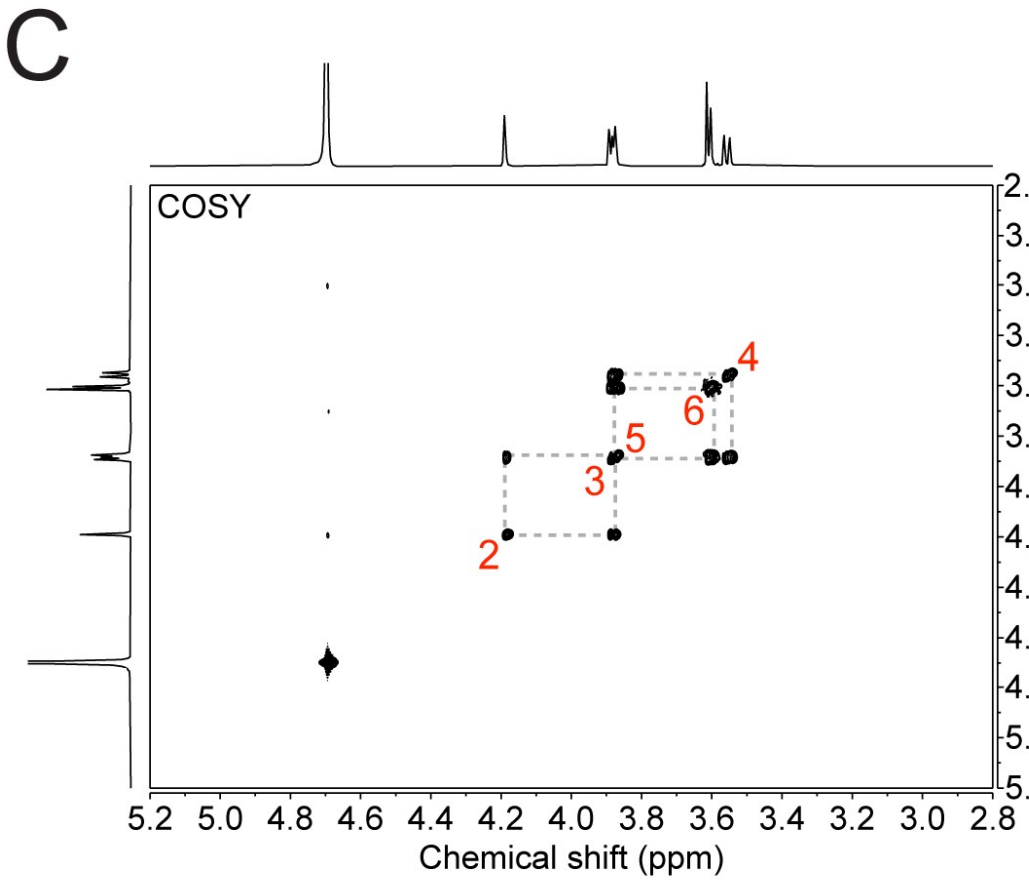

D

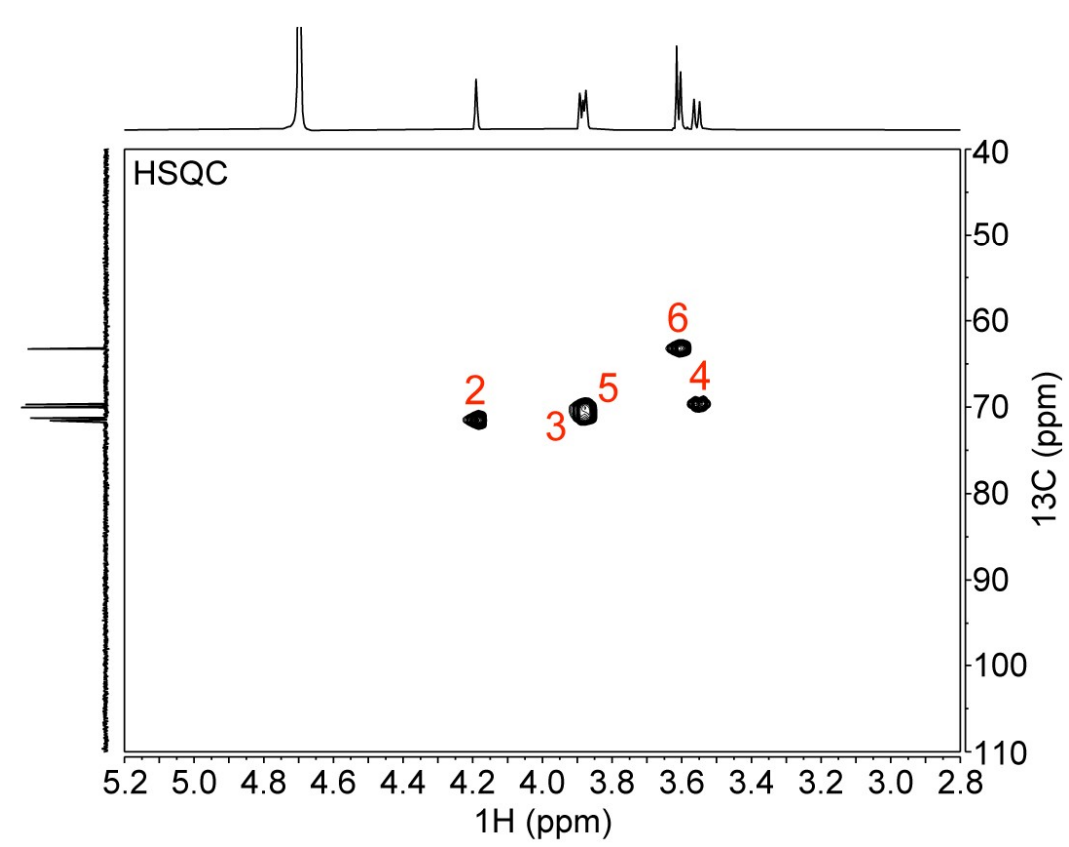

E

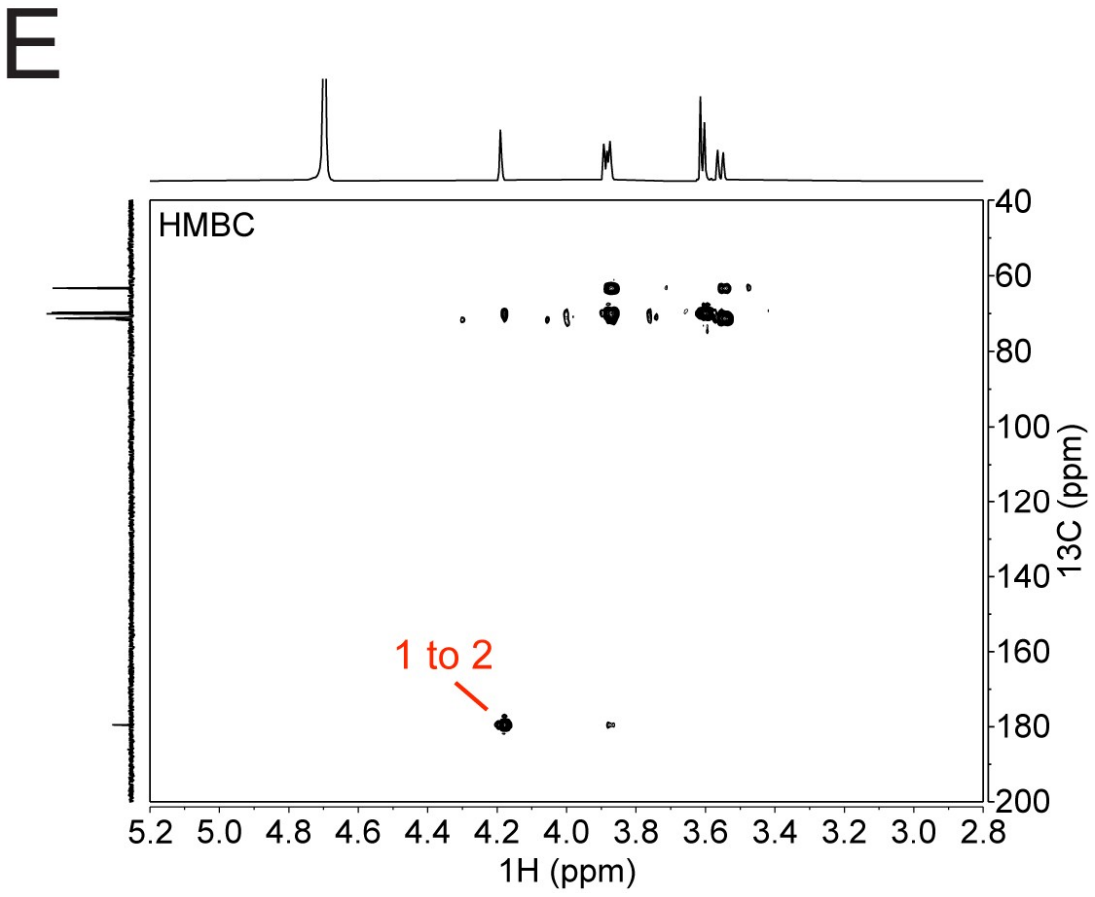

Supplementary Figure 22. NMR analysis of products from incubating NphT with D-ribose. In a Coy anaerobic chamber, the NPMN cofactor was synthesized *in vitro* as described in the methods and incubated with 40 μM NphT for 20 hours. A portion of the loaded protein was set aside for protein mass spectrometry to verify loading. NphT was exchanged into water using a PD-10 column, and fractions were combined and concentrated to 6.39 mg/mL using a 500 μL Modified PES VWR Centrifugal Filter, 10 kDa MWCO (VWR). Reactions (100 μL) containing holo (+NPMN) or apo (-NPMN) NphT (95 μM) were incubated with D-ribose (45 mM) in 20 mM HEPES pH 7.5 for 20 hours at 50°C. After drying down in a speed vacuum concentrator, the samples were resuspended in a final volume of 600 μL in D_2O before being transferred to a tube for NMR analysis. (A) 1-D overlaid ^{1}H NMR spectra. (B) COSY experiment. (C) HSQC experiment. (D) HMBC experiment. (E) Chemical shifts determined from the +NPMN 2D-NMR data. Spectral assignments for products are provided in list format in the NMR analysis of the standard supplemental figures below. -NPMN spectra are shown in black, and +NPMN spectra are shown in red. Residual water appears at ~4.7 ppm. Data are representative of n=3 experimental replicates.

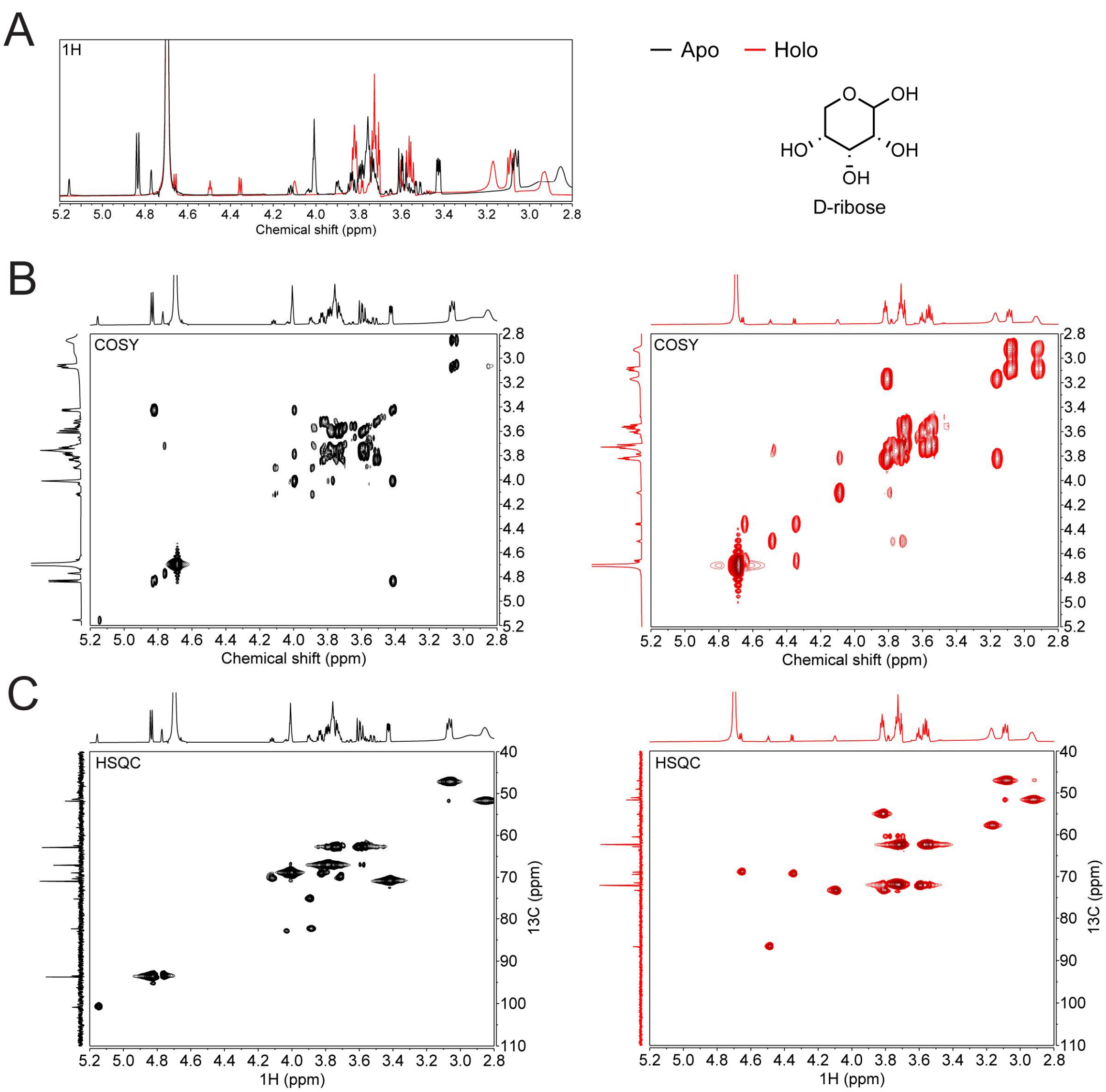

D

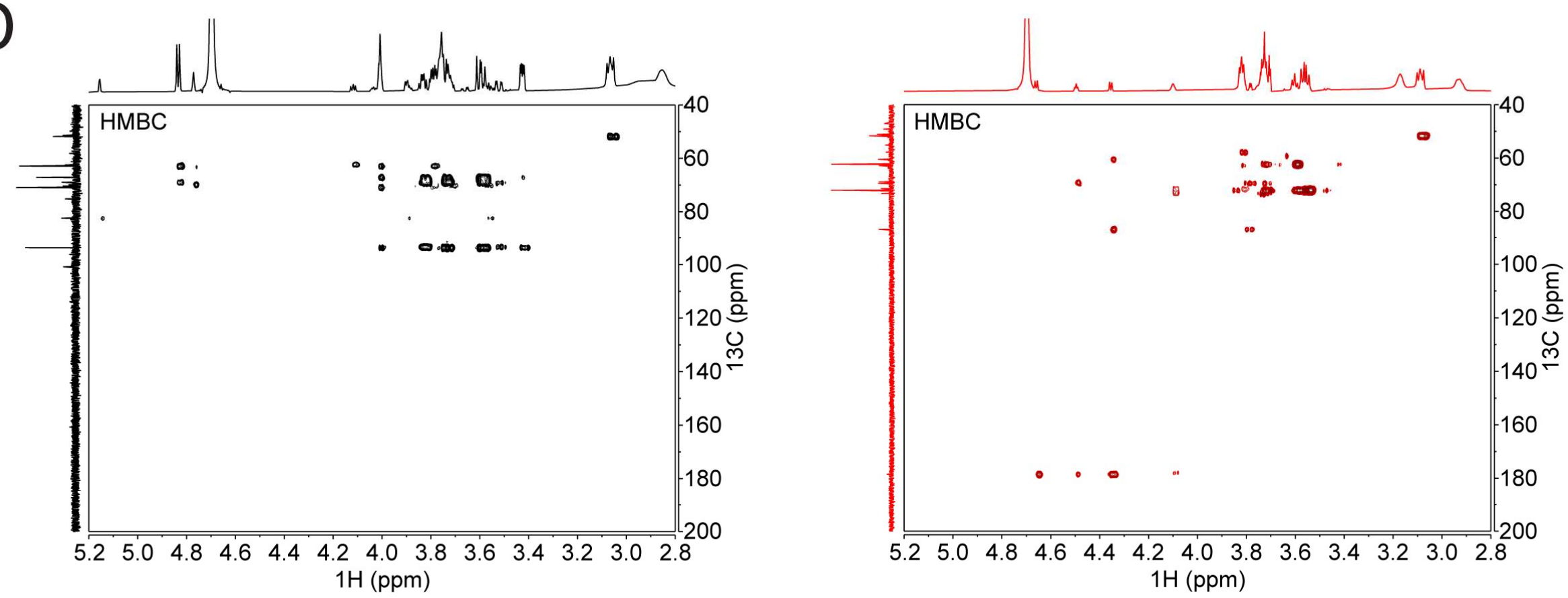

E

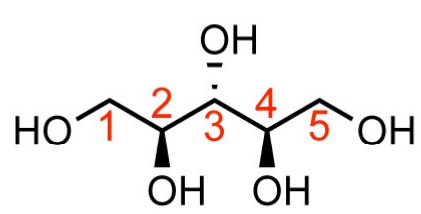

Ribitol

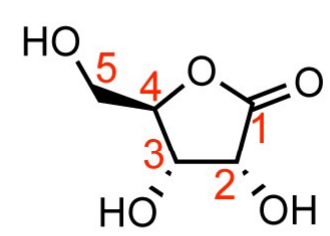

Ribono-1,4-lactone

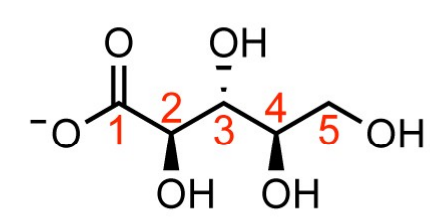

Ribonate

	1H (ppm)	13C (ppm)
1	3.71, dd	62.33
	3.56, dd	
2	3.73, m	72.06
3	3.60, t	72.16
4	3.73, m	72.06
5	3.71, dd	62.33
	3.56, dd	

	1H (ppm)	13C (ppm)
1	-	178.51
2	4.66, d	68.92
3	4.36, d	69.46
4	4.50, t	86.71
5	3.80, dd	60.50
	3.72, dd	

	1H (ppm)	13C (ppm)
1	-	178.06
2	4.08, d	73.47
3	3.81, dd	73.29
4	3.73, m	71.52
5	3.75, dd	62.84
	3.56, dd	

Supplementary Figure 23. NMR analysis of ribitol standard for comparison to NphT products. 5 mM D-ribitol was prepared in 600 µL of D_2O before transferring to a tube for NMR analysis. ^{1}H NMR (600 MHz, D_2O) δ 3.73 (m, 2H), 3.71 (dd, J = 7.3, 3.1 Hz, 2H), 3.60 (t, J = 6.3 Hz, 1H), 3.56 (dd, J = 12.0, 7.1 Hz, 2H). ^{13}C NMR (151 MHz, D_2O) δ 72.16, 72.06, 62.33. (A) Structure and table of NMR assignments. (B) ^{1}H and ^{13}C spectra. (C) COSY experiment. (D) HSQC experiment. (E) HMBC experiment. Residual water appears at ~4.7 ppm. These data were collected once.

A

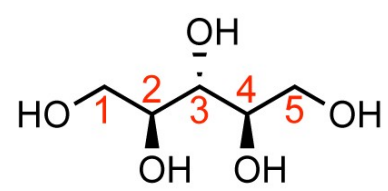

Ribitol

	1H (ppm)	13C (ppm)
1	3.71, dd<br>3.56, dd	62.33
2	3.73, m	72.06
3	3.60, t	72.16
4	3.73, m	72.06
5	3.71, dd<br>3.56, dd	62.33

B

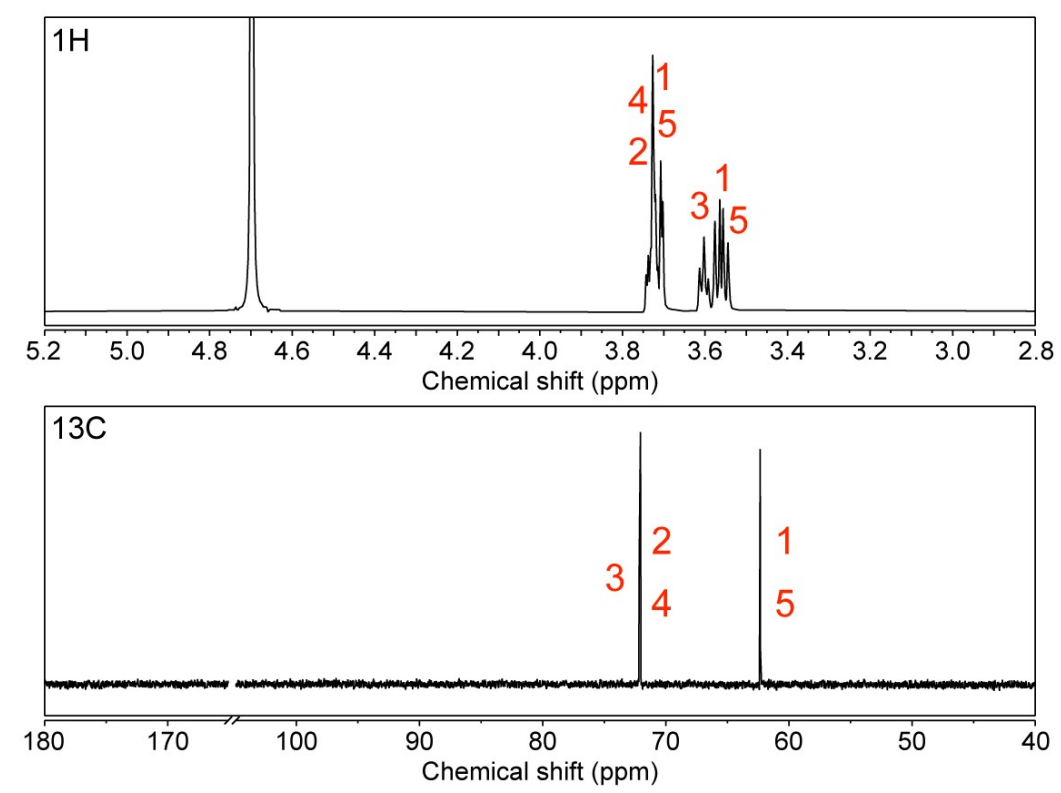

C

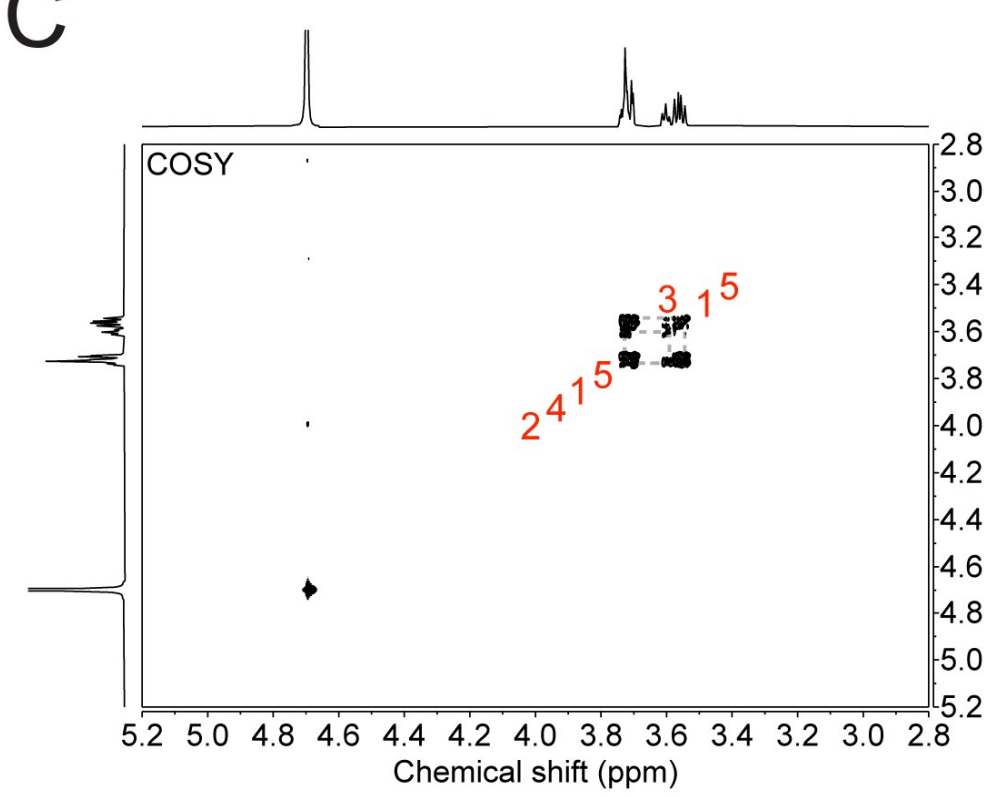

D

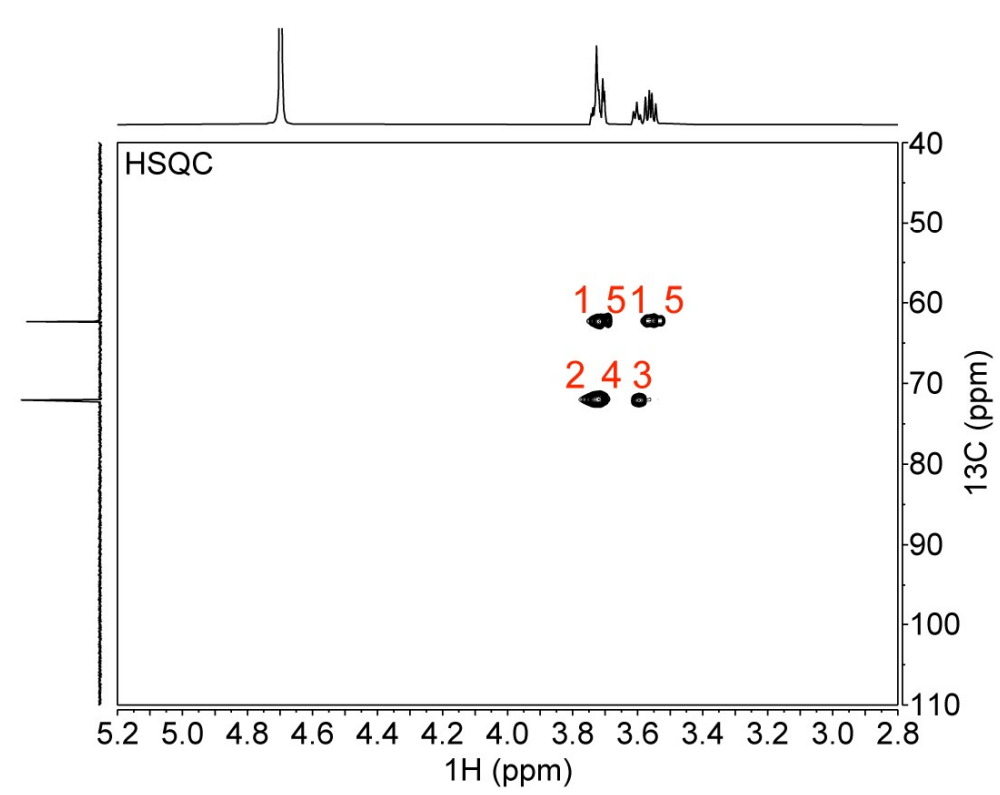

E

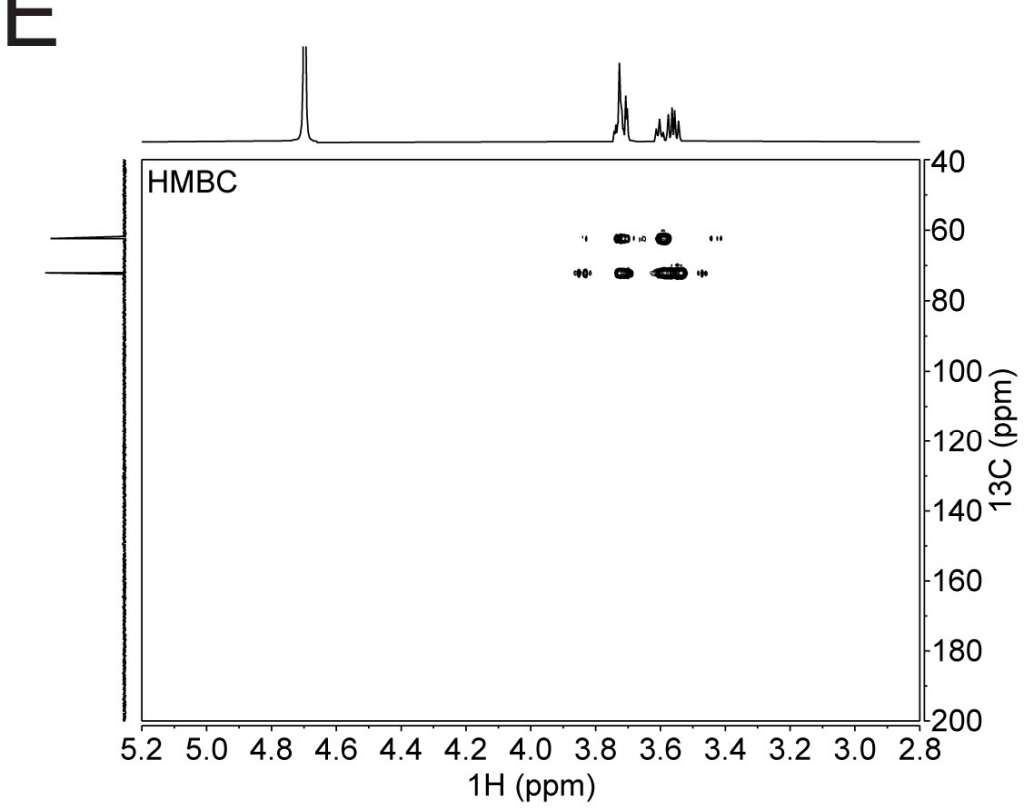

Supplementary Figure 24. NMR analysis of ribonolactone standard for comparison to NphT products. 5 mM D-ribono-1,4-lactone was prepared in 600 µL of D_2O before transferring to a tube for NMR analysis. (A) Structure and table of NMR assignments. ^{1}H NMR (600 MHz, D_2O) δ 4.66 (d, J = 5.6 Hz, 1H), 4.50 (t, J = 3.7 Hz, 1H), 4.36 (d, J = 5.6 Hz, 1H), 3.80 (dd, J = 13.1, 3.2 Hz, 1H), 3.72 (dd, J = 13.0, 4.2 Hz, 1H). ^{13}C NMR (151 MHz, D_2O) δ 178.51, 86.71, 69.46, 68.92, 60.50. (B) ^{1}H and ^{13}C spectra. (C) COSY experiment. (D) HSQC experiment. (E) HMBC experiment. Residual water appears at ~4.7 ppm. These data were collected once.

A

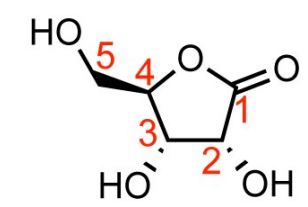

Ribono-1,4-lactone

	1H (ppm)	13C (ppm)
1	-	178.51
2	4.66, d	68.92
3	4.36, d	69.46
4	4.50, t	86.71
5	3.80, dd	60.50
	3.72, dd	

B

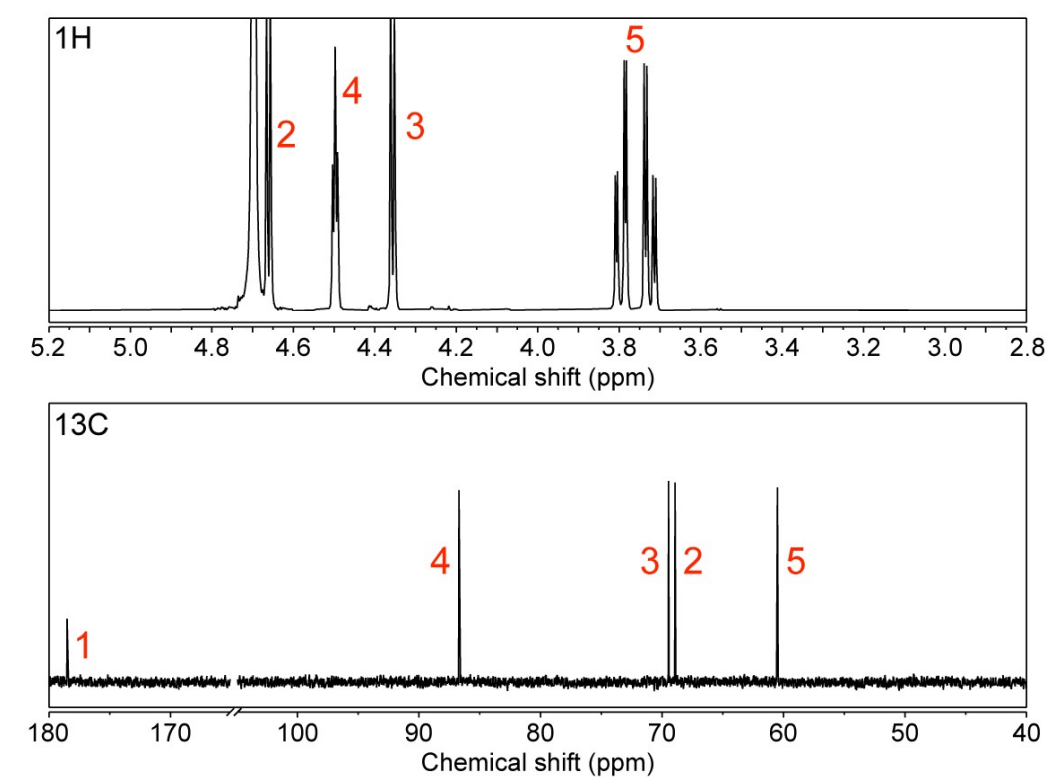

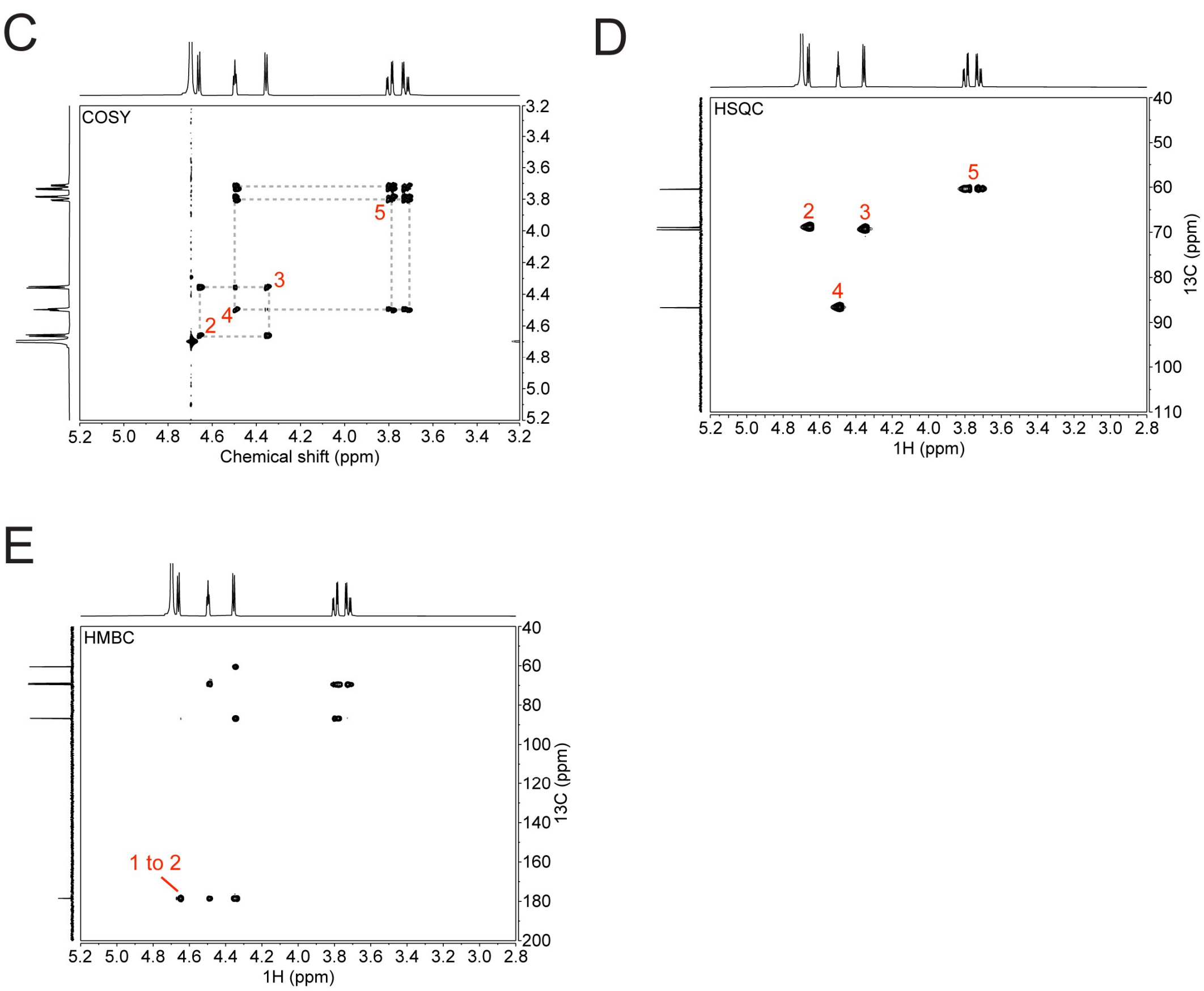

Supplementary Figure 25. NMR analysis of ribonate standard for comparison to NphT products. To make 5 mM D-ribonate, a 100 µL stock of 30 mM D-ribono-1,4-lactone containing 30 mM NaOH prepared from a 3 M stock in D_2O was incubated at 100°C for 1 hour. After cooling, the sample was brought to a final volume of 600 µL in D_2O and transferred to a tube for NMR analysis. Full conversion of ribono-1,4-lactone to ribonate did not occur, resulting in a mixture. In this figure, only the assignments for the ribonate signals are provided. (A) Structure and table of NMR assignments. ^{1}H NMR (600 MHz, D_2O) δ 4.08 (dd, J = 5.8, 1.6 Hz, 1H), 3.81 (dd, J = 6.9, 3.4 Hz, 1H), 3.75 (dd, J = 6.8, 3.2 Hz, 1H), 3.73 (m, 1H), 3.56 (dd, J = 12.0, 6.8 Hz, 1H). ^{13}C NMR (151 MHz, D_2O) δ 178.06, 73.47, 73.29, 71.52, 62.84. (B) ^{1}H and ^{13}C spectra. (C) COSY experiment. (D) HSQC experiment. (E) HMBC experiment. Residual water appears at ~4.7 ppm. These data were collected once.

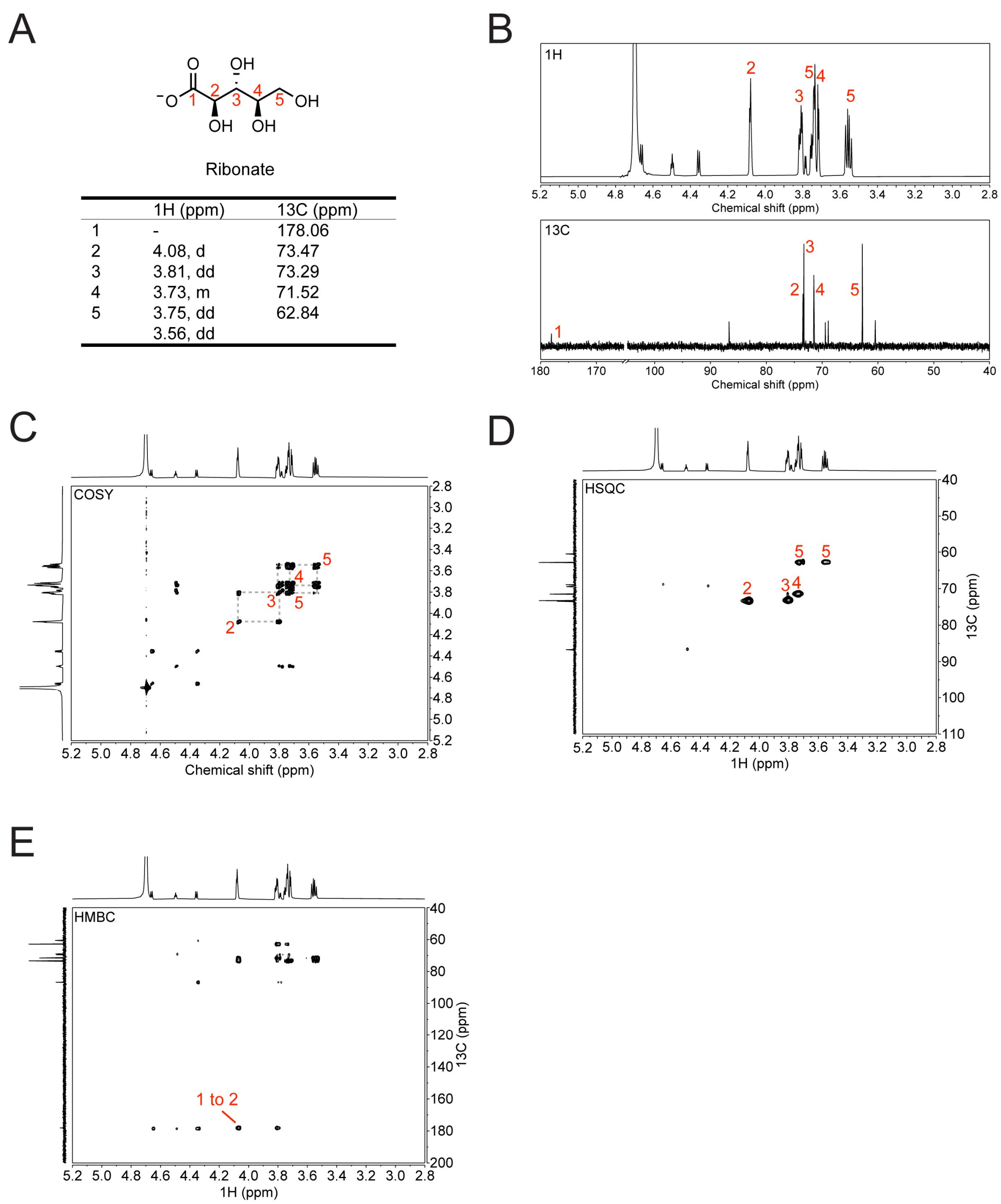

	1H (ppm)	13C (ppm)
1	-	178.06
2	4.08, d	73.47
3	3.81, dd	73.29
4	3.73, m	71.52
5	3.75, dd	62.84
	3.56, dd	

Supplementary Figure 26. Assessment of NphT total turnover number. In a Coy anaerobic chamber, the NPMN cofactor was synthesized *in vitro* as described in the methods and incubated with 40 μM NphT for 20 hours. A portion of the loaded protein was set aside for protein mass spectrometry to verify loading. NphT was exchanged into water using a PD-10 column, and fractions were combined and concentrated to 6.39 mg/mL using a 500 μL Modified PES VWR Centrifugal Filter, 10 kDa MWCO (VWR). Reactions (100 μL) containing holo (+NPMN) or apo (-NPMN) NphT (15 μM) were incubated with D-ribose (50 mM) in 50 mM HEPES pH 7.5 for 20 hours at 50°C. After drying down in a speed vacuum concentrator, the samples were resuspended in a final volume of 600 μL in D_2O before being transferred to a tube for NMR analysis with 10 mM TSP added as a reference. 1D ^1H spectra at 298 K were recorded using the Bruker pulse program zg30 with a spectral width of 20 ppm, centered at 6.175 ppm, an acquisition time of 2.7 s (600MHz), relaxation delay of 1 s, and 8 scans. The ratio of products in each reaction was assessed by utilizing global ^1H peak integration of the α- and β- anomeric protons of the furanose and pyranose forms of D-ribose in Mnova. In this case, the reaction went to completion. Final turnover number was assessed to be approximately 1666, given that a single catalytic cycle consumes two substrate molecules. This experiment was performed once.

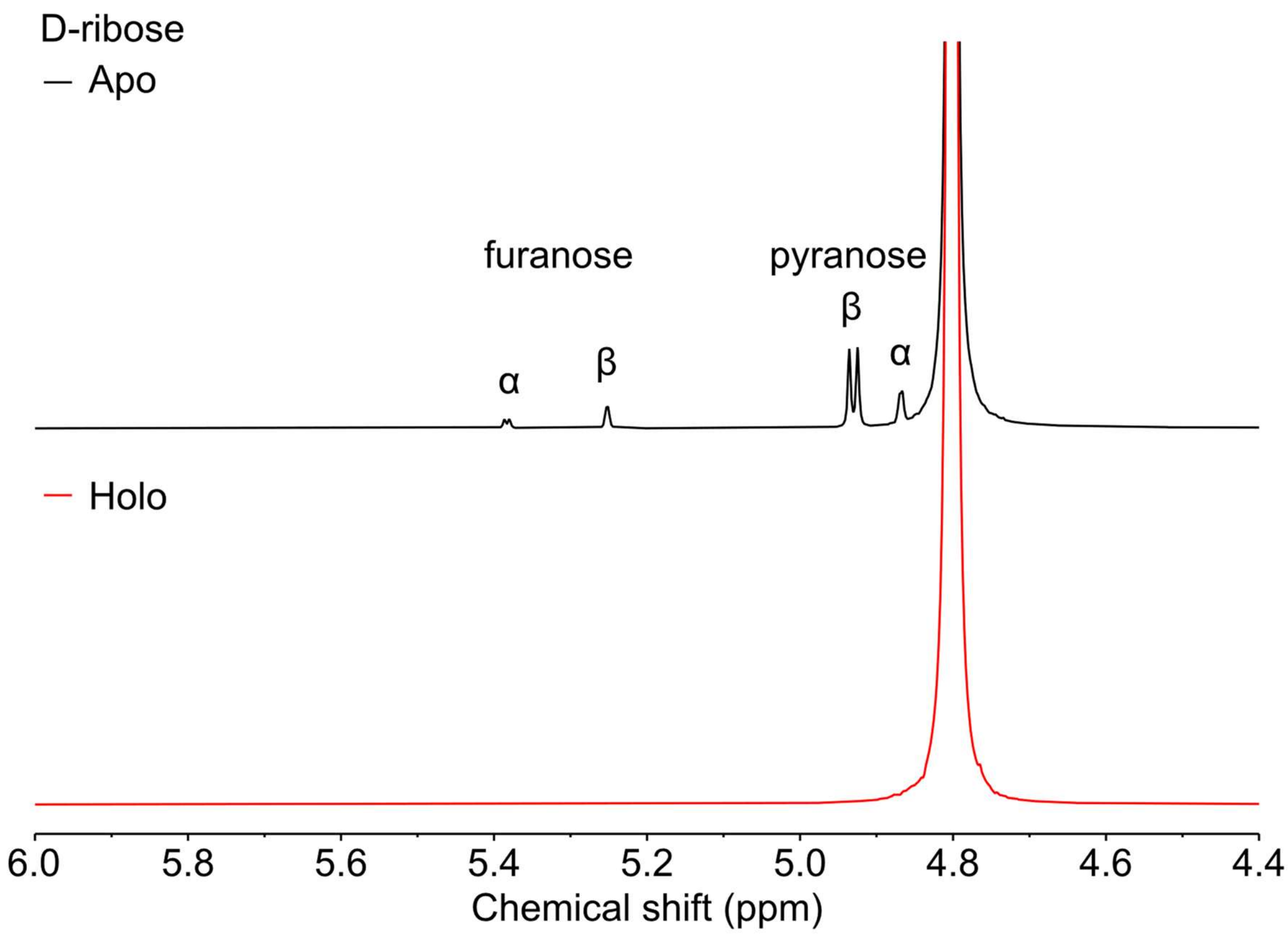

Supplementary Figure 27. Product distribution over time. (A) Reactions (40 µL) contained 10 mM substrate and 5 µM enzyme in 25 mM HEPES pH 7.5 at 50°C. At 2 hours and at 25 hours, 10 µL of the reaction was removed from the chamber and quenched in 70 µL of 6:1 acetonitrile:water and freeze-thawed on dry ice for a total of 3 times. After the final thaw, 10 µL of methanol was added. The sample was briefly vortexed and then centrifuged for 10 minutes at 13000 *x g* before transfer of the supernatant into an HPLC vial for LC-MS analysis. Extracted ion counts ($[M-H]^-$) were recorded for arabitol (151.0611), galactitol (181.0717), and ribitol (151.0611) and plotted relative to their values at 2 hours (left). Extracted ion counts ($[M-H]^-$) were recorded for arabinolactone (147.0298), galactonolactone (177.0404), and ribonolactone (147.0298) and plotted relative to their values at 2 hours (middle). Extracted ion counts ($[M-H]^-$) were recorded for arabonate (165.0404), galactonate (195.0510), and ribonate (165.0404) and plotted relative to their values at 2 hours (right). Over time, the lactone product is hydrolyzed to the hydroxyacid products. The bar graph represents a single (n=1) value which is representative of n=2 experimental replicates. (B) Accompanying schematic of proposed enzymatic reaction and products. Although it is not known if the enzyme directly oxidizes the pyranose, furanose, or aldose forms of the sugars, the schematic is drawn to represent a possible path to the observed lactone, alcohol, and hydroxyacid products.

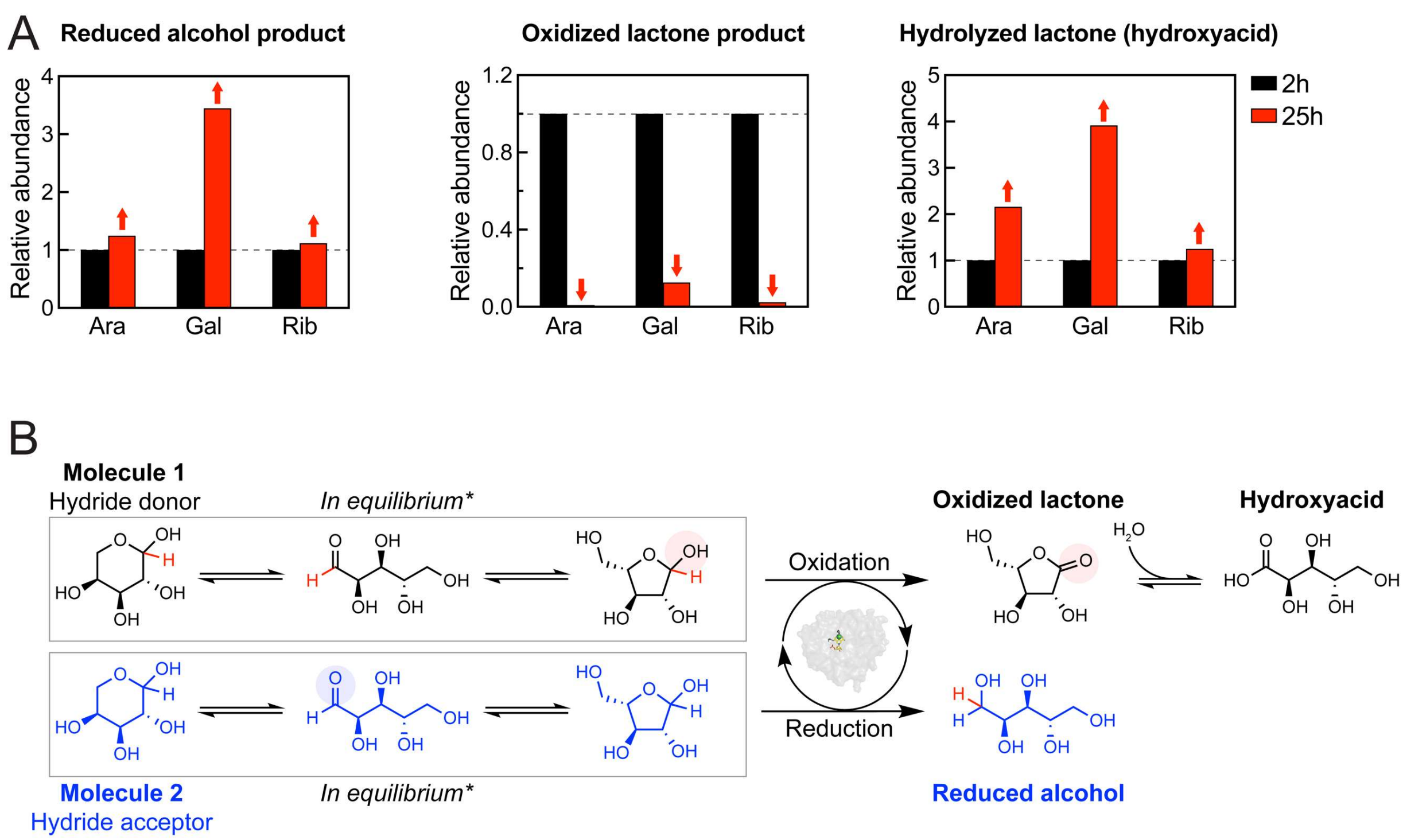

Supplementary Figure 28. Quantitative ^{13}C NMR analysis of products from incubating NphT with L-arabinose, D-galactose, or D-ribose. In a Coy anaerobic chamber, the NPMN cofactor was synthesized *in vitro* as described in the methods and incubated with 40 µM NphT for 20 hours. A portion of the loaded protein was set aside for protein mass spectrometry to verify loading. NphT was exchanged into water using a PD-10 column, and fractions were combined and concentrated to 6.39 mg/mL using a 500 µL Modified PES VWR Centrifugal Filter, 10 kDa MWCO (VWR). Reactions (100 µL) containing holo (+NPMN) or apo (-NPMN) NphT (95 µM) were incubated with sugar substrate (45 mM) in 20 mM HEPES pH 7.5 for 20 hours at 50°C. After drying down in a speed vacuum concentrator, the samples were resuspended in a final volume of 600 µL in D_2O before being transferred to a tube for NMR analysis. Quantitative ^{13}C NMR spectroscopy was performed with 4096 scans and a d1 of 14 s. The ratio of products in each reaction was assessed by utilizing ^{13}C peak integration in Mnova. This experiment was performed once, and integration was performed in triplicate. The data are the average of n=3 integration replicates ± s.d.

Products from L-arabinose substrate

Compound	mol % by ^{13}C NMR
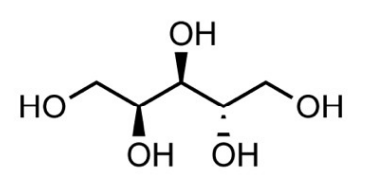 Arabitol	50.12 ± 1.36
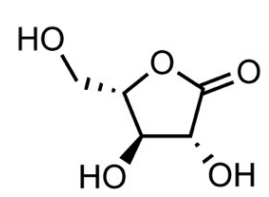 Arabino-1,4-lactone	20.62 ± 0.44
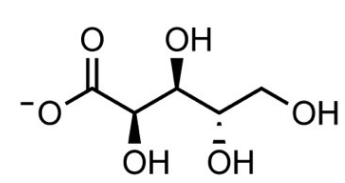 Arabonate	29.26 ± 0.92

Products from D-galactose substrate

Compound	mol % by ^{13}C NMR
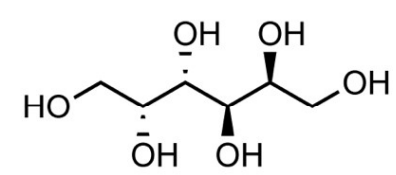 Galactitol	48.76 ± 1.57
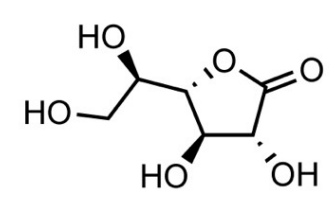 Galactono-1,4-lactone	16.64 ± 1.05
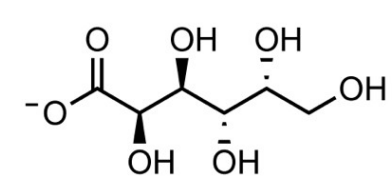 Galactonate	34.60 ± 1.16

Products from D-ribose substrate

Compound	mol % by ^{13}C NMR
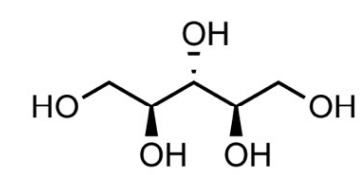 Ribitol	51.65 ± 0.93
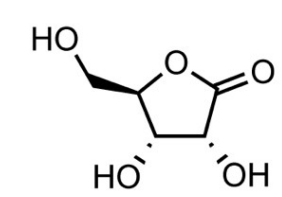 Ribono-1,4-lactone	16.14 ± 0.29
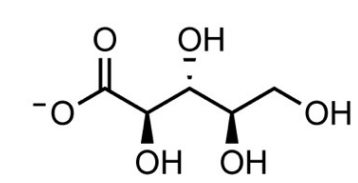 Ribonate	32.21 ± 0.66

Supplementary Figure 29. LC-MS comparison of products to synthetic standards. Reactions (40 µL) contained 10 mM substrate and 5 µM enzyme in 25 mM HEPES pH 7.5 at 50°C. NPMN without enzyme was included as a negative control. Reactions were allowed to proceed for 5 hours (arabinose), 4 hours (galactose), or 2 hours (ribose) at 50°C. These times were selected to optimize the detection of the lactone species before hydrolysis to the carboxylate species. 10 µL of the reaction was removed from the chamber and quenched in 70 µL of 6:1 acetonitrile:water and freeze-thawed on dry ice for a total of 3 times. After the final thaw, 10 µL of methanol was added and the sample was briefly vortexed and then centrifuged for 10 minutes at 13000 x *g* before transfer of the supernatant into an HPLC vial for analysis on a Waters HILIC amide column as described in the methods. HRMS (*m/z*) $[M-H]^-$ for: arabitol (calcd., 151.0611; found, 151.0611); arabinolactone (calcd., 147.0298; found, 147.0298); arabonate (calcd., 165.0404; found, 165.0404); galactitol (calcd., 181.0717; found, 181.0717); galactonolactone (calcd., 177.0404; found, 177.0404); galactonate (calcd., 195.0510; found, 195.0510); ribitol (calcd., 151.0611; found, 151.0611); ribonolactone (calcd., 147.0298; found, 147.0298); ribonate (calcd., 165.0404; found, 165.0404). Data are representative of n=2 technical replicates.

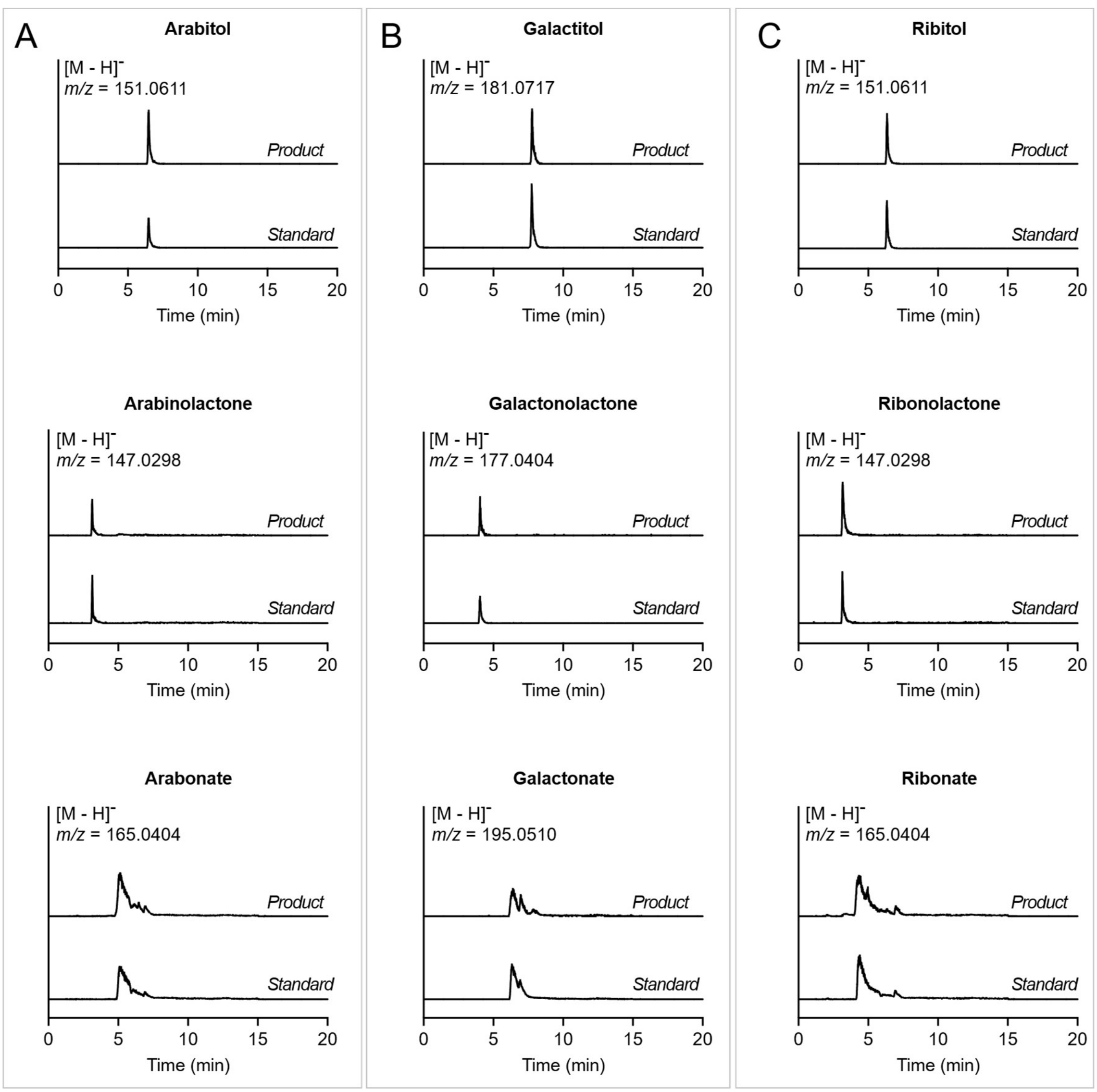

Supplementary Figure 30. LC-MS data for LarA and NphT at 37°C and 50°C. In a Coy anaerobic chamber, *in vitro* cofactor synthesis was performed as previously described. The NPMN cofactor or control with LarC and E omitted from the reaction was incubated with 20 µM NphT or LarA for 20 hours. A portion of the loaded protein was set aside for protein mass spectrometry to verify loading. Reactions (30 µL) contained 10 mM substrate (L-arabinose or D-galactose) in 3.33 mM HEPES (pH 7.5). Reactions were initiated by the addition of enzyme (1.66 µM final concentration) and allowed to proceed for 20 hours at either 50°C or 37°C. For the ribose reactions, conditions were optimized to increase the yield of the lactone product before hydrolysis. The NPMN cofactor reaction was incubated with 40 µM NphT or LarA for 20 hours. Reactions (40 µL) contained 10 mM D-ribose and 5 µM enzyme in 25 mM HEPES pH 7.5 and proceeded for 2 hours at 50°C or 37°C. 15 µL of the reaction was removed from the chamber and quenched with 45 µL of 100% LC-MS-grade acetonitrile. Samples were freeze-thawed on dry ice for a total of 3 times. 10 µL of methanol was then added and the sample was briefly vortexed before centrifugation for 10 minutes at 13000 x *g*. The supernatant was transferred into an HPLC vial and analyzed using a HILIC column (Waters) as described in the methods. (A) Extracted ion chromatograms of sugar alcohol and lactone products from NphT and LarA activity on L-arabinose, D-galactose, and D-ribose at 37°C. (B) Extracted ion chromatograms of hydrolyzed lactone product for reactions run at 37°C and 50°C. Data are representative of n = 3 experimental replicates.

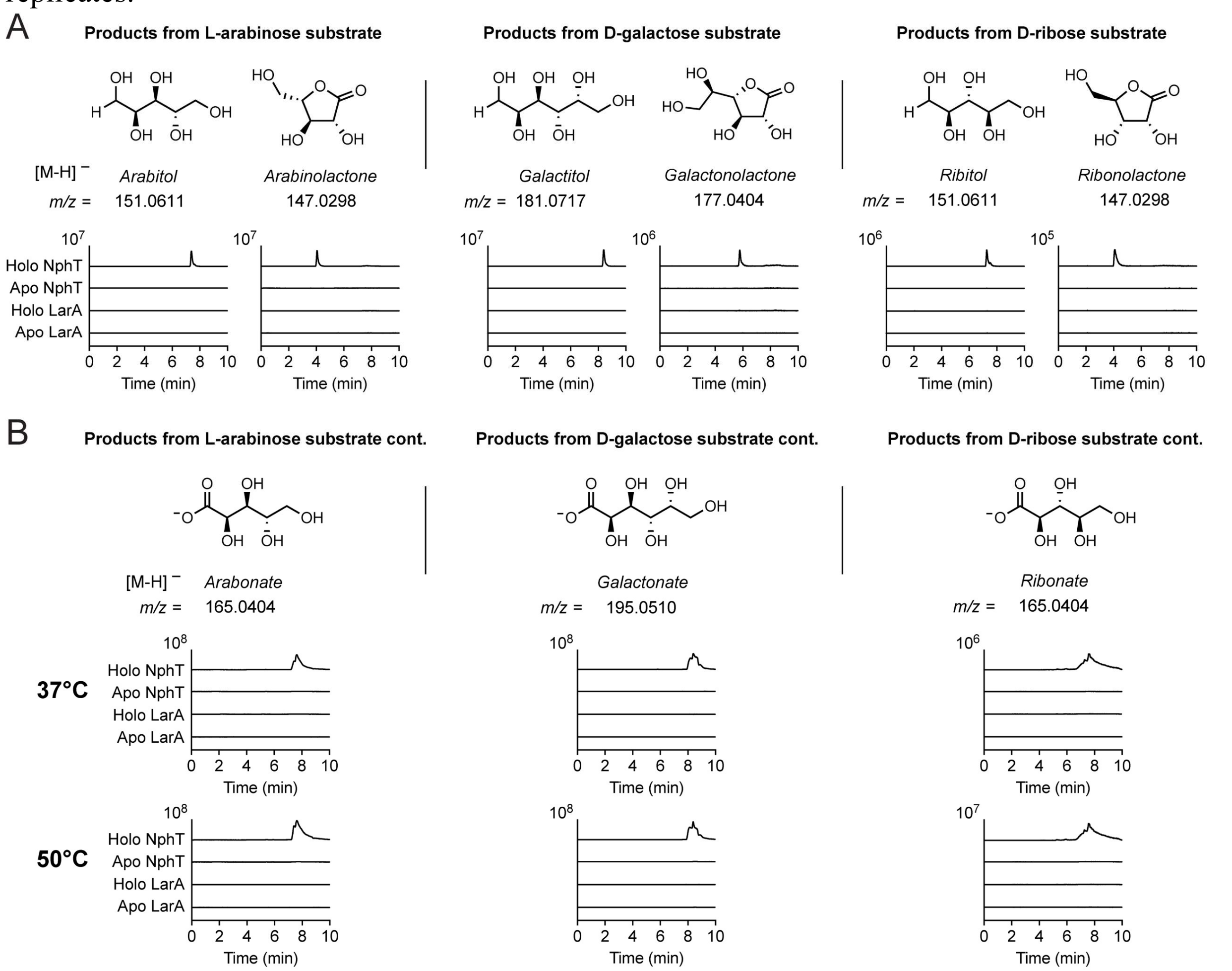

Supplementary Figure 31. LC-MS analysis of holo NphT prior to crystallography. In an anaerobic chamber (Coy), the NPMN cofactor was incubated with 40 µM NphT in a final volume of 685 µL for 18 hours. The following day, the sample was centrifuged at 13,000 x *g* for 5 min to remove precipitate. The remaining soluble holo protein was concentrated with a Pall Nanosep 10k centrifugal filter to a volume of 400 µL and loaded onto a Superdex™ 75 Increase 10/300 GL column (Cytiva) equilibrated with SEC buffer (20 mM HEPES, 100 mM NaCl) and eluted at a flow rate of 0.4 mL/min. Fractions containing NphT were pale orange. NphT samples were combined and concentrated to 10 mg/mL using a 0.5 mL Amicon Ultra Centrifugal Filter, 10 kDa MWCO (Millipore). The protein was diluted 500-fold in LC/MS grade water and then injected on an Agilent LC/MS for analysis using the column and acquisition parameters detailed in the protein LC/MS methods. **Expected (apo)**: 32,559.7 Da; **Expected (holo)**: 33,009.6 Da; **Observed**: 33,010.4 Da. Delta = 450.7 (NPMN). This experiment was performed once.

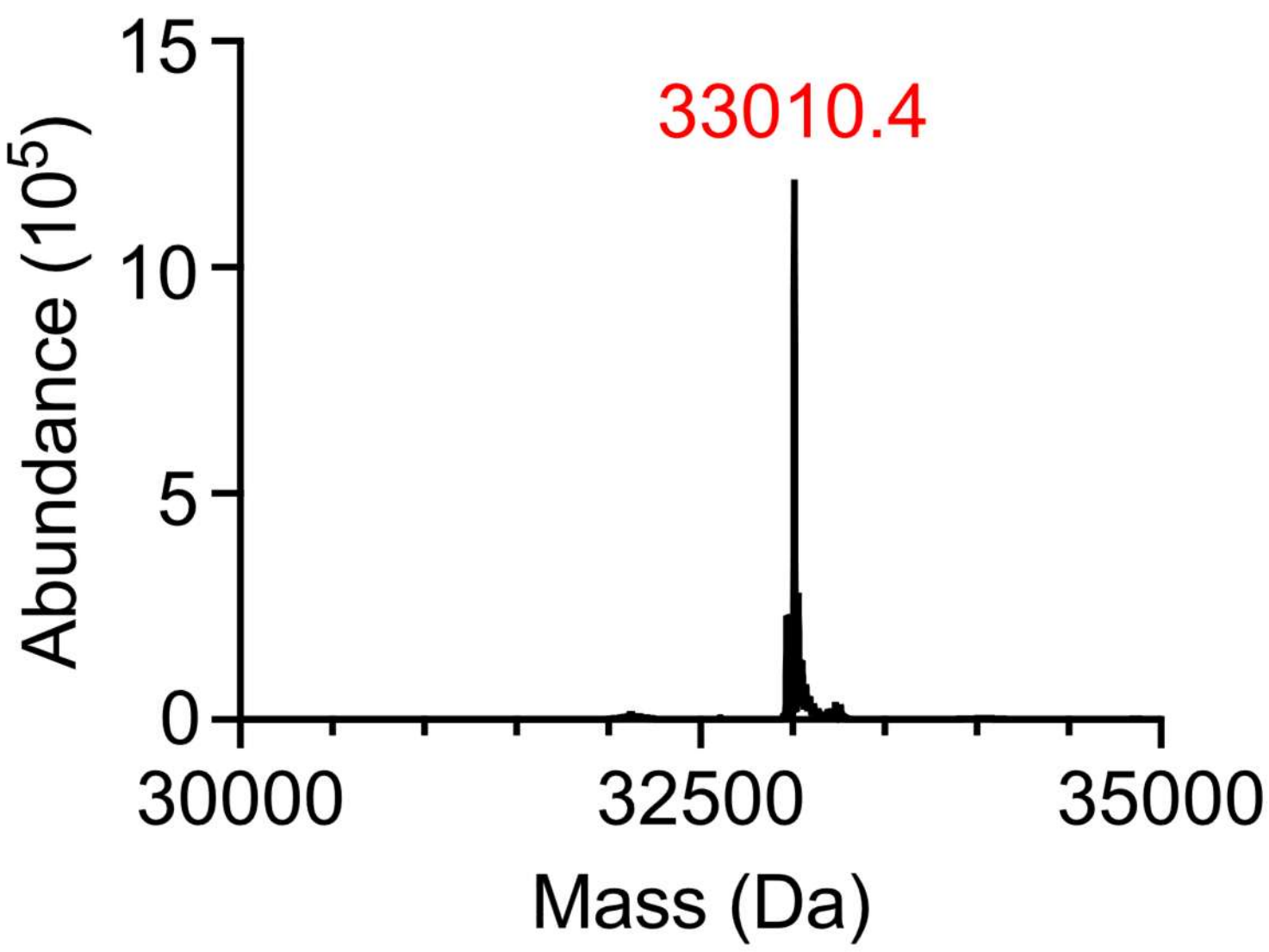

Supplementary Figure 32. Nickel anomalous difference map. (A) Nickel anomalous difference map is shown as a green mesh and contoured to 5σ. Data were collected at ALS BL831 at a wavelength of 1.48 Å. This experiment was performed once.

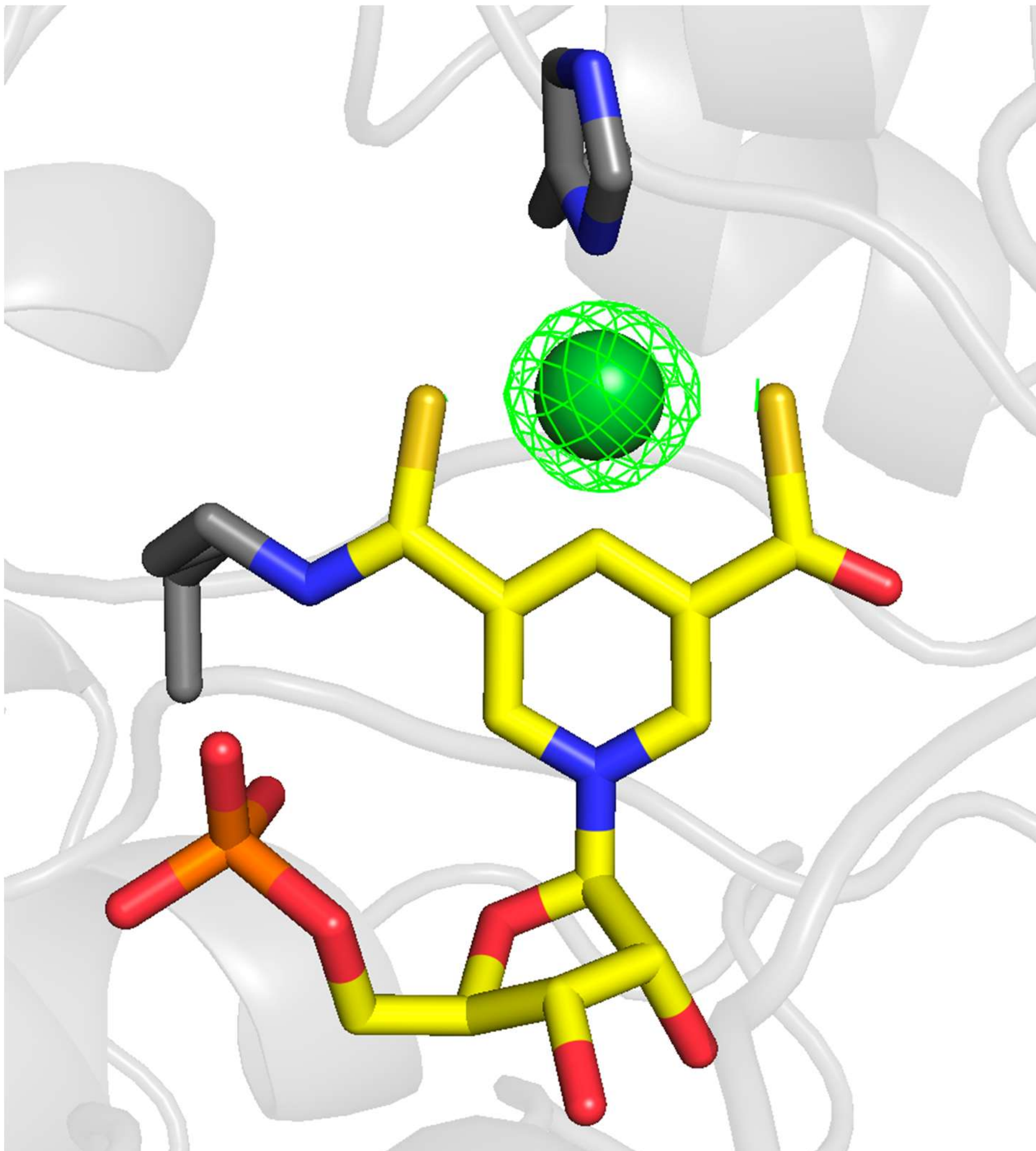

Supplementary Figure 33. Residues contacting the NPMN cofactor. Two views of the NPMN-contacting residues are shown for (A, B) NphT PDB 9YEB, (C, D) LpLarA PDB 5HUQ, and (E, F) SpLarA PDB 9Q3K.

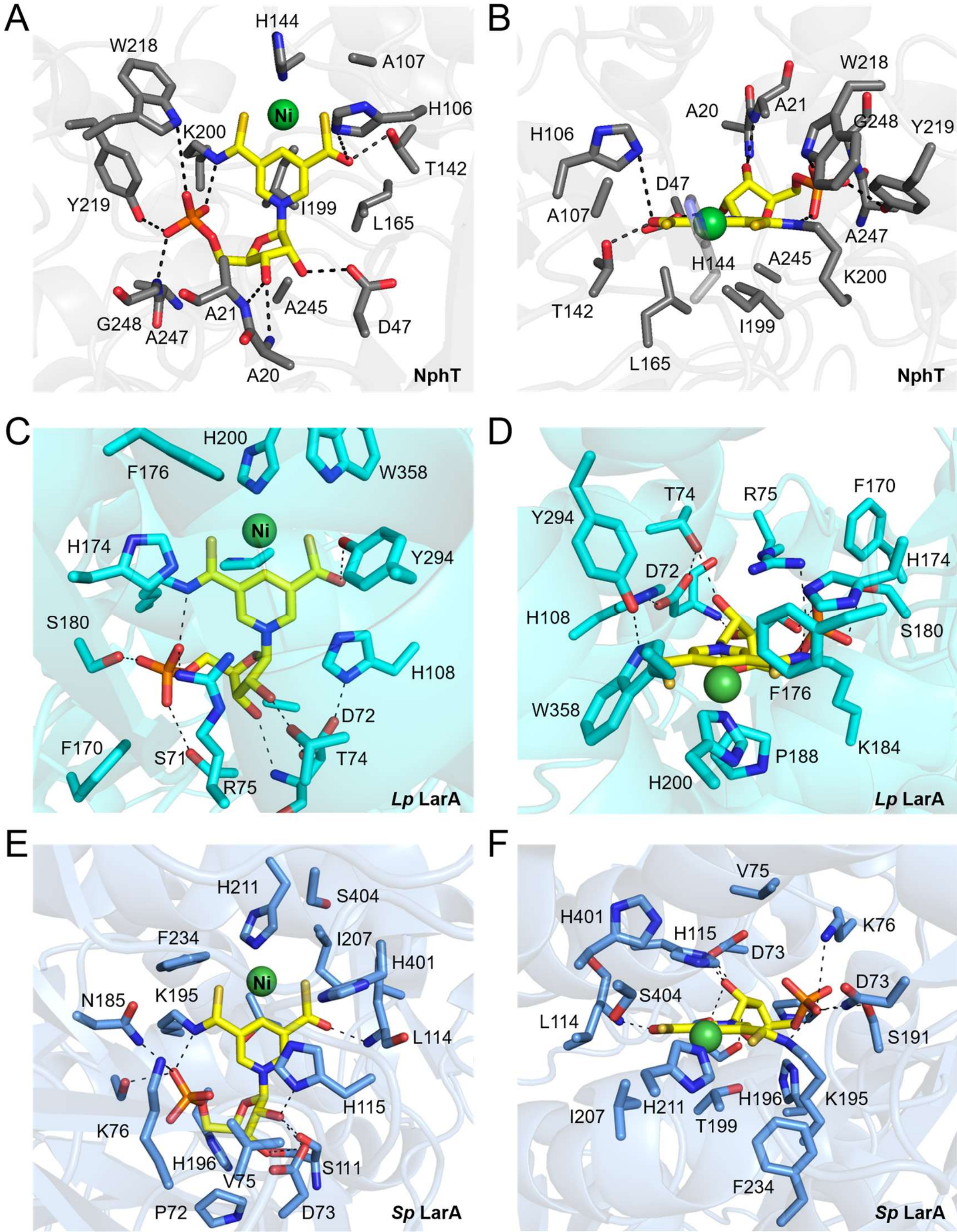

Supplementary Figure 34. Alignment of AKRs in the same SSN cluster as NphT. The sequences for the representative enzymes in the cluster containing NphT from the SSN in **Figure 6** were aligned using Clustal Omega in the Clustal format. Residues contacting the NPMN cofactor in the NphT crystal structure are boxed in grey, and those in orange are backbone contacts. Residues matching H144 and K200 of NphT are highlighted in red. Sequence numbering is based on NphT.

```
                       1         10        20         30        40        50          60         70
NphT        -----------METRRLGRIGHMSSVIIFGAASLSDVSQE-EADASIQFALDSGINHFDTAASYGE--SELRLGPWMPKIRN-KIFLATK
A0A0D8HLA9  ----------MIPQLQFGKTGHMTSRVIFGAAGLGESSQA-TADATLEVILAAGINHIDTASSYGN--SEERIAPWMKDHRN-KFFLATK
A0A0M0BQ91  -----------METRTLGRTGHRSTLITLGGAAARPTTRK-AFDTFAKMALDHGVNHVDVAPTYGSGMAERILGPWVKEYRN-TLFLACK
A0A0M2PNZ0  -----------MQKRKIGRLSYEGSVVMFGAASLGNVTQE-VADQSISFALEHGVNHFDTGADYGD--AELRLASIISKVRN-DIFLATK
A0A0S8CFH0  -----------MEKRRLGRTGHSSSVVTFGSAALWNASQA-EADAAIELALEHGINHFDVSPVYGK--AEKLLGPWMEKHHK-EIFLACK
A0A133UF86  ----------MIEKMNFGRSDHESTKAIFGGAALYQSPRE-EADRALEILLDYGVNHIDTSVSYGE--SEKHIGKWMKEYRD-EFFLATK
A0A151BQE0  -----------MERRRLGRTGQMSSVVAFGCAALGRVSQE-EADAAIELALEHGVNHFDVAPSYGE--AELRLRPWLKDHRD-EIFLACK
A0A1F5DHV4  ----------MIPKRRLGRTGAKVSVITLGGCGVGRLTQE-EADVHIKAAFDAGVNMLDLAPTYGD--SELRLAPWMRTHRG-RVFLAEK
A0A1F8NH80  -----------METRPLGRTGHKSSVAVFGAASLSEVTQA-DADAGLDLALSRGVNHIDVAPSYGG--AEERLAPWLERHRS-SVFLACK
A0A1M3K092  -----------MEKRTFGRTGHQSTVAIFGAAAFWDITQR-LTDTVLNQVLNAGVNHFDVAPSYGV--AEERIAPWMPRIRQ-QIFLGCK
A0A1Q9P4Y9  ----------MIPKIQFGRTGHSSSRIIFGAYALSNATQE-EADEVLKVLLEYGINHIDTARIYGK--SEERIGPWMERHRD-DFFLATK
A0A1V5RY86  ----------MIEKRVFGRTGHLSTRVIFGAAAFSDVTQA-EADRTIDLLMQYGINHFDTAASYGK--SEVLLGPWVKHNRD-KIFLATK
A0A2E3KCK0  ---------------------MSSAVIFGAAALGRVDQY-TADKTLDLLLEYDVNHLDTAPAYGD--AEIRMGPWMDKYRD-NFFLATK
A0A351G6T0  ----------MIARKPFGRTGHTSSRTLFGAVALSGLSRA-EVDRTLELLLGYGVNHIDTAASYGD--SELQLAPWMQHHRR-QFFLATK
A0A363SWS1  ----------MIPKAAFGRTGHISSRVIFGSWALSTASQK-EADQVVELLMEYGINHIDTAPMYGN--AEKLIGTWMKKHRQ-AFFLATK
A0A540VJS1  ----------MIRKQAFGRTGHMSTVTIFGAAALSRVTQE-EADRTLEVLLEYGVNHIDTAASYGD--AELRIGPWMKEHRK-TFFLATK
A0A6B0YYY2  -----------MQKRTLGRTGHDSTVVSFGTYAVGYVDQD-AADRAIQLVLDAGVNHFDIAPTYGH--AMERVAPWIPEIRKHGIFVGAK
A0A6J4PGY2  -----------MEHRRLGRLGRENGVLIFGGAALGEATEE-AADTAIQQALDAGVDHFDTAADYGD--SELHYGRWMPEIRD-RIFISTK
A0A6J4Y308  --------MASIGKQPFGGTGNLSTRAIFGSVCLSRADQD-AADGILEILLDYGINHIDTAPSYGH--AEIRVGHWMKQYRE-QFFLATK
A0A7C7U6Y6  ----------MIEKNLFGSTKHSSSRILFGAAALGAMRQD-RADQVLELLLEYGVNHIDTAAGYGD--SELRVGSWMAEHRK-NFFLATK
A0A7J3I994  MYRKGCGEVFILEYRRLGRTNFKVSVLAIGGYGPGVHPDPQLAIKVVEEVIGEGINIIDIAPSYAE--AETRLGSLIKKYRD-RLVVAEK
A0A7V1H5U5  -----------MEKRRLGRTEHMSSVITFGAAVFVKKKQR-EADRVLEILLERGVNQIDVAPEYEP--AEKRVGKWMKKHRK-LFFLNCK
A0A7W0SB34  -----------METRRLGKTGHPSSVITFGAFAIGHVPQA-EADRAIEWALANGINHFDVAPSYAD--AELRLGDYLGRHPQPGLFISCK
A0A842U755  -----------METRMFGRTGSEVSIVALGGCGPGYVEQE-EADEAVRSALDAGVNMIDIAPTYGK--AENRLKHWIQKDRN-QFFIAEK
A0A846PG70  ----------MIQRQEFGRTGHQSTRVIFGSWALNKATQA-EADSTLALLREYGVNHIDTAPMYGN--AEKVIGPWLAKHRD-EFFVATK
A0A8J3QVV5  -----------MERRALGRTGHESSVAILGGAAFWETTVD-VVAAAFERAVAAGVNHLDIAPQYGM--AEDLAGEVLPQYRD-GMFVACK
A0A8T4Z565  -----------MEKRKLGRTGHWISVMAFGGAALSKVSQS-VADGAVRLAMDHGVNHFDIAPSYGN--AELRLRPWMKDYRS-HIFLASK
A0A8T5TE33  -----------METRVFGKTNANITLITMGGCGLGYVDQN-EADKAVKLAMDHGINMIDVAPTYGK--AEERLRPWIQKYRD-KFFLAEK
A0A916CPH4  ----------MMEQRRFGRTGHASSVAILGAAAFGKITQA-EADSAIENAIALGVNHIDVAPSYGD--AEARLAPWMPRLRK-RMFIGCK
A0A920D8J6  -------MNERMETRRLGRTDHHSSVVIYGAASLGDVTQE-EADASIQYALDAGINHFDTASSYGD--SELRLGPWMGRIRN-RIFLATK
A0A934XWN3  ------------------------------------------------------------------------------------------
A0A9E4BYZ3  -----------MEKRTLGRTGHQSTVATFGAFSVGYVDQD-EADCAIQLILDHGVNHIDIAPSYGH--AMERVAPWMPDIRD-KMFLGSK
A0AA35T6J4  ---------------------------------------------------------------------MERMAPWMPKLRD-SMFLGAK
```

```
                  80        90        100       110       120       130       140       150         160
NphT        TGERTRDEAMRSIERSLKRLQVDSVDLMQLHAVTSFEELDQCTRKGGALEAAIAAKEQGMVKHIGITGHGHLAPKVHLEALRRY-PFETV
A0A0D8HLA9  TGDRTGDGARASIERSLVRLGVDQIDLIQLHNLVEEDEWKIAHSKGGALEALMDARQQGLVKYIGVTGHGTRIPSMHIRSLSAY-DYDSI
A0A0M0BQ91  TQKRTKQAAAEELRASLKNLQTDVIDLYQLHGLDTLEELRTALGEDGAIQAFLEAQEQDLIRYIGITSHN---PVTIRAALERF-DFATV
A0A0M2PNZ0  TGQRTKEKAKAEIYRSLERMKVDSVDLLQLHAVGTIEELDKCTAKGGALEAAIEAKEEGIVKAIGITGHGHQAPVTHLEALKRF-PFATV
A0A0S8CFH0  TWDRSKNKAWESIKRSLESLRVDHFDLFQLHRVNDIETLNVILGHGGALEAVLEAKEKGLVRHIGITGHR---PFVQVEGLNRF-DFDTV
A0A133UF86  VDSRTYKEGKKEIQKSLENLNVDQFDLLQMHELVREKDIDRFFGDKGAMKAIKEAKEEGIARYTGVTSHGPFAPKLMLECIDRY-DFDSV
A0A151BQE0  TTKRTKDEAAMELRRSMERLGVDQVDLYQFHGLDDPKDLEVAFGPGGALEAVLEARDEGLIRYIGITSHR---PPTILEALRRF-DLDTV
A0A1F5DHV4  TTQRTREGAATELHSSLDRLGVRSLDLYQMHSLKTLEDVDQALGPGGAIEAFREARETGLVKHLGITGHQD--MRANLEALKLF-DFDTV
A0A1F8NH80  TMERDRKGAWDELQRSLKRMRVSAFDLYQAHAVREMGELDQILAPGGALEAFEEARERGLARHIGITGHGYQAPAVHAAAIERF-EFETV
A0A1M3K092  TMERSQQGSWAEMQQSRKRLNIDNFDLYQIHAITTFEELDEATKPDGVLATLDKMKKEGLTDYVGITGHGMQSPQIFLEALNRY-DFDSV
A0A1Q9P4Y9  TRSRQYEGAWKDLETSLNMLKVDTIDLWQMHALTNPQGWEKAMGSEGTLKAFLEARESGHVRFLGVTGHGSKVPKVHLQSLERF-DFDSV
A0A1V5RY86  TGERTYQKAKEEIQRSLERLQTDHVDLIQLHYLVDPQEWEVALGPDGALAAAVEARAQGLARFIGVTGHDLAVPKMHMRSLERY-DFDSV
A0A2E3KCK0  TRARDYQSAKDSIQQSLDRLRVNTIDLLQLHALIHPDEWDLAMSDDGALRALIEAREAGQIRFIGVTGHGWNVAAMHRRSVAHF-DFDSV
A0A351G6T0  TGQRTAQAAKDELQRSMERMGVEQVDLWQMHALVNPQEWQAAMGPGGALEALIEAKQQGLARFIGVTGHGLAAPRMHLRSLEVY-DFDSV
A0A363SWS1  TRKRTHHGALADLNNSLQTLGVDSIDLLQLHGLTNMKGWEDTMGTEGALEVLISAREQGKVRHLGVTGHSNQTPAMHKRSLERY-DFDSV
A0A540VJS1  TGERTYQKAKEEFHRSLERLQVDSVDLIQLHNLVHPDDWDVAMGPGGALEALVEAREQGLVRFIGVTGHGLHAPAMHRRSLEHF-DFDSV
A0A6B0YYY2  TAVRDRDGAWRDVESIMQRMNIEDFDLFQLHSVGNMEQLELATAPGRSLETLVEMREQGLTKWLGITGHGPEVPATILEALKRF-DFDTV
A0A6J4PGY2  TGDRDGDAARRSIERSLDRLQVDFVDLIQLHAIGDLEDLDRATGPGGALEAAIQAREEGLVGAIGITGHGHEAPATHLEALSRY-PFDTV
A0A6J4Y308  TDKLTYPEAREQFHQSLERLQVDRVDLLQMHNLTDVVRREFATGPDGALEFMVEARDKGLVRFLGITGHGILAPKMHLQSLTRF-NFDTV
A0A7C7U6Y6  TGERGFAGARESLHRSLERLRVDSVDLIQLHNLVDENEWERALGEGGALEALVAARDEGLVRFIGVTGHGTRVAGMHRRSLERF-DFDSV
A0A7J3I994  TLERTYEGAWRELRTTLSRFGIESIDVYQFHAVSSLDELDKIFSEKGAAKAFIEARDQGLIKFIGITAHSD--MRIVLKALEIF-DFDTV
A0A7V1H5U5  TLMRKKDDVRKDLYGSLERLNTDHFDMYSLHSVDTDDELETTLGPGGGMQIILDARSQGLLKYVGITSHR---LPLLIKALRMF-DFDAI
A0A7W0SB34  TEQRARAQADMELRRTLERLHRDRFDLYQLHAVCTMDDLDDCFAAGGSMEAILAARDEGLVGPIGITGHAMQAPAVHAAALRRF-DFATV
A0A842U755  TLKRKKRGAWRELNKSLERLGTDHFDLYQFHAVASLDELETILGKGGAMEAFKEAKEAGLIKHIGITAHDD--VRVLQKAIEFSDEIDTI
A0A846PG70  TRRRSRKEALDNLKRSLERLRVDCIDLWQMHGLTNPAGWEKAMGPDGALEALVEAREKGLVRFLGVTGHGSKVAAMHRRSLERF-DFDTV
A0A8J3QVV5  TLQRDAAGARAELERSLGKLRTDRFDLYQMHAVNSDEELAAVLAPGGAAEALVRARDEGLVGAIGITGHFQQVPRLFRTALERV-DLDTV
A0A8T4Z565  TLKRTKEEAWKELRSSMERLGVDNLDLHQFHALDKLEELDKVLGLGGAMEAFLEAREKGLIRFIGIMGHK---PPILLEALRCF-DFDTV
A0A8T5TE33  TMKRTKKGAWRQLNRSLERLDTTYFDLYQFHAVSSMEELDQILGKDGAMEAFKEAKETGIIKHIGITGHDD--VRILQKAITLSDDFDTV
A0A916CPH4  TLERTREGALAELRRSLTRLRVRAFDLYQIHAVTSLEELDKVTAPGGALEAMVEARKQGLTRFIGITGHGINAPLVFLEALKRF-DFDSV
A0A920D8J6  ALERKRDDAKREIERSLTRLQVDRVDLLQLHAVKTFEELDEVTAPGGALEALIEARDQGLTKHIGITGHGHLAPSVHLEALRRF-PFDTV
A0A934XWN3  ------------MRRSLQRLRLDHVDLYQIHAITSMVELDAATRTGGALDAIRAAQQAGLTKHIGITGHGADSPAIFLEALRRF-DFDSV
A0A9E4BYZ3  TTRRTRDEAWRDVEQMMKRLNVENFDLFQLHSVGNISELDKATASGGALETLIEMREQGLTQWLGITGHGPQVPITILESLRRF-DFDTV
A0AA35T6J4  TRERTRELAWANIESCMSRLGVGSFDLFQLHSVGTMEDLDKVTGSGGALEALVELRERGLTRWVGITGHGPDAPRVHLEALRRF-DFDTV
                        .      :    .*: . * :                      :  .     *: .*           :       ::

                  170       180       190       200       210             220       230       240
NphT        LTPYNFILYNNPEYRQAFDALVEEVKRQDVGLMTIKAIARGPWPSED------AKKYATWYEPFDDQEHIDRCVSFVLSRPEITGLASAG
A0A0D8HLA9  LFPYNYMMLTNPQYKSDVEELIEIATNKNVAIQTIKSIARRRWSDDNT-----TDRRFSWYEPIKDDGAIKRAVAFVLS-RPNLFLNTSS
A0A0M0BQ91  LLPVNYVLHAHPQPDNDYEPILSLAQQRNLGIIAMKTAAKGPWPTDAR-------PYLTWYQPFTTQRDVDNALRFTLSQY-VTTAASSS
A0A0M2PNZ0  LLPLNYYMYSLPEYRESFDALMEEANKQNVAVRVIKAIAKAPWKEGS------ERPYATWYEPFDQQEVIDACVHFVLSFPGLAGFASAG
A0A0S8CFH0  LFPINRVHAANANDYTDFSFLLELAKEKDVGTIGIKAIAKRPWAAIMH-------KYATWYEPFAEQTEIDKSLWYALSQG-IATAALPA
A0A133UF86  LLPLNYCMMQIPDYAENFKVLIKTCRNRNIAVQTTKSIARRSAGEE--------NPRNTWYEPLEKQENIDRSIHWNLSNFEDVFLCTAG
A0A151BQE0  LFPLNFVLRRHRCPENDYEPVLKIARERDLGTIVMKAFAKGPWPAEER-------RYRTWYEPFDTQREIDLCLWFALRHN-VTTVTTPS
A0A1F5DHV4  LLPVSLCSAVAWHPANDFRPLLRYALDHDLGVTAIKAACKGRWKGER--------RYGTWYEPSDNQHDITLGVRYTLSQDGVTTYSLPC
A0A1F8NH80  MTPINFVQWADPAFRAAAERLLAVSAEREIGVLVIKALTRGPWDDRPH-------RYATWYEPFDEETAIERCVRFALTRH-VTGFATPG
A0A1M3K092  LFPLNFALYARPNYLEASEKLLQICQERNVGTMIIKSIAKGPWKDQPQ-------THITWYQPFSEQELIQQCVNFALSQP-VTGICTVG
A0A1Q9P4Y9  LLPYNYPDMQNPKYEKNFKDLVKLCSERNVALQTIKSVAGRPWEGQPR------NYNTYFYEPLVTQEAIDKAVHWAYA-LENSFLISAG
A0A1V5RY86  LLPYNFPLMQNPEYAASFEALAALCKIRNVPIQTIKGVTRGPWGDKA-------RVRATWYEPLEKQEDLDKAVWWVLG-RSEMFLNTVG
A0A2E3KCK0  LMPYNWVTSHHVTYGKDFEETLKLCNERKIAVQTIKSLARGPWAAGIS------KRRATWYEPLEADEDIKTAVNFVLA-RPGIFLNSVG
A0A351G6T0  LLPYNYVLMQNPX-------------------------------------------AVWYDPLDTDEGIRHAVHWVLG-NPQVFLNTAG
A0A363SWS1  MLPYSYRQMKIPAYAADFADLITTCRKRKVAVQTIKSIARGPWNDQLK------TFNTYFYEPLVTEDAIQKSVHWVLG-LTDCFLITAG
A0A540VJS1  LLPYNYPMMQNTRYAQDFERLFALCQERNVAVQTIKSITRGPWATHE-------RTRSTWYQPLEDQADIDVAVHWVIG-RPGLFLNTVG
A0A6B0YYY2  MFPLNPAAARDPEYRRVSEALLSECEERDIGVQTIKMIARGGWGDGEK-------QTNTWYDPHREQSDIDASLWWVLSHG-VHTAPSCG
A0A6J4PGY2  LTPWNFILSTDEDYRRDYEALVREIKRQDTGLMTIKTISRRNWPEGDP---TGDQRYTTWYEPFDQQEYVDAAVSFVLSYEEITGIAMVG
A0A6J4Y308  LLPCNYLCLQNPNYAADFNQLVAYCREHDIAVQTIKSAARGLYGDKL-------RSHNTWYEPLSNEDAISKSVQWVLG-KPGLFLITTG
A0A7C7U6Y6  LLPYNFAMLAQPAYAEDFNALMKTCRERNVAVQTIKAVARRRWREEDN------SPRYSWYEPLREPDAIGRAVRFVMS-EENLFLNSSS
A0A7J3I994  LIPVYAAAMTIPRPENDFRPVLKAALDRDIGVIAIKSVAKRRYVGER--------RYTTWYEPFDGQDDIDMAVWYTLSQEPVATYSMAG
A0A7V1H5U5  MFPLNFICYADAGYRADYNELMELARSKDVGVMVIKAIAKRNWGEQYHNTPTWDMPYTTWYEPFSTPAEIKRSLDFVLAQN-ISTAVSAS
A0A7W0SB34  MTAVNPYLYNDAAFRRDWDALTALCRAQDAGVHVLKATAKAPWGDRQP-------THNTWYEPLTGQEAIDQAVAWCLSQP-VTTLCASG
A0A842U755  LMPVYAGALVSPHPANDFRQVLQLAQERNIGITAIKSIAKGRWNGEK--------TFNTWYKPLEDQNLIDYAVWFTLSQEGVTTYSLPC
A0A846PG70  MLPYNYMLMQNPSYAADFNELVGLCRKRNVAVQTIKSIARRPRGSRSK------TFNTYFYEPLKTQDAVDKSVHWSLG-FVDSFLITAG
A0A8J3QVV5  MLPVNAPMLALPDYRAHFAALLDVAAQRNLGVMAIKAVARGPWRTEDH-------HATTWYEPHRDPAAIREAVRFTLSQP-VTGFCMPG
A0A8T4Z565  LFPLNFVQKAHRVYEND-------------------------------------------------------------------------
A0A8T5TE33  LLPVYVASLVNPNPVNDFKSILQITREKNIGVTAIKAISKNRWKGDS--------TYNTWYEPLDKQEFVNQAVWFTLSQDGVTTYSLPC
A0A916CPH4  LFPLNFVQYANPVYRQNAHALIRECRARDVGTMVIKSICRRPWGERNR-------TFATWYEPFADPVHIQQAVNFALSQD-VTGLCTAG
A0A920D8J6  LTPLNFILYSNADYRLAFQELETEVRHQDAGLMVIKAVARGPWKSEE------DRRYATWYEPFDEPEYVDRAVSFVLGNRTLTGLASAG
A0A934XWN3  LFPLNFVQVADPRYRENTDELLRQCQARDVGVMIIKAITKGPWGGRPQ-------THTTWYEPFDRMEDIQRAVNFALSFP-VTGLCTAG
A0A9E4BYZ3  MFPLNPASMRDANYRRDAEALIAEANARDIGRSRAAAGAKIQKTTAPG-------TTR---------TENKK----PLTVP-CGGYCPNR
A0AA35T6J4  MFPLNATLYRNDEYREHAEELLSVANQRDVGVHTIKMLARGGWGDRER-------EHTTWYDPHREQSEIDRALWWLLSQP-MHTAPSVG
            :
```

```
              250        260            270          280
NphT          DVRLLPKIVEAYKRYRKL----SGEEQEALMAT--AGSYASPFGPF--------------------------------------------
A0A0D8HLA9    DYSLLPKVIEAANHLPKD----LDLVAIEADAR--SLEMAPLFDGSTLELI---------------------------------------
A0A0M0BQ91    DVRIATMMLDAAERYTPM----DATEQEALLTS--AAAYTPLFPRATP------------------------------------------
A0A0M2PNZ0    DIHLFPKIVDAVERYGSM----NDEEAARILKS--ISGYTTIFGAPTSIA----------------------------------------
A0A0S8CFH0    DLRLWPMVIDAAERASAL----DLKTQEQFVAE--VKDYKPIFPNE--------------------------------------------
A0A133UF86    DVDLLPKFLDAIEKYEGKG---PSNEEMEEMVE--RLGMEVLSGWPRLRD----------------------------------------
A0A151BQE0    DTRLIPLVIDAAERYREL----SDEEVESLLES--AAELKPLFPRT--------------------------------------------
A0A1F5DHV4    ETRLWGMVLKAGEEYTPM----TPEEQEEAITYARGQGFTPLFPE---------------------------------------------
A0A1F8NH80    DLRLLPAALKAAERFRPL----SEAESEALLES--ASAYRPLFARA--------------------------------------------
A0A1M3K092    DITVLPMVLNACQNFTPL----SAEEQNELIRQ--GEKMEMFF-----------------------------------------------
A0A1Q9P4Y9    DMKILPKVLEAANRFKKR----PTDTEMNKFIE--KYGVTSIFSY---------------------------------------------
A0A1V5RY86    DIHVLPRVLDAAARFEHR----PSNQEMQALVA--QAEMAPLFV----------------------------------------------
A0A2E3KCK0    DVGLLPSLLRAASERIEL----PDPNVMEAHSQ--RTGLSSIFGI---------------------------------------------
A0A351G6T0    DIRLLEKVLAAADQFETR----PTDQVMQADLQ--QYGITSLFTGDEL------------------------------------------
A0A363SWS1    DIQLLPKILRAASIFEQR----PSDAEMESLVA--EYDIQPIFKG---------------------------------------------
A0A540VJS1    DIHLLPKVLDAASRFERR----PSDEEIAAVVN--RQHMTPLFA----------------------------------------------
A0A6B0YYY2    EISLLPKVLDAAERFKPL----SEDQMRAVEES--QRPPLPLPDLAIPAAA---------------------------------------
A0A6J4PGY2    DVTLVPMMLEAEGR--RM----PREEAEKVLAA--APNHSSPFISVPF------------------------------------------
A0A6J4Y308    DMQVLPTILAAAADFQTS----PPDEDMELMVA--EQGMQPLFDQ---------------------------------------------
A0A7C7U6Y6    DATLLPTILEAAQKTGKAGQITQRDAELRADAE--RYGVEALFVPGGPEGI---------------------------------------
A0A7J3I994    DLRLWPMILNAGKRFRKL----SIEEQNRVVEVFREKGARPLFPENV-------------------------------------------
A0A7V1H5U5    DATLLTLMLDAAEKVSPV----DKIDQNSLLDE--AERYKPLVFTFSP------------------------------------------
A0A7W0SB34    DLAIFSKTVEAAKRYRSL----GPITAPEVLAT--VPDYGNIFAGAEA------------------------------------------
A0A842U755    DVRLWSKVLNAVTRYRKL----SFEEQQNFVDQAKKSDFHPLYPE---------------------------------------------
A0A846PG70    DTQLLPKMLDAANRFEKR----PSDIEMSALVD--TFGIRPIFSRSAHRKG-KGSQTWGYQMYRALEGL-----IREGFFK--HPNKRTL
A0A8J3QVV5    DVSIHPDALAAAEQFTPL----PQDEIEARIAA--ADRADALVS----------------------------------------------
A0A8T4Z565    ------------------------------------------------------------------------------------------
A0A8T5TE33    DVKLWPLVLNAVKLYKKL----DAKDLEKIVNIARENELQPLFPE---------------------------------------------
A0A916CPH4    DVSILPLMLNACAHYMPM----SVEDQETLMAS--AAQFEPLFT----------------------------------------------
A0A920D8J6    DIHLLPRIVEAVRRRREL----SEEEVRELLRS--APRYGSPFGDLATGQL--GGIPFR-------------------------------
A0A934XWN3    DTTVLPLMLKACQNFTPQ----SAAEREALIHM--GRPVRAVVCRGACQPVIDDCHSASKRSRQRVKRVRLELSMIQGNFICLRPVKQAD
A0A9E4BYZ3    YTPPPPAAKSACCPKSST----P------------PSDSPPSTPTNRPPPSLHSDHPSRFPHWRFL---KCE-DTSVGATPCTI------
A0AA35T6J4    DVELLPRALDAAQRFSPL----SNDDQEAVVVG--QCPPLPEPGLGILAAD---------------------------------------

NphT          ------------------------------------------------------------------------------------------
A0A0D8HLA9    ------------------------------------------------------------------------------------------
A0A0M0BQ91    ------------------------------------------------------------------------------------------
A0A0M2PNZ0    ------------------------------------------------------------------------------------------
A0A0S8CFH0    ------------------------------------------------------------------------------------------
A0A133UF86    ------------------------------------------------------------------------------------------
A0A151BQE0    ------------------------------------------------------------------------------------------
A0A1F5DHV4    ------------------------------------------------------------------------------------------
A0A1F8NH80    ------------------------------------------------------------------------------------------
A0A1M3K092    ------------------------------------------------------------------------------------------
A0A1Q9P4Y9    ------------------------------------------------------------------------------------------
A0A1V5RY86    ------------------------------------------------------------------------------------------
A0A2E3KCK0    ------------------------------------------------------------------------------------------
A0A351G6T0    ------------------------------------------------------------------------------------------
A0A363SWS1    ------------------------------------------------------------------------------------------
A0A540VJS1    ------------------------------------------------------------------------------------------
A0A6B0YYY2    ------------------------------------------------------------------------------------------
A0A6J4PGY2    ------------------------------------------------------------------------------------------
A0A6J4Y308    ------------------------------------------------------------------------------------------
A0A7C7U6Y6    ------------------------------------------------------------------------------------------
A0A7J3I994    ------------------------------------------------------------------------------------------
A0A7V1H5U5    ------------------------------------------------------------------------------------------
A0A7W0SB34    ------------------------------------------------------------------------------------------
A0A842U755    ------------------------------------------------------------------------------------------
A0A846PG70    EHVVK-------------------------ALESKGLPTESKRANITNALAGRVRKGVLKKSEV--------------------ANGWVY
A0A8J3QVV5    ------------------------------------------------------------------------------------------
A0A8T4Z565    ------------------------------------------------------------------------------------------
A0A8T5TE33    ------------------------------------------------------------------------------------------
A0A916CPH4    ------------------------------------------------------------------------------------------
A0A920D8J6    ------------------------------------------------------------------------------------------
A0A934XWN3    LSLLEAWQNDVEIQSEYNAFGLKRFDNQERAFAEHGFLTDQLSTLMIVTPAGE-PVGTMSFHQVHYGPNGGSLAYNIGLNIVPTHRGKGY
A0A9E4BYZ3    ------------------------------------------------------------------------------------------
A0AA35T6J4    ------------------------------------------------------------------------------------------
```

```
       NphT   ---------------------------------------------------------------------
A0A0D8HLA9   ---------------------------------------------------------------------
A0A0M0BQ91   ---------------------------------------------------------------------
A0A0M2PNZ0   ---------------------------------------------------------------------
A0A0S8CFH0   ---------------------------------------------------------------------
A0A133UF86   ---------------------------------------------------------------------
A0A151BQE0   ---------------------------------------------------------------------
A0A1F5DHV4   ---------------------------------------------------------------------
A0A1F8NH80   ---------------------------------------------------------------------
A0A1M3K092   ---------------------------------------------------------------------
A0A1Q9P4Y9   ---------------------------------------------------------------------
A0A1V5RY86   ---------------------------------------------------------------------
A0A2E3KCK0   ---------------------------------------------------------------------
A0A351G6T0   ---------------------------------------------------------------------
A0A363SWS1   ---------------------------------------------------------------------
A0A540VJS1   ---------------------------------------------------------------------
A0A6B0YYY2   ---------------------------------------------------------------------
A0A6J4PGY2   ---------------------------------------------------------------------
A0A6J4Y308   ---------------------------------------------------------------------
A0A7C7U6Y6   ---------------------------------------------------------------------
A0A7J3I994   ---------------------------------------------------------------------
A0A7V1H5U5   ---------------------------------------------------------------------
A0A7W0SB34   ---------------------------------------------------------------------
A0A842U755   ---------------------------------------------------------------------
A0A846PG70   WTE------------------------------------------------------------------
A0A8J3QVV5   ---------------------------------------------------------------------
A0A8T4Z565   ---------------------------------------------------------------------
A0A8T5TE33   ---------------------------------------------------------------------
A0A916CPH4   ---------------------------------------------------------------------
A0A920D8J6   ---------------------------------------------------------------------
A0A934XWN3   GTEAQRLLADYLLRTYPIARVEASTDVTNLPEQRSLDKAGFRREGVLRSAQWRSGAWHDMVLYSKVRGE
A0A9E4BYZ3   ---------------------------------------------------------------------
A0AA35T6J4   ---------------------------------------------------------------------
```

Supplementary Figure 35. Structural comparison of NphT and a canonical AKR. (A) Structural alignment of NphT (PDN 9YEB) and L-glyceraldehyde 3-phosphate reductase (AKR14A1, UniProt Q46851, PDB 4AUB)[21], an NADPH-dependent AKR from *E. coli*. Alignments were performed using the Match Function in Chimera v1.7.1. Parameters: Chain pairing: bb; Alignment algorithm: Needleman-Wunsch; Similarity matrix: BLOSUM-62. RMSD between 125 pruned atom pairs is 1.043 Å. RMSD cross all 262 atom pairs is 5.597 Å. (B) NPMN cofactor binding pocket is capped with several residues that prevent binding of a cofactor containing an extension from the phosphate group. (C) Residues that cap the cofactor binding pocket in NphT would clash with the AMP moiety of the larger, $NADP^+$ cofactor that is found in AKR14A1.

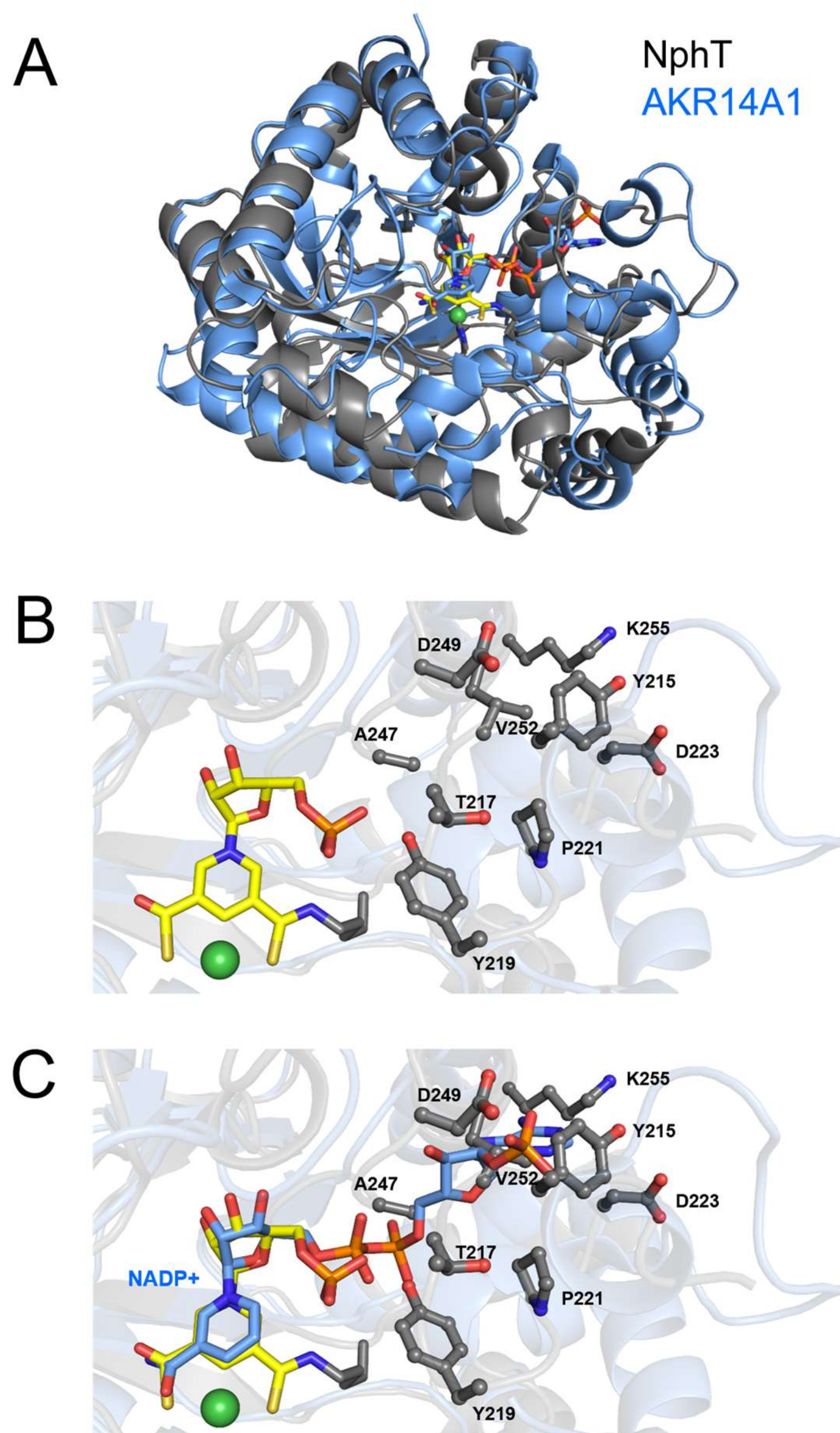

Supplementary Figure 36. Size exclusion chromatography to determine oligomeric state of NphT. 100 µL apo protein sample was injected into a Superdex™ 75 Increase 10/300 GL column (Cytiva) equilibrated with SEC Buffer (20 mM HEPES, 100 mM NaCl) and eluted at a flow rate of 0.4 mL/min. (A) Calibration standards: 1, Bovine serum albumin (66,500 Da); 2, Ovalbumin (44,000 Da); 3, Carbonic anhydrase (29,000 Da); 4, Cytochrome C (12,300 Da). (B) NphT. (C) Calculated NphT M_r = 26,392. Calculated mass of NphT polypeptide = 32,559.7 Da. This experiment was performed once.

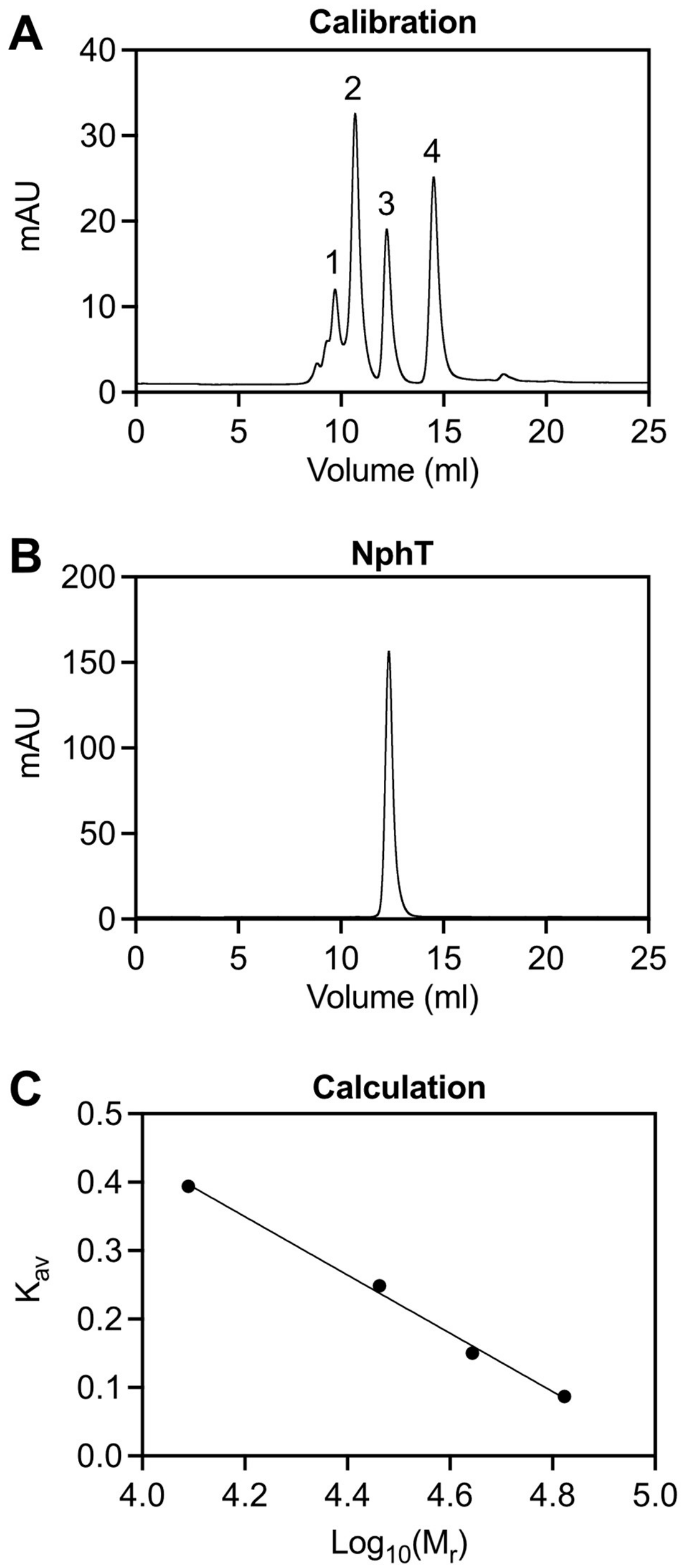

Supplementary Figure 37. Docking of sugar tautomers with NphT. Docking was performed using AutoDock Vina 1.2.7[17,18]. The receptor was obtained as the chain B of the crystal structure of NphT (PDB accession: 9YEB). The water molecules were removed before preparing the receptor using the AutoDock Tools from the AutoDock Suite[19]. The ligands were input as SMILES strings into the Scrubber script (https://github.com/forlilab/molscrub) to generate the 3D conformers of the ligand, which are then parameterized using the Meeko package[20]. The active site of NphT was contained in a cubic grid box of dimensions 15 Å x 15 Å x 15 Å with 0.375 Å spacing, centered around the position -43.928 x 3.180 x 37.358, which corresponds to the Ni center of NPMN. The exhaustiveness parameter was set to 32 and the rest of the docking parameters were set to default. The results and images were analyzed using PyMol. The top pose with a hydride transfer distance <4 Å and proper orientation of the hydroxyl toward Tyr 52 are shown below.

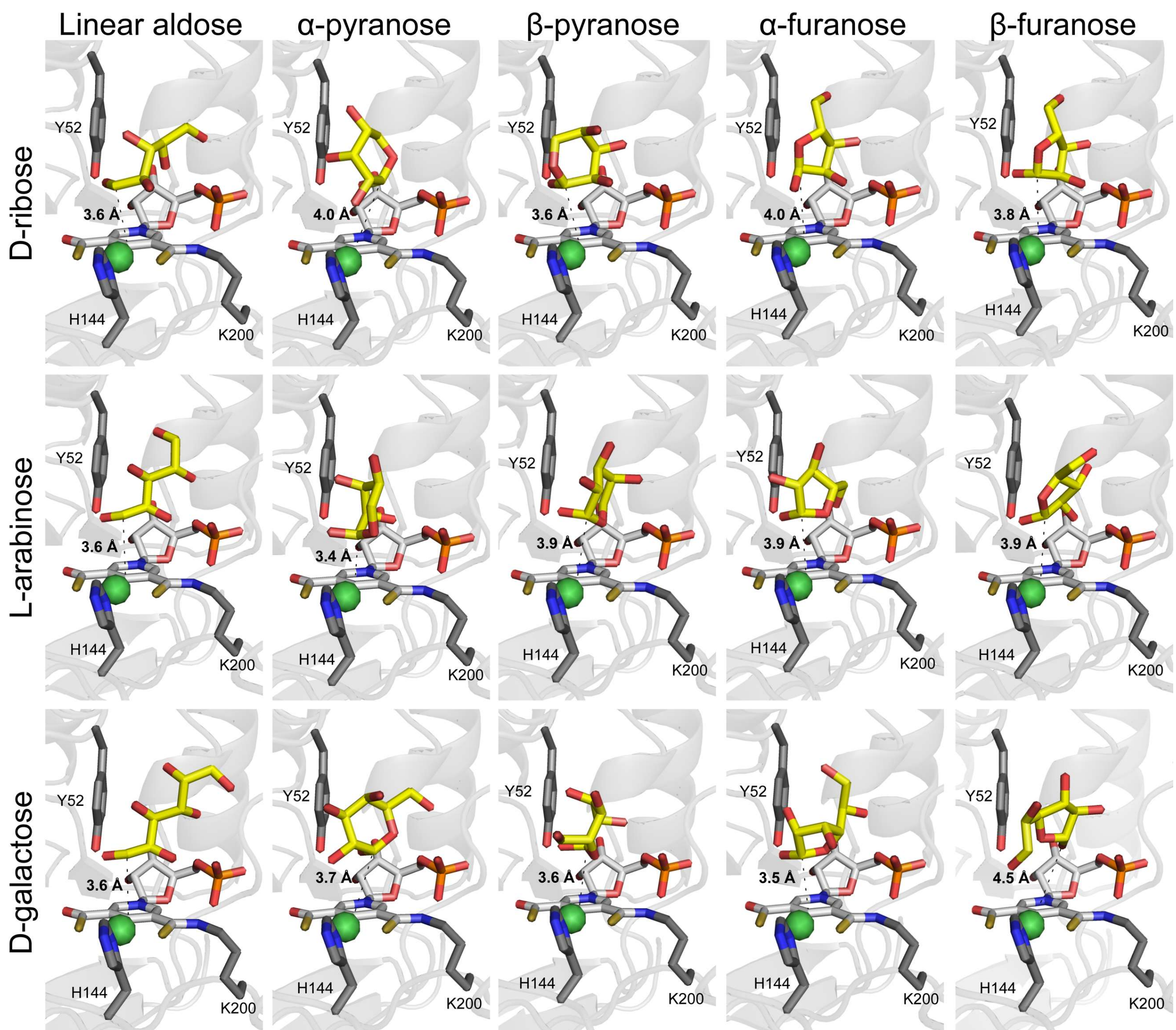

Supplementary Figure 37. Docking of sugar tautomers with NphT (continued).

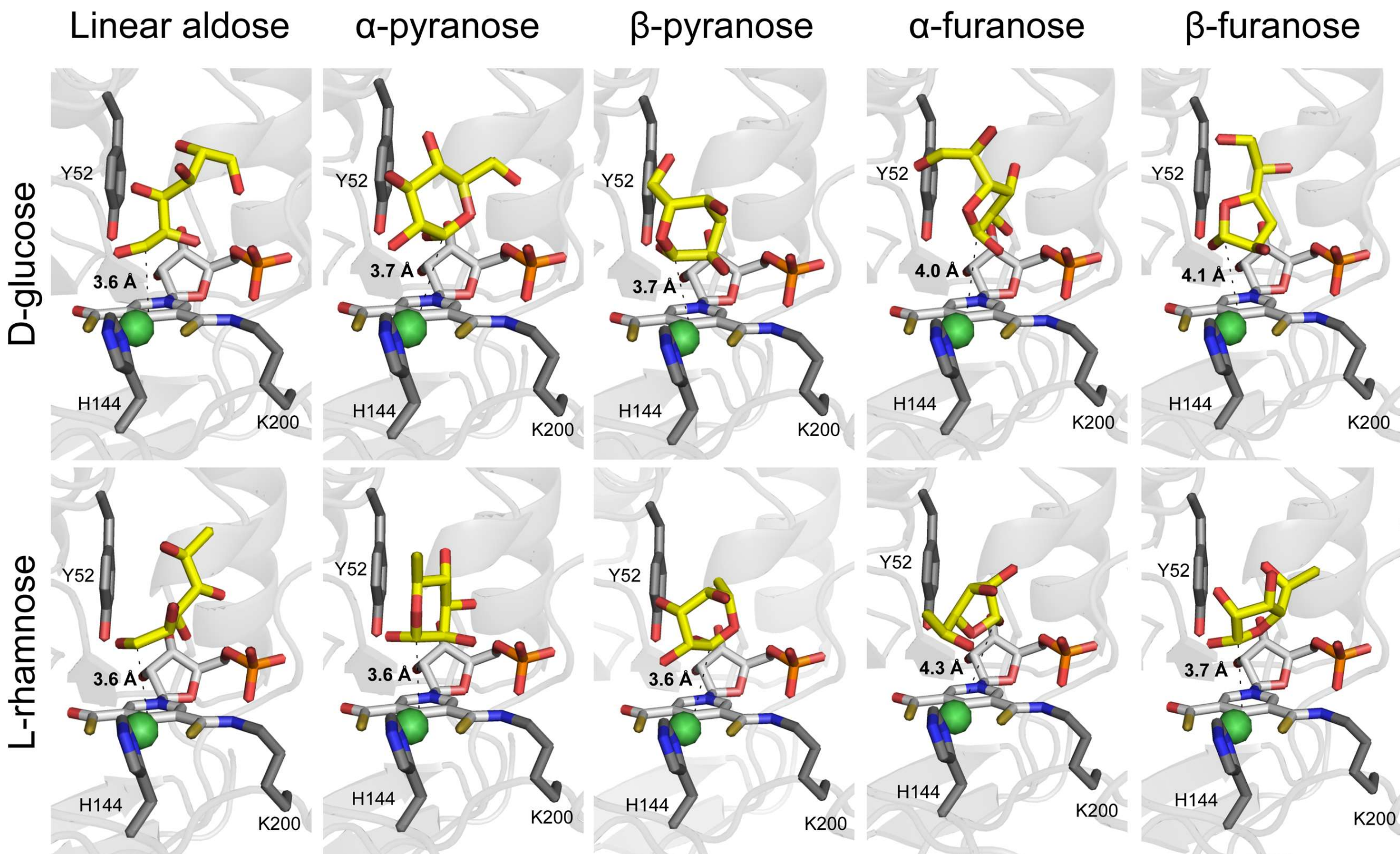

Supplementary Figure 38. Cofactor loading. *In vitro* cofactor synthesis was performed as described in the methods. The NPMN cofactor or control with LarC and E omitted from the reaction was incubated with 20 µM LarA, *E. coli* AKR, or 40 µM NphT or a mutant for 20 hours. Protein samples were prepared and analyzed as described in the methods. LarA -NPMN expected: 47392.4 Da, observed: 47392.3 Da. LarA +NPMN expected: 47842.3 Da, observed: 47842.5. *E. coli* AKR -NPMN expected: 39123.1, observed: 39124.0. *E. coli* AKR +NPMN expected: 39573.0 Da, observed: 39124.0 Da. NphT -NPMN expected: 32559.7 Da, observed: 32560.7 Da. NphT +NPMN expected: 33009.6 Da, observed: 33010.6 Da. K200A -NPMN expected Da: 32502.6, observed: 32503.8 Da. K200A +NPMN expected: 32952.5 Da, observed: 32503.8. H144G -NPMN expected: 32479.6 Da, observed: 32480.7 Da. H144G +NPMN expected: 32929.5 Da, observed: 32930.6 Da. D47A -NPMN expected: 32515.7 Da, observed: 32516.7 Da. D47A +NPMN expected: 32965.6 Da, observed: 32966.6 Da. Data are representative of n=3 experimental replicates.

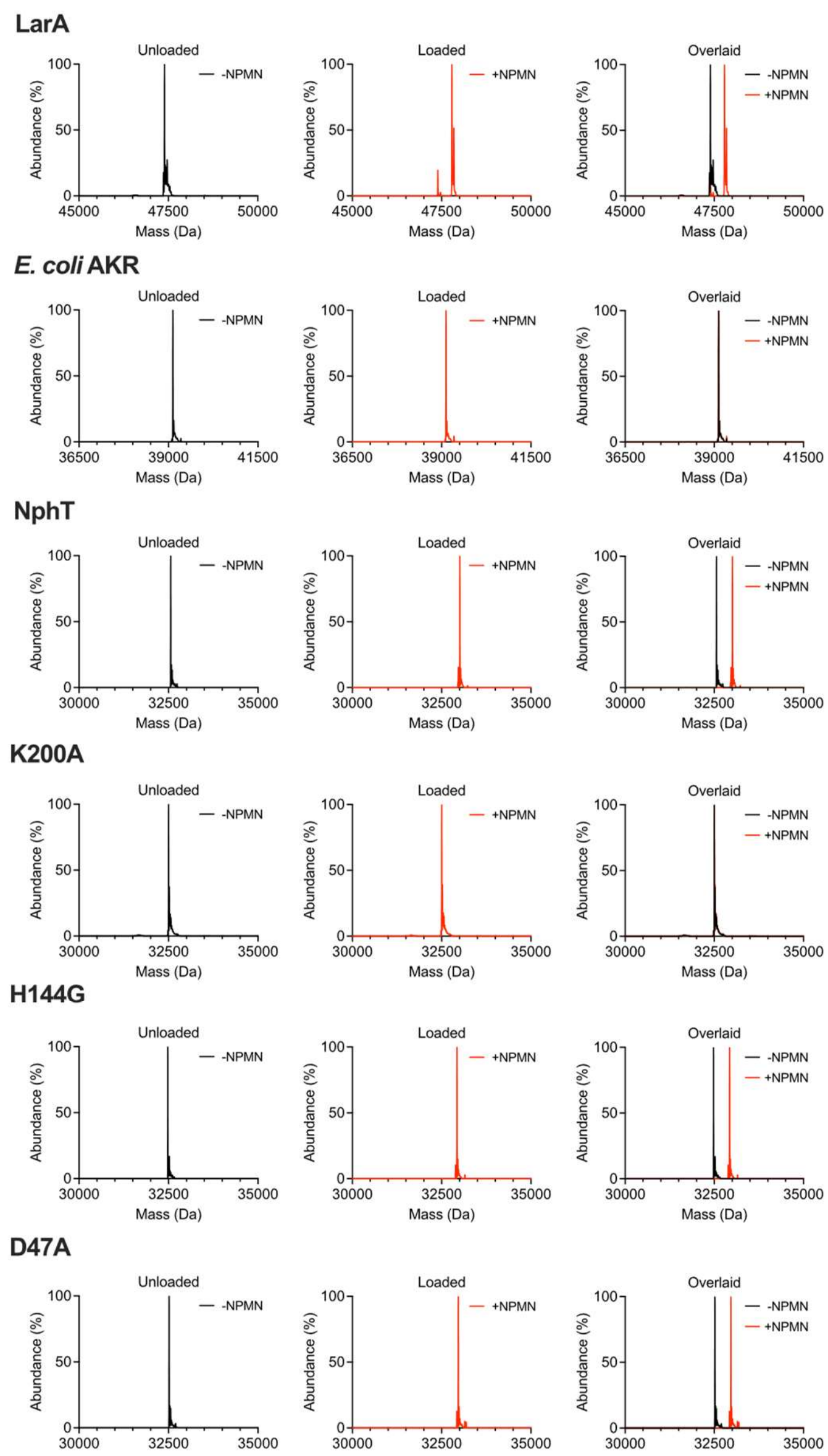

Supplementary Figure 38. Cofactor loading (continued). H106A -NPMN mass (Da) expected: 32493.6, observed: 32494.4. H106A +NPMN mass (Da) expected: 32943.5, observed: 32944.3. K75A -NPMN mass (Da) expected: 32502.6, observed: 32503.6. K75A +NPMN mass (Da) expected: 32952.5, observed 32953.6. W218A -NPMN mass (Da) expected: 32444.6, observed: 32445.6. W218A +NPMN mass (Da) expected: 32894.5, observed: 32895.4. Y52A -NPMN mass (Da) expected: 32467.6, observed: 32468.8. Y52A +NPMN mass (Da) expected: 32917.5, observed: 32918.6. Y52F -NPMN mass (Da) expected: 32543.7, observed: 32544.7. Y52F +NPMN mass (Da) expected: 32993.6, observed: 32994.5. Data are representative of n=3 experimental replicates.

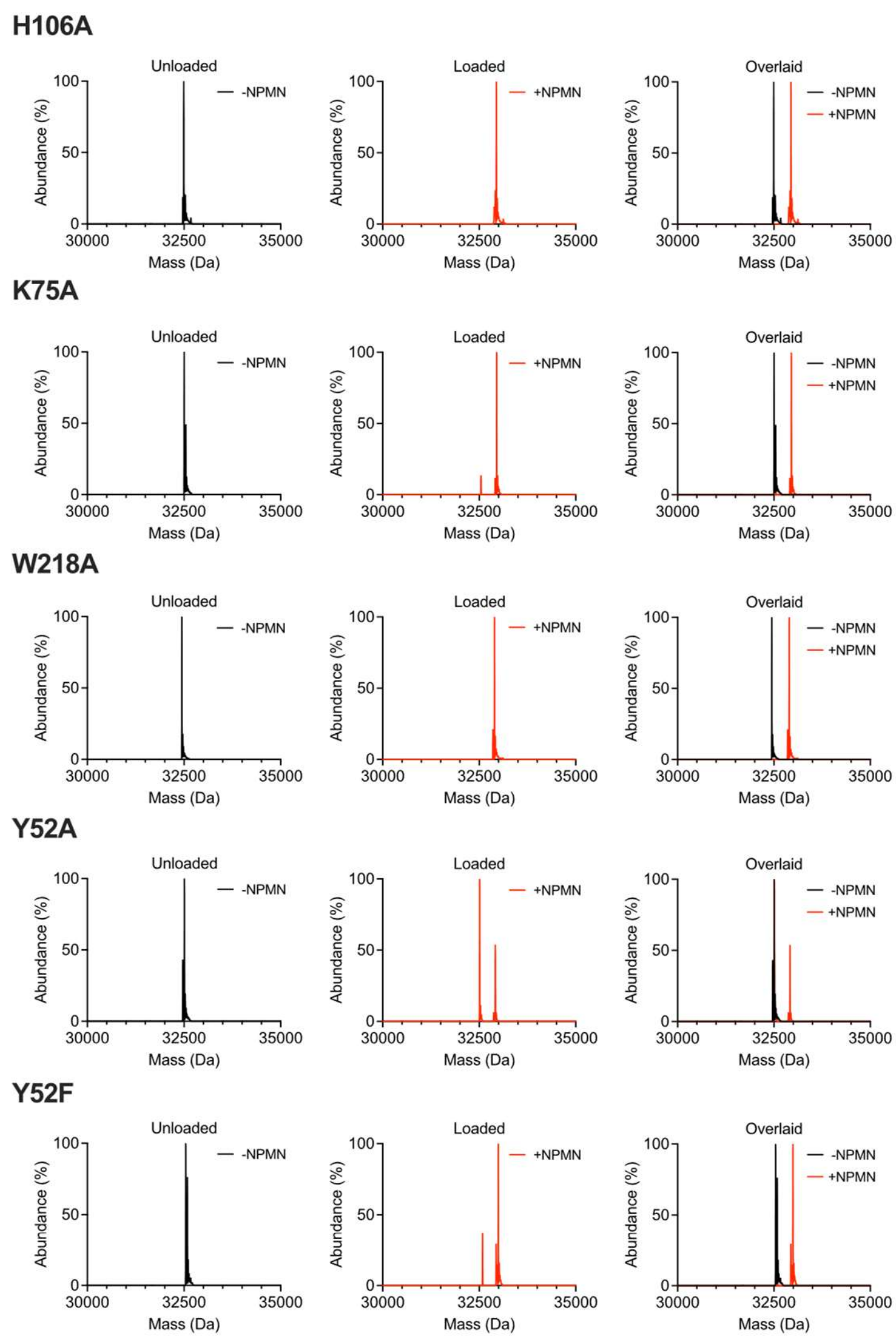

Supplementary Figure 39. Structural comparison of NphT (PDB ID 9YEB) and *Sp* LarA homolog (PDB ID 9Q3K). (A) Active site of NphT. (B) Alternative view of NphT active site with labels for residue distances relative to catalytic tyrosine. (C) Active site of *Sp* LarA. (D) Alternative view of *Sp* LarA active site with labels for residue distances relative to analogous tyrosine residue. *Sp* LarA has several histidine residues positioned near Y307 which could serve an analogous role to H106 found in LarA. Several other histidine and lysine residues could potentially serve a proton shuttling role in *Sp* LarA. The function of *Sp* LarA is not currently known.

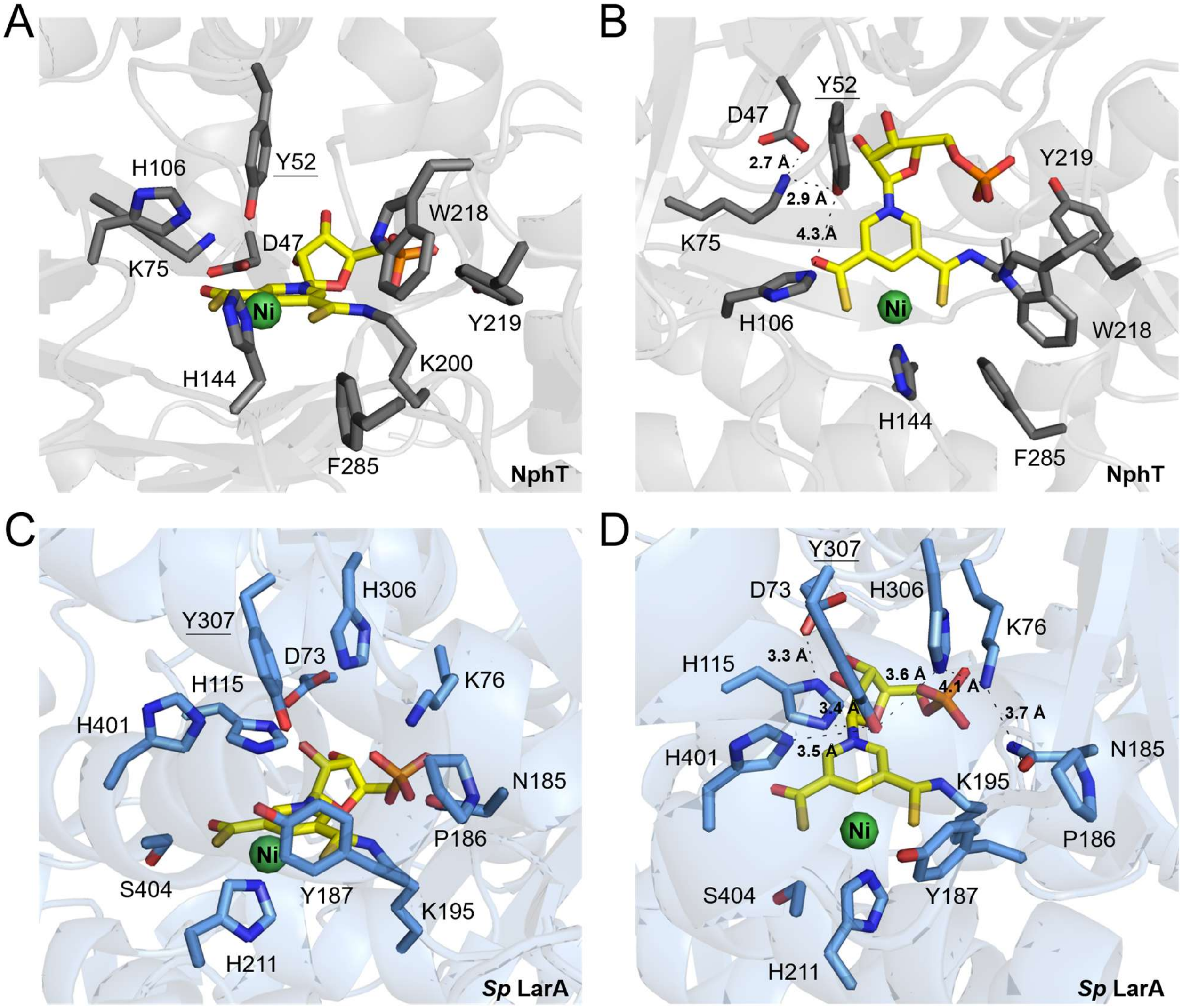

Supplementary Figure 40. Transhydrogenation with L-arabinose and D-[2H]-galactose substrates. Holo NphT was prepared and purified by size exclusion chromatography as described in the methods. Reactions contained NphT (5 μM), 20 mM HEPES pH 7.5, and 10 mM of the following substrates: A) L-[1-H]-arabinose, B) D-[1-H]-galactose, C) D-[1-D]-galactose, D) L-[1-H]-arabinose and D-[1-H]-galactose, E) L-[1-H]-arabinose and D-[1-D]-galactose. Following incubation at 50°C for 5 h, 10 μL of the reaction was quenched in 70 μL of 6:1 acetonitrile:water and freeze-thawed three times to precipitate protein. 10 μL of methanol was added to the sample before centrifugation at 13,000 x *g*. Samples were analyzed by LC-MS in negative ionization mode $[M-H]^-$ using a HILIC column as described in the methods. Data are representative of n=2 experimental replicates.

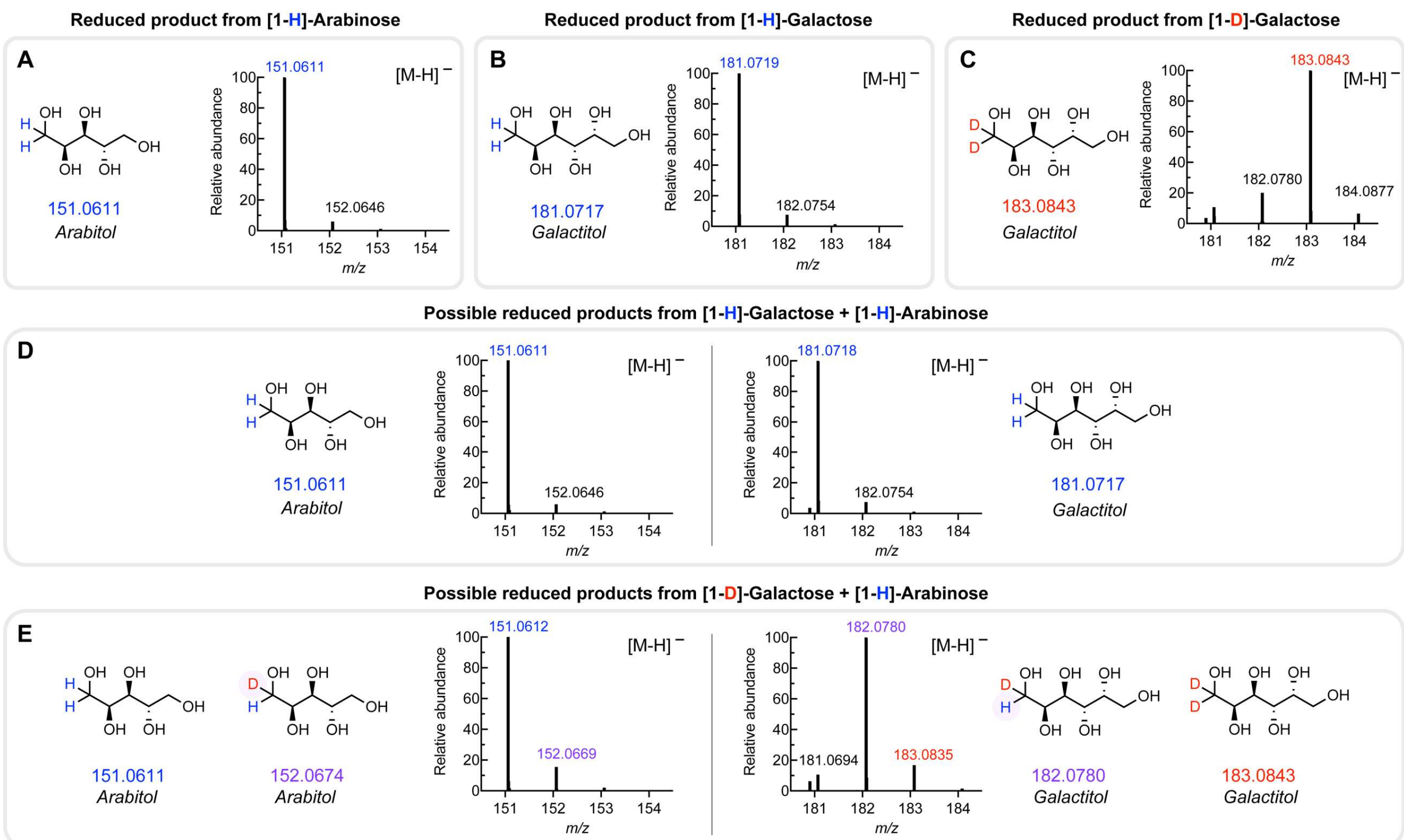

Supplementary Figure 41. Time-dependent substrate competition of NphT with L-arabinose, D-galactose, and D-ribose by quantitative ^{13}C NMR. In a Coy anaerobic chamber, the NPMN cofactor was synthesized *in vitro* as described in the methods and incubated with 40 µM NphT for 20 hours. NphT was exchanged into water using a PD-10 column, and fractions were combined and concentrated to 12.15 mg/mL using a 500 µL Modified PES VWR Centrifugal Filter, 10 kDa MWCO (VWR). A reaction (600 µL) containing holo (+NPMN) NphT (40 µM) was incubated with L-arabinose, D-galactose, and D-ribose (45 mM) in 100 mM HEPES pH 7.5 at 50°C, where 100 µL was quenched by gently bubbling compressed air at 0, 0.5, 2, 5, and 24 hours. After drying down in a speed vacuum concentrator, the samples were resuspended in a final volume of 600 µL in D_2O before being transferred to a tube for NMR analysis. Quantitative ^{13}C NMR spectroscopy was performed with 4096 scans and a d1 of 14 s. This experiment was performed once, and the concentration of each molecule in every reaction was assessed using ^{13}C peak integration in Mnova performed in triplicate. The data are the average of n=3 integration replicates ± s.d. (A) Depletion of substrates L-arabinose, D-galactose, and D-ribose over time. (B) Formation of alcohol products over time. (C) Formation of lactone and acid products over time. (D) Table of the calculated concentrations of each compound over time.

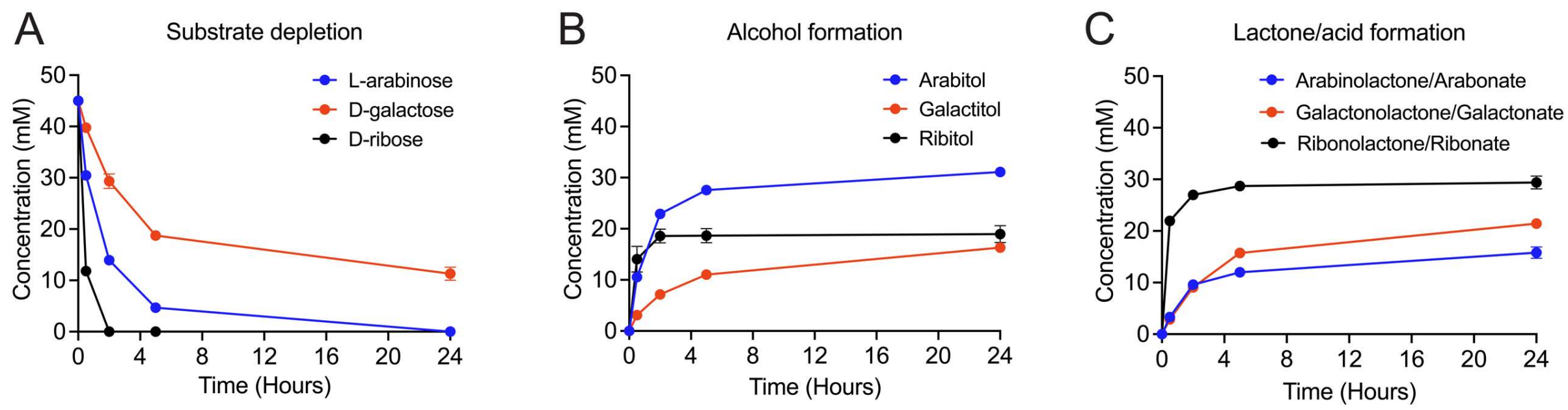

D

Concentration (mM) at given time

Compound	0 hrs	0.5 hrs	2 hrs	5 hrs	24 hrs
L-arabinose	45.00 ± 0.00	30.46 ± 0.27	13.93 ± 0.18	4.69 ± 0.32	0.00 ± 0.00
Arabitol	0.00 + ± 0.00	10.55 ± 0.63	22.92 ± 0.28	27.56 ± 0.64	31.10 ± 0.39
Arabinolactone	0.00 ± 0.00	0.00 ± 0.00	2.02 ± 0.46	2.93 ± 0.08	3.76 ± 0.19
Arabonate	0.00 ± 0.00	3.31 ± 0.01	7.56 ± 0.24	9.08 ± 0.81	12.06 ± 0.91
D-ribose	45.00 ± 0.00	11.80 ± 0.89	0.00 ± 0.00	0.00 ± 0.00	0.00 ± 0.00
Ribitol	0.00 ± 0.00	14.05 ± 2.53	18.57 ± 1.34	18.66 ± 1.38	18.95 ± 1.65
Ribonolactone	0.00 ± 0.00	1.99 ± 0.42	3.07 ± 0.13	4.53 ± 0.14	4.95 ± 0.60
Ribonate	0.00 ± 0.00	19.96 ± 0.31	23.90 ± 0.69	24.17 ± 0.79	24.44 ± 0.67
D-galactose	45.00 ± 0.00	39.76 ± 0.40	29.34 ± 1.40	18.74 ± 0.71	11.30 ± 1.27
Galactitol	0.00 ± 0.00	3.11 ± 0.09	7.14 ± 0.30	11.02 ± 0.40	16.32 ± 0.23
Galactonolactone	0.00 ± 0.00	0.00 ± 0.00	1.37 ± 0.22	4.59 ± 0.18	6.51 ± 0.30
Galactonate	0.00 ± 0.00	2.88 ± 0.30	7.71 ± 0.54	11.15 ± 0.40	14.93 ± 0.18

Supplementary Figure 42. NMR analysis of transhydrogenation between ribose and butanal. In a Coy anaerobic chamber, the NPMN cofactor was synthesized *in vitro* as described in the methods and incubated with 40 µM NphT for 20 hours. NphT was purified using size exclusion chromatography, and fractions were combined and concentrated to 100 µM using a 500 µL Modified PES VWR Centrifugal Filter, 10 kDa MWCO (VWR). (A) Reactions (100 µL) contained varying combinations of holo NphT (10 µM), D-ribose (50 mM), and butanal (30 mM) in 20 mM HEPES pH 7.5 in D_2O. Reactions proceeded at 50°C for 18 hours before removal from the anaerobic chamber. 500 µL D_2O was added to each sample prior to analysis by NMR. (B) Proton NMR of reaction products. (C) Proton NMR zoomed in between 0.7 and 1.8 ppm. (D) Carbon NMR. The peak at 1.2 ppm (^1H) and 24 ppm (^{13}C) seen in the D_2O control corresponds to residual isopropanol. Butanol ^1H NMR (600 MHz, D2O) δ 3.52 (t, J = 6.6 Hz, 2H), 1.43 (p, J = 6.8 Hz, 2H), 1.26 (h, J = 7.4 Hz, 2H), 0.81 (t, J = 7.4 Hz, 3H). ^{13}C NMR (151 MHz, D2O) δ 61.51, 33.40, 18.34, 13.01. Butyrate ^1H NMR (600 MHz, D2O) δ 2.06 (t, J = 7.3 Hz, 2H), 1.46 (h, J = 7.4 Hz, 2H), 0.80 (t, J = 7.4 Hz, 3H). ^{13}C NMR (151 MHz, D2O) δ 184.14, 39.58, 19.33, 13.18. Data shown are representative of n=2 experimental replicates.

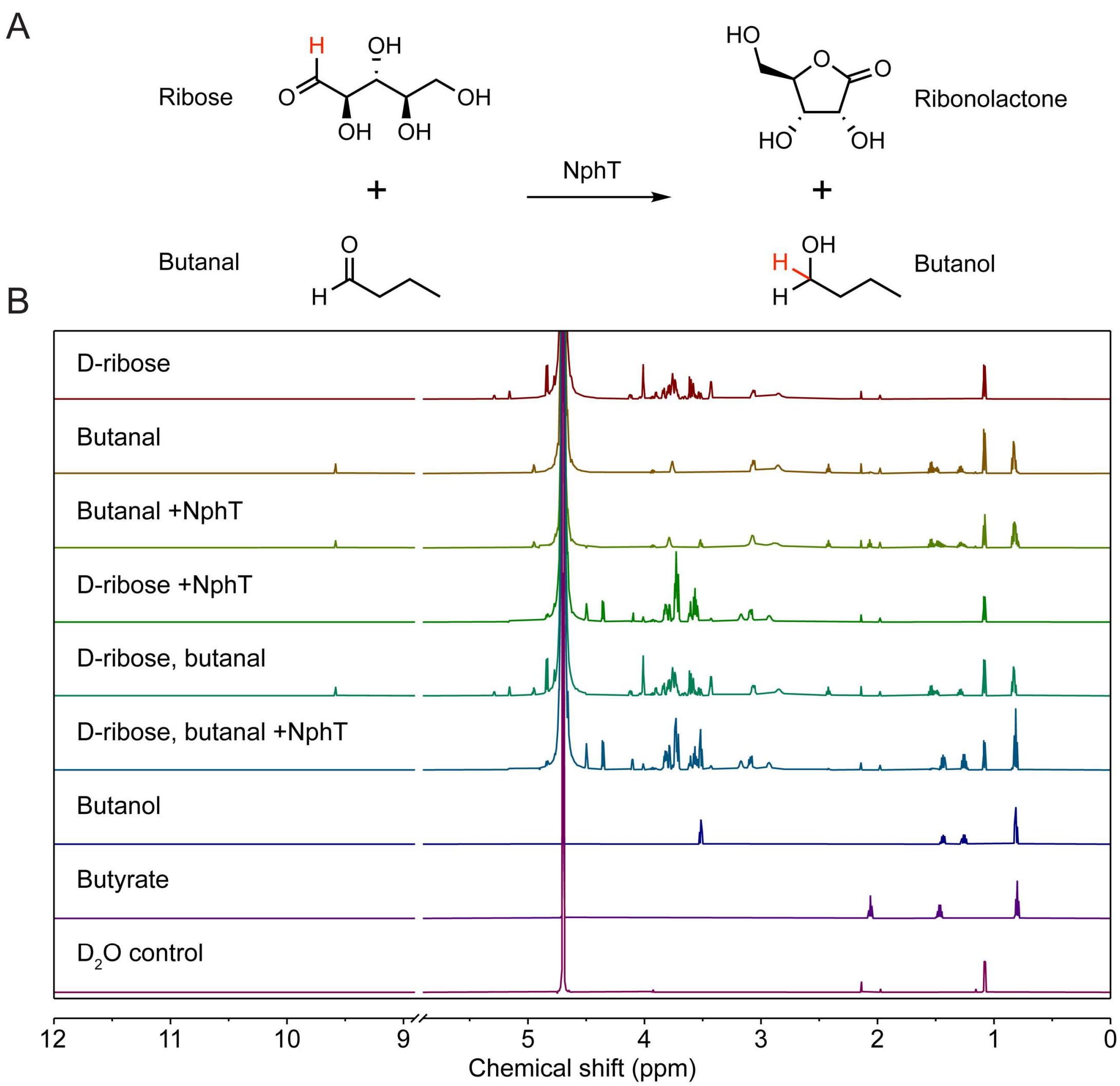

C

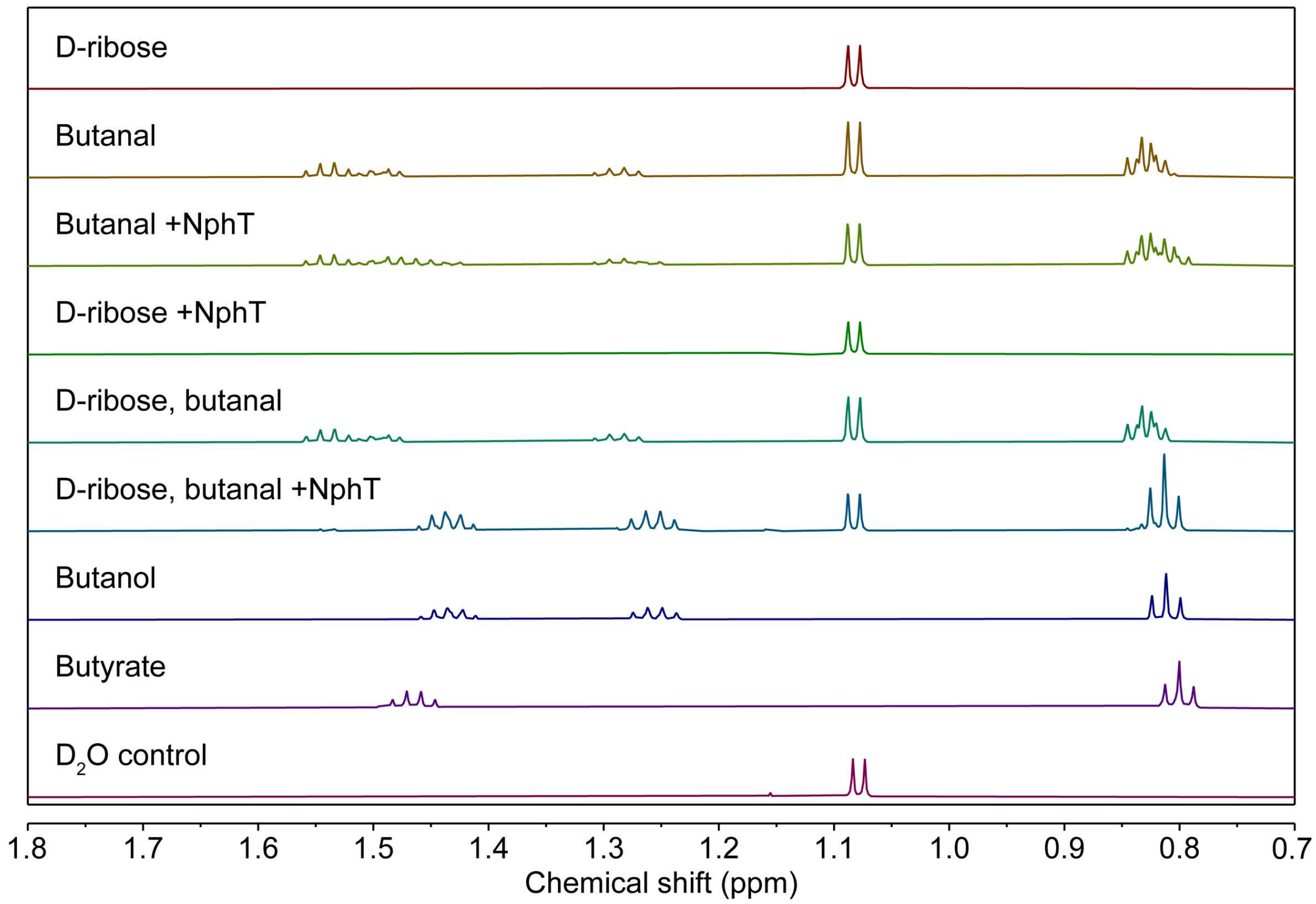
D-ribose
Butanal
Butanal +NphT
D-ribose +NphT
D-ribose, butanal
D-ribose, butanal +NphT
Butanol
Butyrate
D_2O control
1.8
1.7
1.6
1.5
1.4
1.3
1.2
1.1
1.0
0.9
0.8
0.7
Chemical shift (ppm)

D

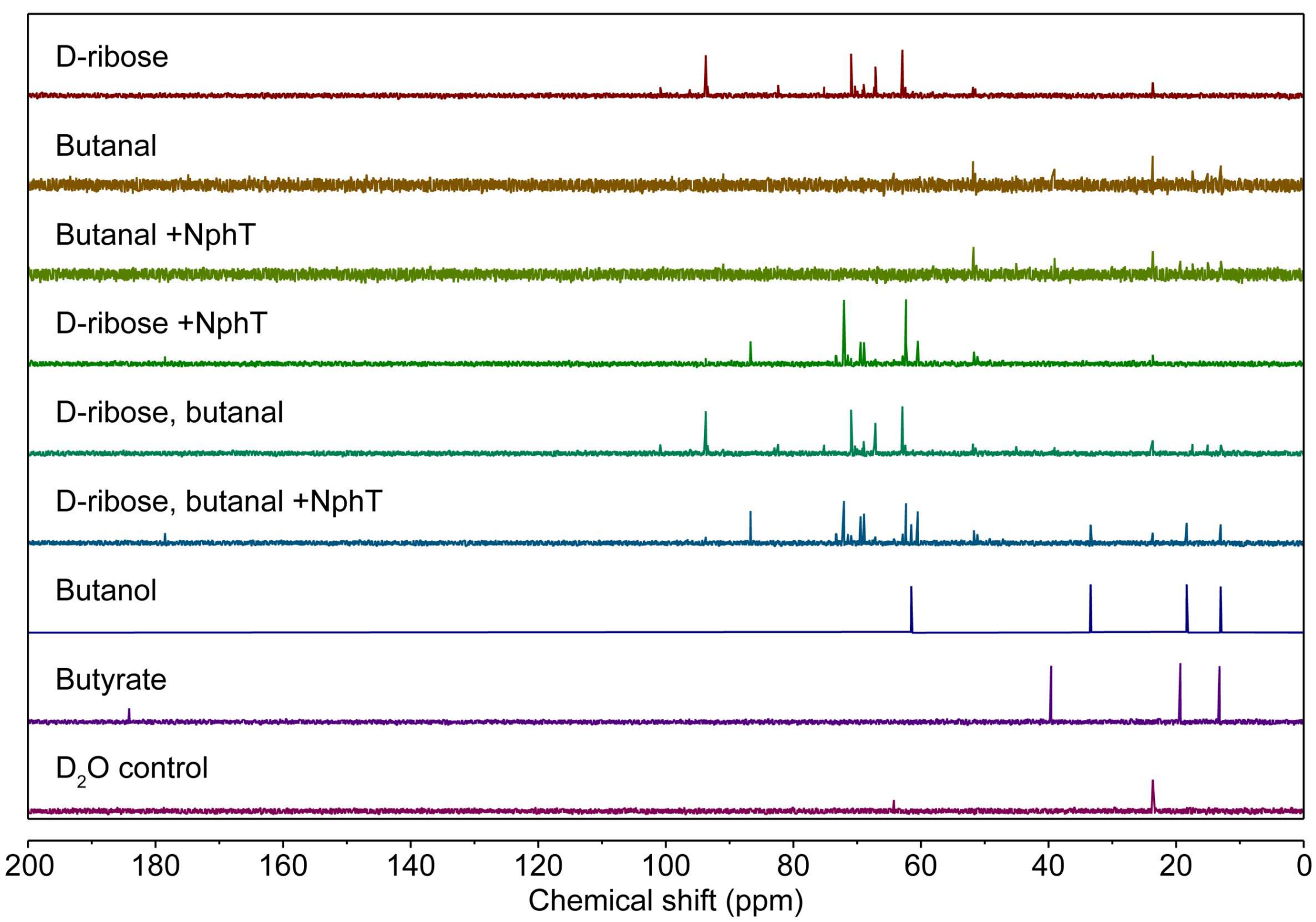
D-ribose
Butanal
Butanal +NphT
D-ribose +NphT
D-ribose, butanal
D-ribose, butanal +NphT
Butanol
Butyrate
D_2O control
200
180
160
140
120
100
80
60
40
20
0
Chemical shift (ppm)

Supplementary Figure 43. NMR analysis of inositol incubation with NphT. In a Coy anaerobic chamber, the NPMN cofactor was synthesized *in vitro* as described in the methods and incubated with 40 μM NphT for 20 hours. NphT was purified using size exclusion chromatography, and fractions were combined and concentrated to 100 μM using a 500 μL Modified PES VWR Centrifugal Filter, 10 kDa MWCO (VWR). Reactions (100 μL) contained varying combinations of holo NphT (8 μM), D-ribose (50 mM), inositol (50 mM) and butanal (30 mM) in 20 mM HEPES pH 7.5 in D_2O. Reactions proceeded at 50°C for 18 hours before removal from the anaerobic chamber. 500 μL D_2O was added to each sample prior to analysis by NMR. This experiment was performed once.

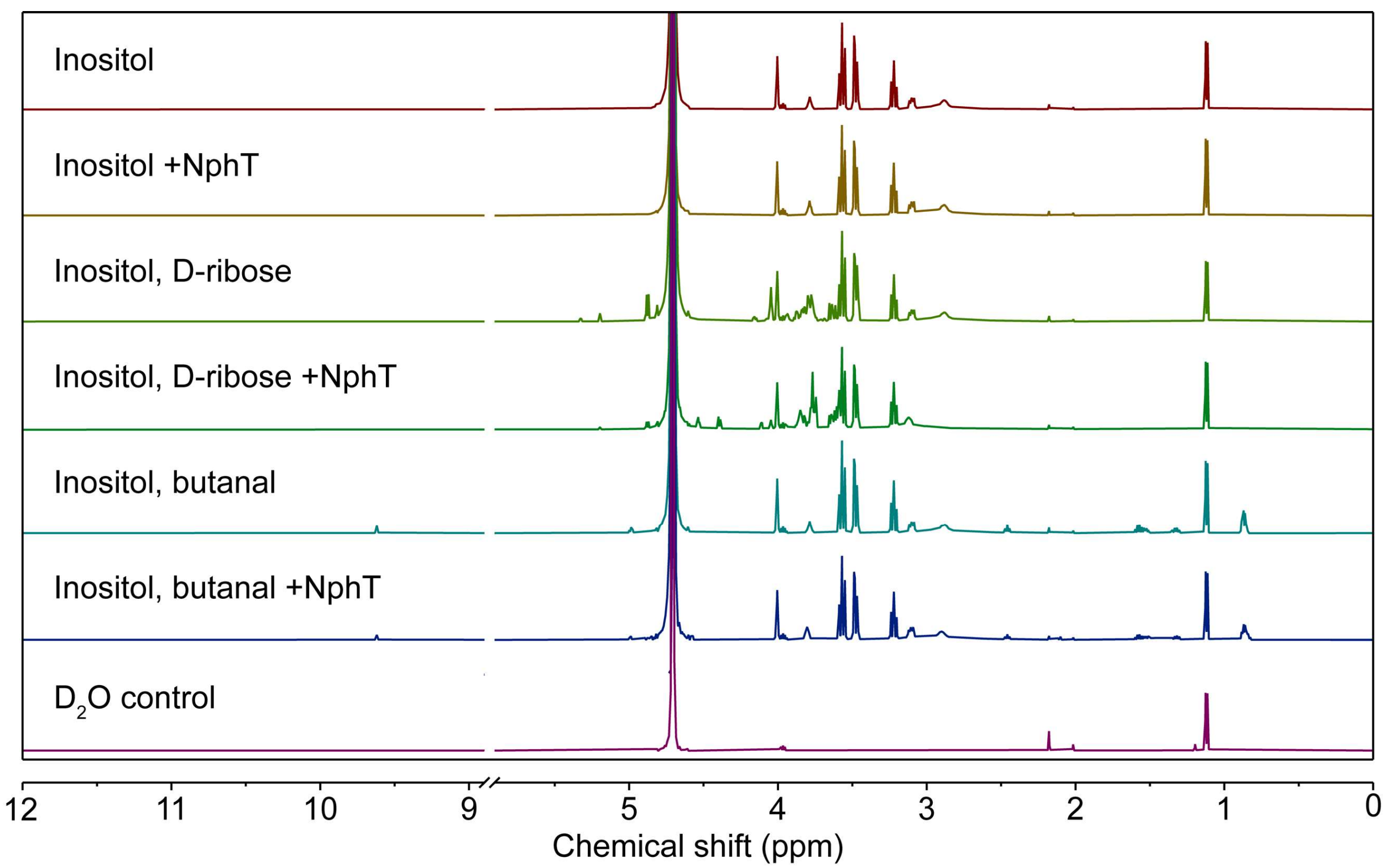

Supplementary Figure 44. SDS-PAGE analysis of purified proteins used in this study. Lane **1**, LarB S127A; **2**, LarC; **3**, LarE; **4**, LarA **5**, *E. coli* AKR; **6**, NphT WT; **7**, NphT K200A; **8**, NphT H144G; **9**, NphT D47A**10**, NphT H106A; **11**, NphT K75A; **12**, NphT W218A; **13**, NphT Y52A, and **14**, NphT Y52F. Protein purification of NphT variants and *E. coli* AKR was performed once. Purification of NphT was performed n=4 times with similar results. LarB S127A, LarC, and LarE were purified more than n=5 times each with similar results.

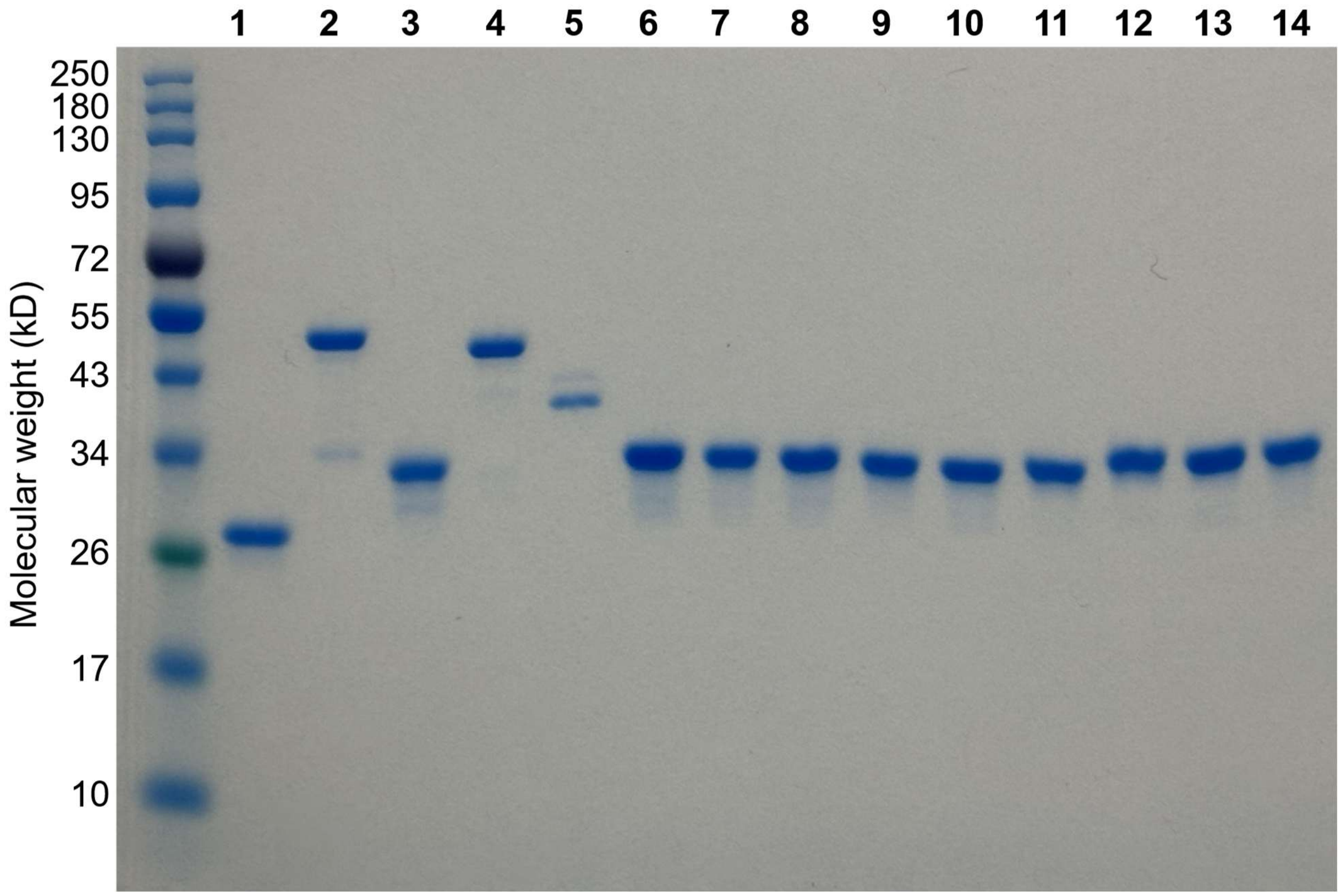

Supplementary Figure 45. Raw gel image. Raw gel image for Supplementary Figure 44.

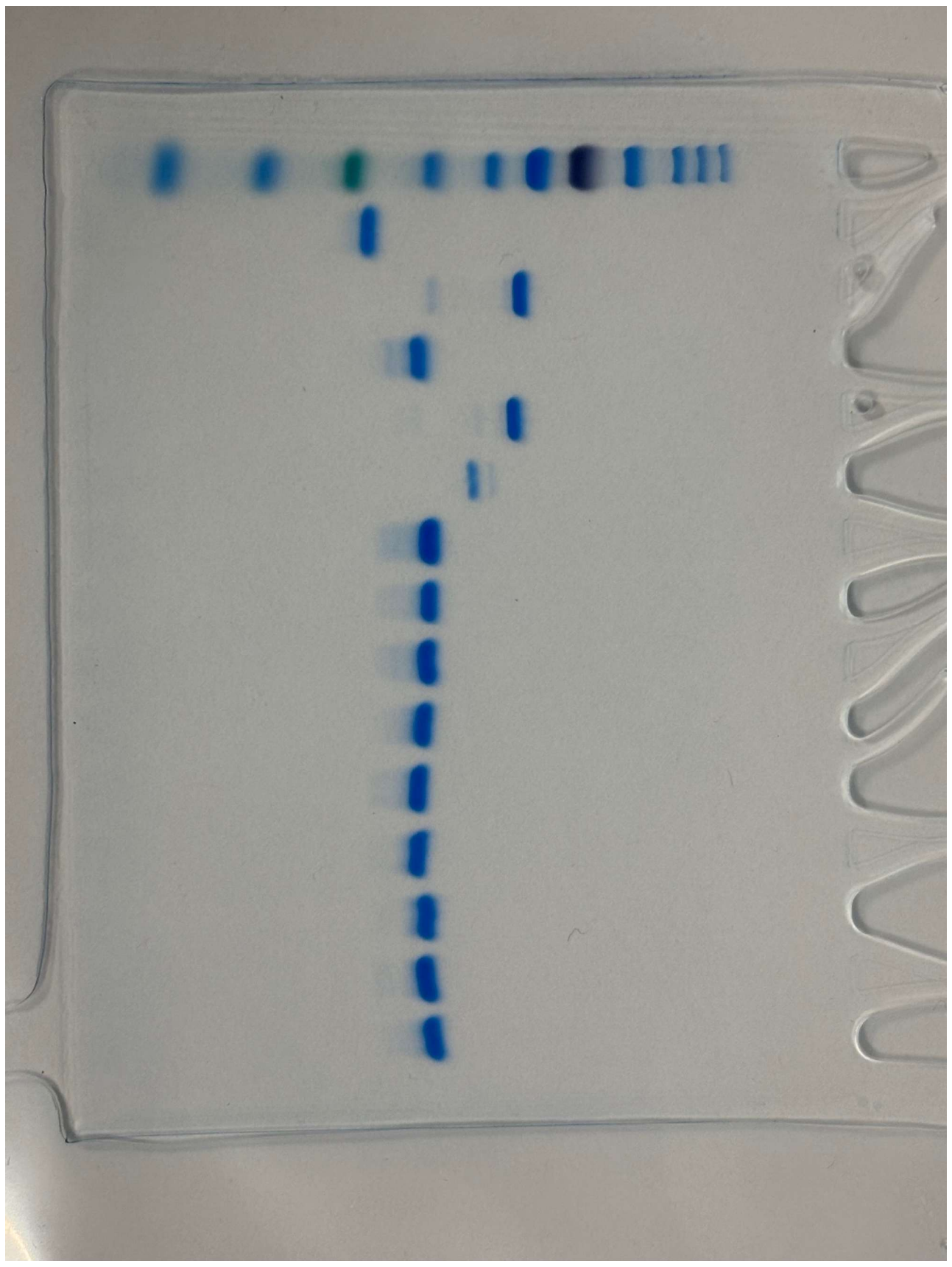

Supplementary Note 1. Discussion of potential NphT mechanisms. Sugars exist as equilibrium mixtures of the linear aldose, α-pyranose, β-pyranose, α-furanose, and β-furanose tautomers. As a result, it is challenging to determine which of these tautomers is the direct substrate for NphT. While we cannot conclude the mechanism, we consider several possibilities below:

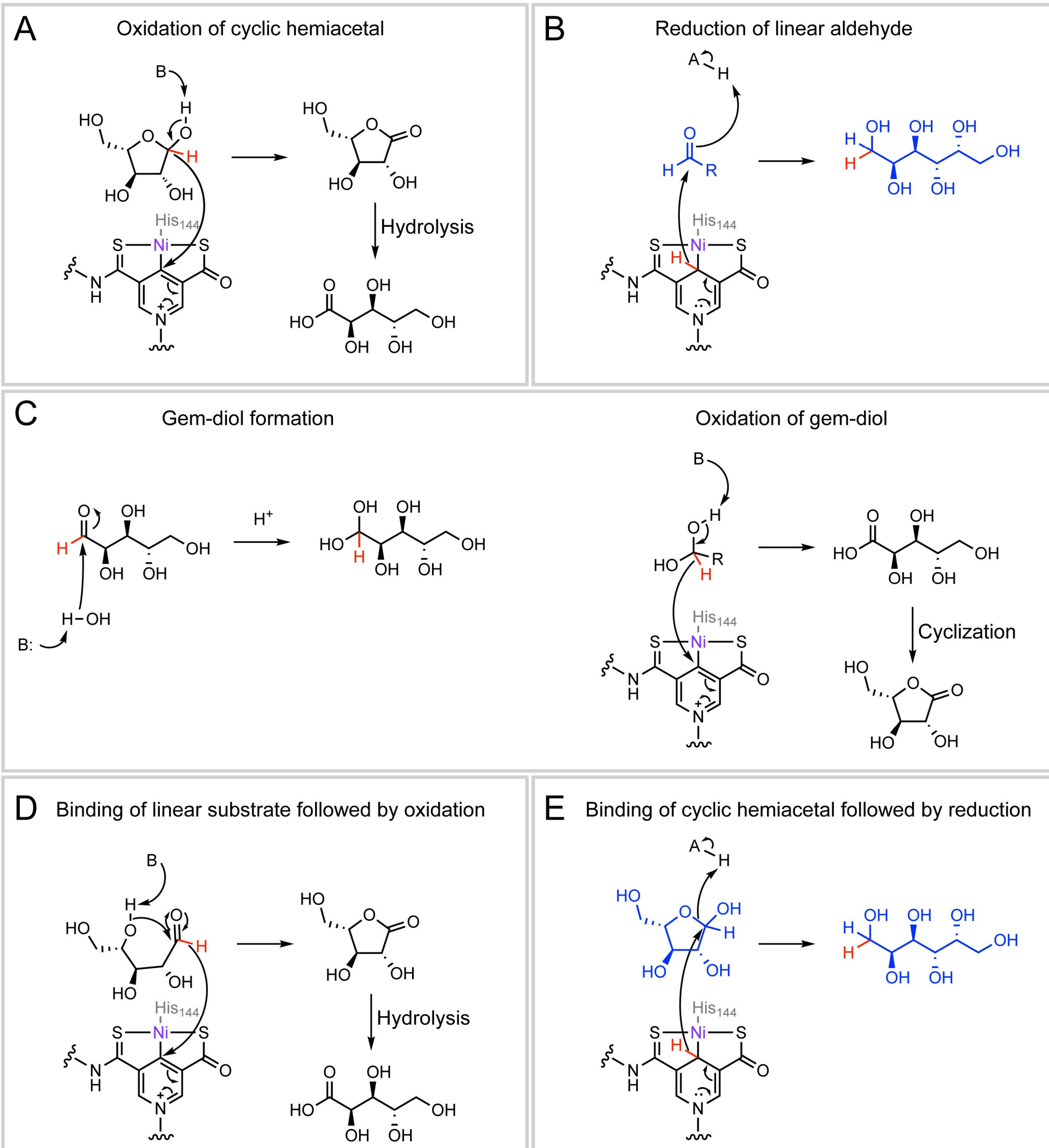

Possible mechanisms for NphT-catalyzed redox reactions on sugar substrates. (A) Oxidation of a pyranose or furanose cyclic hemiacetal. (B) Reduction of linear aldehyde. (C) Oxidation of linear gem-diol. (D) Oxidation of linear substrate. (E) Reduction of cyclic substrate.

Oxidation of cyclic hemiacetal (A). One possible tautomer for oxidation is the cyclic hemiacetal. NAD^+-dependent sugar dehydrogenases, such as D-galactose dehydrogenase (GDH) have been shown to specifically oxidize the β-galactopyranose to yield D-galactono-1,5-lactone, which rearranges to D-galactono-1,4-lactone[22]. The lactone can hydrolyze over time to yield the acid. In our experiments, we observe the formation of 1,4-lactone species at early time points, which hydrolyzes to yield the aldonic acids (**Supplementary Fig. 27**). This experimental observation could be explained by direct oxidation of the furanose or by oxidation of the pyranose followed by rapid rearrangement of the lactone and subsequent hydrolysis, as reported in the GDH enzymes.

Reduction of linear aldehyde (B). One possible substrate for reduction is the linear aldose form of the sugar. To investigate this, we experimentally probed whether a linear substrate could be reduced by our enzyme. This cannot be tested with ribose alone since the tautomers are in equilibrium, so we mixed ribose with butyraldehyde, which cannot cyclize. Since the linear aldose fraction of ribose is low (0.05%)[23], we reasoned that mM concentrations of butyraldehyde could compete as a linear hydride acceptor, while ribose could still serve as a hydride donor. We found that when mixed with ribose, butyraldehyde can be reduced to butanol (**Supplementary Fig. 42**). These data support the occurrence of transhydrogenation and indicate that linear substrates can be reduced by our enzyme. Canonical NADPH-dependent aldo-keto reductases are reported to reduce linear substrates[3,24], so these results may be consistent with that observation.

Gem-diol oxidation (C). The linear aldehyde can exist as the hydrate (gem-diol) in solution. The gem-diol could bind in the enzyme active site and serve as a substrate for oxidation. There is synthetic and enzymatic precedent for oxidation of gem-diol species during conversion of aldehydes to carboxylic acids[25,26]. While the direct formation of the aldonic acid is not consistent with our observation of a cyclic lactone product that hydrolyzes over time (**Supplementary Fig. 27**), we docked the gem-diol species into the active site of NphT and found that they can be accommodated in the enzyme active site.

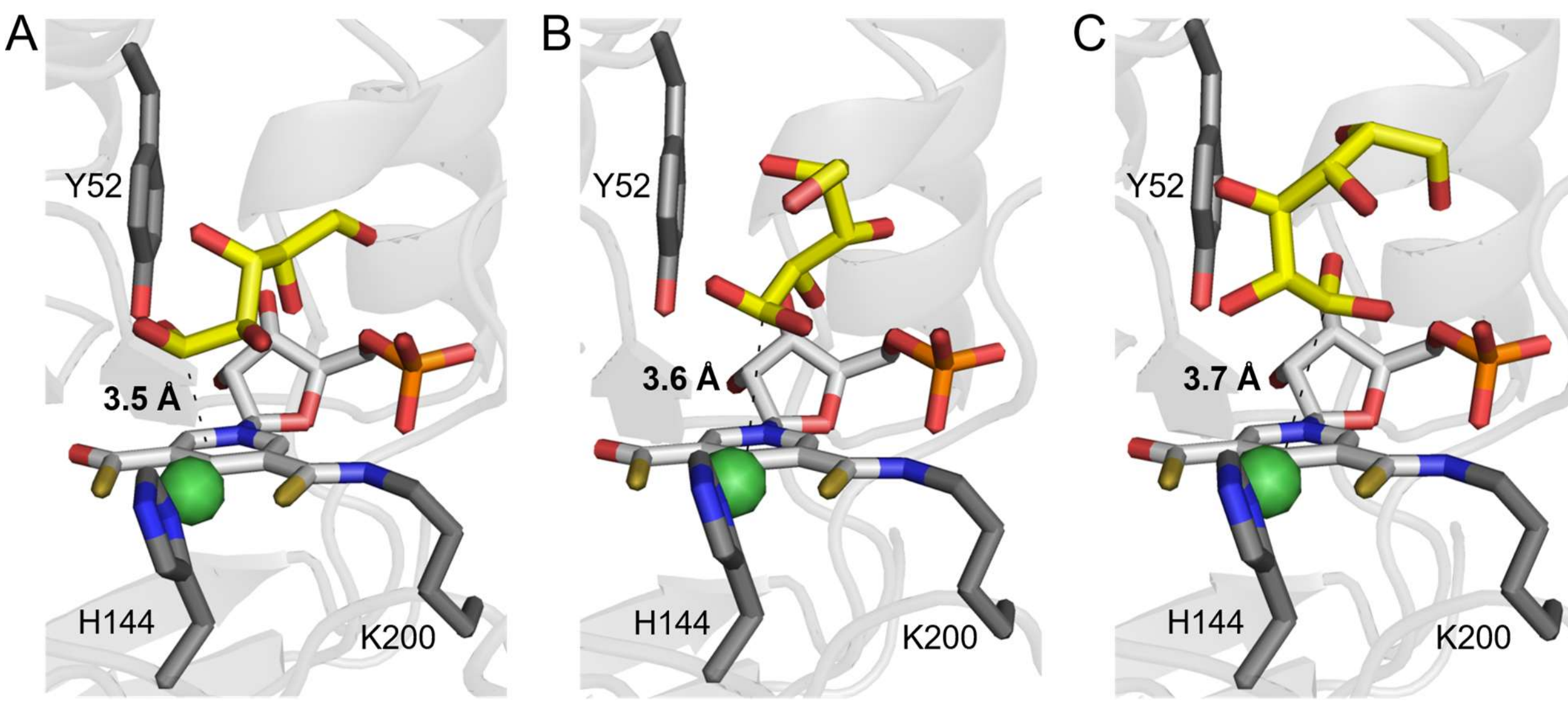

Docking of gem-diol sugars into NphT. (A) D-ribose. (B) L-arabinose. (C). D-galactose.

Oxidation of linear substrate (D). Another possibility is that the linear aldose could bind in the enzyme active site. Next, a hydroxyl group from the sugar could attack the aldehyde to form the hemiacetal inside the active site. There are enzymes that oxidize aldehydes in mechanistically

similar manners. For example, glyceraldehyde 3-phosphate dehydrogenase oxidizes an aldehyde by first generating a thiohemiacetal using an active site cysteine residue[27]. Close examination of the NphT active site shows that there are no cysteine residues nearby, so this thiohemiacetal mechanism is unlikely in NphT. We cannot rule out binding of the linear aldehyde and generation of the cyclic hemiacetal upon attack of the sugar hydroxyl within the active site, but we note that the cyclic hemiacetal species are already much more abundant in solution (see table below) relative to the linear aldehyde (0.05% for ribose).

Reduction of cyclic hemiacetal (E). Another possibility is nucleophilic attack by a hydride onto the anomeric carbon of a cyclic hemiacetal, leading to reduced alcohol formation. This nucleophilic substitution pathway is less favorable than the others because the anomeric carbon of a hemiacetal is a poor electrophile compared to the carbonyl of a free aldehyde. Furthermore, the ring oxygen would serve as a leaving group, leaving as an alkoxide, which is a poor leaving group. However, this could potentially be alleviated by protonation by a residue in the active site. There is so far no precedent for this mechanism in aldose reductases, which have thus far been shown to reduce linear aldehyde substrates[12].

Comments on substrate selectivity. Because it is unclear whether linear or cyclic sugar tautomers are the direct substrates for NphT, and because all sugars were accommodated within the large, solvent exposed active site, our docking studies (AutodockVina[17]) were inconclusive in explaining the observed differences in activity between D-galactose, L-arabinose, and D-ribose, and the weak substrates D-glucose and L-rhamnose (**Supplementary Fig. 37**). One possibility is that these sugars differ in the equilibrium fractions of linear aldoses, α-pyranose, β-pyranose, α-furanose, and β-furanose species[23], which could impact the rate of consumption depending on which is used by the enzyme. For example, we observe the highest activity on D-ribose, which has the highest aldehyde fraction, and the lowest activity on D-glucose, which has the lowest aldehyde fraction of the five substrates below (25-fold lower than D-ribose). This trend holds across the intermediate substrates as well. Differences in the furanose abundances could also be important if the furanose is one of the relevant species. Future structural studies capturing NphT in complex with bound substrates may help resolve this question.

The composition (%) of sugars from Ref.[23]

	Pyranose		Furanose		
**Sugar**	**α**	**β**	**α**	**β**	**Aldehyde**
Ribose	21.5	58.5	6.5	13.5	0.05
Arabinose	60	35.5	2.5	2	0.03
Galactose	30	64	2.5	3.5	0.02
Rhamnose	60	40	---	---	0.006
Glucose	38	62	---	---	0.002

In light of these considerations, we explicitly indicate in Figs. 3f and 5d that we cannot yet conclude which tautomers are the direct substrates for NphT.

References

1. Gerlt, J. A. *et al.* Enzyme Function Initiative-Enzyme Similarity Tool (EFI-EST): A web tool for generating protein sequence similarity networks. *Biochimica et Biophysica Acta (BBA) - Proteins and Proteomics* **1854**, 1019–1037 (2015).
2. van Kempen, M. *et al.* Fast and accurate protein structure search with Foldseek. *Nat Biotechnol* **42**, 243–246 (2024).
3. Penning, T. M. The aldo-keto reductases (AKRs): Overview. *Chem Biol Interact* **234**, 236–246 (2015).
4. Gibson, D. G. *et al.* Enzymatic assembly of DNA molecules up to several hundred kilobases. *Nat Methods* **6**, 343–345 (2009).
5. Gasteiger, E. *et al.* ExPASy: The proteomics server for in-depth protein knowledge and analysis. *Nucleic acids research* **31**, 3784–8 (2003).
6. Chatterjee, S., Nevarez, J. L., Rankin, J. A., Hu, J. & Hausinger, R. P. Structure of the LarB-Substrate Complex and Identification of a Reaction Intermediate during Nickel-Pincer Nucleotide Cofactor Biosynthesis. *Biochemistry* **62**, 3096–3104 (2023).
7. Desguin, B. *et al.* Biosynthesis of the nickel-pincer nucleotide cofactor of lactate racemase requires a CTP-dependent cyclometallase. *J Biol Chem* **293**, 12303–12317 (2018).
8. Kabsch, W. XDS. *Acta Crystallogr D Biol Crystallogr* **66**, 125–132 (2010).
9. Winn, M. D. *et al.* Overview of the CCP4 suite and current developments. *Acta Crystallogr D Biol Crystallogr* **67**, 235–242 (2011).
10. Adams, P. D. *et al.* PHENIX: a comprehensive Python-based system for macromolecular structure solution. *Acta Crystallogr D Biol Crystallogr* **66**, 213–221 (2010).
11. Abramson, J. *et al.* Accurate structure prediction of biomolecular interactions with AlphaFold 3. *Nature* **630**, 493–500 (2024).
12. Emsley, P., Lohkamp, B., Scott, W. G. & Cowtan, K. Features and development of Coot. *Acta Crystallogr D Biol Crystallogr* **66**, 486–501 (2010).
13. Pettersen, E. F. *et al.* UCSF Chimera--a visualization system for exploratory research and analysis. *J Comput Chem* **25**, 1605–1612 (2004).
14. The PyMOL Molecular Graphics System, Version 1.2r3pre, Schrödinger, LLC.
15. Kay, L. E., Nicholson, L. K., Delaglio, F., Bax, A. & Torchia, D. A. Pulse sequences for removal of the effects of cross correlation between dipolar and chemical-shift anisotropy relaxation mechanisms on the measurement of heteronuclear *T*1 and *T*2 values in proteins. *Journal of Magnetic Resonance (1969)* **97**, 359–375 (1992).
16. Yamazaki, T., Muhandiram, R. & Kay, L. E. NMR Experiments for the Measurement of Carbon Relaxation Properties in Highly Enriched, Uniformly 13C,15N-Labeled Proteins: Application to 13C.alpha. Carbons. *J. Am. Chem. Soc.* **116**, 8266–8278 (1994).
17. Trott, O. & Olson, A. J. AutoDock Vina: improving the speed and accuracy of docking with a new scoring function, efficient optimization, and multithreading. *J Comput Chem* **31**, 455–461 (2010).
18. Eberhardt, J., Santos-Martins, D., Tillack, A. F. & Forli, S. AutoDock Vina 1.2.0: New Docking Methods, Expanded Force Field, and Python Bindings. *J Chem Inf Model* **61**, 3891–3898 (2021).
19. Morris, G. M. *et al.* AutoDock4 and AutoDockTools4: Automated docking with selective receptor flexibility. *J Comput Chem* **30**, 2785–2791 (2009).

20. Santos-Martins, D. *et al.* Meeko: Molecule Parametrization and Software Interoperability for Docking and Beyond. *J Chem Inf Model* **65**, 13045–13050 (2025).
21. Desai, K. K. & Miller, B. G. A metabolic bypass of the triosephosphate isomerase reaction. *Biochemistry* **47**, 7983–7985 (2008).
22. Ueberschär, K. H., Blachnitzky, E. O. & Kurz, G. Reaction mechanism of D-galactose dehydrogenases from Pseudomonas saccharophila and Pseudomonas fluorescens. Formation and rearrangemnt of aldono-1,5-lactones. *Eur J Biochem* **48**, 389–405 (1974).
23. Angyal, S. J. The Composition of Reducing Sugars in Solution. in *Advances in Carbohydrate Chemistry and Biochemistry* (eds. Tipson, R. S. & Horton, D.) vol. 42 15–68 (Academic Press, 1984).
24. Balestri, F. *et al.* L-Idose: an attractive substrate alternative to D-glucose for measuring aldose reductase activity. *Biochem Biophys Res Commun* **456**, 891–895 (2015).
25. Rocek, J. & Ng, C.-S. Chromium(IV) oxidation of aliphatic aldehydes. *J. Am. Chem. Soc.* **96**, 1522–1529 (1974).
26. Puetz, H., Puchľová, E., Vranková, K. & Hollmann, F. Biocatalytic Oxidation of Alcohols. *Catalysts* **10**, 952 (2020).
27. Wolfson-Stofko, B., Hadi, T. & Blanchard, J. S. Kinetic and mechanistic characterization of the glyceraldehyde 3-phosphate dehydrogenase from Mycobacterium tuberculosis. *Arch Biochem Biophys* **540**, 53–61 (2013).

Supplementary Code 1. R script for the bioinformatic identification of nickel pincer enzymes to keep LarC accessions containing LarB and LarE and removing genomes containing LarA.

```
### This script processes a genome neighborhood diagram (.sqlite file) with
larC as the anchor gene, isolating larC accessions whose neighboring genes
include larB and larE but exclude larA.

library(RSQLite)
library(DBI)
library(dplyr)
library(stringr)
library(openxlsx)

# Extract data from .sqlite file ####

# Open .sqlite file from a given genome neighborhood diagram
con <- dbConnect(RSQLite::SQLite(), "path/to/input.sqlite")

# List all tables in the database
tables <- dbListTables(con)
tables

# Extract tables from .sqlite
# neighbors and attributes are our primary ones of interest
neighbors2tibble <- "SELECT * FROM neighbors"
attributes2tibble <- "SELECT * FROM attributes"

# Retrieve table data as tibbles
neighbors_tibble <- dbGetQuery(con, neighbors2tibble) %>% as_tibble()
attributes_tibble <- dbGetQuery(con, attributes2tibble) %>% as_tibble()

# Create larBCE no larA UniProt accession list by filtering ####
# this is for larC as anchor

# Filter 'neighbors_tibble' to include anything with larB, larE, larA
filtered_neighbors <- neighbors_tibble %>%
  filter(str_detect(family_desc, regex("larB|larE|larA", ignore_case = TRUE))
|
          str_detect(ipro_family_desc, regex("larB|larE|larA", ignore_case =
TRUE)))


# Create new tibble to remove 'gene_key' entries in filtered_neighbors where
larA is found
filtered_out_larA <- neighbors_tibble %>%
  filter(str_detect(family_desc, regex("larA", ignore_case = TRUE)) |
          str_detect(ipro_family_desc, regex("larA", ignore_case = TRUE)))


# Remove rows in filtered_neighbors that have 'gene_key' values present in
filtered_out_larA
filtered_neighbors_noA <- filtered_neighbors %>%
  anti_join(filtered_out_larA, by = "gene_key")

# Count the occurrences of each 'gene_key' in filtered_neighbors_noA
# and filter to only include counts greater than or equal to 2
```

```
# b/c we want both larB and larE to be present
filtered_neighbors_noA_counts <- filtered_neighbors_noA %>%
  count(gene_key) %>%
  filter(n >= 2)

# Filter 'filtered_attributes' based on 'sort_key' presence and occurrence
count
# This will contain final list of LarC accessions to put into EFI-EST
filtered_attributes <- attributes_tibble %>%
  semi_join(filtered_neighbors_noA_counts, by = c("sort_key" = "gene_key"))

# Set working directory and save list as excel file
setwd("path/to/directory")
write.xlsx(filtered_attributes, file = "output.xlsx")

# Optional: find all larBCE neighbors ####

# This will sort neighbors_tibble to only show neighbors based on
filtered_attributes
all_bce_neighbors <- neighbors_tibble %>%
  semi_join(filtered_attributes, by = "id") %>%
  mutate(anchor_accession = filtered_attributes$accession[match(id,
filtered_attributes$id)]) %>%
  mutate(cluster_num = filtered_attributes$cluster_num[match(id,
filtered_attributes$id)]) %>%
  mutate(anchor = as.character(c("larc")))
```

Supplementary Code 2. R script for profiling Pfam occurrences.

```
### This script processes a genome neighborhood diagram (.sqlite file)
generated with larC as the anchor gene where larBE but not larA are present
among the surrounding genes. It generates a table of Pfam occurrences for all
neighboring genes of the larC anchor.

library(RSQLite)
library(DBI)
library(dplyr)
library(tidyr)
library(stringr)
library(openxlsx)


# Extract data from .sqlite file ####

# Open .sqlite file from a given genome neighborhood diagram
con <- dbConnect(RSQLite::SQLite(), "path/to/input.sqlite")

# List all tables in the database
tables <- dbListTables(con)
tables

# Extract tables from .sqlite
# neighbors and attributes are our primary ones of interest
neighbors2tibble <- "SELECT * FROM neighbors"
```

```
attributes2tibble <- "SELECT * FROM attributes"

# Retrieve table data as tibbles
neighbors_tibble <- dbGetQuery(con, neighbors2tibble) %>% as_tibble()
attributes_tibble <- dbGetQuery(con, attributes2tibble) %>% as_tibble()

# Create Pfam list ####

# New tibble that splits pfams from neighbors_tibble
all_pfams_bce <- neighbors_tibble %>%
  mutate(
    family = str_split(family, "-"),
    family_desc = str_split(family_desc, ";")
  ) %>%
  unnest(c(family, family_desc))


# Count number of unique families present in all_pfams_bce
unique_pfam_count_bce <- all_pfams_bce %>%
  summarise(unique_pfam_count_bce = n_distinct(family))
print(unique_pfam_count_bce)

# Make tibble with counts of each Pfam with description and sorts in
descending order
# AKR superfamily is PF00248
pfam_counts_total_bce <- all_pfams_bce %>%
  count(family) %>%
  arrange(desc(n)) %>%
  left_join(
    all_pfams_bce %>% distinct(family, family_desc),
    by = "family"
  )

# From tibble of counts of each Pfam with description:
# removes LarE (PF02540), LarB (PF00731), LarC (PF01969), and Pfam of "none"
and sorts in descending order
pfam_counts_filtered_bce <- pfam_counts_total_bce %>%
  filter(!family %in% c("PF02540", "PF00731", "PF01969", "none")) %>%
  arrange(desc(n))

# Set working directory and save list as excel file
setwd("path/to/directory")
write.xlsx(pfam_counts_total_bce, file = "output1.xlsx")
write.xlsx(pfam_counts_filtered_bce, file = "output2.xlsx")
```

Supplementary Code 3. R script for annotation of AKRs containing the histidine/lysine motif.

```
###This file takes in an alignment (FASTA FORMAT). By providing the known
character positions of NphT H144 and K200 in the alignment, it extracts the
UniProt accession numbers of additional sequences containing the
histidine/lysine motif.

library(Biostrings)
```

```
# Load aligned FASTA
fasta <- readAAStringSet("path/to/inputMSA.fasta")

# Convert to character matrix
seq_matrix <- as.matrix(fasta)

# Filter by conditions at character positions 14688 and 17904
# these are the locations of H144 and K200 of histidine/lysine motif in the
MSA, but can alter character positions based on custom MSA input

matching_seqs <- fasta[which(seq_matrix[, 14688] == "H" & seq_matrix[, 17904]
== "K")]

# Save output list of Uniprot accessions with histidine/lysine motif
# note: this list can be used as a selection input in Cytoscape
writeXStringSet(matching_seqs, filepath = "output.fasta")
accessions <- sub("^[^|]*\\|[^|]*\\|([^_]*)_.*", "\\1", names(matching_seqs))

# used to isolate accession number
writeLines(accessions, "output_accessions.txt")
```

Supplementary Code 4. R script for counting iron and nickel enzymes.

```
###This file takes the spreadsheet of PDB entries containing iron or nickel
(.xlsx format) and refines it to retain entries whose corresponding UniProt
entry includes the keyword "nickel" or "iron" in the ligand section, also
removing duplicate EC numbers to ensure functionally distinct enzymes are
counted only once.

library(tidyverse)
library(readxl)

# Open excel file ####
MegaPDBmetals <- read_excel path/to/input.xlsx")

# First pass metal count ####
ni_entries <- MegaPDBmetals %>% filter(Metal == "NI")
fe_entries <- MegaPDBmetals %>% filter(Metal == "FE")

# Extract Uniprot entries with nickel or iron keyword ####

library(httr)
library(jsonlite)
library(dplyr)

# Function: check if UniProt entry has the keyword "Nickel" in ligand section
has_nickel_keyword <- function(uniprot_id) {
  url <- paste0("https://rest.uniprot.org/uniprotkb/", uniprot_id, ".json")
  res <- GET(url)
  if (status_code(res) != 200) return(FALSE)

  data <- fromJSON(content(res, as = "text", encoding = "UTF-8"))

  if (!"keywords" %in% names(data)) return(FALSE)

  any(grepl("nickel", data$keywords$name, ignore.case = TRUE))
}
```

```
# Function: check if UniProt entry has the keyword "Iron" in ligand section
has_iron_keyword <- function(uniprot_id) {
  url <- paste0("https://rest.uniprot.org/uniprotkb/", uniprot_id, ".json")
  res <- GET(url)
  if (status_code(res) != 200) return(FALSE)

  data <- fromJSON(content(res, as = "text", encoding = "UTF-8"))

  if (!"keywords" %in% names(data)) return(FALSE)

  any(grepl("iron", data$keywords$name, ignore.case = TRUE))
}

# NICKEL: run has_nickel_keyword function and remove rows that have no EC
number and duplicate ECs
ni_refined <- ni_entries %>%
  filter(sapply(Uniprot, has_nickel_keyword)) %>%
  filter(!is.na(EC_numbers) & EC_numbers != "") %>%   # remove blank ECs
  separate_rows(EC_numbers, sep = ",\\s*") %>%        # split on comma
  filter(grepl("^\\d+\\.\\d+\\.\\d+\\.(\\d+|-)$", EC_numbers)) %>%  # keep
x.x.x.x or x.x.x.- EC numbers
  distinct(EC_numbers, .keep_all = TRUE)              # remove duplicate ECs

# IRON: run has_iron_keyword function and remove rows that have no EC number
and duplicate ECs
fe_refined <- fe_entries %>%
  filter(sapply(Uniprot, has_iron_keyword)) %>%
  filter(!is.na(EC_numbers) & EC_numbers != "") %>%   # remove blank ECs
  separate_rows(EC_numbers, sep = ",\\s*") %>%        # split on comma
  filter(grepl("^\\d+\\.\\d+\\.\\d+\\.(\\d+|-)$", EC_numbers)) %>%  # keep
x.x.x.x or x.x.x.- EC numbers
  distinct(EC_numbers, .keep_all = TRUE)              # remove duplicate ECs

# Optional: save list as excel file ####
library(openxlsx)
setwd("path/to/directory")
write.xlsx(ni_refined, file = "output_ni.xlsx")
write.xlsx(fe_refined, file = "output_fe.xlsx")
```
