# Supplementary material for: Discovery and characterization of a nickel enzyme family that catalyses intermolecular hydride shuttling
Source: Nat Chem. 2026 Jun 17;18(9):1582–92. doi: 10.1038/s41557-026-02184-9 (PMC13524815; doi:10.1038/s41557-026-02184-9)
Supplement: Supplementary file 3 [file 41557_2026_2184_MOESM11_ESM.zip › SupplementaryFigure43_NMR_inositol/C2601281514_MN16/10/pdata/1/email_C2601281514_MN16_10_1.pdf]

H1_standard.UW D2O /home/meneugebauer/callisto meneugebauer 52

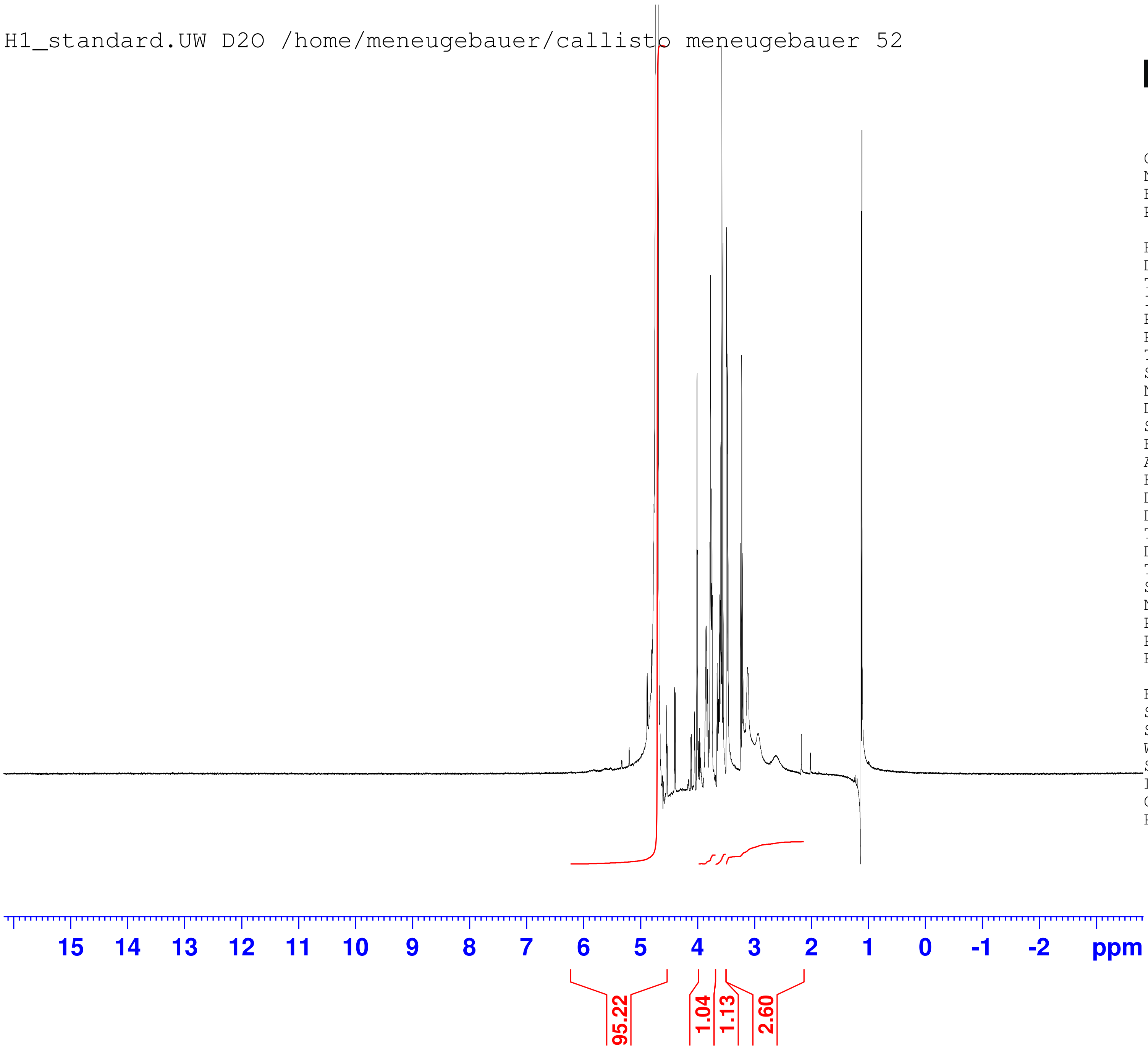

BRUKER

Current Data Parameters
NAME C2601281514_MN16
EXPNO 10
PROCNO 1

F2 - Acquisition Parameters
Date_ 20260128
Time 15.59 h
INSTRUM spect
PROBHD Z119248_0003 (
PULPROG zg30
TD 65536
SOLVENT D2O
NS 18
DS 2
SWH 10000.000 Hz
FIDRES 0.305176 Hz
AQ 3.2767999 sec
RG 30.38
DW 50.000 usec
DE 18.00 usec
TE 298.2 K
D1 1.00000000 sec
TD0 1
SFO1 500.0230878 MHz
NUC1 1H
P0 4.45 usec
P1 13.35 usec
PLW1 15.00000000 W

F2 - Processing parameters
SI 65536
SF 500.0200000 MHz
WDW EM
SSB 0
LB 0.30 Hz
GB 0
PC 1.00
