# Supplementary material for: Discovery and characterization of a nickel enzyme family that catalyses intermolecular hydride shuttling
Source: Nat Chem. 2026 Jun 17;18(9):1582–92. doi: 10.1038/s41557-026-02184-9 (PMC13524815; doi:10.1038/s41557-026-02184-9)
Supplement: Supplementary file 9 [file 41557_2026_2184_MOESM8_ESM.zip › Raw_NMR_data/20250909_13Cquant__1A_4096scans_2/10/pdata/1/email_20250909_13Cquant__1A_4096scans_10_1.pdf]

C13_quant.UW D2O /home/zsemersky/av600 zsemersky 16

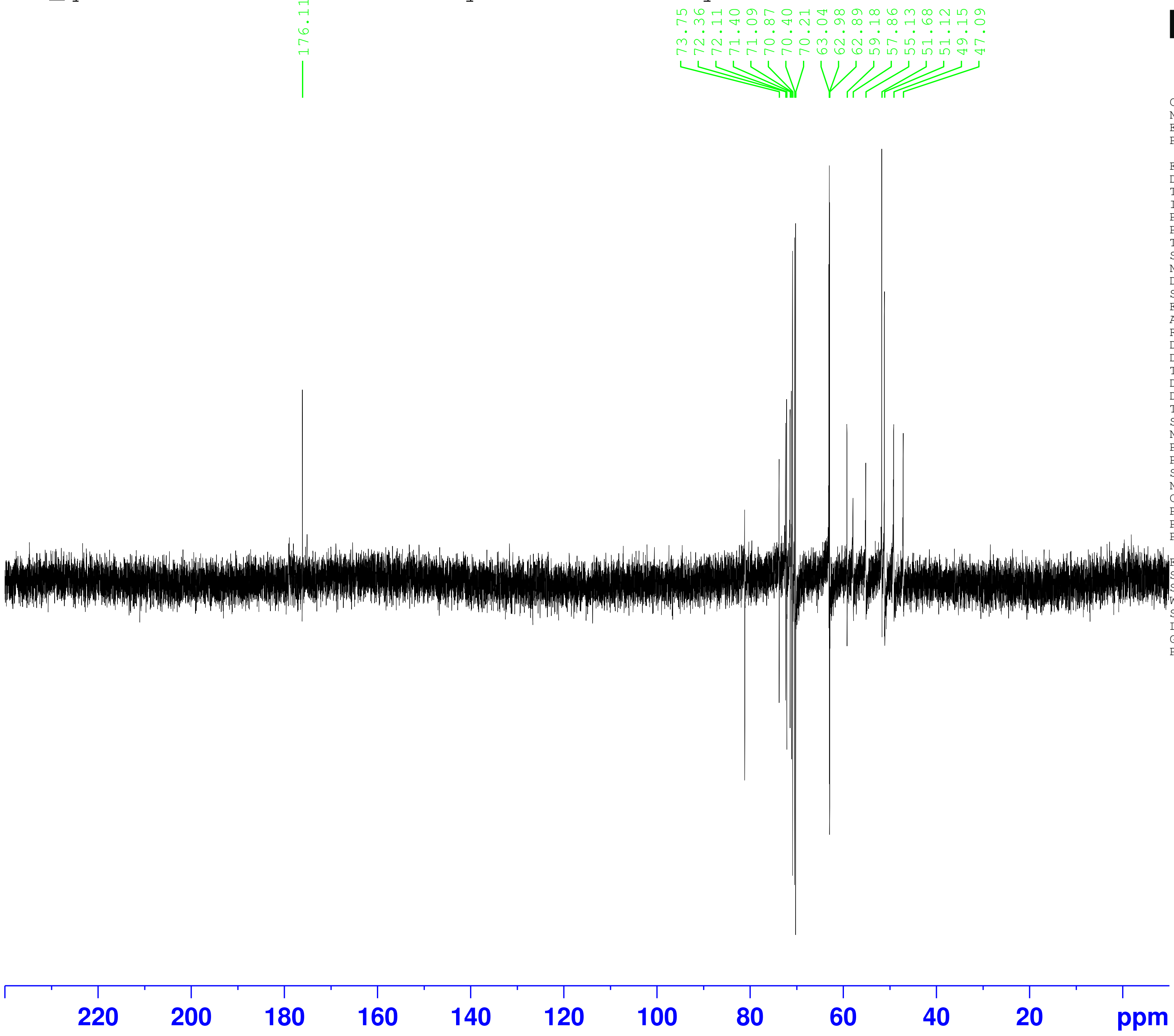

BRUKER

Current Data Parameters
NAME 20250909_13Cquant__1A_4096scans
EXPNO 10
PROCNO 1

F2 - Acquisition Parameters
Date_ 20250910
Time 7.08 h
INSTRUM spect
PROBHD Z150313_0001 (
PULPROG zgig
TD 71424
SOLVENT D2O
NS 4096
DS 4
SWH 42613.637 Hz
FIDRES 1.193258 Hz
AQ 0.8380416 sec
RG 190.75
DW 11.733 usec
DE 20.00 usec
TE 297.1 K
D1 14.00000000 sec
D11 0.03000000 sec
TD0 1
SFO1 150.8288758 MHz
NUC1 13C
P1 11.60 usec
PLW1 90.00000000 W
SFO2 599.7726990 MHz
NUC2 1H
CPDPRG[2 waltz16
PCPD2 70.00 usec
PLW2 7.80000019 W
PLW12 0.09194400 W

F2 - Processing parameters
SI 65536
SF 150.8122865 MHz
WDW EM
SSB 0
LB 1.00 Hz
GB 0
PC 1.40
