# Supplementary material for: Discovery and characterization of a nickel enzyme family that catalyses intermolecular hydride shuttling
Source: Nat Chem. 2026 Jun 17;18(9):1582–92. doi: 10.1038/s41557-026-02184-9 (PMC13524815; doi:10.1038/s41557-026-02184-9)
Supplement: Supplementary file 9 [file 41557_2026_2184_MOESM8_ESM.zip › Raw_NMR_data/20250910_13Cquant_2A_4096scans_2/10/pdata/1/email_20250910_13Cquant_2A_4096scans_10_1.pdf]

C13_quant.UW D2O /home/zsemersky/av600 zsemersky 22

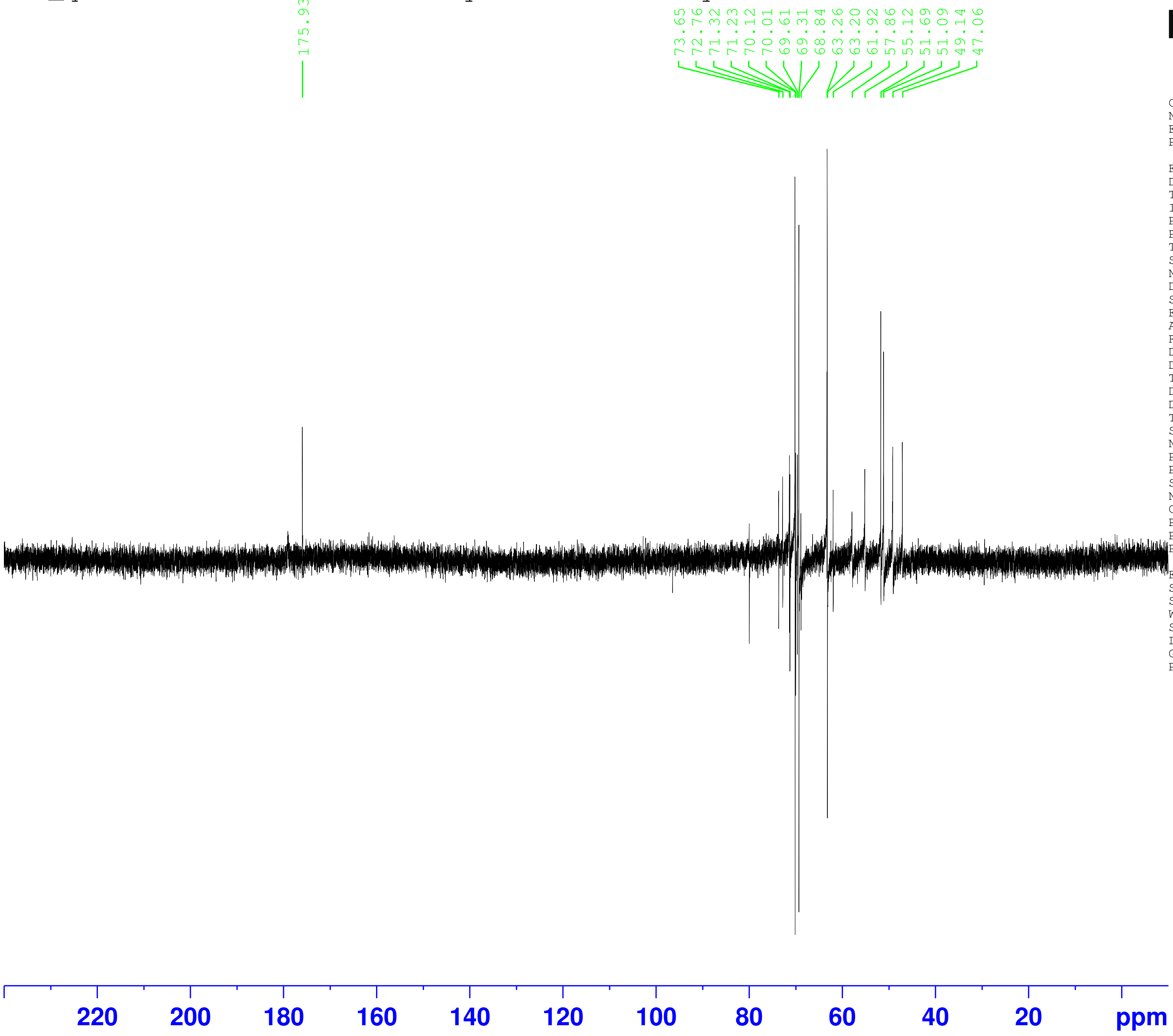

BRUKER

```
Current Data Parameters
NAME      20250910_13Cquant_2A_4096scans
EXPNO                     10
PROCNO                     1

F2 - Acquisition Parameters
Date_               20250911
Time                  2.55 h
INSTRUM                spect
PROBHD    Z150313_0001 (
PULPROG                 zgig
TD                     71424
SOLVENT                  D2O
NS                      4096
DS                         4
SWH               42613.637 Hz
FIDRES             1.193258 Hz
AQ                0.8380416 sec
RG                    190.75
DW                    11.733 usec
DE                     20.00 usec
TE                     297.1 K
D1               14.00000000 sec
D11               0.03000000 sec
TD0                        1
SFO1             150.8288758 MHz
NUC1                     13C
P1                     11.60 usec
PLW1             90.00000000 W
SFO2             599.7726990 MHz
NUC2                      1H
CPDPRG[2             waltz16
PCPD2                  70.00 usec
PLW2              7.80000019 W
PLW12             0.09194400 W

F2 - Processing parameters
SI                     65536
SF               150.8122865 MHz
WDW                       EM
SSB                        0
LB                      1.00 Hz
GB                         0
PC                      1.40
```
