# Supplementary material for: Discovery and characterization of a nickel enzyme family that catalyses intermolecular hydride shuttling
Source: Nat Chem. 2026 Jun 17;18(9):1582–92. doi: 10.1038/s41557-026-02184-9 (PMC13524815; doi:10.1038/s41557-026-02184-9)
Supplement: Supplementary file 10 [file 41557_2026_2184_MOESM9_ESM.zip › SupplementaryFigure41_q13C_NMR_Competition/20251222_Comp_H1_q13C_2/12/pdata/1/email_20251222_Comp_H1_q13C_12_1.pdf]

C13_quant.UW D2O /home/zsemersky/av600 zsemersky 10

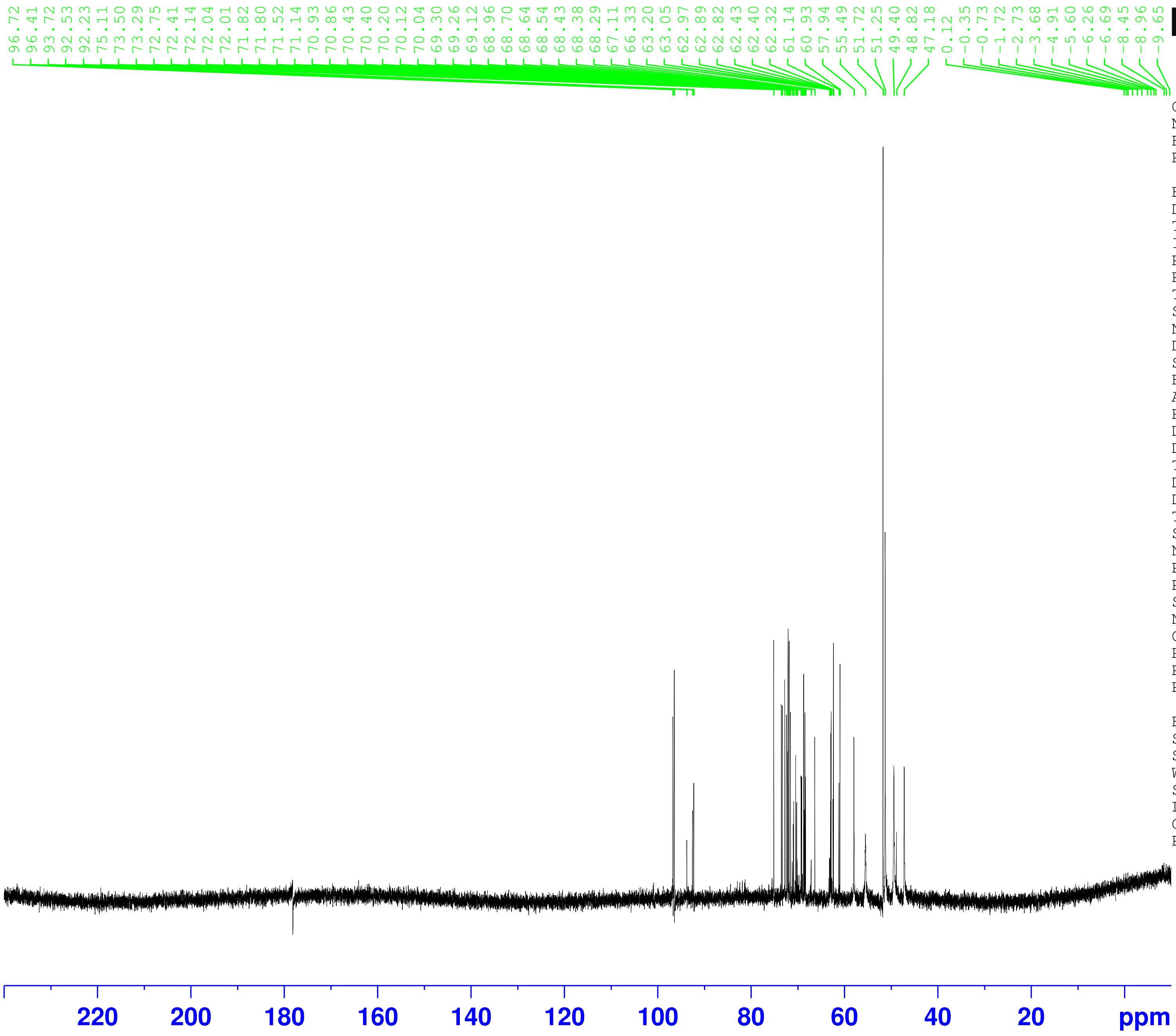


BRUKER

| Current Data Parameters | |
|---|---|
| NAME | 20251222_Comp_H1_q13C |
| EXPNO | 12 |
| PROCNO | 1 |

| F2 - Acquisition Parameters | |
|---|---|
| Date_ | 20251223 |
| Time | 3.09 h |
| INSTRUM | spect |
| PROBHD | Z150313_0001 ( |
| PULPROG | zgig |
| TD | 71424 |
| SOLVENT | D2O |
| NS | 4096 |
| DS | 4 |
| SWH | 42613.637 Hz |
| FIDRES | 1.193258 Hz |
| AQ | 0.8380416 sec |
| RG | 190.75 |
| DW | 11.733 usec |
| DE | 20.00 usec |
| TE | 297.1 K |
| D1 | 14.00000000 sec |
| D11 | 0.03000000 sec |
| TD0 | 1 |
| SFO1 | 150.8288758 MHz |
| NUC1 | 13C |
| P1 | 11.60 usec |
| PLW1 | 90.00000000 W |
| SFO2 | 599.7726990 MHz |
| NUC2 | 1H |
| CPDPRG[2 | waltz16 |
| PCPD2 | 70.00 usec |
| PLW2 | 7.80000019 W |
| PLW12 | 0.09194400 W |

| F2 - Processing parameters | |
|---|---|
| SI | 65536 |
| SF | 150.8122865 MHz |
| WDW | EM |
| SSB | 0 |
| LB | 1.00 Hz |
| GB | 0 |
| PC | 1.40 |
