# Supplementary material for: Discovery and characterization of a nickel enzyme family that catalyses intermolecular hydride shuttling
Source: Nat Chem. 2026 Jun 17;18(9):1582–92. doi: 10.1038/s41557-026-02184-9 (PMC13524815; doi:10.1038/s41557-026-02184-9)
Supplement: Supplementary file 10 [file 41557_2026_2184_MOESM9_ESM.zip › SupplementaryFigure41_q13C_NMR_Competition/20251223_Comp_A1_q13C_4096scans_2/12/pdata/1/email_20251223_Comp_A1_q13C_4096scans_12_1.pdf]

C13_quant.UW D2O /home/zsemersky/av600 zsemersky 1

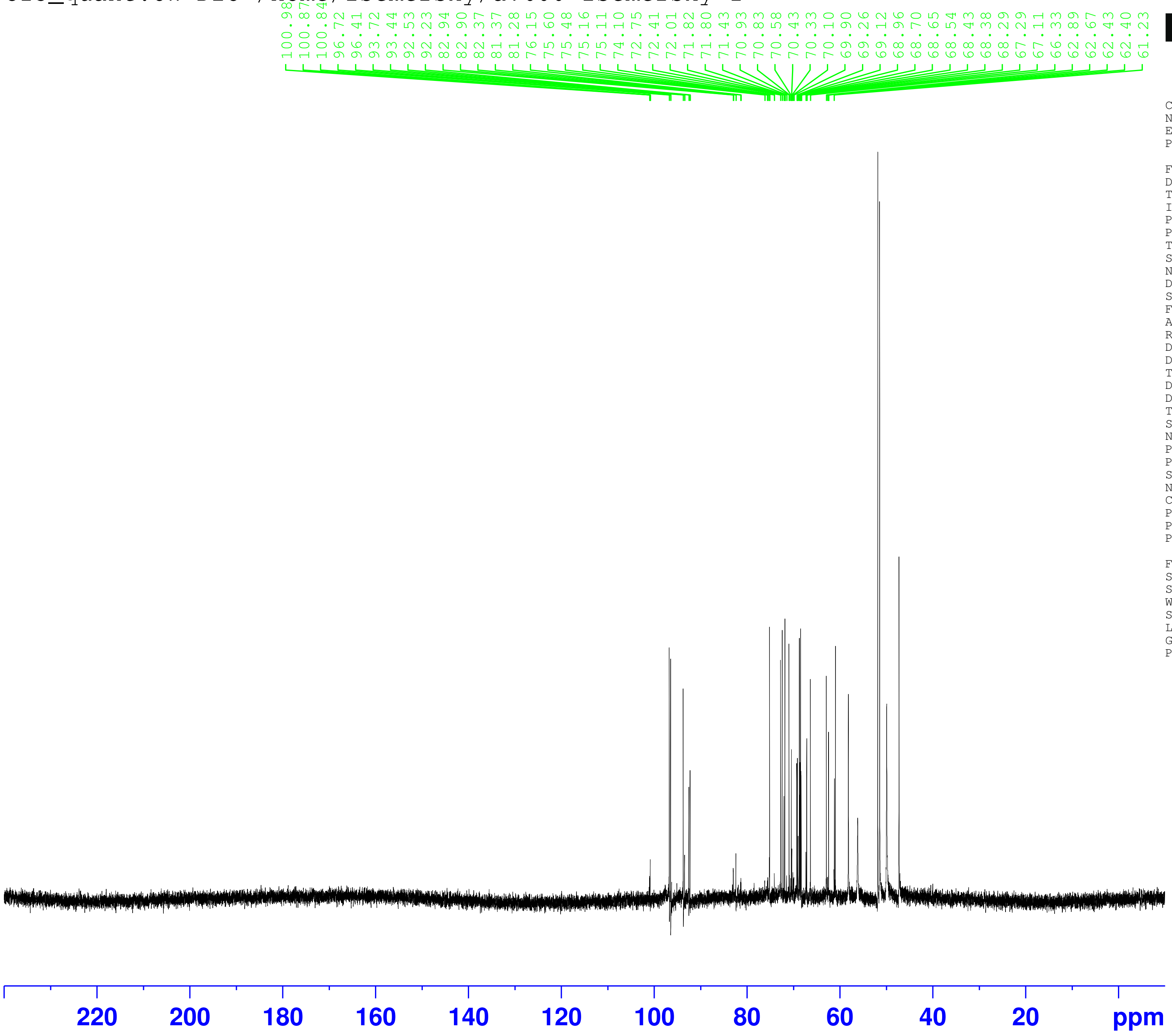

BRUKER

Current Data Parameters

NAME	20251223_Comp_A1_q13C_4096scans
EXPNO	12
PROCNO	1

F2 - Acquisition Parameters

Date_	20251224
Time	10.09 h
INSTRUM	spect
PROBHD	Z150313_0001 (
PULPROG	zgig
TD	71424
SOLVENT	D2O
NS	4096
DS	4
SWH	42613.637 Hz
FIDRES	1.193258 Hz
AQ	0.8380416 sec
RG	190.75
DW	11.733 usec
DE	20.00 usec
TE	297.1 K
D1	14.00000000 sec
D11	0.03000000 sec
TD0	1
SFO1	150.8288758 MHz
NUC1	13C
P1	11.60 usec
PLW1	90.00000000 W
SFO2	599.7726990 MHz
NUC2	1H
CPDPRG[2	waltz16
PCPD2	70.00 usec
PLW2	7.80000019 W
PLW12	0.09194400 W

F2 - Processing parameters

SI	65536
SF	150.8122865 MHz
WDW	EM
SSB	0
LB	1.00 Hz
GB	0
PC	1.40
