# Supplementary material for: Discovery and characterization of a nickel enzyme family that catalyses intermolecular hydride shuttling
Source: Nat Chem. 2026 Jun 17;18(9):1582–92. doi: 10.1038/s41557-026-02184-9 (PMC13524815; doi:10.1038/s41557-026-02184-9)
Supplement: Supplementary file 10 [file 41557_2026_2184_MOESM9_ESM.zip › SupplementaryFigure41_q13C_NMR_Competition/20251224_Comp_H2_q13C_4096scans_2/25/pdata/1/email_20251224_Comp_H2_q13C_4096scans_25_1.pdf]

C13_quant.UW D2O /home/zsemersky/av600 zsemersky 2

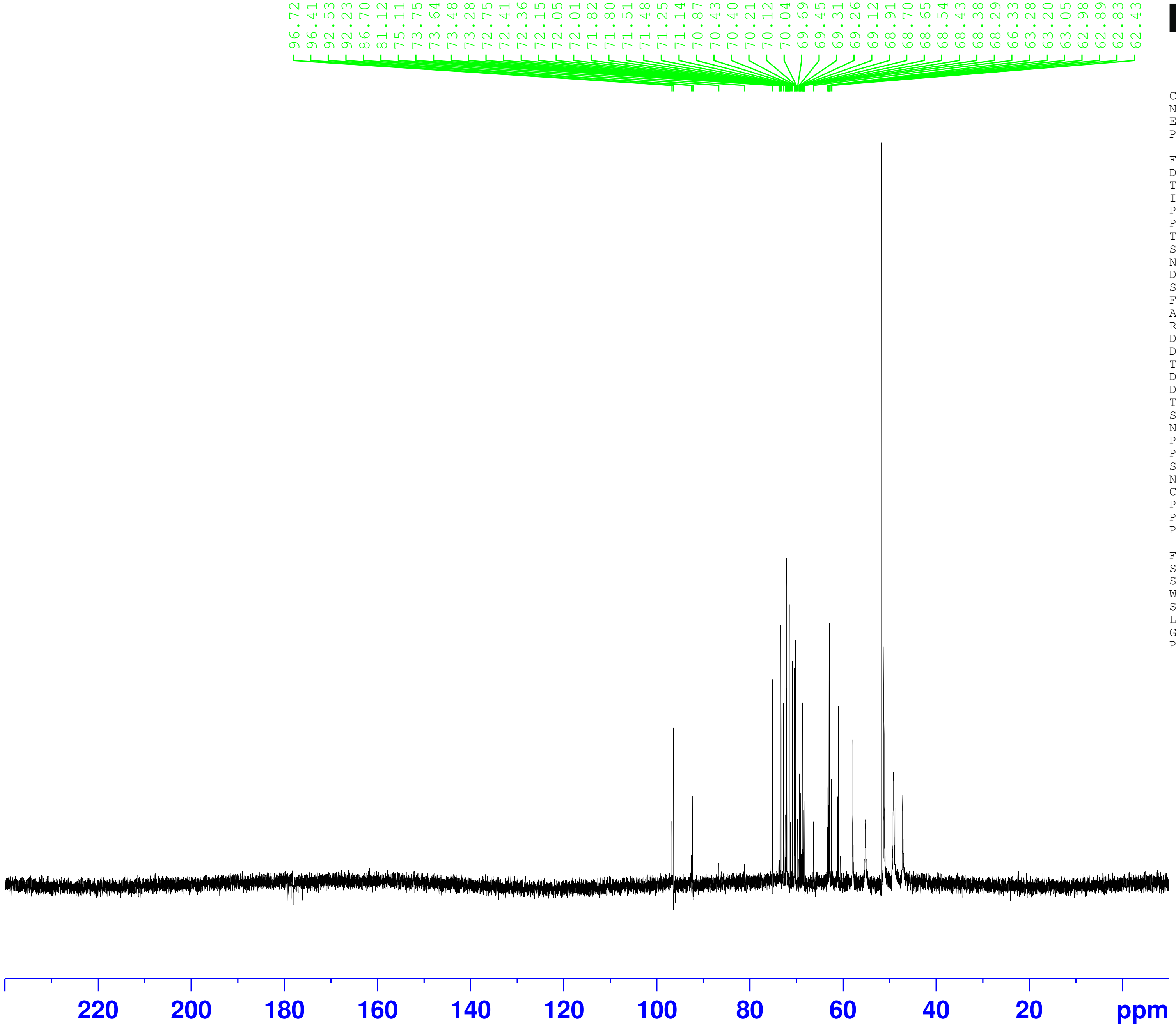

BRUKER

```
Current Data Parameters
NAME      20251224_Comp_H2_q13C_4096scans
EXPNO                         25
PROCNO                         1

F2 - Acquisition Parameters
Date_                   20251225
Time                        3.12 h
INSTRUM                    spect
PROBHD    Z150313_0001 (
PULPROG                     zgig
TD                         71424
SOLVENT                      D2O
NS                          4096
DS                             4
SWH                    42613.637 Hz
FIDRES                  1.193258 Hz
AQ                     0.8380416 sec
RG                        190.75
DW                        11.733 usec
DE                         20.00 usec
TE                         297.1 K
D1                   14.00000000 sec
D11                   0.03000000 sec
TD0                            1
SFO1                 150.8288758 MHz
NUC1                         13C
P1                         11.60 usec
PLW1                 90.00000000 W
SFO2                 599.7726990 MHz
NUC2                          1H
CPDPRG[2                 waltz16
PCPD2                      70.00 usec
PLW2                  7.80000019 W
PLW12                 0.09194400 W

F2 - Processing parameters
SI                         65536
SF                   150.8122865 MHz
WDW                           EM
SSB                            0
LB                          1.00 Hz
GB                             0
PC                          1.40
```
