# Supplementary material for: Discovery and characterization of a nickel enzyme family that catalyses intermolecular hydride shuttling
Source: Nat Chem. 2026 Jun 17;18(9):1582–92. doi: 10.1038/s41557-026-02184-9 (PMC13524815; doi:10.1038/s41557-026-02184-9)
Supplement: Supplementary file 2 [file 41557_2026_2184_MOESM10_ESM.zip › SupplementaryFigure42_NMR_butanal/V2601211905_MN4_2/42/pdata/1/email_V2601211905_MN4_42_1.pdf]

H1_standard.UW D2O /home/zsemersky/av600 zsemersky 11

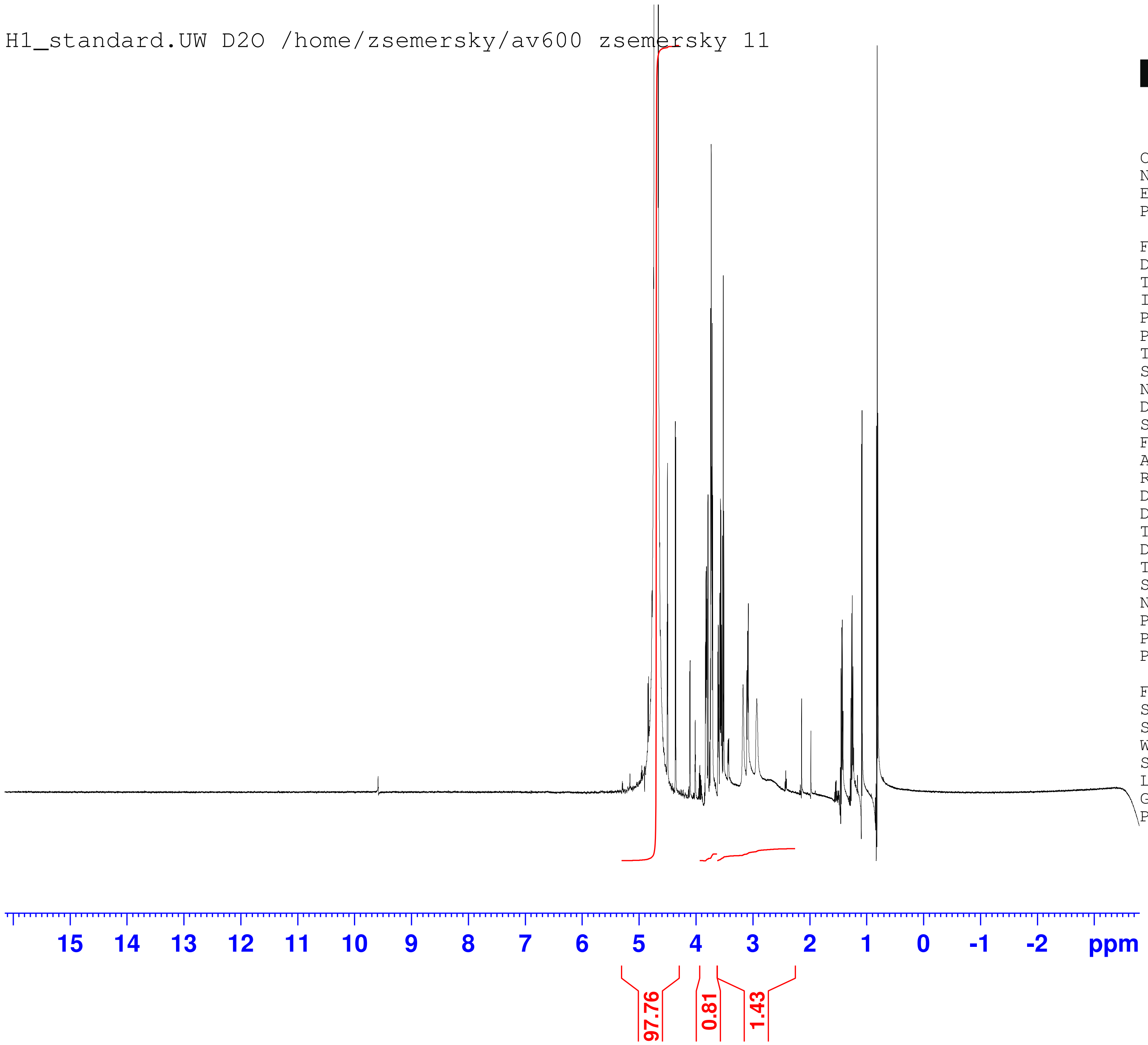

Current Data Parameters
NAME V2601211905_MN4
EXPNO 42
PROCNO 1

F2 - Acquisition Parameters
Date_ 20260121
Time 20.42 h
INSTRUM spect
PROBHD Z150313_0001 (
PULPROG zg30
TD 65536
SOLVENT D2O
NS 8
DS 2
SWH 11961.723 Hz
FIDRES 0.365043 Hz
AQ 2.7394049 sec
RG 11.25
DW 41.800 usec
DE 20.00 usec
TE 298.2 K
D1 1.00000000 sec
TD0 1
SFO1 599.7737038 MHz
NUC1 1H
P0 2.36 usec
P1 7.07 usec
PLW1 7.80000019 W

F2 - Processing parameters
SI 65536
SF 599.7700000 MHz
WDW EM
SSB 0
LB 0.30 Hz
GB 0
PC 1.00
