# Supplementary material for: Discovery and characterization of a nickel enzyme family that catalyses intermolecular hydride shuttling
Source: Nat Chem. 2026 Jun 17;18(9):1582–92. doi: 10.1038/s41557-026-02184-9 (PMC13524815; doi:10.1038/s41557-026-02184-9)
Supplement: Supplementary file 2 [file 41557_2026_2184_MOESM10_ESM.zip › SupplementaryFigure42_NMR_butanal/V2601211905_MN7_2/73/pdata/1/email_V2601211905_MN7_73_1.pdf]

C13_standard.UW D2O /home/zsemersky/av600 zsemersky 14

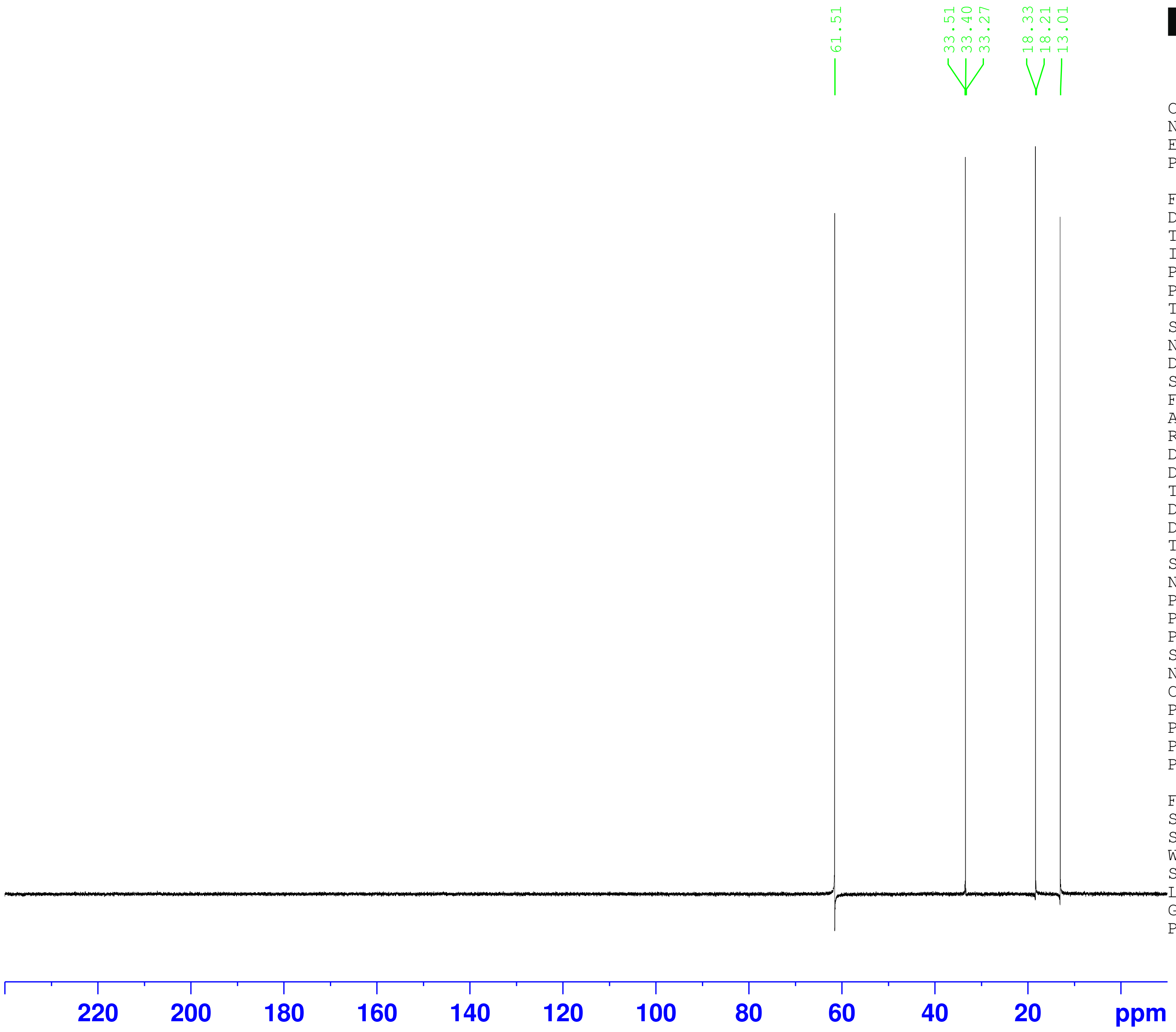

Current Data Parameters

NAME	V2601211905_MN7
EXPNO	73
PROCNO	1

F2 - Acquisition Parameters

Date_	20260121
Time	22.38 h
INSTRUM	spect
PROBHD	Z150313_0001 (
PULPROG	zgpg30
TD	71424
SOLVENT	D2O
NS	512
DS	2
SWH	42613.637 Hz
FIDRES	1.193258 Hz
AQ	0.8380416 sec
RG	190.75
DW	11.733 usec
DE	20.00 usec
TE	298.2 K
D1	2.00000000 sec
D11	0.03000000 sec
TD0	1
SFO1	150.8288758 MHz
NUC1	13C
P0	3.87 usec
P1	11.60 usec
PLW1	90.00000000 W
SFO2	599.7726990 MHz
NUC2	1H
CPDPRG[2	bi_waltz65_128
PCPD2	70.00 usec
PLW2	7.80000019 W
PLW12	0.09194400 W
PLW13	0.04624700 W

F2 - Processing parameters

SI	65536
SF	150.8122865 MHz
WDW	EM
SSB	0
LB	1.00 Hz
GB	0
PC	1.40
