# Supplementary material for: Discovery and characterization of a nickel enzyme family that catalyses intermolecular hydride shuttling
Source: Nat Chem. 2026 Jun 17;18(9):1582–92. doi: 10.1038/s41557-026-02184-9 (PMC13524815; doi:10.1038/s41557-026-02184-9)
Supplement: Supplementary file 2 [file 41557_2026_2184_MOESM10_ESM.zip › SupplementaryFigure42_NMR_butanal/V2601211905_MN2_2/23/pdata/1/email_V2601211905_MN2_23_1.pdf]

C13_standard.UW D2O /home/zsemersky/av600 zsemersky 9

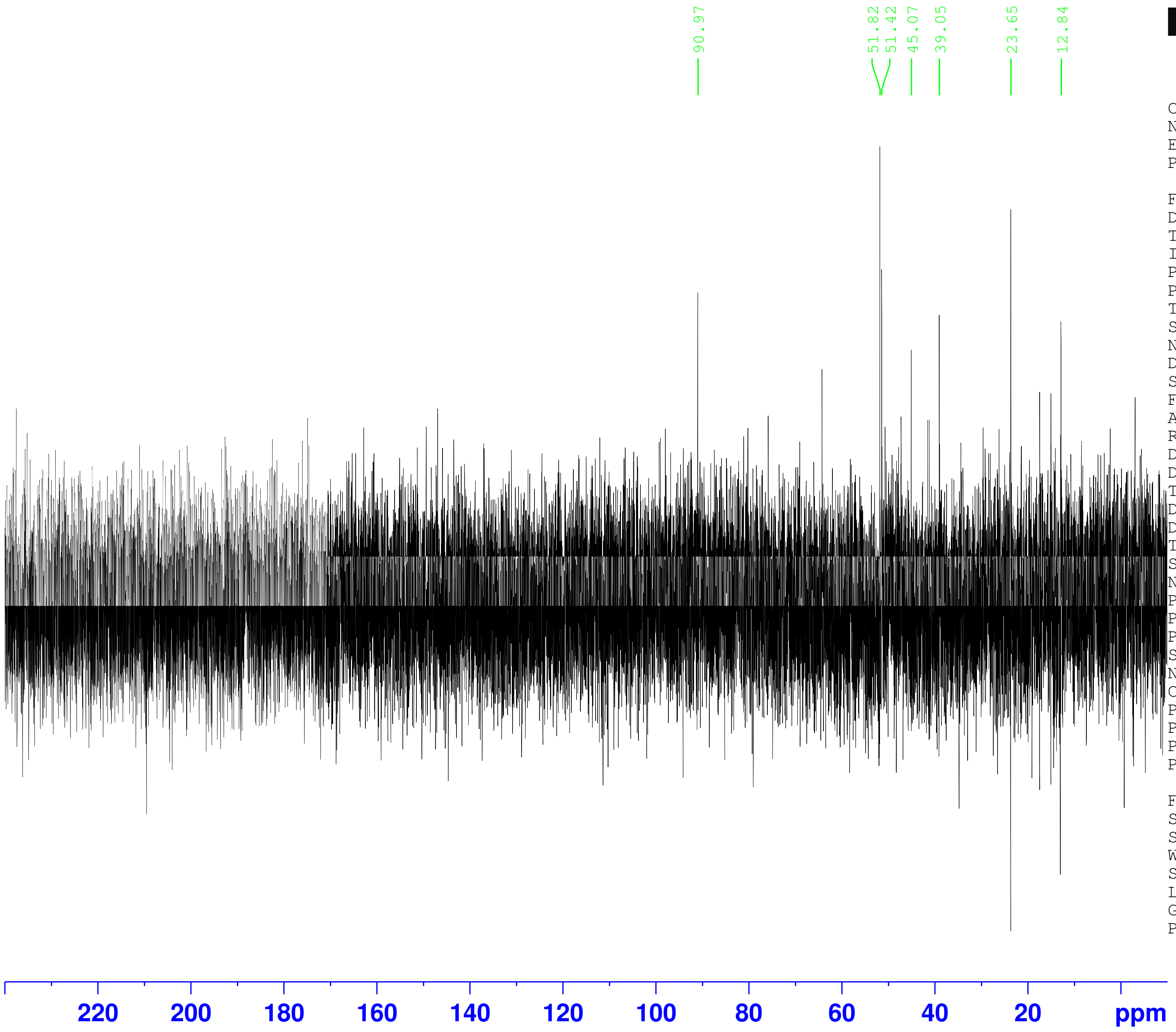

Current Data Parameters

NAME	V2601211905_MN2	
EXPNO	23	
PROCNO	1	

F2 - Acquisition Parameters

Date_	20260121	
Time	20.09	h
INSTRUM	spect	
PROBHD	Z150313_0001 (	
PULPROG	zgpg30	
TD	71424	
SOLVENT	D2O	
NS	512	
DS	2	
SWH	42613.637	Hz
FIDRES	1.193258	Hz
AQ	0.8380416	sec
RG	190.75	
DW	11.733	usec
DE	20.00	usec
TE	298.2	K
D1	2.00000000	sec
D11	0.03000000	sec
TD0	1	
SFO1	150.8288758	MHz
NUC1	13C	
P0	3.87	usec
P1	11.60	usec
PLW1	90.00000000	W
SFO2	599.7726990	MHz
NUC2	1H	
CPDPRG[2	bi_waltz65_128	
PCPD2	70.00	usec
PLW2	7.80000019	W
PLW12	0.09194400	W
PLW13	0.04624700	W

F2 - Processing parameters

SI	65536	
SF	150.8122865	MHz
WDW	EM	
SSB	0	
LB	1.00	Hz
GB	0	
PC	1.40	
