# Supplementary material for: Discovery and characterization of a nickel enzyme family that catalyses intermolecular hydride shuttling
Source: Nat Chem. 2026 Jun 17;18(9):1582–92. doi: 10.1038/s41557-026-02184-9 (PMC13524815; doi:10.1038/s41557-026-02184-9)
Supplement: Supplementary file 2 [file 41557_2026_2184_MOESM10_ESM.zip › SupplementaryFigure42_NMR_butanal/V2601211905_MN2_2/22/pdata/1/email_V2601211905_MN2_22_1.pdf]

H1_standard.UW D2O /home/zsemersky/av600 zsemersky 9

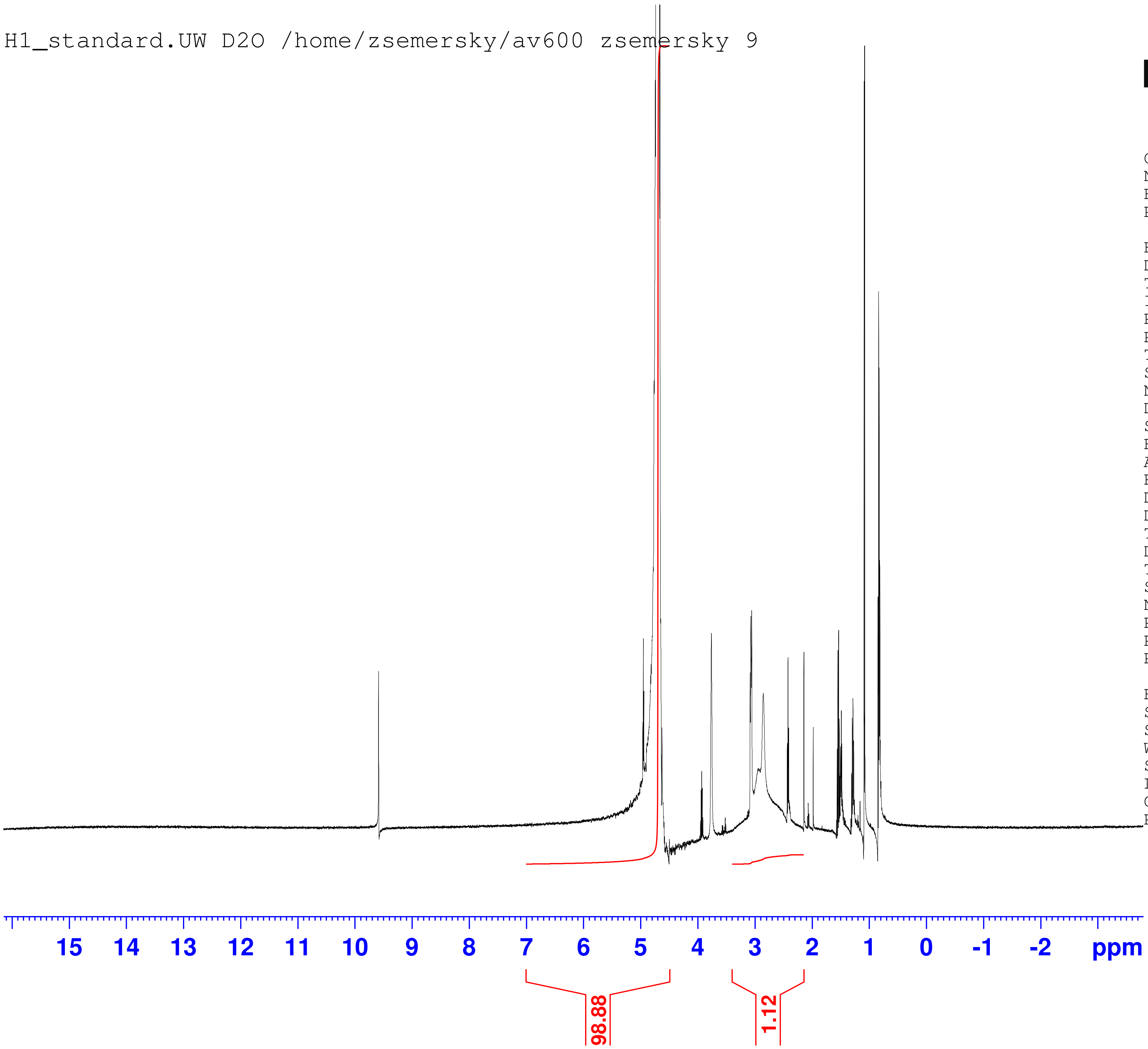

Current Data Parameters

NAME	V2601211905_MN2	
EXPNO	22	
PROCNO	1	

F2 - Acquisition Parameters

Date_	20260121	
Time	19.43	h
INSTRUM	spect	
PROBHD	Z150313_0001 (	
PULPROG	zg30	
TD	65536	
SOLVENT	D2O	
NS	8	
DS	2	
SWH	11961.723	Hz
FIDRES	0.365043	Hz
AQ	2.7394049	sec
RG	12.62	
DW	41.800	usec
DE	20.00	usec
TE	298.2	K
D1	1.00000000	sec
TD0	1	
SFO1	599.7737038	MHz
NUC1	1H	
P0	2.36	usec
P1	7.07	usec
PLW1	7.80000019	W

F2 - Processing parameters

SI	65536	
SF	599.7700000	MHz
WDW	EM	
SSB	0	
LB	0.30	Hz
GB	0	
PC	1.00	
