# Supplementary material for: Discovery and characterization of a nickel enzyme family that catalyses intermolecular hydride shuttling
Source: Nat Chem. 2026 Jun 17;18(9):1582–92. doi: 10.1038/s41557-026-02184-9 (PMC13524815; doi:10.1038/s41557-026-02184-9)
Supplement: Supplementary file 2 [file 41557_2026_2184_MOESM10_ESM.zip › SupplementaryFigure42_NMR_butanal/Run2/C2605051036_MN19/11/pdata/1/email_C2605051036_MN19_11_1.pdf]

C13_H1dec.UW D2O /home/zsemersky/callisto zsemersky 43

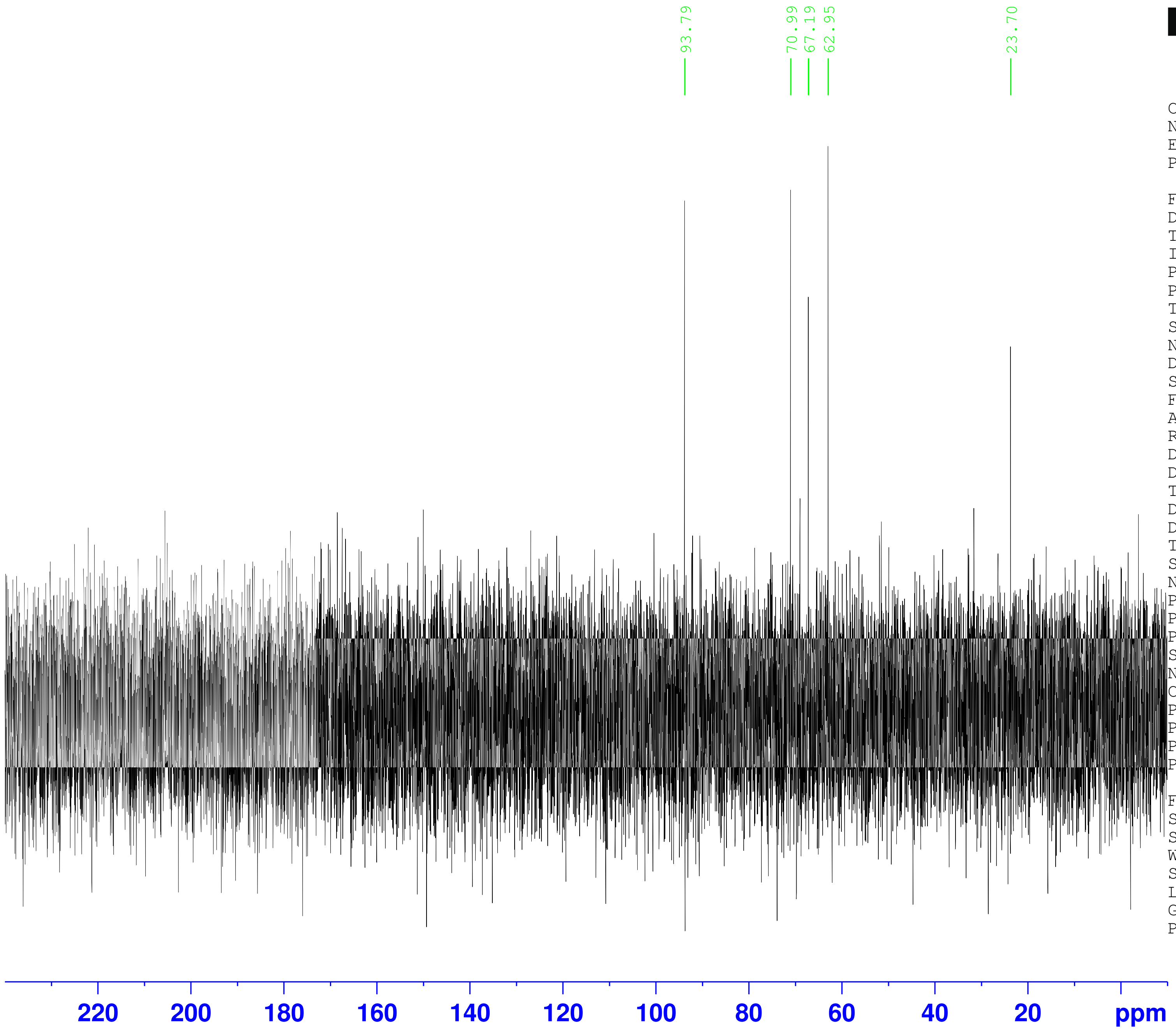

Current Data Parameters
NAME C2605051036_MN19
EXPNO 11
PROCNO 1

F2 - Acquisition Parameters
Date_ 20260505
Time 11.17 h
INSTRUM spect
PROBHD Z119248_0003 (
PULPROG zgpg30
TD 71424
SOLVENT D2O
NS 32
DS 2
SWH 35714.285 Hz
FIDRES 1.000064 Hz
AQ 0.9999360 sec
RG 203.75
DW 14.000 usec
DE 22.00 usec
TE 298.1 K
D1 2.00000000 sec
D11 0.03000000 sec
TD0 1
SFO1 125.7439593 MHz
NUC1 13C
P0 3.50 usec
P1 10.50 usec
PLW1 24.79999924 W
SFO2 500.0222501 MHz
NUC2 1H
CPDPRG[2 waltz16
PCPD2 80.00 usec
PLW2 15.00000000 W
PLW12 0.36329001 W
PLW13 0.18273000 W

F2 - Processing parameters
SI 65536
SF 125.7301290 MHz
WDW EM
SSB 0
LB 1.00 Hz
GB 0
PC 1.40
