# Supplementary material for: Discovery and characterization of a nickel enzyme family that catalyses intermolecular hydride shuttling
Source: Nat Chem. 2026 Jun 17;18(9):1582–92. doi: 10.1038/s41557-026-02184-9 (PMC13524815; doi:10.1038/s41557-026-02184-9)
Supplement: Supplementary file 2 [file 41557_2026_2184_MOESM10_ESM.zip › SupplementaryFigure42_NMR_butanal/Run2/C2605051036_MN19/10/pdata/1/email_C2605051036_MN19_10_1.pdf]

H1_standard.UW D2O /home/zsemersky/callisto zsemersky 43

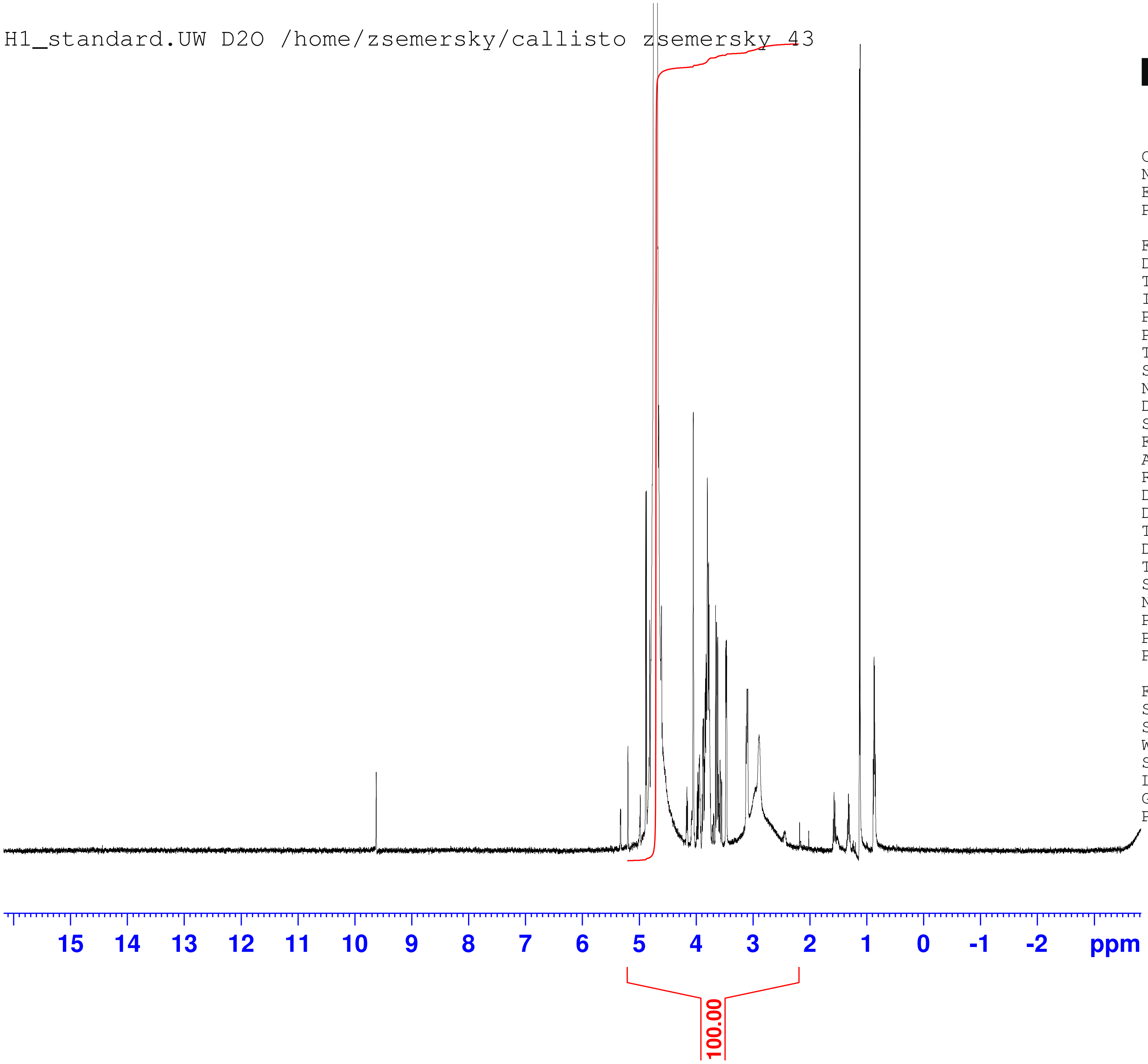


BRUKER

Current Data Parameters

| | |
|---|---|
| NAME | C2605051036_MN19 |
| EXPNO | 10 |
| PROCNO | 1 |

F2 - Acquisition Parameters

| | |
|---|---|
| Date_ | 20260505 |
| Time | 11.14 h |
| INSTRUM | spect |
| PROBHD | Z119248_0003 ( |
| PULPROG | zg30 |
| TD | 65536 |
| SOLVENT | D2O |
| NS | 8 |
| DS | 2 |
| SWH | 10000.000 Hz |
| FIDRES | 0.305176 Hz |
| AQ | 3.2767999 sec |
| RG | 30.38 |
| DW | 50.000 usec |
| DE | 18.00 usec |
| TE | 298.1 K |
| D1 | 1.00000000 sec |
| TD0 | 1 |
| SFO1 | 500.0230878 MHz |
| NUC1 | 1H |
| P0 | 4.45 usec |
| P1 | 13.35 usec |
| PLW1 | 15.00000000 W |

F2 - Processing parameters

| | |
|---|---|
| SI | 65536 |
| SF | 500.0200000 MHz |
| WDW | EM |
| SSB | 0 |
| LB | 0.30 Hz |
| GB | 0 |
| PC | 1.00 |
