# Supplementary material for: Discovery and characterization of a nickel enzyme family that catalyses intermolecular hydride shuttling
Source: Nat Chem. 2026 Jun 17;18(9):1582–92. doi: 10.1038/s41557-026-02184-9 (PMC13524815; doi:10.1038/s41557-026-02184-9)
Supplement: Supplementary file 3 [file 41557_2026_2184_MOESM11_ESM.zip › SupplementaryFigure43_NMR_inositol/C2601291118_D2O_GB/10/pdata/1/email_C2601291118_D2O_GB_10_1.pdf]

H1_standard.UW D2O /home/zsemersky/callisto zsemersky 34

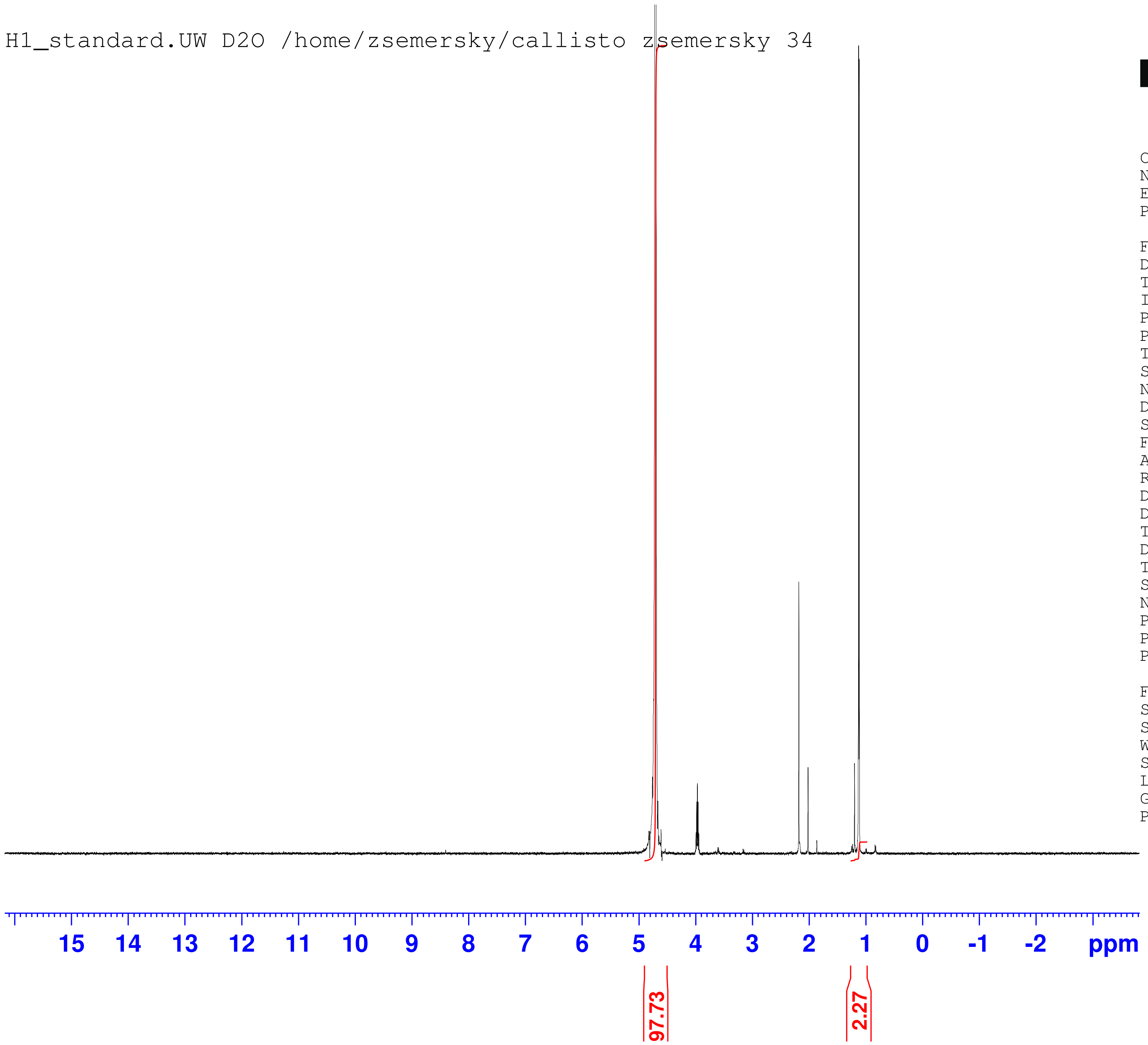

Current Data Parameters

NAME	C2601291118_D2O_GB	
EXPNO	10	
PROCNO	1	

F2 - Acquisition Parameters

Date_	20260129	
Time	12.07	h
INSTRUM	spect	
PROBHD	Z119248_0003 (	
PULPROG	zg30	
TD	65536	
SOLVENT	D2O	
NS	8	
DS	2	
SWH	10000.000	Hz
FIDRES	0.305176	Hz
AQ	3.2767999	sec
RG	50.79	
DW	50.000	usec
DE	18.00	usec
TE	298.2	K
D1	1.00000000	sec
TD0	1	
SFO1	500.0230878	MHz
NUC1	1H	
P0	4.45	usec
P1	13.35	usec
PLW1	15.00000000	W

F2 - Processing parameters

SI	65536	
SF	500.0200000	MHz
WDW	EM	
SSB	0	
LB	0.30	Hz
GB	0	
PC	1.00	
