# Supplementary material for: Discovery and characterization of a nickel enzyme family that catalyses intermolecular hydride shuttling
Source: Nat Chem. 2026 Jun 17;18(9):1582–92. doi: 10.1038/s41557-026-02184-9 (PMC13524815; doi:10.1038/s41557-026-02184-9)
Supplement: Supplementary file 4 [file 41557_2026_2184_MOESM3_ESM.zip › Combined_files/20251222_L-rham_20uM/13/pdata/1/email_20251222_L-rham_20uM_13_1.pdf]

C13_H1dec.UW D2O /home/zsemersky/callisto zsemersky 2

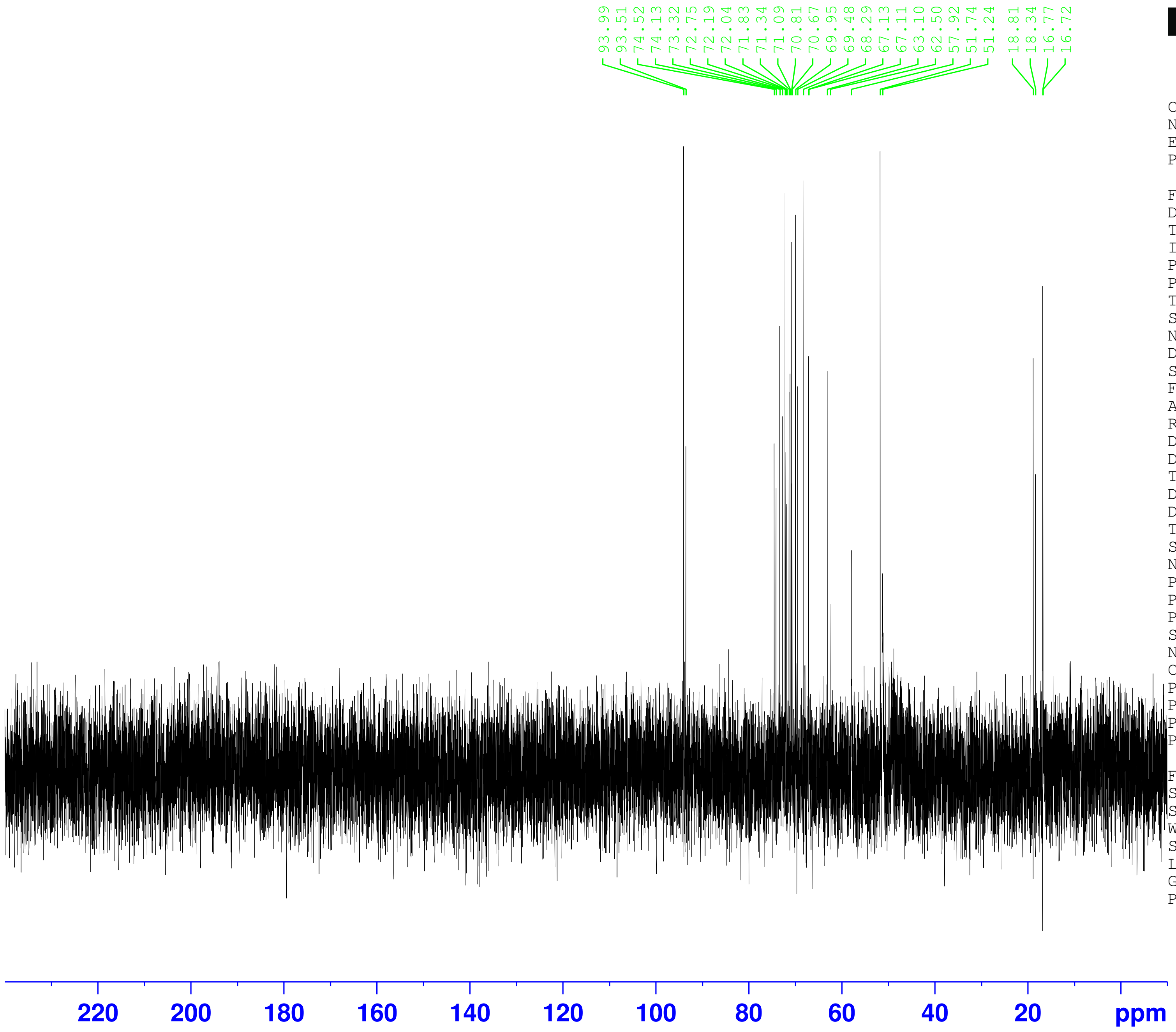

BRUKER

Current Data Parameters

NAME	20251222_L-rham_20uM
EXPNO	13
PROCNO	1

F2 - Acquisition Parameters

Date_	20251222
Time	11.26 h
INSTRUM	spect
PROBHD	Z119248_0003 (
PULPROG	zgpg30
TD	71424
SOLVENT	D2O
NS	128
DS	2
SWH	35714.285 Hz
FIDRES	1.000064 Hz
AQ	0.9999360 sec
RG	203.75
DW	14.000 usec
DE	22.00 usec
TE	298.2 K
D1	2.00000000 sec
D11	0.03000000 sec
TD0	1
SFO1	125.7439593 MHz
NUC1	13C
P0	3.50 usec
P1	10.50 usec
PLW1	24.79999924 W
SFO2	500.0222501 MHz
NUC2	1H
CPDPRG[2	waltz16
PCPD2	80.00 usec
PLW2	15.00000000 W
PLW12	0.36329001 W
PLW13	0.18273000 W

F2 - Processing parameters

SI	65536
SF	125.7301290 MHz
WDW	EM
SSB	0
LB	1.00 Hz
GB	0
PC	1.40
