# Supplementary material for: Discovery and characterization of a nickel enzyme family that catalyses intermolecular hydride shuttling
Source: Nat Chem. 2026 Jun 17;18(9):1582–92. doi: 10.1038/s41557-026-02184-9 (PMC13524815; doi:10.1038/s41557-026-02184-9)
Supplement: Supplementary file 4 [file 41557_2026_2184_MOESM3_ESM.zip › Combined_files/20251222_D-glu_20uM/12/pdata/1/email_20251222_D-glu_20uM_12_1.pdf]

H1_standard.UW D2O /home/zsemersky/callisto zsemersky 6

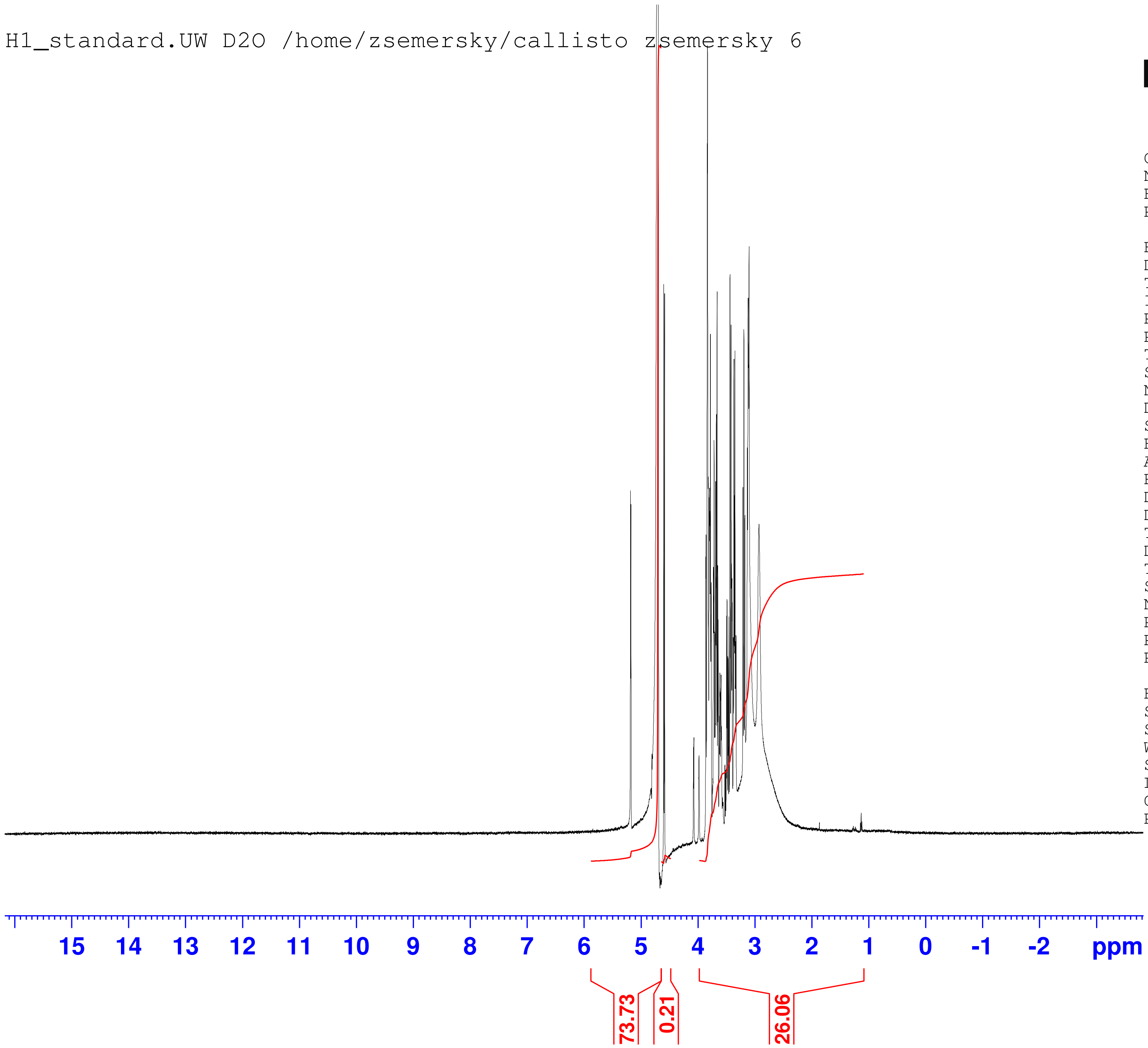

BRUKER

Current Data Parameters

NAME	20251222_D-glu_20uM
EXPNO	12
PROCNO	1

F2 - Acquisition Parameters

Date_	20251222
Time	14.34 h
INSTRUM	spect
PROBHD	Z119248_0003 (
PULPROG	zg30
TD	65536
SOLVENT	D2O
NS	8
DS	2
SWH	10000.000 Hz
FIDRES	0.305176 Hz
AQ	3.2767999 sec
RG	65.27
DW	50.000 usec
DE	18.00 usec
TE	298.1 K
D1	1.00000000 sec
TD0	1
SFO1	500.0230878 MHz
NUC1	1H
P0	4.45 usec
P1	13.35 usec
PLW1	15.00000000 W

F2 - Processing parameters

SI	65536
SF	500.0200000 MHz
WDW	EM
SSB	0
LB	0.30 Hz
GB	0
PC	1.00
