# Supplementary material for: Discovery and characterization of a nickel enzyme family that catalyses intermolecular hydride shuttling
Source: Nat Chem. 2026 Jun 17;18(9):1582–92. doi: 10.1038/s41557-026-02184-9 (PMC13524815; doi:10.1038/s41557-026-02184-9)
Supplement: Supplementary file 4 [file 41557_2026_2184_MOESM3_ESM.zip › Combined_files/D-gal_holo/15/pdata/1/email_D-gal_holo_15_1.pdf]

C13_H1dec.UW D2O /home/zsemersky/callisto zsemersky 4

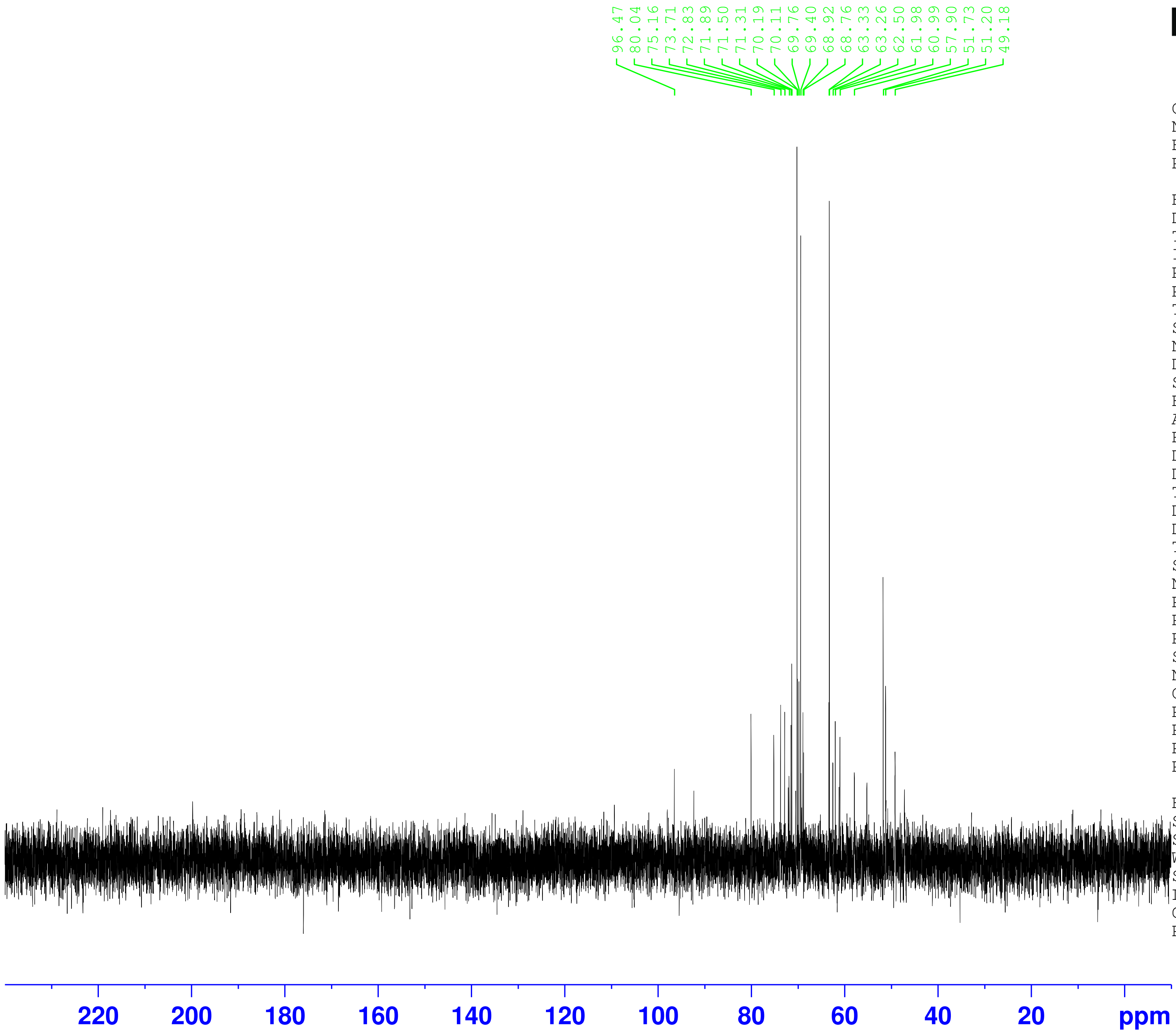

BRUKER

Current Data Parameters

NAME	D-gal_holo	
EXPNO	15	
PROCNO	1	

F2 - Acquisition Parameters

Date_	20251222	
Time	12.00	h
INSTRUM	spect	
PROBHD	Z119248_0003 (	
PULPROG	zgpg30	
TD	71424	
SOLVENT	D2O	
NS	128	
DS	2	
SWH	35714.285	Hz
FIDRES	1.000064	Hz
AQ	0.9999360	sec
RG	203.75	
DW	14.000	usec
DE	22.00	usec
TE	298.2	K
D1	2.00000000	sec
D11	0.03000000	sec
TD0	1	
SFO1	125.7439593	MHz
NUC1	13C	
P0	3.50	usec
P1	10.50	usec
PLW1	24.79999924	W
SFO2	500.0222501	MHz
NUC2	1H	
CPDPRG[2	waltz16	
PCPD2	80.00	usec
PLW2	15.00000000	W
PLW12	0.36329001	W
PLW13	0.18273000	W

F2 - Processing parameters

SI	65536	
SF	125.7301290	MHz
WDW	EM	
SSB	0	
LB	1.00	Hz
GB	0	
PC	1.40	
