# Supplementary material for: Discovery and characterization of a nickel enzyme family that catalyses intermolecular hydride shuttling
Source: Nat Chem. 2026 Jun 17;18(9):1582–92. doi: 10.1038/s41557-026-02184-9 (PMC13524815; doi:10.1038/s41557-026-02184-9)
Supplement: Supplementary file 6 [file 41557_2026_2184_MOESM5_ESM.zip › SupplementaryFigure14_NphT_L-arab_NMR/SuppFig14_L-arabinose_-NPMN/14/pdata/1/email_1b_phoebe_14_1.pdf]

HC_hmbc.UW D2O /home/zsemersky/av600 zsemersky 11

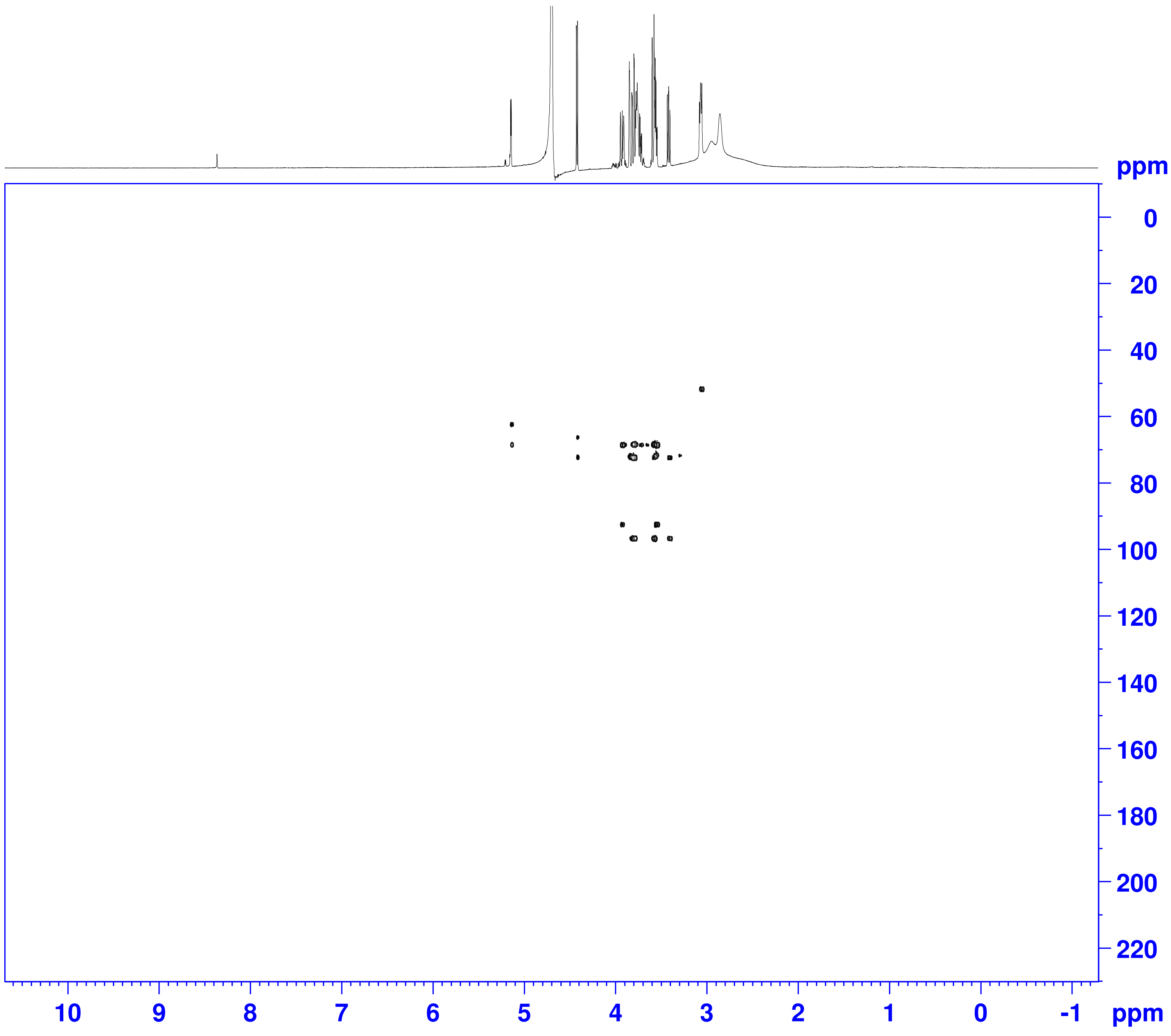


```
Current Data Parameters
NAME            1b_phoebe
EXPNO                  14
PROCNO                  1

F2 - Acquisition Parameters
Date_            20250819
Time                15.33 h
INSTRUM             spect
PROBHD     Z150313_0001 (
PULPROG      hmbcetgpl3nd
TD                   4096
SOLVENT               D2O
NS                      8
DS                     32
SWH              7183.908 Hz
FIDRES           3.507768 Hz
AQ              0.2850816 sec
RG                 190.75
DW                 69.600 usec
DE                  20.00 usec
TE                  298.1 K
CNST6         120.0000000
CNST7         170.0000000
CNST13          8.0000000
D0             0.00000300 sec
D1             2.00000000 sec
D6             0.06250000 sec
D16            0.00020000 sec
IN0            0.00001380 sec
TDav                    1
SFO1          599.7728189 MHz
NUC1                   1H
P1                   7.07 usec
P2                  14.14 usec
PLW1           7.80000019 W
SFO2          150.8288758 MHz
NUC2                  13C
P3                  12.20 usec
P24               2000.00 usec
PLW2          90.00000000 W
SPNAM[7]      Crp60comp.4
SPOAL7              0.500
SPOFFS7                 0 Hz
SPW7          20.46699905 W
GPNAM[1]       SMSQ10.100
GPZ1                80.00 %
GPNAM[3]       SMSQ10.100
GPZ3                14.00 %
GPNAM[4]       SMSQ10.100
GPZ4                -8.00 %
GPNAM[5]       SMSQ10.100
GPZ5                -4.00 %
GPNAM[6]       SMSQ10.100
GPZ6                -2.00 %
P16               1000.00 usec

F1 - Acquisition parameters
TD                    256
SFO1             150.8289 MHz
FIDRES         283.061584 Hz
SW                240.218 ppm
FnMODE       Echo-Antiecho

F2 - Processing parameters
SI                   4096
SF            599.7700000 MHz
WDW                  SINE
SSB                     4
LB                      0 Hz
GB                      0
PC                   1.40

F1 - Processing parameters
SI                   1024
MC2         echo-antiecho
SF            150.8122865 MHz
WDW                 QSINE
SSB                     2
LB                      0 Hz
GB                      0
```
