# Supplementary material for: Discovery and characterization of a nickel enzyme family that catalyses intermolecular hydride shuttling
Source: Nat Chem. 2026 Jun 17;18(9):1582–92. doi: 10.1038/s41557-026-02184-9 (PMC13524815; doi:10.1038/s41557-026-02184-9)
Supplement: Supplementary file 6 [file 41557_2026_2184_MOESM5_ESM.zip › SupplementaryFigure14_NphT_L-arab_NMR/SuppFig14_L-arabinose_-NPMN/12/pdata/1/email_1b_phoebe_12_1.pdf]

HH_cosy.UW D2O /home/zsemersky/av600 zsemersky 11

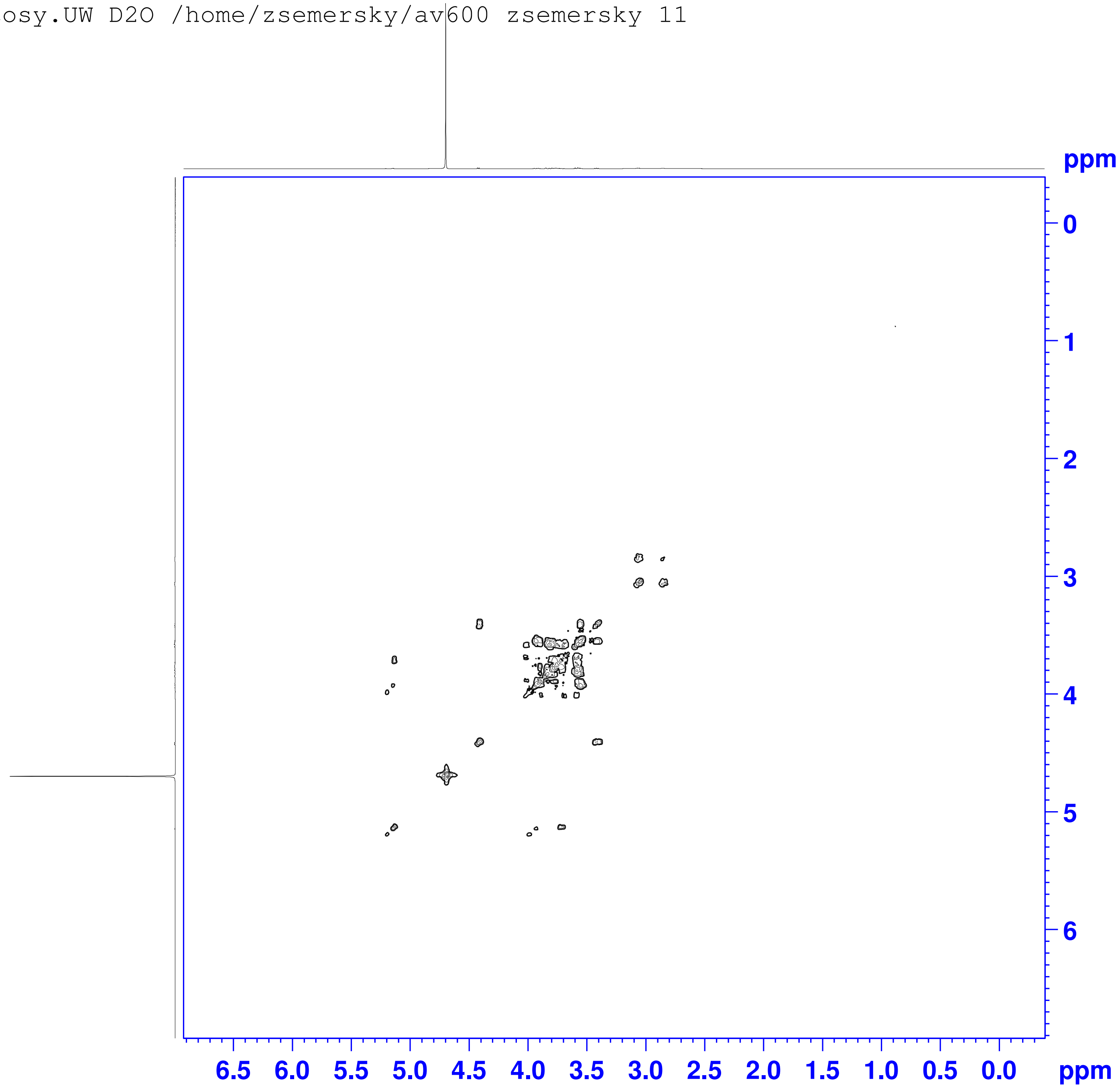

BRUKER

```
Current Data Parameters
NAME              1b_phoebe
EXPNO                    12
PROCNO                    1

F2 - Acquisition Parameters
Date_              20250819
Time                  13.30 h
INSTRUM               spect
PROBHD    Z150313_0001 (
PULPROG   cosygp45ppqf.UW
TD                     2048
SOLVENT                 D2O
NS                        4
DS                       32
SWH                4385.965 Hz
FIDRES             4.283169 Hz
AQ                0.2334720 sec
RG                    48.71
DW                  114.000 usec
DE                    20.00 usec
TE                    298.1 K
D0               0.00000300 sec
D1               1.90906894 sec
D11              0.03000000 sec
D12              0.00002000 sec
D13              0.00000400 sec
D16              0.00020000 sec
IN0              0.00022800 sec
TDav                      1
SFO1            599.7719597 MHz
NUC1                     1H
P0                     7.07 usec
P1                     7.07 usec
P17                 2500.00 usec
PLW1             7.80000019 W
PLW10            0.62380999 W
GPNAM[1]         SMSQ10.100
GPZ1                  10.00 %
P16                 1000.00 usec

F1 - Acquisition parameters
TD                      256
SFO1                599.772 MHz
FIDRES            34.265350 Hz
SW                    7.313 ppm
FnMODE                   QF

F2 - Processing parameters
SI                     1024
SF              599.7700000 MHz
WDW                   QSINE
SSB                       0
LB                        0 Hz
GB                        0
PC                     1.40

F1 - Processing parameters
SI                     1024
MC2                      QF
SF              599.7700000 MHz
WDW                   QSINE
SSB                       0
LB                        0 Hz
GB                        0
```
