# Supplementary material for: Discovery and characterization of a nickel enzyme family that catalyses intermolecular hydride shuttling
Source: Nat Chem. 2026 Jun 17;18(9):1582–92. doi: 10.1038/s41557-026-02184-9 (PMC13524815; doi:10.1038/s41557-026-02184-9)
Supplement: Supplementary file 6 [file 41557_2026_2184_MOESM5_ESM.zip › SupplementaryFigure14_NphT_L-arab_NMR/SuppFig14_L-arabinose_+NPMN/11/pdata/1/email_Phoebe_2588_1A_11_1.pdf]

C13_standard.UW D2O /home/zsemersky/av600 zsemersky 13

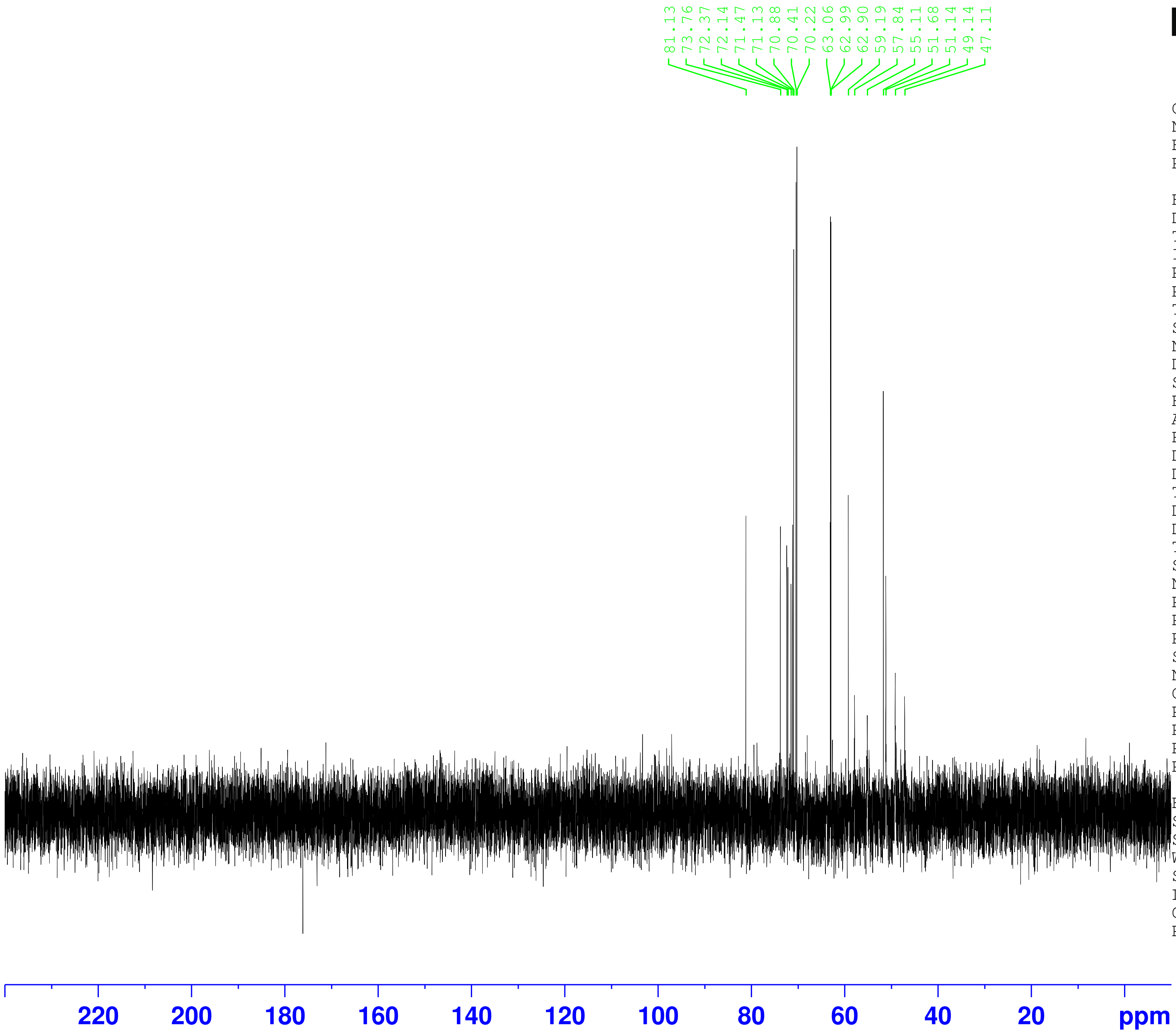

Current Data Parameters
NAME Phoebe_2588_1A
EXPNO 11
PROCNO 1

F2 - Acquisition Parameters
Date_ 20250814
Time 12.08 h
INSTRUM spect
PROBHD Z150313_0001 (
PULPROG zgpg30
TD 71424
SOLVENT D2O
NS 600
DS 2
SWH 42613.637 Hz
FIDRES 1.193258 Hz
AQ 0.8380416 sec
RG 190.75
DW 11.733 usec
DE 20.00 usec
TE 298.1 K
D1 2.00000000 sec
D11 0.03000000 sec
TD0 1
SFO1 150.8288758 MHz
NUC1 13C
P0 3.87 usec
P1 11.60 usec
PLW1 90.00000000 W
SFO2 599.7726990 MHz
NUC2 1H
CPDPRG[2 bi_waltz65_128
PCPD2 70.00 usec
PLW2 7.80000019 W
PLW12 0.09194400 W
PLW13 0.04624700 W

F2 - Processing parameters
SI 65536
SF 150.8122865 MHz
WDW EM
SSB 0
LB 1.00 Hz
GB 0
PC 1.40
