# Supplementary material for: Discovery and characterization of a nickel enzyme family that catalyses intermolecular hydride shuttling
Source: Nat Chem. 2026 Jun 17;18(9):1582–92. doi: 10.1038/s41557-026-02184-9 (PMC13524815; doi:10.1038/s41557-026-02184-9)
Supplement: Supplementary file 6 [file 41557_2026_2184_MOESM5_ESM.zip › SupplementaryFigure14_NphT_L-arab_NMR/SuppFig14_L-arabinose_+NPMN/12/pdata/1/email_Phoebe_2588_1A_12_1.pdf]

HH_cosy.UW D2O /home/zsemersky/av600 zsemersky 13

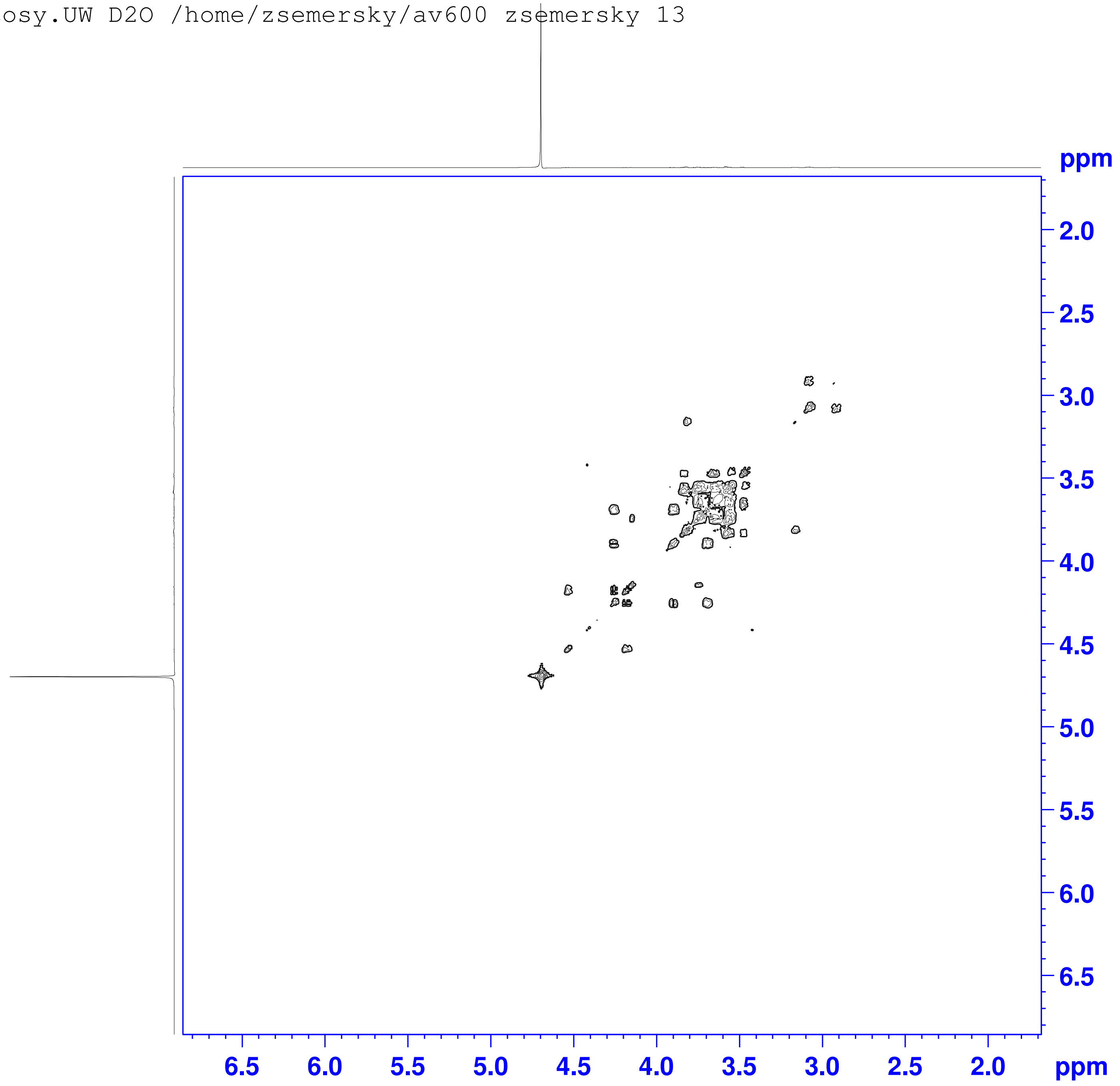

Current Data Parameters

NAME	Phoebe_2588_1A
EXPNO	12
PROCNO	1

F2 - Acquisition Parameters

Date_	20250814
Time	12.48 h
INSTRUM	spect
PROBHD	Z150313_0001 (
PULPROG	cosygp45ppqf.UW
TD	2048
SOLVENT	D2O
NS	4
DS	32
SWH	3105.590 Hz
FIDRES	3.032803 Hz
AQ	0.3297280 sec
RG	34.52
DW	161.000 usec
DE	20.00 usec
TE	298.1 K
D0	0.00000300 sec
D1	1.81281304 sec
D11	0.03000000 sec
D12	0.00002000 sec
D13	0.00000400 sec
D16	0.00020000 sec
IN0	0.00032200 sec
TDav	1
SFO1	599.7725601 MHz
NUC1	1H
P0	7.07 usec
P1	7.07 usec
P17	2500.00 usec
PLW1	7.80000019 W
PLW10	0.62380999 W
GPNAM[1]	SMSQ10.100
GPZ1	10.00 %
P16	1000.00 usec

F1 - Acquisition parameters

TD	256
SFO1	599.7726 MHz
FIDRES	24.262423 Hz
SW	5.178 ppm
FnMODE	QF

F2 - Processing parameters

SI	1024
SF	599.7700000 MHz
WDW	QSINE
SSB	0
LB	0 Hz
GB	0
PC	1.40

F1 - Processing parameters

SI	1024
MC2	QF
SF	599.7700000 MHz
WDW	QSINE
SSB	0
LB	0 Hz
GB	0
