# Supplementary material for: Discovery and characterization of a nickel enzyme family that catalyses intermolecular hydride shuttling
Source: Nat Chem. 2026 Jun 17;18(9):1582–92. doi: 10.1038/s41557-026-02184-9 (PMC13524815; doi:10.1038/s41557-026-02184-9)
Supplement: Supplementary file 6 [file 41557_2026_2184_MOESM5_ESM.zip › SupplementaryFigure14_NphT_L-arab_NMR/SuppFig14_L-arabinose_+NPMN/10/pdata/1/email_Phoebe_2588_1A_10_1.pdf]

H1_standard.UW D2O /home/zsemersky/av600 zsemersky 13

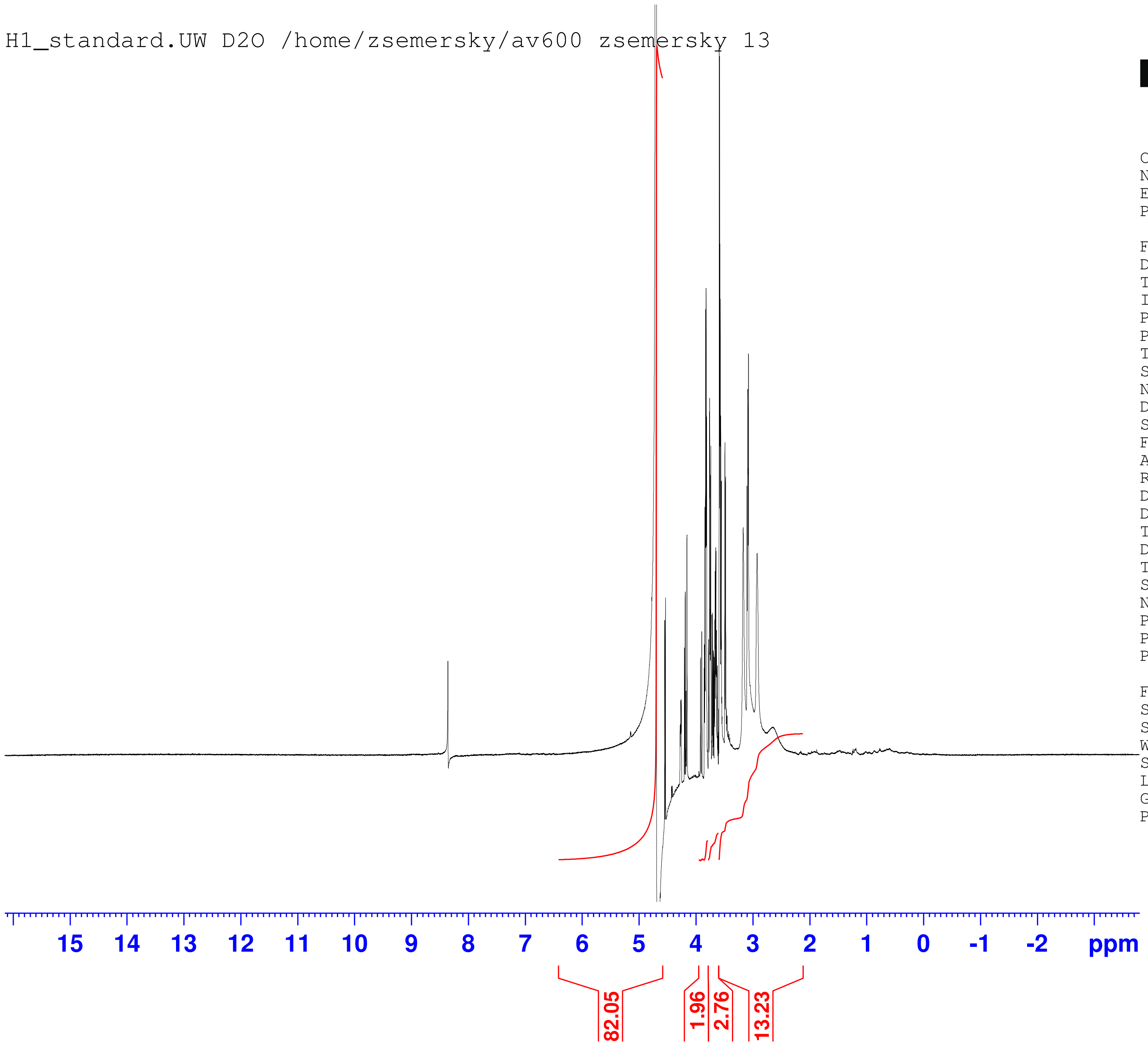

BRUKER

Current Data Parameters

NAME	Phoebe_2588_1A
EXPNO	10
PROCNO	1

F2 - Acquisition Parameters

Date_	20250814
Time	11.38 h
INSTRUM	spect
PROBHD	Z150313_0001 (
PULPROG	zg30
TD	65536
SOLVENT	D2O
NS	16
DS	2
SWH	11961.723 Hz
FIDRES	0.365043 Hz
AQ	2.7394049 sec
RG	22.33
DW	41.800 usec
DE	20.00 usec
TE	298.1 K
D1	1.00000000 sec
TD0	1
SFO1	599.7737038 MHz
NUC1	1H
P0	2.36 usec
P1	7.07 usec
PLW1	7.80000019 W

F2 - Processing parameters

SI	65536
SF	599.7700000 MHz
WDW	EM
SSB	0
LB	0.30 Hz
GB	0
PC	1.00
