# Supplementary material for: Discovery and characterization of a nickel enzyme family that catalyses intermolecular hydride shuttling
Source: Nat Chem. 2026 Jun 17;18(9):1582–92. doi: 10.1038/s41557-026-02184-9 (PMC13524815; doi:10.1038/s41557-026-02184-9)
Supplement: Supplementary file 7 [file 41557_2026_2184_MOESM6_ESM.zip › SupplementaryFigure18_NphT_D-gal_NMR/SuppFig18_D-galactose_+NPMN/27/pdata/1/email_Phoebe_2588_2A_27_1.pdf]

HH_cosy.UW D2O /home/zsemersky/av600 zsemersky 14

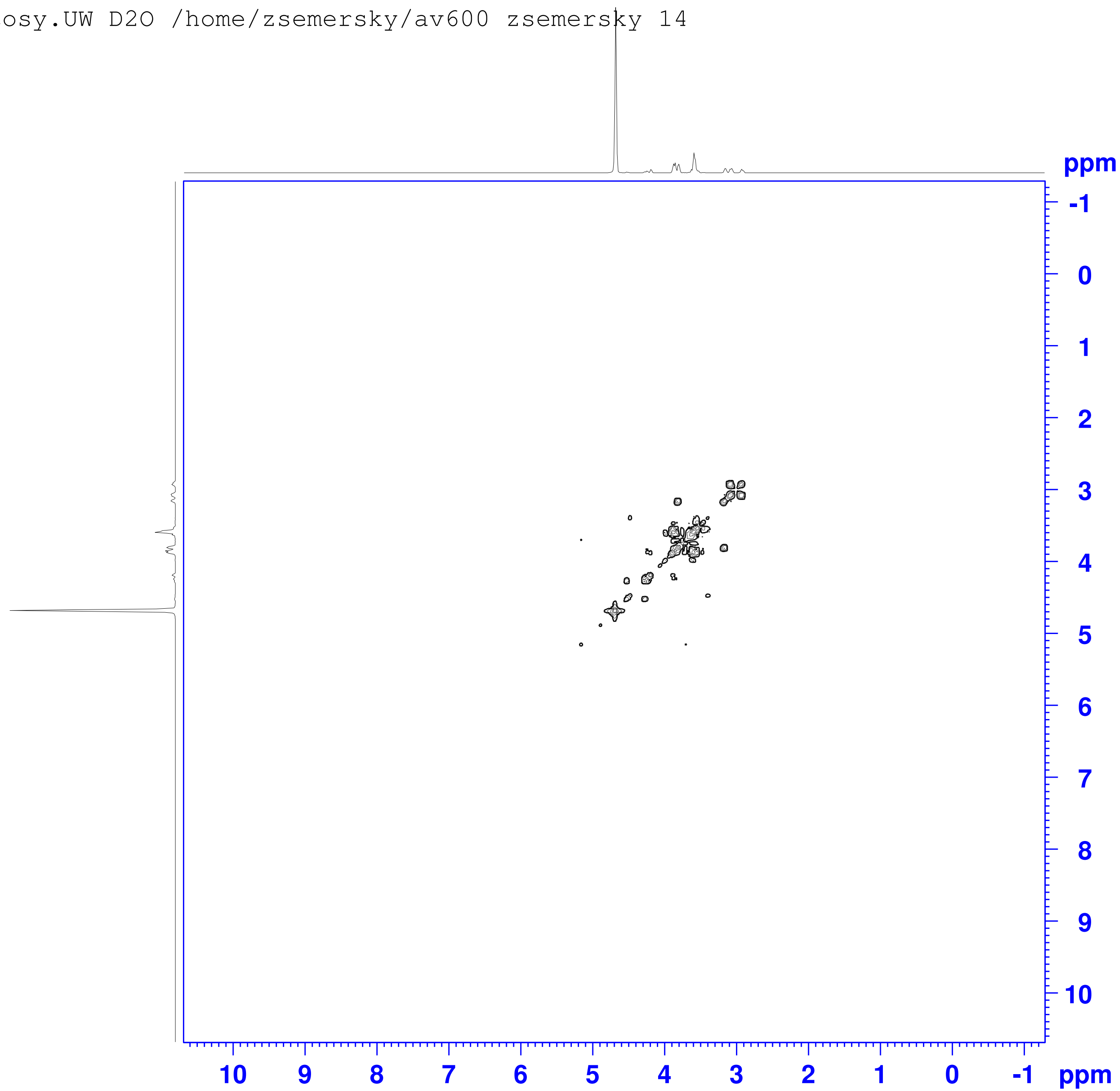


```
Current Data Parameters
NAME      Phoebe_2588_2A
EXPNO                 27
PROCNO                 1

F2 - Acquisition Parameters
Date_           20250814
Time               16.05 h
INSTRUM            spect
PROBHD    Z150313_0001 (
PULPROG  cosygp45ppqf.UW
TD                  2048
SOLVENT              D2O
NS                     4
DS                    32
SWH             7183.908 Hz
FIDRES          7.015535 Hz
AQ             0.1425408 sec
RG                  38.8
DW                69.600 usec
DE                 20.00 usec
TE                 298.1 K
D0            0.00000300 sec
D1            2.00000000 sec
D11           0.03000000 sec
D12           0.00002000 sec
D13           0.00000400 sec
D16           0.00020000 sec
IN0           0.00013920 sec
TDav                   1
SFO1         599.7728189 MHz
NUC1                  1H
P0                  7.07 usec
P1                  7.07 usec
P17              2500.00 usec
PLW1          7.80000019 W
PLW10         0.62380999 W
GPNAM[1]      SMSQ10.100
GPZ1               10.00 %
P16              1000.00 usec

F1 - Acquisition parameters
TD                   256
SFO1            599.7728 MHz
FIDRES         56.124283 Hz
SW                11.978 ppm
FnMODE                QF

F2 - Processing parameters
SI                  1024
SF           599.7700000 MHz
WDW                QSINE
SSB                    0
LB                     0 Hz
GB                     0
PC                  1.40

F1 - Processing parameters
SI                  1024
MC2                   QF
SF           599.7700000 MHz
WDW                QSINE
SSB                    0
LB                     0 Hz
GB                     0
```
