# Supplementary material for: Discovery and characterization of a nickel enzyme family that catalyses intermolecular hydride shuttling
Source: Nat Chem. 2026 Jun 17;18(9):1582–92. doi: 10.1038/s41557-026-02184-9 (PMC13524815; doi:10.1038/s41557-026-02184-9)
Supplement: Supplementary file 7 [file 41557_2026_2184_MOESM6_ESM.zip › SupplementaryFigure18_NphT_D-gal_NMR/SuppFig18_D-galactose_+NPMN/29/pdata/1/email_Phoebe_2588_2A_29_1.pdf]

HC_hmbc.UW D2O /home/zsemersky/av600 zsemersky 14

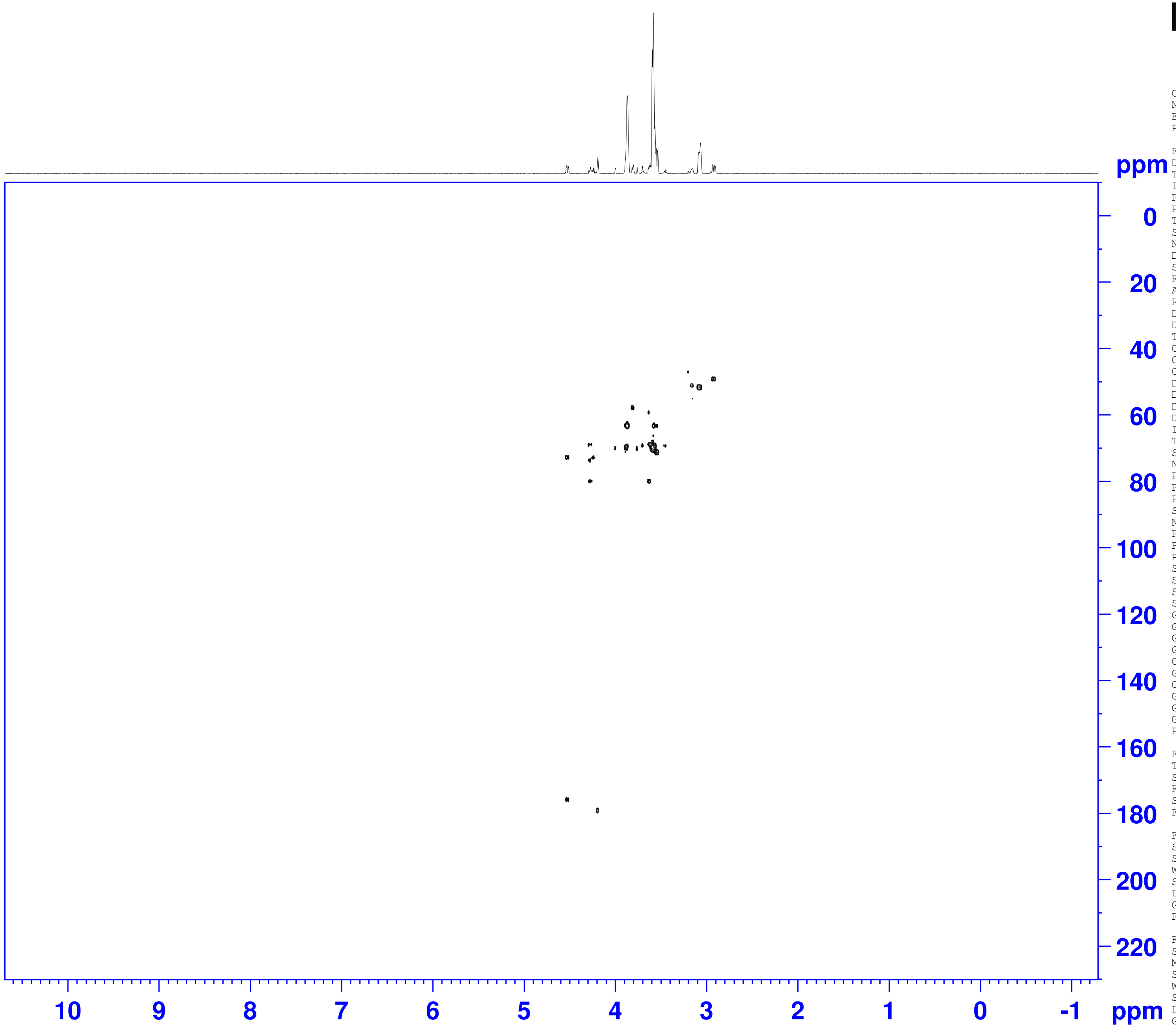

Current Data Parameters
NAME Phoebe_2588_2A
EXPNO 29
PROCNO 1

F2 - Acquisition Parameters
Date_ 20250814
Time 18.07 h
INSTRUM spect
PROBHD Z150313_0001 (
PULPROG hmbcetgpl3nd
TD 4096
SOLVENT D2O
NS 8
DS 32
SWH 7183.908 Hz
FIDRES 3.507768 Hz
AQ 0.2850816 sec
RG 190.75
DW 69.600 usec
DE 20.00 usec
TE 298.1 K
CNST6 120.0000000
CNST7 170.0000000
CNST13 8.0000000
D0 0.00000300 sec
D1 2.00000000 sec
D6 0.06250000 sec
D16 0.00020000 sec
IN0 0.00001380 sec
TDav 1
SFO1 599.7728189 MHz
NUC1 1H
P1 7.07 usec
P2 14.14 usec
PLW1 7.80000019 W
SFO2 150.8288758 MHz
NUC2 13C
P3 12.20 usec
P24 2000.00 usec
PLW2 90.00000000 W
SPNAM[7] Crp60comp.4
SPOAL7 0.500
SPOFFS7 0 Hz
SPW7 20.46699905 W
GPNAM[1] SMSQ10.100
GPZ1 80.00 %
GPNAM[3] SMSQ10.100
GPZ3 14.00 %
GPNAM[4] SMSQ10.100
GPZ4 -8.00 %
GPNAM[5] SMSQ10.100
GPZ5 -4.00 %
GPNAM[6] SMSQ10.100
GPZ6 -2.00 %
P16 1000.00 usec

F1 - Acquisition parameters
TD 256
SFO1 150.8289 MHz
FIDRES 283.061584 Hz
SW 240.218 ppm
FnMODE Echo-Antiecho

F2 - Processing parameters
SI 4096
SF 599.7700000 MHz
WDW SINE
SSB 4
LB 0 Hz
GB 0
PC 1.40

F1 - Processing parameters
SI 1024
MC2 echo-antiecho
SF 150.8122865 MHz
WDW QSINE
SSB 2
LB 0 Hz
GB 0
