# Supplementary material for: Discovery and characterization of a nickel enzyme family that catalyses intermolecular hydride shuttling
Source: Nat Chem. 2026 Jun 17;18(9):1582–92. doi: 10.1038/s41557-026-02184-9 (PMC13524815; doi:10.1038/s41557-026-02184-9)
Supplement: Supplementary file 7 [file 41557_2026_2184_MOESM6_ESM.zip › SupplementaryFigure18_NphT_D-gal_NMR/SuppFig18_D-galactose_-NPMN/24/pdata/1/email_2b_phoebe_24_1.pdf]

HC_hmbc.UW D2O /home/zsemersky/av600 zsemersky 12

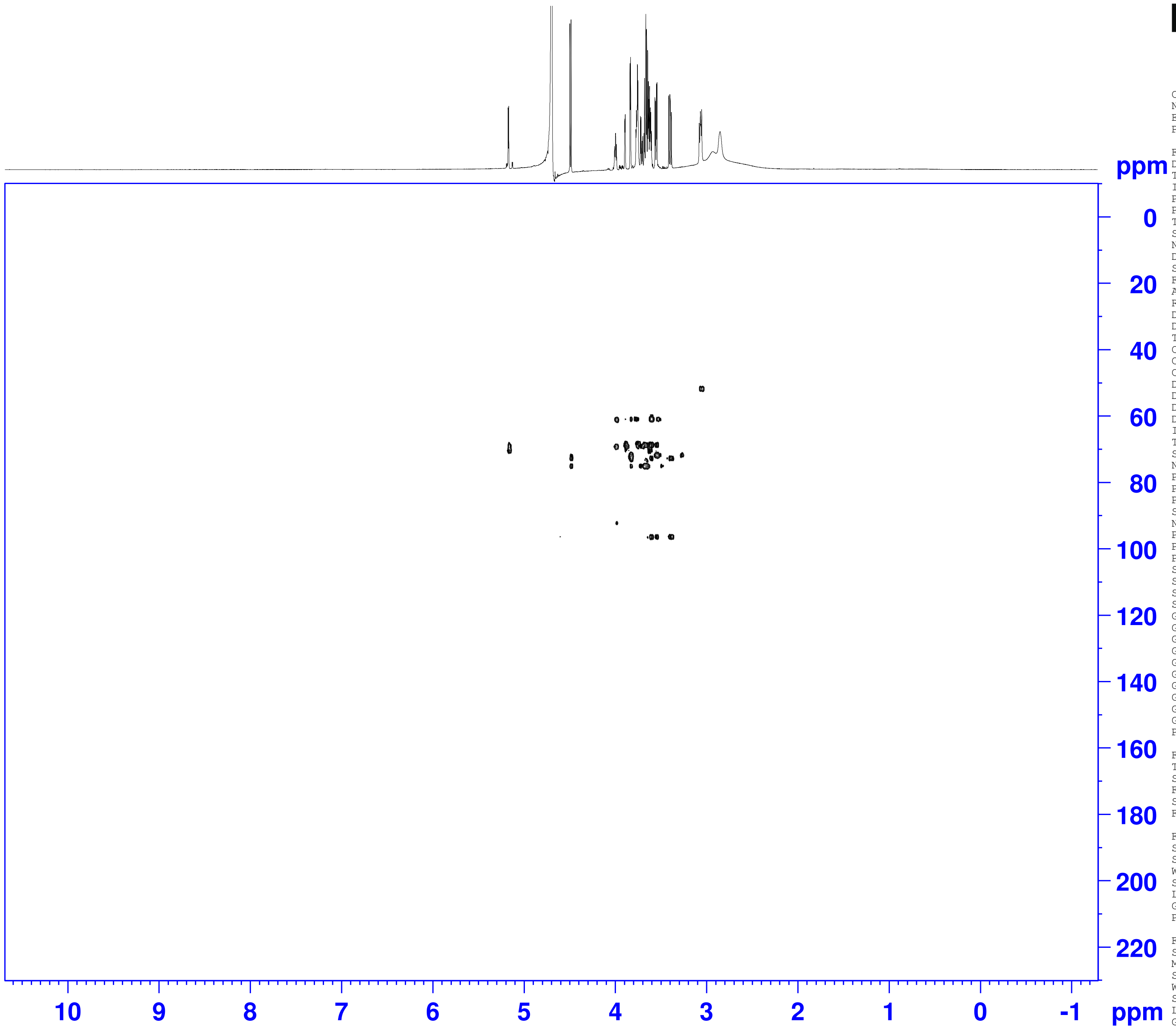


```
Current Data Parameters
NAME              2b_phoebe
EXPNO                    24
PROCNO                    1

F2 - Acquisition Parameters
Date_              20250819
Time                  18.49 h
INSTRUM               spect
PROBHD     Z150313_0001 (
PULPROG      hmbcetgpl3nd
TD                     4096
SOLVENT                 D2O
NS                        8
DS                       32
SWH                7183.908 Hz
FIDRES             3.507768 Hz
AQ                0.2850816 sec
RG                   190.75
DW                   69.600 usec
DE                    20.00 usec
TE                    298.1 K
CNST6           120.0000000
CNST7           170.0000000
CNST13            8.0000000
D0               0.00000300 sec
D1               2.00000000 sec
D6               0.06250000 sec
D16              0.00020000 sec
IN0              0.00001380 sec
TDav                      1
SFO1            599.7728189 MHz
NUC1                     1H
P1                     7.07 usec
P2                    14.14 usec
PLW1             7.80000019 W
SFO2            150.8288758 MHz
NUC2                    13C
P3                    12.20 usec
P24                 2000.00 usec
PLW2            90.00000000 W
SPNAM[7]       Crp60comp.4
SPOAL7                0.500
SPOFFS7                   0 Hz
SPW7            20.46699905 W
GPNAM[1]        SMSQ10.100
GPZ1                  80.00 %
GPNAM[3]        SMSQ10.100
GPZ3                  14.00 %
GPNAM[4]        SMSQ10.100
GPZ4                  -8.00 %
GPNAM[5]        SMSQ10.100
GPZ5                  -4.00 %
GPNAM[6]        SMSQ10.100
GPZ6                  -2.00 %
P16                 1000.00 usec

F1 - Acquisition parameters
TD                      256
SFO1               150.8289 MHz
FIDRES           283.061584 Hz
SW                  240.218 ppm
FnMODE        Echo-Antiecho

F2 - Processing parameters
SI                     4096
SF              599.7700000 MHz
WDW                    SINE
SSB                       4
LB                        0 Hz
GB                        0
PC                     1.40

F1 - Processing parameters
SI                     1024
MC2          echo-antiecho
SF              150.8122865 MHz
WDW                   QSINE
SSB                       2
LB                        0 Hz
GB                        0
```
