# Supplementary material for: Discovery and characterization of a nickel enzyme family that catalyses intermolecular hydride shuttling
Source: Nat Chem. 2026 Jun 17;18(9):1582–92. doi: 10.1038/s41557-026-02184-9 (PMC13524815; doi:10.1038/s41557-026-02184-9)
Supplement: Supplementary file 8 [file 41557_2026_2184_MOESM7_ESM.zip › SupplementaryFigure22_NphT_D-rib_NMR/SuppFig22_D-ribose_-NPMN/14/pdata/1/email_Phoebe_D-ribo_5b-redo_14_1.pdf]

HC_hmbc.UW D2O /home/zsemersky/av600 zsemersky 23

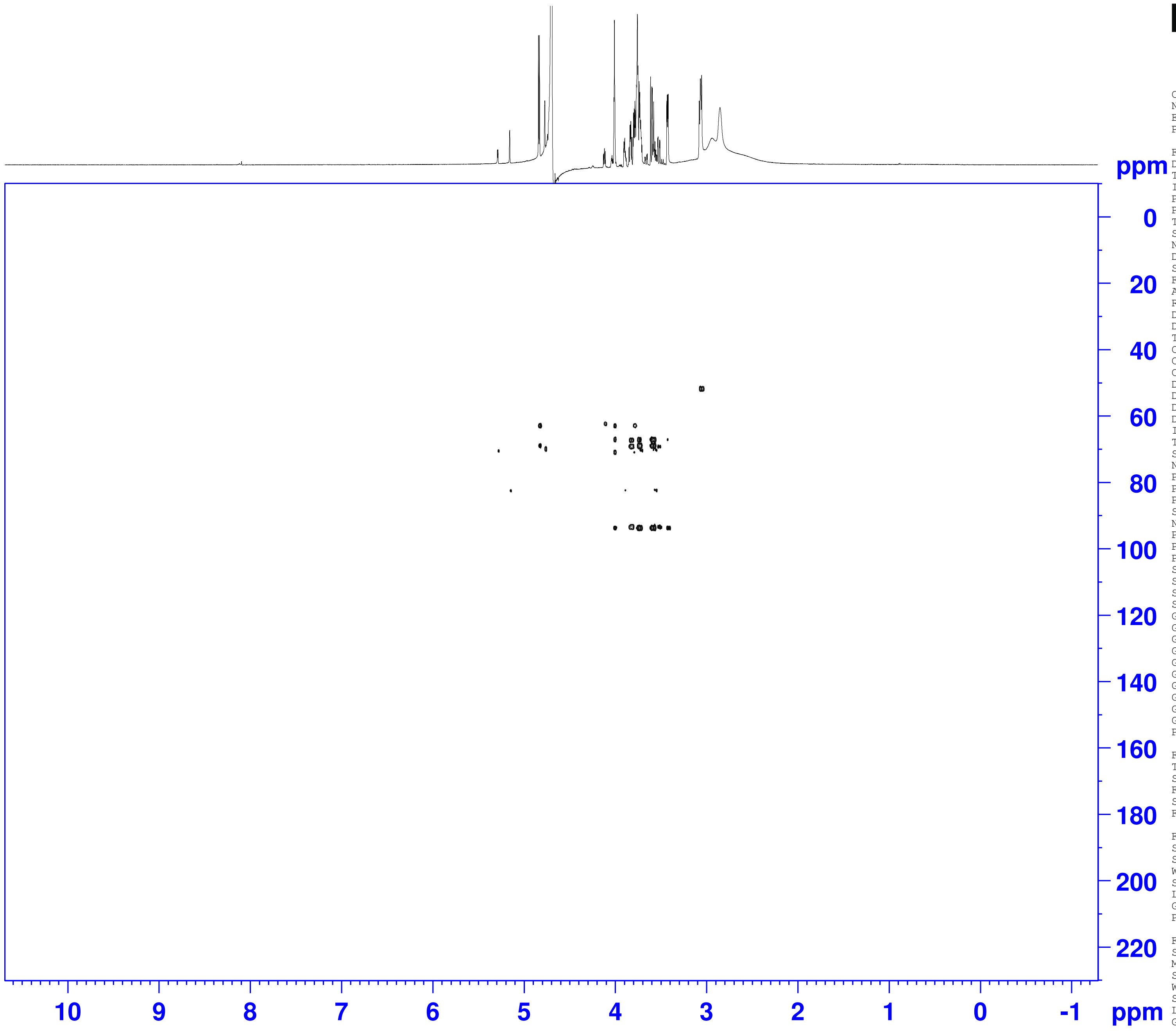

Current Data Parameters

NAME	Phoebe_D-ribo_5b-redo	
EXPNO	14	
PROCNO	1	

F2 - Acquisition Parameters

Date_	20250820	
Time	22.33	h
INSTRUM	spect	
PROBHD	Z150313_0001 (	
PULPROG	hmbcetgpl3nd	
TD	4096	
SOLVENT	D2O	
NS	8	
DS	32	
SWH	7183.908	Hz
FIDRES	3.507768	Hz
AQ	0.2850816	sec
RG	190.75	
DW	69.600	usec
DE	20.00	usec
TE	298.1	K
CNST6	120.0000000	
CNST7	170.0000000	
CNST13	8.0000000	
D0	0.00000300	sec
D1	2.00000000	sec
D6	0.06250000	sec
D16	0.00020000	sec
IN0	0.00001380	sec
TDav	1	
SFO1	599.7728189	MHz
NUC1	1H	
P1	7.07	usec
P2	14.14	usec
PLW1	7.80000019	W
SFO2	150.8288758	MHz
NUC2	13C	
P3	12.20	usec
P24	2000.00	usec
PLW2	90.00000000	W
SPNAM[7]	Crp60comp.4	
SPOAL7	0.500	
SPOFFS7	0	Hz
SPW7	20.46699905	W
GPNAM[1]	SMSQ10.100	
GPZ1	80.00	%
GPNAM[3]	SMSQ10.100	
GPZ3	14.00	%
GPNAM[4]	SMSQ10.100	
GPZ4	-8.00	%
GPNAM[5]	SMSQ10.100	
GPZ5	-4.00	%
GPNAM[6]	SMSQ10.100	
GPZ6	-2.00	%
P16	1000.00	usec

F1 - Acquisition parameters

TD	256	
SFO1	150.8289	MHz
FIDRES	283.061584	Hz
SW	240.218	ppm
FnMODE	Echo-Antiecho	

F2 - Processing parameters

SI	4096	
SF	599.7700000	MHz
WDW	SINE	
SSB	4	
LB	0	Hz
GB	0	
PC	1.40	

F1 - Processing parameters

SI	1024	
MC2	echo-antiecho	
SF	150.8122865	MHz
WDW	QSINE	
SSB	2	
LB	0	Hz
GB	0	
