# Supplementary material for: Discovery and characterization of a nickel enzyme family that catalyses intermolecular hydride shuttling
Source: Nat Chem. 2026 Jun 17;18(9):1582–92. doi: 10.1038/s41557-026-02184-9 (PMC13524815; doi:10.1038/s41557-026-02184-9)
Supplement: Supplementary file 8 [file 41557_2026_2184_MOESM7_ESM.zip › SupplementaryFigure22_NphT_D-rib_NMR/SuppFig22_D-ribose_-NPMN/12/pdata/1/email_Phoebe_D-ribo_5b-redo_12_1.pdf]

HH_cosy.UW D2O /home/zsemersky/av600 zsemersky 23

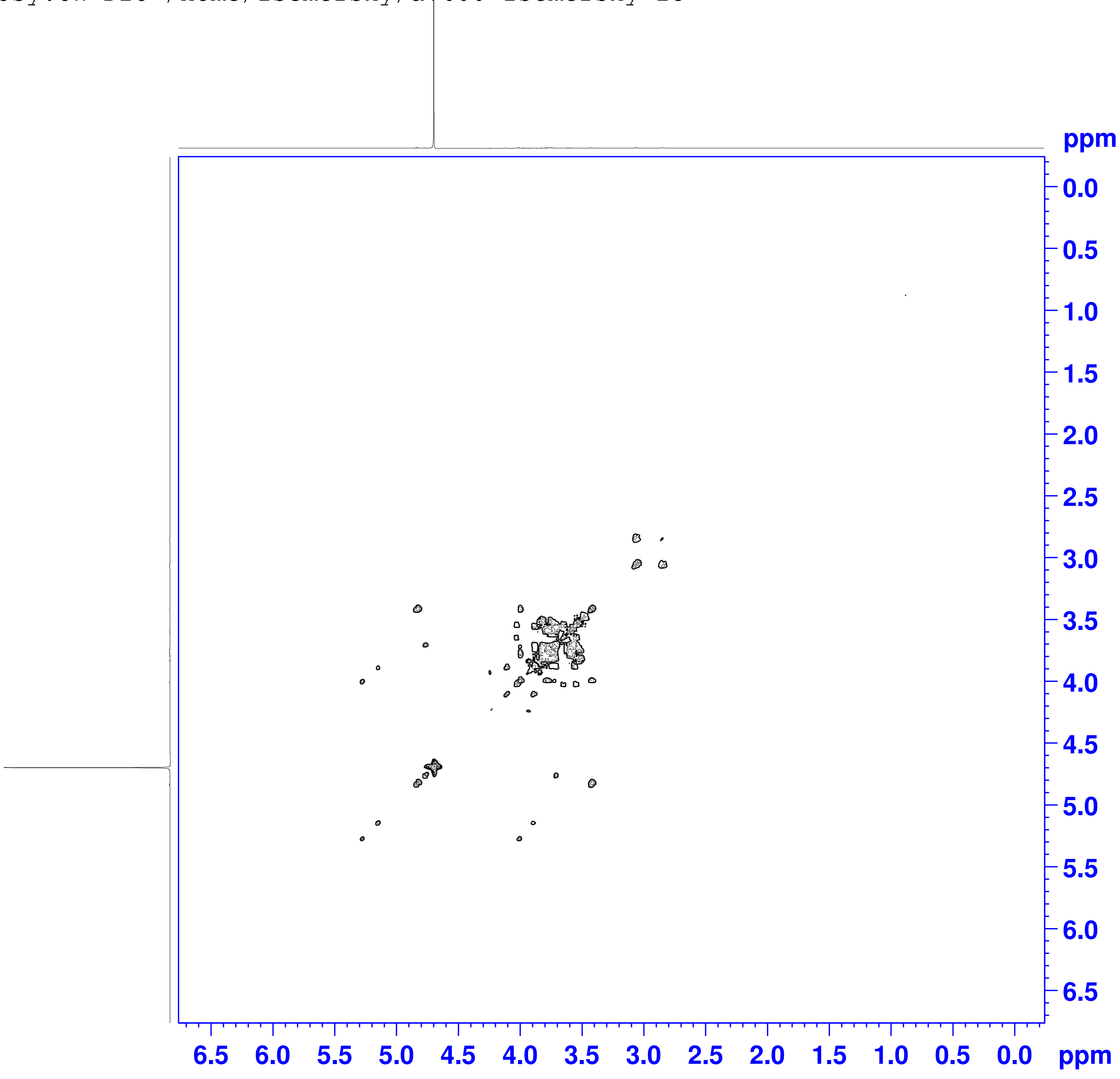

Current Data Parameters
NAME Phoebe_D-ribo_5b-redo
EXPNO 12
PROCNO 1

F2 - Acquisition Parameters
Date_ 20250820
Time 20.30 h
INSTRUM spect
PROBHD Z150313_0001 (
PULPROG cosygp45ppqf.UW
TD 2048
SOLVENT D2O
NS 4
DS 32
SWH 4201.681 Hz
FIDRES 4.103204 Hz
AQ 0.2437120 sec
RG 34.52
DW 119.000 usec
DE 20.00 usec
TE 298.1 K
D0 0.00000300 sec
D1 1.89882898 sec
D11 0.03000000 sec
D12 0.00002000 sec
D13 0.00000400 sec
D16 0.00020000 sec
IN0 0.00023800 sec
TDav 1
SFO1 599.7719558 MHz
NUC1 1H
P0 7.07 usec
P1 7.07 usec
P17 2500.00 usec
PLW1 7.80000019 W
PLW10 0.62380999 W
GPNAM[1] SMSQ10.100
GPZ1 10.00 %
P16 1000.00 usec

F1 - Acquisition parameters
TD 256
SFO1 599.772 MHz
FIDRES 32.825630 Hz
SW 7.005 ppm
FnMODE QF

F2 - Processing parameters
SI 1024
SF 599.7700000 MHz
WDW QSINE
SSB 0
LB 0 Hz
GB 0
PC 1.40

F1 - Processing parameters
SI 1024
MC2 QF
SF 599.7700000 MHz
WDW QSINE
SSB 0
LB 0 Hz
GB 0
