# Supplementary material for: Discovery and characterization of a nickel enzyme family that catalyses intermolecular hydride shuttling
Source: Nat Chem. 2026 Jun 17;18(9):1582–92. doi: 10.1038/s41557-026-02184-9 (PMC13524815; doi:10.1038/s41557-026-02184-9)
Supplement: Supplementary file 8 [file 41557_2026_2184_MOESM7_ESM.zip › SupplementaryFigure22_NphT_D-rib_NMR/SuppFig22_D-ribose_+NPMN/57/pdata/1/email_Phoebe_2588_5A_57_1.pdf]

HH_cosy.UW D2O /home/zsemersky/av600 zsemersky 17

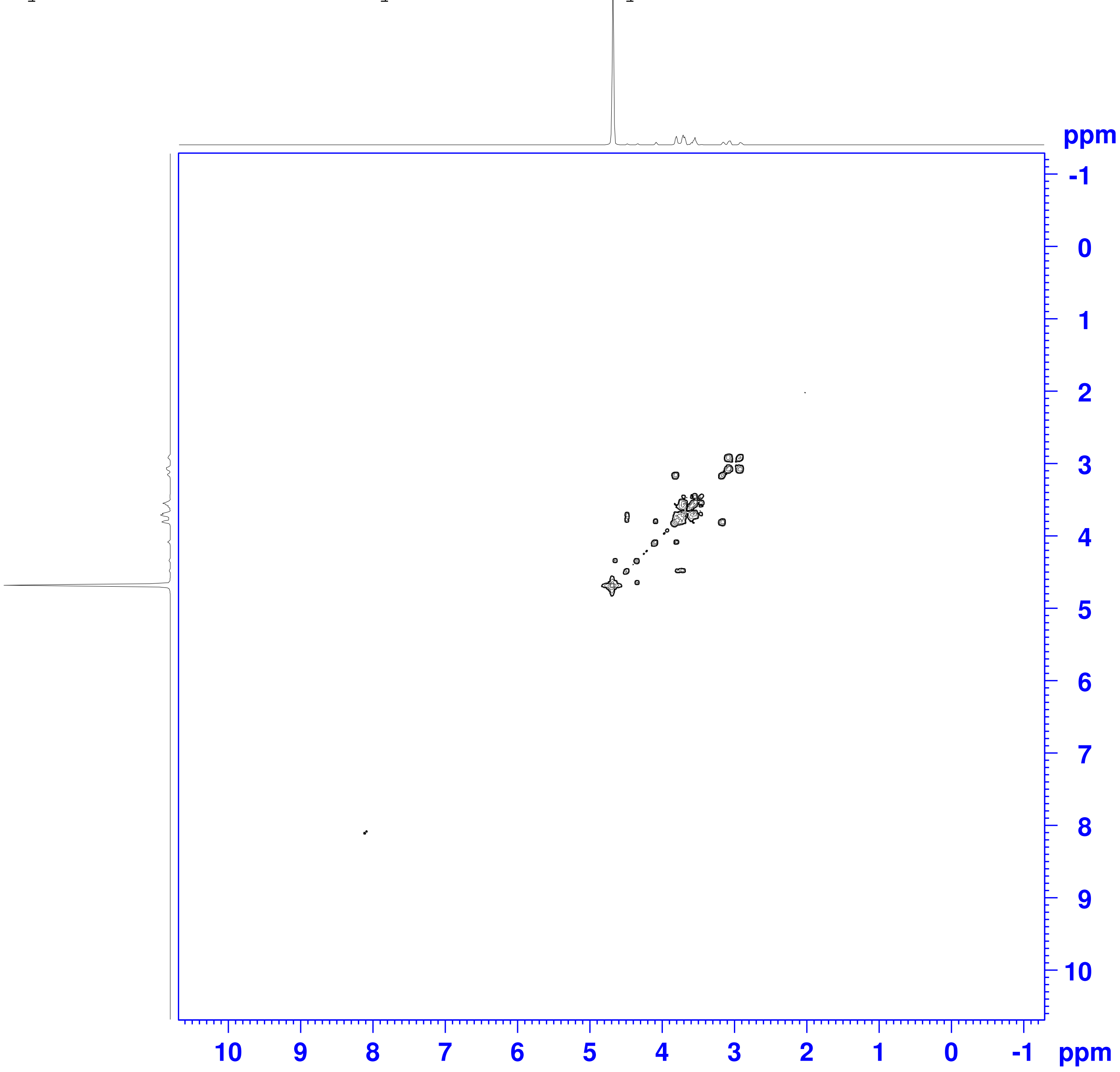


```
Current Data Parameters
NAME       Phoebe_2588_5A
EXPNO                  57
PROCNO                  1

F2 - Acquisition Parameters
Date_            20250815
Time                 1.54 h
INSTRUM             spect
PROBHD     Z150313_0001 (
PULPROG    cosygp45ppqf.UW
TD                   2048
SOLVENT               D2O
NS                      4
DS                     32
SWH              7183.908 Hz
FIDRES           7.015535 Hz
AQ              0.1425408 sec
RG                   38.8
DW                 69.600 usec
DE                  20.00 usec
TE                  298.1 K
D0             0.00000300 sec
D1             2.00000000 sec
D11            0.03000000 sec
D12            0.00002000 sec
D13            0.00000400 sec
D16            0.00020000 sec
IN0            0.00013920 sec
TDav                    1
SFO1          599.7728189 MHz
NUC1                   1H
P0                   7.07 usec
P1                   7.07 usec
P17               2500.00 usec
PLW1           7.80000019 W
PLW10          0.62380999 W
GPNAM[1]       SMSQ10.100
GPZ1                10.00 %
P16               1000.00 usec

F1 - Acquisition parameters
TD                    256
SFO1             599.7728 MHz
FIDRES          56.124283 Hz
SW                 11.978 ppm
FnMODE                 QF

F2 - Processing parameters
SI                   1024
SF            599.7700000 MHz
WDW                 QSINE
SSB                     0
LB                      0 Hz
GB                      0
PC                   1.40

F1 - Processing parameters
SI                   1024
MC2                    QF
SF            599.7700000 MHz
WDW                 QSINE
SSB                     0
LB                      0 Hz
GB                      0
```
