# Supplementary material for: Discovery and characterization of a nickel enzyme family that catalyses intermolecular hydride shuttling
Source: Nat Chem. 2026 Jun 17;18(9):1582–92. doi: 10.1038/s41557-026-02184-9 (PMC13524815; doi:10.1038/s41557-026-02184-9)
Supplement: Supplementary file 8 [file 41557_2026_2184_MOESM7_ESM.zip › SupplementaryFigure22_NphT_D-rib_NMR/SuppFig22_D-ribose_+NPMN/59/pdata/1/email_Phoebe_2588_5A_59_1.pdf]

HC_hmbc.UW D2O /home/zsemersky/av600 zsemersky 17

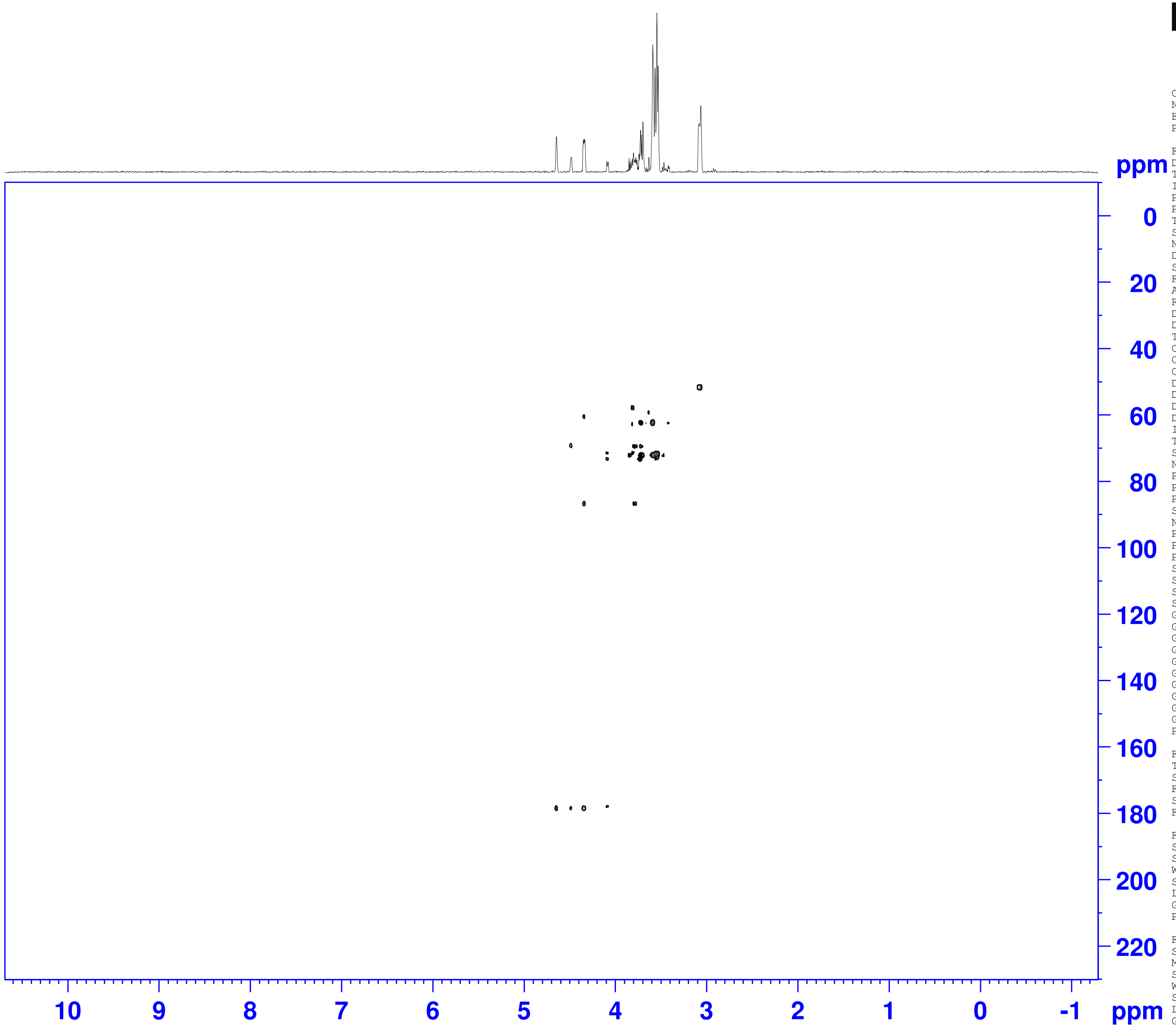


```
Current Data Parameters
NAME      Phoebe_2588_5A
EXPNO                 59
PROCNO                 1

F2 - Acquisition Parameters
Date_           20250815
Time                3.57 h
INSTRUM            spect
PROBHD   Z150313_0001 (
PULPROG   hmbcetgpl3nd
TD                  4096
SOLVENT              D2O
NS                     8
DS                    32
SWH             7183.908 Hz
FIDRES          3.507768 Hz
AQ             0.2850816 sec
RG                190.75
DW                69.600 usec
DE                 20.00 usec
TE                 298.1 K
CNST6        120.0000000
CNST7        170.0000000
CNST13         8.0000000
D0            0.00000300 sec
D1            2.00000000 sec
D6            0.06250000 sec
D16           0.00020000 sec
IN0           0.00001380 sec
TDav                   1
SFO1         599.7728189 MHz
NUC1                  1H
P1                  7.07 usec
P2                 14.14 usec
PLW1          7.80000019 W
SFO2         150.8288758 MHz
NUC2                 13C
P3                 12.20 usec
P24              2000.00 usec
PLW2         90.00000000 W
SPNAM[7]     Crp60comp.4
SPOAL7             0.500
SPOFFS7                0 Hz
SPW7         20.46699905 W
GPNAM[1]       SMSQ10.100
GPZ1               80.00 %
GPNAM[3]       SMSQ10.100
GPZ3               14.00 %
GPNAM[4]       SMSQ10.100
GPZ4               -8.00 %
GPNAM[5]       SMSQ10.100
GPZ5               -4.00 %
GPNAM[6]       SMSQ10.100
GPZ6               -2.00 %
P16              1000.00 usec

F1 - Acquisition parameters
TD                   256
SFO1            150.8289 MHz
FIDRES        283.061584 Hz
SW               240.218 ppm
FnMODE      Echo-Antiecho

F2 - Processing parameters
SI                  4096
SF           599.7700000 MHz
WDW                 SINE
SSB                    4
LB                     0 Hz
GB                     0
PC                  1.40

F1 - Processing parameters
SI                  1024
MC2        echo-antiecho
SF           150.8122865 MHz
WDW                QSINE
SSB                    2
LB                     0 Hz
GB                     0
```
